# Identification of transcription factors potentially involved in human adipogenesis in vitro

**DOI:** 10.1002/mgg3.269

**Published:** 2017-03-03

**Authors:** Melvin Anyasi Ambele, Michael Sean Pepper

**Affiliations:** ^1^ Department of Immunology and Institute for Cellular and Molecular MedicineSAMRC Extramural Unit for Stem Cell Research and TherapyFaculty of Health SciencesUniversity of PretoriaPretoriaSouth Africa

**Keywords:** Adipocyte differentiation, human adipogenesis, human adipose‐derived stromal cells, obesity, transcription factor

## Abstract

**Background:**

Increased adiposity in humans leads to obesity, which is a major risk factor for cardiovascular disease, type 2 diabetes, and cancer. We previously conducted an extensive unbiased in vitro transcriptomic analysis of adipogenesis, using human adipose‐derived stromal cells (ASCs). Here, we have applied computational methods to these data to identify transcription factors (TFs) that constitute the upstream gene regulatory networks potentially, driving adipocyte formation in human ASCs.

**Methods:**

We used Affymetrix Transcription Analysis Console™ v3.0 for calculating differentially expressed genes. MATCH™ and F‐MATCH™ algorithms for TF identification. STRING v10 to predict protein–protein interactions between TFs.

**Results:**

A number of TFs that were reported to have a significant role in adipogenesis, as well as novel TFs that have not previously been described in this context, were identified. Thus, 32 upstream TFs were identified, with most belonging to the C2H2‐type zinc finger and HOX families, which are potentially involved in regulating most of the differentially expressed genes observed during adipocyte differentiation. Furthermore, 17 important upstream TFs were found to have increased regulatory effects on their downstream target genes and were consistently up‐regulated during the differentiation process. A strong hypothetical functional interaction was observed among these TFs, which supports their common role in the downstream regulation of gene expression during adipogenesis.

**Conclusion:**

Our results support several previous findings on TFs involved in adipogenesis and thereby validate the comprehensive and systematic in silico approach described in this study. In silico analysis also allowed for the identification of novel regulators of adipocyte differentiation.

## Introduction

Adipocyte hypertrophy (increased adipocyte size) and hyperplasia (increased number of adipocytes) both lead to an increase in adiposity, which in turn gives rise to obesity. Obese individuals are at a greater risk of developing cardiovascular disease, type 2 diabetes, and cancer than their nonobese counterparts (Stephens 2012). Understanding the mechanisms that lead to adipocyte hypertrophy and/or hyperplasia at the transcriptome level could provide valuable information to combat the increase in obesity worldwide. The regulation of gene expression is tightly controlled by multiple dedicated components from diverse molecular circuits, and transcription factors (TFs) are considered critical in a variety of biological processes such as cell differentiation, developmental processes and the response to external and internal stimuli (Farmer 2006). The differentiation of a preadipocyte into a mature adipocyte is controlled by a large network of TFs that work together, directly or indirectly, to regulate the expression of hundreds of downstream protein‐coding genes and long noncoding RNAs (lncRNAs) that are responsible for adipogenesis and the phenotypic characteristics of mature adipocytes (Farmer 2006; Lefterova and Lazar 2009; Stephens 2012; Sun et al. 2013).

The gene expression patterns observed in microarray experiments are largely determined by upstream TFs acting directly or indirectly on gene regulatory regions, causing either the up‐ or down‐regulation of gene expression depending on the perceived biological signal. *PPARG* (601487) and *CEBPA* (116897) are key TFs at the center of adipocyte differentiation, and they play a pivotal role in determining the fate of differentiating preadipocytes (Lefterova and Lazar 2009). Cells deficient in Pparg do not express any of the known phenotypic characteristics of mature adipocytes, highlighting Pparg as a master regulator of adipogenesis (Rosen et al. 2002). In contrast, cells lacking Cebpa are still capable of differentiating into adipocytes but are insulin resistant (Wu et al. 1999). Recent studies in knockout mice (Cebpa^−/−^ and Pparg^−/−^) revealed a distinct regulatory role for Cebpa and Pparg in the maturation of both embryonic and adult adipocytes (Wang et al. 2015). In adults, *CEBPA* was shown to be essential for adipogenesis in white adipose tissue in muscle, whereas terminal embryonic adipogenesis is *PPARG* dependent (Wang et al. 2015). In addition, many other TFs such as *EBF1* (164343), *KLF4* (602,253), *KLF5* (602903), *KLF6* (602053), *KLF15* (606465), *EGR2* (129010), *CEBPA* (116897), *CEBPB*,* CEBPG* (138972), *SREBP1* (184956), *ARNTL* (602550), and *PPARG* have been shown to have a positive effect on adipogenesis in preadipocytes, whereas *GATA2* (164343), *GATA3* (131320), *KLF2* (602016), *KLF3* (609392), *IRF3* (603734), and *IRF4* (601900) are reported to have a negative effect on adipogenesis (Lefterova and Lazar 2009).

To better understand adipogenesis in humans, we have used human adipose‐derived stromal/stem cells (ASCs) to study adipogenic differentiation at multiple time points, ranging from early‐ to late‐stage adipogenesis, to identify novel molecular players that could contribute to our understanding of human adipogenesis across the entire process (Ambele et al. 2016). A well‐designed 21‐day time‐course of in vitro adipogenic differentiation, using ASCs at a relatively high passage number, in which the rate of differentiation was accurately determined (Durandt et al. 2016), previously reported the differential expression of genes that encode TFs known to play a role in adipogenesis, such as *PPARG*,* CEBP's*,* FOXO1A* (136533), *KLF15*,* FOXD1* (601091), *E2F7* (612046), *FOXP1* (605515), *HMGA1* (600701), *KLF2*,* JUN* (165160), *KLF16* (606139), *GATA6* (601656), *KLF12* (607531), *FOXM1* (602341) and *STAT4* (600558) (Ambele et al. 2016). In this study, we sought to employ computational methods to conduct an in silico analysis to identify the upstream candidate regulators that might constitute the regulatory network of TFs potentially responsible for driving changes in the expression of these differentially expressed downstream TFs and other genes during adipogenesis in ASCs. Furthermore, we wished to identify TFs with increased regulatory effects on gene expression over time based on the increased abundance of their binding sites in genes that were consistently up‐regulated in a differentiation‐dependent manner. Identification of these upstream candidate TFs will provide a unique resource for further investigation through functional studies that will provide greater insight into our understanding of adipogenesis in humans and could potentially lead to the discovery and development of anti‐obesity drugs.

## Material and Methods

### Ethical compliance

All participating subjects provided written informed consent, and the study was approved by the Faculty of Health Sciences Research Ethics Committee of the University of Pretoria, South Africa (Ethics Reference No.: 421/2013).

### Microarray gene expression experiment

The microarray data used for this study were previously reported by Ambele et al. (GEO accession number GSE77532) (Ambele et al. 2016). Affymetrix Transcription Analysis Console™ v3.0 downloaded from Affymetrix website ([www.affymetrix.com] 3420 Central Expressway, Santa Clara, CA) was used to calculate the fold change in each probe set or transcript cluster identifier number from the downloaded CHP files and mapped to the corresponding gene. A fold change of ≥2 and ≤−2 with a *P* < 0.05 was applied to select differentially expressed genes (DEGs). The use of a twofold expression cutoff in this study as opposed to a fourfold change in our previous publication (Ambele et al. 2016) has been chosen to provide a large gene list which is inclusive of all possible DEGs as determined by microarray experiments for statistical motif enrichment analysis.

### Software

We used databases and computational algorithms provided by licensed software, namely, TRANSFAC^®^ by BIOBASE GmbH (www.biobase.de). The TRANSFAC^®^ (version 2014.4) database contains a position weight matrix (PWM) library that contains the consensus sequences or motifs of the transcription factor‐binding sites (TFBS) in the promoter regions of all eukaryotic genes and all known TFs.

### In silico prediction of TFBS in the promoter regions of DEGs

The MATCH™ tool (Kel et al. 2003) was used to predict TFBS in the promoter regions of DEGs on the different days of adipogenesis. The analysis was performed using a promoter window of 2000 bp upstream and 1000 bp downstream of each transcription start site (TSS). The PWMs used were from the Matrix library found in the TRANSFAC matrix table (release 2015.1). The profile list of PWMs used was vertebrate_non_redundant_minFP (minimize false positive). High‐quality matrices for individual TFBS that met the specified minFP cutoff values were selected. Furthermore, we filtered only for matrices with sites/sequence values ≥1.5. The sites per sequence value represent the average number of binding sites predicted per promoter sequence for a selected PWM within the query gene set.

### In silico prediction of TFBS overrepresented in one gene set (query set) compared to another (background gene set)

The F‐MATCH™ tool was used to predict TFBS that were overrepresented in the promoter regions of a query set (or Yes‐set) compared with a background gene set (or No‐set). We searched for a promoter window of 2000 bp upstream and 1000 bp downstream of each TSS. The profile list of PWMs used is from vertebrate_non_redundant_minFP, and high‐quality matrices for the individual TFBS that met the specified minFP cutoff values at *P *=* *0.01 were considered. Matrices with Yes/No values ≥2 were considered. Ratios yes/no represent the relative abundance of a selected PWM in the query gene set compared to the background gene set. Therefore, only TFs with a twofold abundance of their binding sites in the Yes set compared to the No set was selected.

### Functional protein association networks (protein–protein interactions) of TFs

STRING v10 (Szklarczyk et al. 2015) was used to determine known and predicted interactions among TFs at the protein level, as well as to predict other functional proteins that interact with these TFs in *Homo sapiens*. This analysis is important because proteins that interact with each other are likely to be involved in common biological processes or functions. Information on functional interactions and the identification of other functional interacting partners was obtained from gene fusion, co‐expression, neighborhood, co‐occurrence, database, experiments, text mining, and homology data. The number of interacting partners in the network analysis was limited to four.

## Results and Discussion

### Gene expression profile during adipogenic differentiation in ASCs

**Figure 1:**
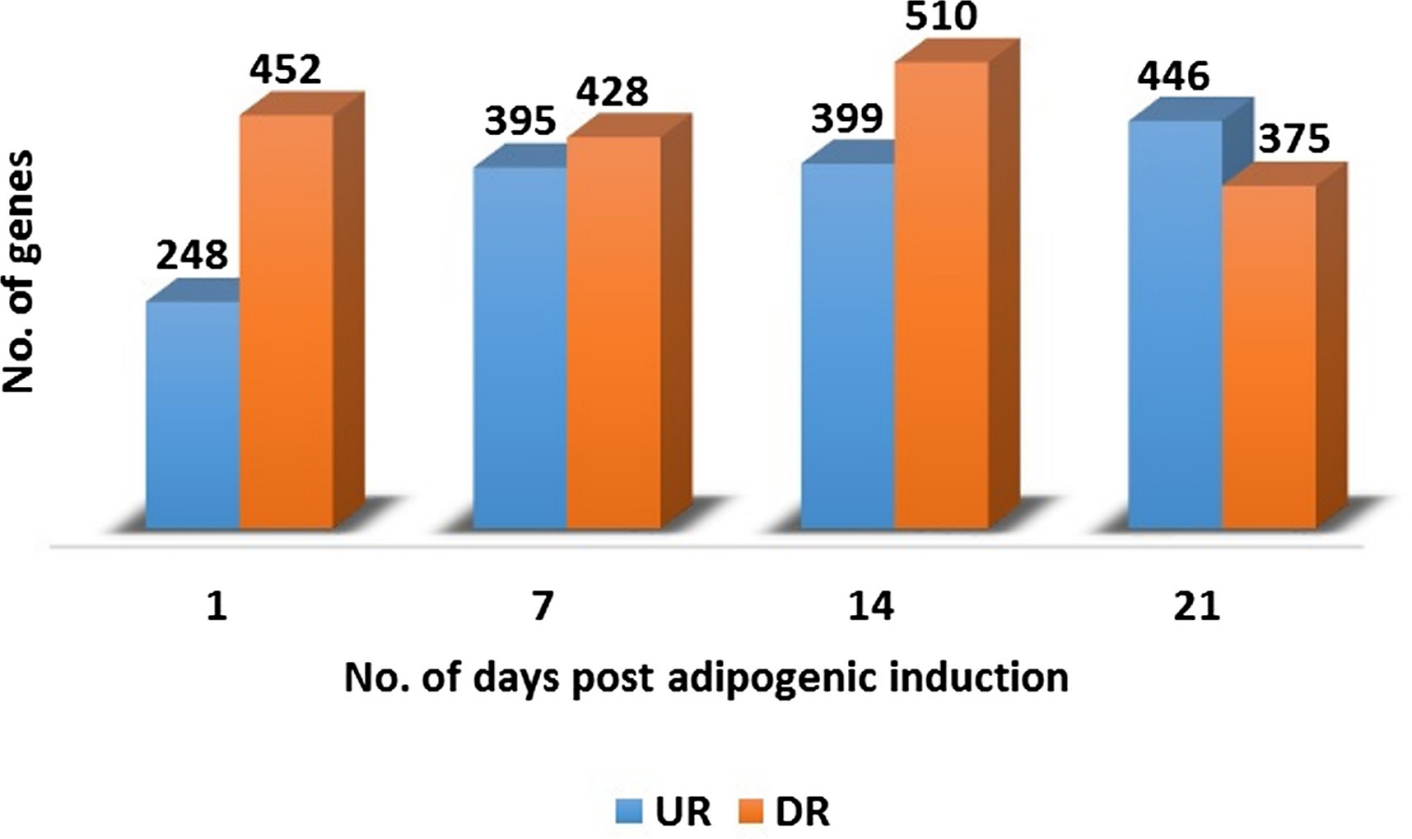
Gene expression on different days during ASC adipogenic differentiation. The number of genes significantly differentially expressed during ASC differentiation on D1, D7, D14 and D21 were 700, 823, 909 and 821 respectively.

The number of genes that were significantly up‐ and down‐regulated was 248 and 452 on D1, 395 and 428 on D7, 399 and 510 on D14, and 446 and 378 on D21 (Fig. 1). A complete list of the DEGs on the different days is provided in Tables S1 to S4. A large initial increase in the number of significantly up‐regulated genes (248 to 395) was observed from D1 to D7, after which (D14 to D21) the number of genes remained fairly constant (Fig. 1). The initial increase in the number of up‐regulated genes from D1 to D7 might indicate an important early window period (within one week of adipogenic induction) that is involved in priming ASCs for differentiation.

### TFs potentially responsible for the observed day‐specific differentially expressed gene profiles

MATCH™ analysis identified 39 different PWMs that were enriched in the promoter regions of up‐ and down‐regulated genes on D1. These PWMs of TFBS represent the TFs responsible for the observed gene expression profile in ASCs on D1 of adipocyte differentiation. Of the 39 PWMs, 35 were common to both the up‐ and down‐regulated gene sets. V$RFX1_01 and V$HBP1_03 are matrices identified to be enriched only in the promoter regions of up‐regulated genes and represent TFBS for the *RFX1* (600006) (regulatory factor X, 1) and *HBP1* (616714) (high‐mobility group‐box protein 1) TFs, respectively. Conversely, V$ZFP206_01, which represents a TFBS for *ZFP206*, was enriched only in the promoter regions of down‐regulated genes. This TF has been shown to regulate pluripotency in embryonic stem cells by activating Oct4 and Nanog transcription (Wang et al. 2007).

The promoter regions of genes that were up‐ and down‐regulated on D7 were enriched for 41 and 38 PWMs, respectively, of which 35 PWMs were common to both the up‐ and down‐regulated gene sets. V$HBP1_03, V$RFX1_01, V$NKX25_Q6 and V$POU6F1_02 represent the binding sites for *HBP1*,* RFX1*,* NKX2‐5* (600584), and *POU6F1*, respectively, which were enriched only in the promoter sequences of the up‐regulated gene sets. V$HBP1_03 and V$RFX1_01 were previously identified in the D1 up‐regulated genes. *NKX2‐5* is a cardiac‐specific homeobox TF that functions in adipogenesis in arrhythmogenic cardiomyopathy, which is a clinical condition where fibro‐adipocytes replace cardiac myocytes (Liu et al. 2009; Chen et al. 2014). Conversely, V$FREAC3_01, which is the binding site for *FOXC1* (601090), was enriched only in the D7 down‐regulated genes. The promoter regions of the D14 up‐ and down‐regulated gene sets were enriched for 40 and 36 PWMs, respectively, of which 32 PWMs were common to both the up‐ and down‐regulated gene sets. Five matrices, V$HBP1_03, V$NKX25_Q6, V$RFX1_01, V$TBX5_01 and V$CDPCR1_01, which correspond to the binding sites for *HBP1*,* NKX2‐5*,* RFX1*,* TBX5* (601620), and *CDP/CR1*, respectively, were enriched only in the promoter regions of the up‐regulated genes, whereas V$FREAC3_01 was again enriched only in the D7 down‐regulated genes.

Finally, the promoter regions of the up‐ and down‐regulated genes on D21 were enriched for 40 and 39 PWMs, respectively, of which 34 were common to both the up‐ and down‐regulated gene sets. V$CDPCR1_01, V$HBP1_03, V$RFX1_01, and V$TBX5_01 were enriched only in the up‐regulated gene sets, whereas V$FPM315_01, V$FREAC3_01 and V$POU6F1_02 were enriched only in the down‐regulated gene sets. All these PWMs have already been identified in the promoter regions of DEGs during adipogenesis, with the exception of V$FPM315_01, which represents a binding site for *ZNF263* (604191). This TF has not previously been shown to play a role in adipogenesis. V$GEN_INI_B and V$MUSCLEINI_B are matrices enriched in the promoter regions of all DEGs from D1 to D21 that did not represent binding sites for any known TFs.

MATCH™ analysis therefore identified 35, 35, 32, and 34 TFs whose binding sites were enriched in the promoter regions of both up‐ and down‐regulated genes on D1, D7, D14, and D21, respectively. The enrichment of binding sites for *HBP1* and *RFX1* in the promoter regions of the up‐regulated genes from D1 to 21 suggests an important role for these TFs throughout adipogenesis. The siRNA or shRNA knockdown studies in 3T3‐L1 cells showed that Hbp1 is an important regulator of adipocyte differentiation (Shih et al. 1998). The fact that TFBS for *TBX5* were predominantly enriched in genes up‐regulated at D14 and D21, which coincides with the terminal differentiation and maturation of adipocytes, supports previous findings implicating *TBX5* as a regulator of adipocyte differentiation in abdominal subcutaneous adipose tissue (Pinnick et al. 2014). In contrast, *FOXC1‐*binding sites were found to be enriched specifically in the promoter regions of down‐regulated genes on D7 to D21 only.

### TFs potentially involved in the regulation of the majority of DEGs throughout ASC differentiation

To identify which TFs are potentially involved in regulating the majority of the observed DEGs during adipogenesis, we selected only TFs whose binding sites were enriched in both up‐ and down‐regulated gene sets on all days (D1 to D21) studied during in vitro adipogenesis. This analysis yielded 32 PWMs (Table 1) for TFBS that were enriched in all the significant DEGs throughout adipogenesis, suggesting an important role for these TFs in regulating the observed gene expression profile obtained during adipogenesis in ASCs. Interestingly, 10 and 4 of these TFs belong to the C2H2‐type zinc finger, and homeobox (HOX) families, respectively, which play an essential role in developmental processes, as well as in the pathogenesis of various diseases (Liu et al. 2015). In particular, HOX genes are known to play a crucial role in various stem cell differentiation processes, including ASC adipogenesis (Seifert 2015).

**Table 1:** Position weight matrix's of TFBS enriched in the promoter regions of all the significant DEGs from D1 to 21 of adipogenesis in ASCs. For each TF, the letter ‘P’, ‘N’, and ‘–’ represents positive, negative, and no reported regulatory effect on adipogenesis, respectively

Matrix	Consensus sequence	Factor family (TF)	Classification	Effect on diff.
*V$AHR_Q6*	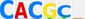	AHR	BHLH	N (Shimba et al. 2001; Shin et al. 2007)
*V$AP2ALPHA_03*	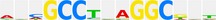	AP‐2alphaA (TFAP2A)	BHSH	N (Liu et al. 2009; Mitchell et al. 2006; Orso et al. 2010)
*V$CREBP1_01*	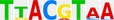	ATF‐2	BZIP	P (Lee et al. 2001; Maekawa et al. 2010)
*V$BBX_03*	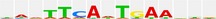	Bbx	HMG	P (Choi et al. 2014)
*V$BEN_01*	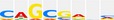	NACC2	BHLH	–
*V$CDX2_Q5_02*	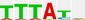	CDX‐2	HOX	P (Ochs‐Balcom et al. 2011)
*V$CHCH_01*	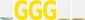	Churchill	CHCH	–
*V$CPBP_Q6*	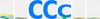	CPBP (KLF6)	ZFC2H2	P (Li et al. 2005)
*V$CRX_Q4_01*	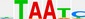	CRX	HOX	–
*V$DRI1_01*	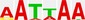	DRI1 (ARID3A)	ARID	N (Webb and Kincade 2010)
*V$EGR1_Q6*	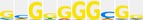	Egr‐1 (KROX24)	ZFC2H2	N (Boyle et al. 2009; Shen et al. 2011; Yu et al. 2011)
*V$ERALPHA_01*	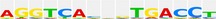	ER‐alpha (ESR1)	ZFC4‐NR	– (Nilsson et al. 2007)
*V$FAC1_01*	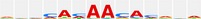	FAC1 (BPTF)	ZFPHD	–
*V$GKLF_Q4*	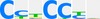	GKLF (KLF4)	ZFC2H2	P (Zeni Wu and Wang 2013)
*V$GMEB2_04*		GMEB2	SAND	–
*V$HMGIY_Q3*	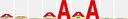	HMGIY (HMGA1)	ATHOOK	P (Esposito et al. 2009)
*V$IK_Q5_01*	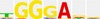	Ikaros (IKZF1)	ZFC2H2	P (Park and Pyo 2013)
*V$ING4_01*	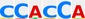	ING4	ZFPHD	–
*V$MAZ_Q6_01*	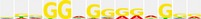	MAZ	ZFC2H2	– (Madeira et al. 2012)
*V$MYOGENIN_Q6_01*	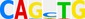	Myogenin (MYOG)	BHLH	–
*V$MZF1_Q5*	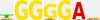	MZF‐1	ZFC2H2	–
*V$NFAT1_Q4*	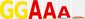	NF‐AT1	REL	P (Ho et al. 1998)
*V$P53_04*	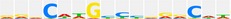	p53	p53	P/N (Molchadsky et al. 2013; Huang et al. 2014)
*V$PIT1_Q6_01*	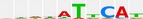	Pit‐1 (POU1F1)	HOX	– (Brandebourg et al. 2007; Ben‐Batalla et al. 2010)
*V$RNF96_01*	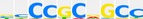	RNF96 (TRIM28)	ZFPHD	– (Youngson and Morris 2013)
*V$RREB1_01*	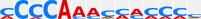	RREB‐1	ZFC2H2	–
*V$SP1_Q6_01*	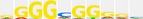	Sp1	ZFC2H2	N (Tang et al. 1999)
*V$SP100_04*	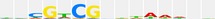	SP100 secondary motif	SAND	–
*V$SRY_Q6*	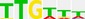	SRY	HMG	N (Sul 2009; Lowe et al. 2011)
*V$XVENT1_01*	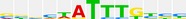	Xvent‐1	HOX	–
*V$ZFP161_04*	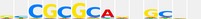	ZF5 secondary motif (ZFP161)	ZFC2H2	–
*V$ZNF333_01*	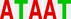	ZNF333	ZFC2H2	–

Of the 32 TFs identified in this study, some have previously been shown to have diverse roles in adipogenesis. Aryl hydrocarbon receptor (*AHR*) (600253) negatively regulates adipogenesis in 3T3‐L1 cells and mouse embryonic fibroblasts through the transcriptional regulation of its downstream target genes (Shimba et al. 2001; Shin et al. 2007). Sp1 negatively regulates 3T3‐L1 preadipocyte differentiation by binding to the *CEBPA* promoter prior to adipocyte differentiation, thereby preventing the binding and activation of *CEBPA* by *CEBPB* and *CEBPG* (Tang et al. 1999). ATF‐2 functions in adipocyte differentiation and fat storage by transcriptionally regulating the *PPARG* and *CEBPA* genes (Lee et al. 2001; Maekawa et al. 2010).

*BBX* plays an important regulatory role in dental pulp stem cell/progenitor differentiation (Choi et al. 2014), which suggests that further studies are warranted to uncover its possible regulatory role in adipocyte differentiation. *CDX2* (600297) has a positive effect on adipogenesis through its transcriptional regulation of the vitamin D receptor gene (Ochs‐Balcom et al. 2011). Klf6 promotes adipogenesis in 3T3‐L1 cells by inhibiting Dlk1/Pref‐1 (176290) expression through Hdac3 (Li et al. 2005). *ARID3A* (603265) is a novel regulator of pluripotency as described in US patent WO2010009015 A2, and cells lacking this TF differentiate into nerve cells, endothelial cells, and adipocytes (Webb and Kincade 2010). Egr‐1 negatively regulates adipocyte differentiation and induces systematic insulin resistance when overexpressed in murine epididymal fat (Boyle et al. 2009; Shen et al. 2011; Yu et al. 2011). The role of *ESR1* (133430) in lipolysis or adipogenesis is unknown (Nilsson et al. 2007); well‐designed functional studies are therefore required to elucidate its possible regulatory role in human adipogenesis. Kfl4 knockdown in 3T3‐L1 cells significantly reduces lipid formation and aP2 and Pparg expression (Zeni Wu and Wang 2013), suggesting a positive role in adipogenesis. *HMGA1* promotes adipocyte differentiation through its interaction with retinoblastoma protein (Esposito et al. 2009). *Ikaros* (603023) promotes adipocyte differentiation in 3T3‐L1 cells by suppressing the expression of c‐Myc, and knocking down this TF impairs lipid droplet accumulation in 3T3‐L1 cells (Park and Pyo 2013). *MAZ* (600999) has been identified as one of the most significant transcriptional regulators of gene expression in long‐term cultures of human mesenchymal stem cells (Madeira et al. 2012), which validates its identification in this study since the microarray data in our previous study (Ambele et al. 2016) that were used here were generated from relatively late passage ASCs. Nfat1 (Nfatc2) (600490) transcriptionally activates the adipocyte‐specific gene aP2 to mediate adipocyte differentiation in 3T3‐L1 cells (Ho et al. 1998). The effect of *TP53* (191170) on adipocyte differentiation is cell type dependent; its overexpression in white adipocytes from both humans and mice suppresses adipogenesis, but enhances brown adipocyte differentiation (Molchadsky et al. 2013; Huang et al. 2014). Pituitary‐specific transcription factor 1 (*POU1F1*) (173110) plays a role in cell proliferation by increasing cyclin D1 expression and prolactin (*PRL*) (176760) levels (Ben‐Batalla et al. 2010). D‐type cyclins are cell‐cycle proteins that have been implicated in adipocyte differentiation both in vivo and in vitro (Lefterova and Lazar 2009). Conversely, active *PRL* produced in human adipose tissue plays a role in adipose tissue function, yet its expression in adipose tissue is thought to be regulated through a nonpituitary, alternative superdistal promoter (Brandebourg et al. 2007). Therefore, the observed expression of *PRL* from D7 to D21 in this study, coupled with the enrichment of *POU1F1* TFBS in the promoter region of all the up‐regulated genes during adipogenesis (D1 to 21), may offer the first indication that *PRL* is regulated by *POU1F1*. *TRIM28* (601742) is an epigenetic modifier, and mice with a heterozygous mutation in this gene have impaired glucose tolerance and adipocyte hypertrophy (Youngson and Morris 2013). These effects suggest a role for epigenetic modifications in obesity and other related metabolic diseases. *SP100* (604585) is a beige adipocyte‐specific gene (Rajakumari et al. 2013) whose regulatory role in gene expression during adipogenesis in ASCs remains unknown. *SRY* (480000) has a negative regulatory effect on adipogenesis by down‐regulating *CEBPB* and *CEBPG* expression. *SRY* is the target through which *DLK1* acts to inhibit adipogenesis (Sul 2009; Lowe et al. 2011). *ESR1*,* MOYG*,* SP100*,* NACC2 (BEN)* (615786), and *CRX* are a few of the TFs identified that have no defined role in adipogenesis.

### In silico identification of TFBS that are overrepresented in the promoter regions of up‐regulated genes

The F‐MATCH^®^ algorithm was used to identify TFBS that were significantly overrepresented in the promoter regions of the up‐regulated gene sets (referred to as the query sets) compared with the down‐regulated gene sets (referred to as the background gene sets) on each day of adipogenesis. We identified 34, 43, 46, and 41 PWMs that were significantly overrepresented in the up‐regulated gene set on D1, D7, D14, and D21, respectively (Table 2). This finding suggests that these TFs are likely to be involved in the overexpression of genes that are up‐regulated on the different days of adipogenesis.

**Table 2:** TFs whose binding sites are overrepresented on the different days of adipogenic differentiation in ASCs

Day 1	Day 7	Day 14	Day 21
AP‐2alphaA (TFA2A)	AIRE	AIRE	AIRE
Arid5a	AP‐2alphaA (TFA2A)	AP‐2alphaA (TFA2A)	AP‐2alphaA (TFA2A)
ATF‐2	ATF‐2	Arid5a	BBX secondary motif
NACC2	BBX secondary motif	BBX secondary motif	NACC2
CDX‐2	NACC2	NACC2	CDX‐2
Churchill (CHURC1)	CDX‐2	CDX‐2	Churchill (CHURC1)
CPBP (KLF16)	Churchill (CHURC1)	Churchill (CHURC1)	CPBP (KLF16)
CREB1	CPBP (KLF16)	CPBP (KLF16)	CRX
CRX	CRX	CREB1	CTCF
CTCF	CTCF	CRX	dlx‐3
dlx‐3	dlx‐3	CTCF	DRI1 (ARID3A)
DRI1 (ARID3A)	DRI1 (ARID3A)	dlx‐3	E2F (E2F1)
E2A (TCF3 or TCF7L1)	Ebox (ASCL1)	DRI1 (ARID3A)	Ets (ELF1)
E2F (E2F1)	ER‐alpha (ESR1)	E2F (E2F1)	FAC1 (BPTF)
FAC1 (BPTF)	Ets (ELF1)	ER‐alpha (ESR1)	FPM315 (ZNF263)
FPM315 (ZNF263)	FAC1 (BPTF)	Ets (ELF1)	GKLF (KLF4)
Freac‐3 (FOXC1)	FPM315 (ZNF263)	FAC1 (BPTF)	Hic1
GKLF (KLF4)	Freac‐3 (FOXC1)	FPM315 (ZNF263)	HMGIY (HMGA1)
HES‐1	GKLF (KLF4)	Freac‐3 (FOXC1)	HNF‐1alpha (HNF1A)
HMGIY (HMGA1)	HES‐1	GKLF (KLF4)	ING4
MAZ	HMGIY (HMGA1)	HMGIY (HMGA1)	LEF‐1
MECP2	HNF‐3beta (FOXA2)	ING4	LRH‐1 (NR5A2)
NF‐Y (NFYA)	LRH‐1 (NR5A2)	LEF‐1	MAF (BACH1)
Pit‐1 (POU1F1)	MAF (BACH1)	LRH‐1 (NR5A2)	MAZ
POU6F1	MAZ	MAZ	MAZR (PATZ1)
RNF96 (TRIM28)	MEIS1	MRF2 (ARID5B)	Muscle initiator
Sp1	Muscle initiator	Muscle initiator	myogenin (MOYG)
SP100 secondary motif	Myogenin (MYOG)	Myogenin (MYOG)	Pit‐1 (POU1F1)
SRY	Nkx2.5 (NKX2‐5)	NF‐Y (NFYA)	POU6F1
Tbx5	p53 (TFP53)	Nkx2.5	RelA‐p65 (RELA)
Xvent‐1 (ventx1.2)	Pit‐1 (POU1F1)	Pit‐1 (POU1F1)	RNF96 (TRIM28)
ZF5 secondary motif	POU6F1	POU2F1	Sp1
Zfx	RNF96 (TRIM28)	POU6F1	SP100 secondary motif
ZNF333	RREB‐1	RNF96 (TRIM28)	SRY
	RUSH‐1alpha (HLTF)	RREB‐1	STAT1
	SP100 secondary motif	Sp1	Tbx5
	SRY	SP100 secondary motif	Xvent‐1 (vent1.2)
	Tbx5	SREBP (SREPF1)	ZF5 secondary motif
	Xvent‐1 (vent1.2)	SRY	ZFP105 secondary motif
	ZF5 secondary motif	Tbx5	Zfx
	ZFP105 secondary motif	TEF‐1 (TEAD1)	ZNF333
	Zfx	Xvent‐1 (vent1.2)	
	ZNF333	ZF5 secondary motif	
		ZFP105 secondary motif	
		Zfx	
		ZNF333	

Interestingly, the binding sites for *TCF3* (E2A or TCF7L1) (147141) and *MECP2* (300005) (methyl‐CpG‐binding protein 2) were significantly overrepresented only in the promoter regions of the up‐regulated genes on D1. Tcf3 is induced in preadipocytes at confluence in a cell–cell‐contact‐dependent manner and regulates lineage differentiation in multipotent stem cells. Tcf3 also promotes adipogenesis in precursor cells by repressing cytostructural genes and activating adipocyte genes (Merrill et al. 2001; Cristancho et al. 2011). This evidence strongly supports our findings of overrepresentation of the binding sites for this TF in the promoter regions of genes that were up‐regulated on D1 and therefore implicates *TCF3* as an important regulatory molecule in the initial phase of adipogenesis in ASCs. *MECP2* is an epigenetic modifier whose downstream regulatory role in adipogenesis is not yet well understood. Functional studies are therefore needed to elucidate its role in preadipocyte differentiation and provide insight into the role of epigenetic modifiers in the initial stage of adipogenesis. The binding sites for *ASCL1* (100790), *FOXA2* (600288), *MEIS1* (601739), *TP53,* and *SMARCA3 (HLTF)* (603257*)* were significantly overrepresented only in the D7 up‐regulated gene sets. Meis1 is known to interact with other HOX proteins in a large transcriptional network, which has been reported to exhibit transcriptional up‐regulation on day 6 of adipocyte differentiation (Billon et al. 2010). The expression of Foxa2 in preadipocytes has been reported to inhibit adipogenesis (Wolfrum et al. 2003).

Transcription factor‐binding sites for *ARID5B* (608538), *POU2F1* (164172), and *TEAD1* (*TEF1*) (189967) were significantly overrepresented only in the promoter regions of the D14 up‐regulated gene sets, whereas TFBS for *HIC1 (603825)*,* HNF1A* (142410), *ZNF278* (*PATZ1*) (605165), *RELA* (164014), and *STAT1* (600555) were overrepresented only in the up‐regulated gene sets on D21. Mutations in *HNF1A* are associated with diabetes in both men and women (Hegele et al. 2000). *ZNF278* has been shown to interact with *TP53* and to regulate the expression of *TP53*‐dependent genes that are involved in cell migration and the epithelial‐mesenchymal transition (Chiappetta et al. 2015; Rönn et al. 2015). RelA is a key component of the NF‐κB family, and the inhibition of this TF impairs differentiation and reduces the accumulation of fat in adipocytes, which suggests a positive role in adipocyte differentiation (Ray et al. 2014). *STAT1* undergoes differentiation‐dependent expression in preadipocytes and is suggested to promote adipogenesis and inhibit lipolysis in mature adipocytes (J. M. Stephens et al. 1999; Zhao and Stephens 2013). The binding sites of TFs with functional roles not only in adipocyte differentiation but also in the function of mature adipocytes and adipose tissue were overrepresented in the promoter regions of the up‐regulated genes on D21, suggesting that other D21 upstream TFs play an important regulatory role in the expression of genes that define the function of adipose tissue and mature adipocytes. Furthermore, binding sites for 24 TFs (*TFAP2A*,* NACC2*,* CDX2*,* CHURC1* (608577), *KLF16*,* CRX*,* CTCF (*604167), *DLX3* (600525), *ARID3A*,* BPTF* (601819), *ZNF263* (604191), *KLF4*,* HMGA1*,* MAZ*,* POU1F1*,* POU6F1*,* TRIM28*,* SP100*,* SRY*,* TBX5*, Vent1.2, *ZFP161* (ZF5 or *ZBTB14*) (602126), *ZFX* (314980) and *ZNF333* (611811)) were significantly overrepresented in all the up‐regulated gene sets during adipogenesis (D1 to D21). Vent1.2 is a TF with a TFBS consensus sequence found in *Xenopus laevis*, as annotated in the TRANSFAC^®^ software database.

### Identification of 18 upstream TFs most likely to have a dominant regulatory effect on gene expression during ASC differentiation

**Figure 2:**
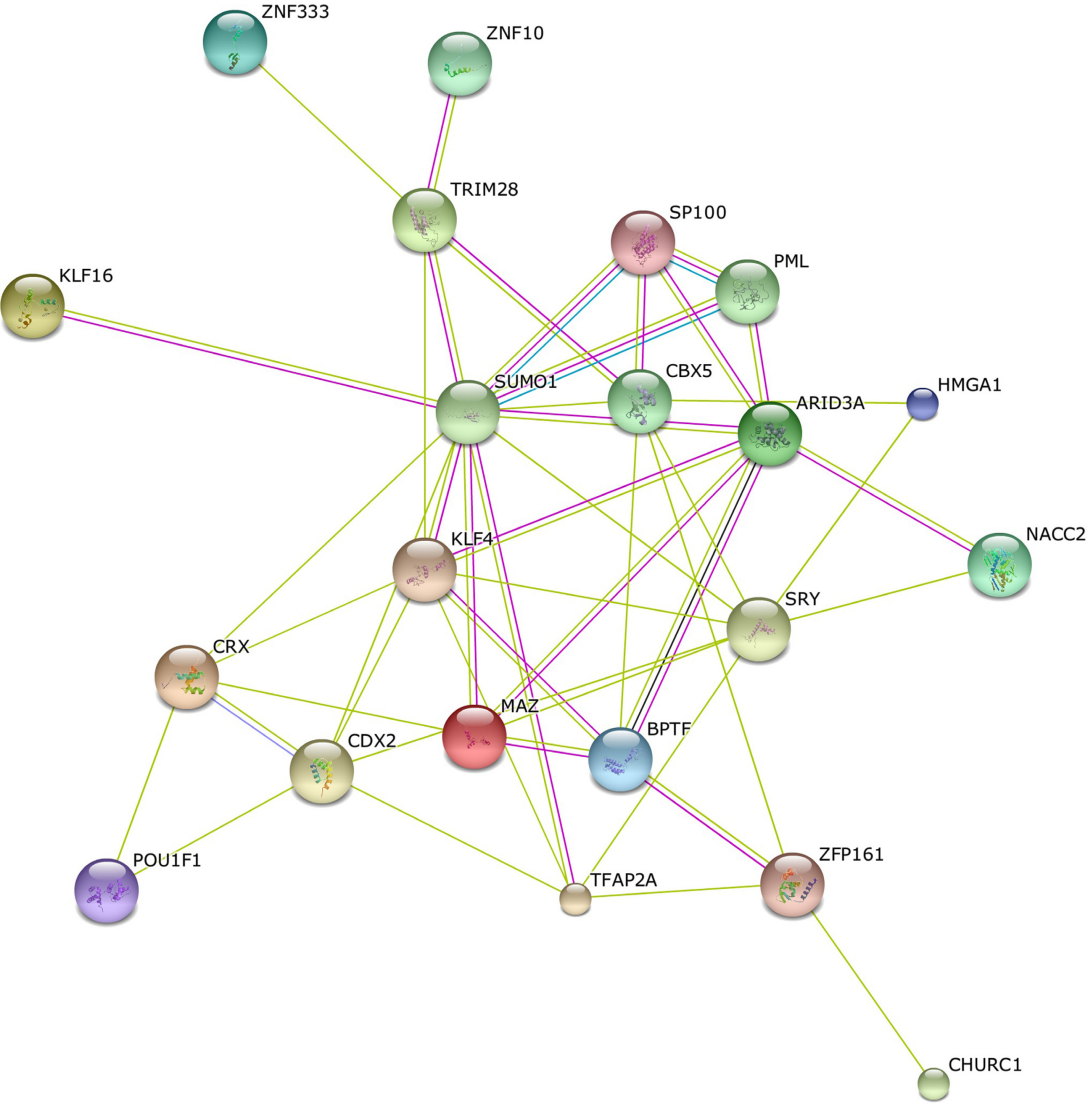
Functional association network between TFs. STRING v10 showed functional protein‐protein interactions of 18 upstream TFs with the exception of ventx1.2 that was predicted most likely to have a dominant regulatory effect on gene expression during ASC differentiation.

We reasoned that of the 32 TFs potentially involved in the regulation of the DEGs observed during ASC differentiation, those TFs whose binding sites are overrepresented in the promoter regions of up‐regulated genes by more than twofold on each day throughout the 21‐day differentiation period would represent the subset of TFs most likely to have a dominant regulatory effect on gene expression during adipogenesis. We identified 18 upstream TFs, namely, *TFAP2A*,* NACC2*,* CDX2*,* CHURC1*,* KLF16*,* CRX*,* ARID3A*,* BPTF*,* KLF4*,* HMGA1*,* MAZ*,* POU1F1*,* TRIM28*,* SP100*,* SRY*, Ventx1.2, *ZFP161* and *ZNF333*, that constitute this subset. STRING v10 was used to assess the functional associations between these 18 TFs. Except for ventx1.2, all the TFs formed a functional association network (Fig. 2) together with other identified functional interacting partners (*SUM01*,* PML* (102578), *CBX5* (604478) and *ZNF10* (194538)). Hence, this analysis suggests the involvement of these TFs in a common biological process, such as the regulation of gene expression during adipocyte differentiation *in vitro*.

Notably, 10 of these 18 TFs belong to the HOX (*CDX2*,* CRX*,* POU1F1* and Ventx1.2) and zinc finger (*KLF16*,* KLF4*,* MAZ*,* TRIM28*,* ZFP161* and *ZNF33*) protein families. A few members of these TF families (*CDX2*,* KLF16* and *KLF4*) have already been shown to have a functional role in adipogenesis, whereas others such as *ZFP161*,* ZNF333*,* MAZ*, and *CRX* have no reported role in this process. Owing to the importance of these two protein families in the transcriptional regulation of many biological processes including ASC differentiation (Liu et al. 2015; Seifert 2015), further functional studies on TFs with no reported role in adipocyte differentiation may assist in understanding this process in humans. Importantly, none of the TFs identified in this study were differentially expressed during adipogenesis according to the microarray data (Ambele et al. 2016) used in this study. This observation supports our methodology and our aim to identify upstream TFs that work together either directly or indirectly (as observed in Fig. 2) to regulate gene expression during adipocyte differentiation.

### TFs with an increased regulatory effect on gene expression over time in ASC adipogenesis

In this study, we observed that the number of up‐regulated genes increased during adipogenesis (Fig. 1). We therefore investigated, using *in silico* prediction, whether this increase was associated with a corresponding increase in the regulatory activity of some of the TFs. By considering the up‐regulated genes on each day or time point to be a query set and those of the previous day or time point to be a background set, we can use the F‐MATCH™ algorithm to identify TFs whose binding sites are overrepresented in the promoter regions of up‐regulated genes that were expressed in a differentiation‐dependent manner from D7 to D21. Day 7 represents the starting time point at which most of the characteristic markers for adipocyte differentiation, such as *FABP4* (600434) and *ADIPOQ* (605441), are significantly expressed (Ambele et al. 2016). TFs whose binding sites were increasingly overrepresented from D7 to D21 would suggest an increase in their regulatory effect on gene expression over time as adipogenesis progresses. F‐MATCH™ revealed a significant overrepresentation (by more than twofold) of 38 PWMs for TFBS in the promoter regions of the up‐regulated genes on D7 compared with those on D1 (D7 to D1). The number of PWMs with an overrepresentation of more than twofold from D14 to D7 and D21 to D14 were 32 and 36, respectively. We then identified 28 PWMs (Table 3) for 28 known TFs that were common in the F‐MATCH™ analysis results obtained for the D7 (D7 to D1), D14 (D14 to D7), and D21 (D21 to D14) up‐regulated gene query sets. This finding implies that the number of genes with binding sites for these 28 TFs increased in a differentiation‐dependent manner from D7 to D21 and further suggests an increase in the regulatory effects of these 28 upstream TFs on their downstream target genes, which were consistently up‐regulated in a differentiation‐dependent manner during adipogenesis in ASCs. Interestingly, with the exception of *BPTF*, 17 of the 18 upstream TFs that were identified to have a dominant regulatory effect on downstream gene expression, during ASC adipogenesis were also predicted among the 28 TFs that were identified to have an increased regulatory effect on gene expression during adipogenesis. This correlation further strengthens the identification of TFs that are potentially important in driving pre‐/adipocytes through their differentiation, including the expression of downstream target genes that were observed to be differentially expressed during adipogenesis in ASCs (Ambele et al. 2016).

**Table 3:** F‐MATCH analysis revealed 28 PWMs for 28 TFBS that were significantly overrepresented by more than twofold in the promoter regions of genes that were up‐regulated over time (D7 to 21), suggesting a role for these TFs in regulating gene expression during adipogenesis in ASCs

Matrix	Consensus sequence	Factor name
*V$AP2ALPHA_03*	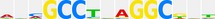	AP‐2alphaA (TFAP2A)
*V$BEN_01*	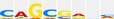	NACC2
*V$CDX2_Q5_02*	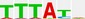	CDX‐2
*V$CHCH_01*	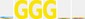	Churchill (CHURC1)
*V$CPBP_Q6*	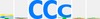	CPBP (KLF16)
*V$CREB1_Q6*	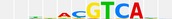	CREB1
*V$CRX_Q4_01*	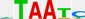	CRX
*V$DRI1_01*	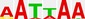	DRI1 (ARID3A)
*V$E2F_Q6_01*	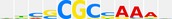	E2F (E2F1)
*V$EGR1_Q6*	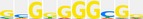	Egr‐1
*V$FPM315_01*	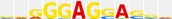	FPM315 (ZNF263)
*V$GATA_Q6*	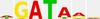	GATA
*V$GKLF_Q4*	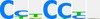	GKLF (KLF4)
*V$HES1_Q6*	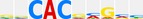	HES‐1
*V$HMGIY_Q3*	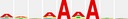	HMGIY (HMGA1)
*V$IK_Q5_01*	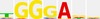	Ikaros (IKZF1)
*V$ING4_01*	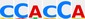	ING4
*V$MAZ_Q6_01*	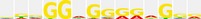	MAZ
*V$PIT1_Q6_01*	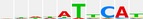	Pit‐1 (POU1F1)
*V$RNF96_01*	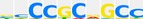	RNF96 (TRIM28)
*V$SP100_04*	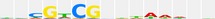	SP100 secondary motif (Sp100)
*V$SRY_Q6*	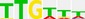	SRY
*V$XVENT1_01*	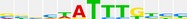	Xvent‐1 (ventx1.2)
*V$YY1_Q6_03*	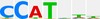	YY1
*V$ZFP161_04*	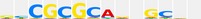	ZF5 secondary motif (ZFP161)
*V$ZFP105_04*	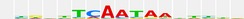	ZFP105 secondary motif (Zfp105)
*V$ZFX_01*	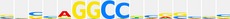	Zfx
*V$ZNF333_01*	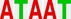	ZNF333

## Conclusions

The majority of the TFs previously described to play a role in adipocyte differentiation have been identified from studies, using either 3T3‐L1 cells or preadipocytes from animal models. In this study, we used ASCs derived from humans to study adipogenesis in vitro. This approach successfully identified TFs with previously described roles in adipogenesis as well as TFs with no previously described role in this process. These results strongly validate our comprehensive and systematic in silico approach which aims to unravel new regulators that are important for human ASC adipogenic differentiation. Exploring these novel TFs in functional studies may provide a greater understanding of this process in humans and could potentially lead to the discovery and development of anti‐obesity drugs.

## Conflict of Interest

The authors declare that they have no competing interests.

## Supporting information


**Table S1.** A complete list of differentially expressed genes on day 1.

**Table S2.** A complete list of differentially expressed genes on day 7.

**Table S3.** A complete list of differentially expressed genes on day 14.

**Table S4.** A complete list of differentially expressed genes on day 21.
