# Supplementary material for: Identification of transcription factors potentially involved in human adipogenesis in vitro
Source: Mol Genet Genomic Med. 2017 Mar 3;5(3):210–22. doi: 10.1002/mgg3.269 (PMC5441431; doi:10.1002/mgg3.269)
Supplement: Supplementary file 1 — Table S1. A complete list of differentially expressed genes on day 1. [file MGG3-5-210-s001.doc]

|  |  |  |  |  |  |
| --- | --- | --- | --- | --- | --- |
| **Up-regulated genes on day 1** | |  |  |  |  |
| Transcript Cluster ID | Fold Change (linear) (Induced vs. Control) | ANOVA p-value (Induced vs. Control) | FDR p-value (Induced vs. Control) | Gene Symbol | Description |
| 16774303 | 120.79 | 0.000043 | 0.021075 | RGCC | regulator of cell cycle |
| 17059955 | 117.74 | 0.008184 | 0.196563 | PDK4 | pyruvate dehydrogenase kinase, isozyme 4 |
| 16779958 | 42.56 | 0.002059 | 0.108275 | EDNRB | endothelin receptor type B |
| 16767335 | 39.19 | 0.000108 | 0.031133 | CPM | carboxypeptidase M; NULL |
| 16676988 | 33.09 | 0.000026 | 0.018656 | HSD11B1 | hydroxysteroid (11-beta) dehydrogenase 1 |
| 16826738 | 23.03 | 0.000077 | 0.026736 | MT1G | metallothionein 1G |
| 16821541 | 22.36 | 0.000204 | 0.040274 | CRISPLD2 | cysteine-rich secretory protein LCCL domain containing 2 |
| 16830883 | 21.68 | 0.000002 | 0.00823 | ALOX15B | arachidonate 15-lipoxygenase, type B |
| 16675323 | 20.16 | 0.000006 | 0.011074 | RGS2 | regulator of G-protein signaling 2, 24kDa; NULL |
| 16819224 | 18.13 | 0.000007 | 0.011546 | MT1M | metallothionein 1M |
| 17061759 | 15.79 | 0.000003 | 0.008747 | NRCAM | neuronal cell adhesion molecule; NULL |
| 16938133 | 14.38 | 0.001332 | 0.088615 | GALNT15 | UDP-N-acetyl-alpha-D-galactosamine:polypeptide N-acetylgalactosaminyltransferase 15 |
| 16751190 | 14.17 | 0.000335 | 0.048518 | METTL7A | methyltransferase like 7A; NULL |
| 16841907 | 11.05 | 0.000784 | 0.069374 | RASD1 | RAS, dexamethasone-induced 1 |
| 17102829 | 11.02 | 0.003463 | 0.137229 | MAOA | monoamine oxidase A |
| 16790744 | 11.02 | 0.000043 | 0.021075 | SLC7A8 | solute carrier family 7 (amino acid transporter light chain, L system), member 8; NULL |
| 16952782 | 10.99 | 0.001909 | 0.105973 | TMEM158 | transmembrane protein 158 (gene/pseudogene) |
| 16704320 | 9.73 | 0.013127 | 0.237516 | RASSF4 | Ras association (RalGDS/AF-6) domain family member 4; NULL |
| 17021510 | 8.93 | 0.011758 | 0.227199 | CNR1 | cannabinoid receptor 1 (brain); NULL |
| 16851026 | 8.79 | 0.000438 | 0.052725 | IMPA2 | inositol(myo)-1(or 4)-monophosphatase 2 |
| 16819264 | 8.71 | 0.000079 | 0.027098 | MT1X | metallothionein 1X |
| 16666485 | 8.69 | 0.000016 | 0.014596 | IFI44L | interferon-induced protein 44-like; NULL |
| 16889268 | 7.48 | 0.000093 | 0.028629 | AOX1 | aldehyde oxidase 1; NULL |
| 17113346 | 7.04 | 0.003695 | 0.141227 | CHRDL1 | chordin-like 1; NULL |
| 17001846 | 7.03 | 0.006861 | 0.182149 | CCDC69 | coiled-coil domain containing 69; NULL |
| 16851309 | 6.89 | 0.002413 | 0.118843 | GREB1L | growth regulation by estrogen in breast cancer-like; NULL |
| 17075589 | 6.87 | 0.00952 | 0.209266 | NEFL | neurofilament, light polypeptide |
| 17067011 | 6.8 | 0.035527 | 0.352753 | NEFM | neurofilament, medium polypeptide |
| 16761938 | 6.16 | 0.000567 | 0.058703 | LMO3 | LIM domain only 3 (rhombotin-like 2); NULL |
| 16819252 | 6.06 | 0.037603 | 0.360309 | MT1F | metallothionein 1F; NULL |
| 16840846 | 5.92 | 0.000064 | 0.024908 | PER1 | period circadian clock 1 |
| 16919022 | 5.8 | 0.000776 | 0.068866 | SAMHD1 | SAM domain and HD domain 1 |
| 17012859 | 5.71 | 0.000074 | 0.026566 | PDE7B | phosphodiesterase 7B |
| 16950609 | 5.63 | 0.01131 | 0.224821 | CIDEC | cell death-inducing DFFA-like effector c |
| 16778392 | 5.56 | 0.000431 | 0.052725 | FOXO1 | forkhead box O1 |
| 16780917 | 5.35 | 0.000019 | 0.016123 | IRS2 | insulin receptor substrate 2 |
| 17102129 | 5.25 | 0.000011 | 0.013102 | SAT1 | spermidine/spermine N1-acetyltransferase 1; NULL |
| 16819233 | 5.12 | 0.015875 | 0.255658 | MT1A | metallothionein 1A |
| 16876777 | 5.06 | 0.000884 | 0.074754 | RNF144A | ring finger protein 144A; NULL |
| 17022623 | 5.05 | 0.00008 | 0.02719 | REV3L | REV3-like, polymerase (DNA directed), zeta, catalytic subunit; NULL |
| 16883647 | 5.04 | 0.000029 | 0.019043 | IL1R1 | interleukin 1 receptor, type I; NULL |
| 16696177 | 4.97 | 0.00066 | 0.063288 | SLC19A2 | solute carrier family 19 (thiamine transporter), member 2 |
| 16713562 | 4.93 | 0.013379 | 0.239323 | C10orf10 | chromosome 10 open reading frame 10 |
| 16700888 | 4.87 | 0.003368 | 0.135243 | NID1 | nidogen 1 |
| 16958638 | 4.76 | 0.004771 | 0.157798 | KLF15 | Kruppel-like factor 15 |
| 17018497 | 4.7 | 0.000184 | 0.039103 | FKBP5; LOC285847 | FK506 binding protein 5; uncharacterized LOC285847 |
| 16819213 | 4.67 | 0.003514 | 0.138002 | MT1L; NUTF2 | metallothionein 1L (gene/pseudogene); nuclear transport factor 2 |
| 16696979 | 4.66 | 0.000013 | 0.014085 | GLUL | glutamate-ammonia ligase; NULL |
| 17095056 | 4.59 | 0.001501 | 0.093669 | PRUNE2 | prune homolog 2 (Drosophila); NULL |
| 16850107 | 4.56 | 0.000154 | 0.036445 | FASN | fatty acid synthase |
| 16696811 | 4.38 | 0.012054 | 0.229591 | ANGPTL1 | angiopoietin-like 1 |
| 16955197 | 4.38 | 0.002703 | 0.124034 | WNT5A | wingless-type MMTV integration site family, member 5A |
| 16698356 | 4.37 | 0.006708 | 0.18066 | PIK3C2B | phosphatidylinositol-4-phosphate 3-kinase, catalytic subunit type 2 beta; NULL |
| 17113147 | 4.32 | 0.000151 | 0.036125 | TSC22D3 | TSC22 domain family, member 3; NULL |
| 16852702 | 4.21 | 0.000393 | 0.050838 | CDH20 | cadherin 20, type 2 |
| 17058826 | 4.21 | 0.000053 | 0.022892 | HIP1 | huntingtin interacting protein 1; NULL |
| 16731441 | 4.18 | 0.000087 | 0.027826 | ZBTB16 | zinc finger and BTB domain containing 16; NULL |
| 16947173 | 4.16 | 0.00024 | 0.041932 | MME | membrane metallo-endopeptidase; NULL |
| 17095499 | 4.11 | 0.000407 | 0.051911 | GAS1 | growth arrest-specific 1 |
| 16819217 | 4.05 | 0.00125 | 0.086384 | MT1E | metallothionein 1E |
| 16762759 | 4.01 | 0.006396 | 0.177376 | TMTC1 | transmembrane and tetratricopeptide repeat containing 1 |
| 16985518 | 3.98 | 0.000037 | 0.020894 | PIK3R1 | phosphoinositide-3-kinase, regulatory subunit 1 (alpha); NULL |
| 16763577 | 3.94 | 0.013339 | 0.239232 | SLC38A4 | solute carrier family 38, member 4 |
| 16708249 | 3.93 | 0.00054 | 0.057488 | SCD | stearoyl-CoA desaturase (delta-9-desaturase) |
| 17102951 | 3.9 | 0.003408 | 0.136046 | CHST7 | carbohydrate (N-acetylglucosamine 6-O) sulfotransferase 7 |
| 17061662 | 3.86 | 0.000002 | 0.008304 | LAMB1 | laminin, beta 1 |
| 16977378 | 3.83 | 0.009407 | 0.208383 | TMEM150C | transmembrane protein 150C |
| 16819229 | 3.82 | 0.001307 | 0.087393 | MT1JP | metallothionein 1J, pseudogene |
| 16763182 | 3.78 | 0.028376 | 0.324122 | ABCD2 | ATP-binding cassette, sub-family D (ALD), member 2 |
| 16784207 | 3.76 | 0.000205 | 0.040274 | PTGER2 | prostaglandin E receptor 2 (subtype EP2), 53kDa |
| 17087790 | 3.76 | 0.00001 | 0.012224 | SLC44A1 | solute carrier family 44, member 1 |
| 16874935 | 3.75 | 0.009356 | 0.207897 | HAS1 | hyaluronan synthase 1 |
| 16836311 | 3.72 | 0.00888 | 0.203052 | NOG | noggin |
| 16689546 | 3.71 | 0.000422 | 0.052365 | TGFBR3 | transforming growth factor, beta receptor III; NULL |
| 16739132 | 3.66 | 0.00001 | 0.012224 | FADS1; MIR1908 | fatty acid desaturase 1; microRNA 1908; NULL |
| 17002820 | 3.66 | 0.000032 | 0.020309 | SH3PXD2B | SH3 and PX domains 2B |
| 16982047 | 3.64 | 0.000038 | 0.020894 | ACSL1 | acyl-CoA synthetase long-chain family member 1; NULL |
| 17023150 | 3.56 | 0.000508 | 0.05609 | MAN1A1 | mannosidase, alpha, class 1A, member 1 |
| 17110071 | 3.55 | 0.000002 | 0.008241 | SRPX | sushi-repeat containing protein, X-linked |
| 17080082 | 3.53 | 0.003279 | 0.133706 | ANGPT1 | angiopoietin 1 |
| 17063461 | 3.52 | 0.001032 | 0.080351 | HIPK2 | homeodomain interacting protein kinase 2 |
| 16886564 | 3.46 | 0.00127 | 0.086384 | FMNL2 | formin-like 2 |
| 16919962 | 3.43 | 0.004638 | 0.155542 | SULF2 | sulfatase 2; NULL |
| 16701975 | 3.42 | 0.000005 | 0.010118 | AKR1C1 | aldo-keto reductase family 1, member C1; NULL |
| 17076609 | 3.42 | 0.002977 | 0.128923 | SFRP1 | secreted frizzled-related protein 1 |
| 16846864 | 3.4 | 0.000194 | 0.039411 | MMD | monocyte to macrophage differentiation-associated |
| 16947148 | 3.39 | 0.000007 | 0.011546 | ARHGEF26 | Rho guanine nucleotide exchange factor (GEF) 26 |
| 16883715 | 3.28 | 0.003301 | 0.134145 | IL18R1 | interleukin 18 receptor 1 |
| 17095882 | 3.27 | 0.030407 | 0.332568 | OMD | osteomodulin |
| 17002052 | 3.25 | 0.00918 | 0.205819 | FAXDC2; C5orf4 | fatty acid hydroxylase domain containing 2; NULL |
| 17106183 | 3.25 | 0.000005 | 0.010118 | TMEM164 | transmembrane protein 164 |
| 16891603 | 3.2 | 0.000073 | 0.026566 | KCNE4 | potassium voltage-gated channel, Isk-related family, member 4 |
| 16909303 | 3.19 | 0.000392 | 0.050838 | PID1 | phosphotyrosine interaction domain containing 1 |
| 16953597 | 3.18 | 0.000013 | 0.014085 | SLC26A6 | solute carrier family 26, member 6; NULL |
| 16681304 | 3.16 | 0.000061 | 0.024317 | ERRFI1 | ERBB receptor feedback inhibitor 1 |
| 17022362 | 3.14 | 0.000362 | 0.050343 | SESN1 | sestrin 1 |
| 16851022 | 3.13 | 0.001527 | 0.094398 | CHMP1B | charged multivesicular body protein 1B |
| 16800980 | 3.12 | 0.002009 | 0.10805 | GABPB1-AS1; GABPB1 | GABPB1 antisense RNA 1; GA binding protein transcription factor, beta subunit 1; NULL |
| 16739208 | 3.07 | 0.000326 | 0.047816 | FTH1 | ferritin, heavy polypeptide 1; NULL |
| 16722720 | 3.06 | 0.000595 | 0.0602 | NAV2 | neuron navigator 2; NULL |
| 16748989 | 3.06 | 0.032997 | 0.342719 | PDE3A | phosphodiesterase 3A, cGMP-inhibited |
| 17063005 | 3.06 | 0.0247 | 0.306765 | PLXNA4 | plexin A4 |
| 17024144 | 3.01 | 5.14E-07 | 0.005824 | IFNGR1 | interferon gamma receptor 1; NULL |
| 16711343 | 2.98 | 0.000555 | 0.058225 | AKR1C2; LOC101060798 | aldo-keto reductase family 1, member C2; aldo-keto reductase family 1 member C2-like; NULL |
| 16768413 | 2.93 | 0.003269 | 0.133706 | DCN | decorin; NULL |
| 16955822 | 2.9 | 0.004709 | 0.156511 | ADAMTS9 | ADAM metallopeptidase with thrombospondin type 1 motif, 9; NULL |
| 16858386 | 2.9 | 0.00332 | 0.134631 | LDLR | low density lipoprotein receptor; NULL |
| 16837191 | 2.89 | 0.000575 | 0.059378 | PITPNC1 | phosphatidylinositol transfer protein, cytoplasmic 1 |
| 16928204 | 2.88 | 0.046584 | 0.389702 | POM121L9P; LOC727983 | POM121 transmembrane nucleoporin-like 9, pseudogene; putative POM121-like protein 1-like |
| 16980974 | 2.86 | 0.00104 | 0.080351 | FAM198B | family with sequence similarity 198, member B |
| 16744049 | 2.86 | 0.004657 | 0.155542 | SLC35F2 | solute carrier family 35, member F2; NULL |
| 16769761 | 2.85 | 0.000821 | 0.071362 | TMEM119 | transmembrane protein 119 |
| 16809687 | 2.84 | 0.003388 | 0.135588 | NEDD4 | neural precursor cell expressed, developmentally down-regulated 4, E3 ubiquitin protein ligase; NULL |
| 16773453 | 2.82 | 0.000012 | 0.013496 | WASF3 | WAS protein family, member 3 |
| 17076063 | 2.81 | 0.005753 | 0.170266 | GSR | glutathione reductase |
| 17005276 | 2.81 | 0.00197 | 0.107324 | SOX4 | SRY (sex determining region Y)-box 4 |
| 16906733 | 2.81 | 0.001301 | 0.087142 | STK17B | serine/threonine kinase 17b |
| 17075973 | 2.8 | 0.003264 | 0.133706 | DUSP4 | dual specificity phosphatase 4 |
| 16743111 | 2.78 | 0.000142 | 0.035273 | CTSC | cathepsin C; NULL |
| 17095703 | 2.77 | 0.000244 | 0.042163 | NFIL3 | nuclear factor, interleukin 3 regulated |
| 16964888 | 2.76 | 0.049168 | 0.398523 | HTRA3 | HtrA serine peptidase 3 |
| 16708192 | 2.75 | 0.005161 | 0.161846 | ABCC2 | ATP-binding cassette, sub-family C (CFTR/MRP), member 2 |
| 16834091 | 2.75 | 0.000162 | 0.037892 | IGFBP4 | insulin-like growth factor binding protein 4 |
| 16686557 | 2.73 | 0.000488 | 0.054855 | PIK3R3; OTTHUMG00000007603; RP11-322N21.2 | phosphoinositide-3-kinase, regulatory subunit 3 (gamma); NULL |
| 16942270 | 2.7 | 0.000017 | 0.015346 | PTPRG | protein tyrosine phosphatase, receptor type, G; NULL |
| 17067231 | 2.69 | 0.001341 | 0.088837 | PTK2B | protein tyrosine kinase 2 beta; NULL |
| 16730967 | 2.67 | 0.002804 | 0.126143 | C11orf87 | chromosome 11 open reading frame 87 |
| 16912362 | 2.67 | 0.013464 | 0.239855 | ID1 | inhibitor of DNA binding 1, dominant negative helix-loop-helix protein |
| 17102512 | 2.66 | 0.000166 | 0.038063 | PRRG1 | proline rich Gla (G-carboxyglutamic acid) 1; NULL |
| 17048072 | 2.65 | 0.000285 | 0.045354 | STEAP1 | six transmembrane epithelial antigen of the prostate 1 |
| 16791991 | 2.64 | 0.000469 | 0.053473 | NFKBIA | nuclear factor of kappa light polypeptide gene enhancer in B-cells inhibitor, alpha |
| 16702399 | 2.63 | 0.020183 | 0.283543 | CELF2 | CUGBP, Elav-like family member 2; NULL |
| 17002846 | 2.61 | 0.000164 | 0.038063 | DUSP1 | dual specificity phosphatase 1 |
| 16800921 | 2.6 | 0.047373 | 0.392799 | FGF7 | fibroblast growth factor 7 |
| 17103303 | 2.6 | 0.039665 | 0.367126 | PORCN | porcupine homolog (Drosophila); NULL |
| 16879385 | 2.57 | 0.004255 | 0.149493 | PKDCC | protein kinase domain containing, cytoplasmic; NULL |
| 16850069 | 2.56 | 0.000455 | 0.052973 | DCXR | dicarbonyl/L-xylulose reductase; NULL |
| 16890207 | 2.56 | 0.005128 | 0.161846 | MAP2 | microtubule-associated protein 2; NULL |
| 16823866 | 2.56 | 0.011089 | 0.222358 | SOCS1 | suppressor of cytokine signaling 1 |
| 17093031 | 2.55 | 0.013866 | 0.242279 | MOB3B | MOB kinase activator 3B |
| 17081447 | 2.55 | 0.000121 | 0.032886 | ST3GAL1 | ST3 beta-galactoside alpha-2,3-sialyltransferase 1; NULL |
| 16688332 | 2.54 | 0.041029 | 0.371583 | DIRAS3 | DIRAS family, GTP-binding RAS-like 3 |
| 16687208 | 2.54 | 0.027562 | 0.320512 | ZCCHC11 | zinc finger, CCHC domain containing 11; NULL |
| 16857886 | 2.53 | 0.027067 | 0.318483 | ANGPTL4 | angiopoietin-like 4; NULL |
| 16678114 | 2.53 | 0.020318 | 0.284431 | EPHX1 | epoxide hydrolase 1, microsomal (xenobiotic) |
| 17010929 | 2.51 | 0.000232 | 0.041768 | PNRC1 | proline-rich nuclear receptor coactivator 1 |
| 17097132 | 2.5 | 0.00175 | 0.10252 | LPAR1 | lysophosphatidic acid receptor 1 |
| 16771602 | 2.48 | 0.038304 | 0.362995 | HPD | 4-hydroxyphenylpyruvate dioxygenase |
| 16962584 | 2.47 | 0.000245 | 0.042163 | BCL6 | B-cell CLL/lymphoma 6; NULL |
| 16731084 | 2.46 | 0.002447 | 0.119562 | SIK2 | salt-inducible kinase 2 |
| 16748788 | 2.45 | 0.000009 | 0.011546 | MGST1 | microsomal glutathione S-transferase 1; NULL |
| 16848123 | 2.44 | 0.009615 | 0.209906 | ABCA8 | ATP-binding cassette, sub-family A (ABC1), member 8; NULL |
| 16938630 | 2.44 | 0.033143 | 0.343313 | GPD1L | glycerol-3-phosphate dehydrogenase 1-like; NULL |
| 16798238 | 2.44 | 0.003079 | 0.13054 | SNRPN; LOC100506948; SNORD116-28; SNORD115-26; SNORD115-13; SNORD115-7; SNORD107; SNHG14 | small nuclear ribonucleoprotein polypeptide N; uncharacterized LOC100506948; small nucleolar RNA, C/D box 116-28; small nucleolar RNA, C/D box 115-26; small nucleolar RNA, C/D box 115-13; small nucleolar RNA, C/D box 115-7; small nucleolar RNA, C/D box 107; NULL |
| 16947287 | 2.44 | 0.000226 | 0.041323 | TIPARP | TCDD-inducible poly(ADP-ribose) polymerase; NULL |
| 16956316 | 2.43 | 0.001894 | 0.105826 | CNTN3 | contactin 3 (plasmacytoma associated) |
| 16725742 | 2.43 | 0.000177 | 0.038908 | FADS2 | fatty acid desaturase 2 |
| 16785379 | 2.43 | 0.025252 | 0.309564 | HSPA2 | heat shock 70kDa protein 2 |
| 16935620 | 2.43 | 0.025181 | 0.309315 | RRP7B | ribosomal RNA processing 7 homolog B (S. cerevisiae) |
| 16773165 | 2.43 | 0.009765 | 0.210689 | TNFRSF19 | tumor necrosis factor receptor superfamily, member 19 |
| 17076861 | 2.42 | 0.000383 | 0.050703 | CEBPD | CCAAT/enhancer binding protein (C/EBP), delta |
| 16933140 | 2.42 | 0.042772 | 0.377375 | GGT5 | gamma-glutamyltransferase 5 |
| 16836528 | 2.42 | 0.008514 | 0.200391 | YPEL2 | yippee-like 2 (Drosophila); NULL |
| 16847933 | 2.41 | 0.037177 | 0.358512 | AXIN2; OTTHUMG00000179524; CTD-2535L24.2 | axin 2; NULL |
| 16708179 | 2.4 | 0.000699 | 0.065214 | CUTC | cutC copper transporter homolog (E. coli); NULL |
| 16676983 | 2.36 | 0.003356 | 0.135112 | G0S2 | G0/G1switch 2 |
| 16847795 | 2.35 | 0.000057 | 0.023942 | TEX2 | testis expressed 2; NULL |
| 16922147 | 2.34 | 0.01376 | 0.241819 | EVA1C | eva-1 homolog C (C. elegans); NULL |
| 16861997 | 2.34 | 0.001091 | 0.081445 | ZFP36 | ZFP36 ring finger protein |
| 16674973 | 2.33 | 0.008195 | 0.196563 | C1orf21 | chromosome 1 open reading frame 21 |
| 16699533 | 2.33 | 0.00184 | 0.104656 | DUSP10 | dual specificity phosphatase 10; NULL |
| 16936947 | 2.32 | 0.007295 | 0.185943 | ITPR1 | inositol 1,4,5-trisphosphate receptor, type 1; NULL |
| 16956285 | 2.32 | 0.010958 | 0.221978 | PDZRN3 | PDZ domain containing ring finger 3; NULL |
| 16658864 | 2.32 | 0.005282 | 0.162984 | PGD | phosphogluconate dehydrogenase; NULL |
| 16705011 | 2.3 | 0.000278 | 0.044715 | DKK1 | dickkopf WNT signaling pathway inhibitor 1; NULL |
| 16955800 | 2.3 | 0.000143 | 0.035433 | PRICKLE2 | prickle homolog 2 (Drosophila) |
| 16828210 | 2.3 | 0.002974 | 0.128923 | ZFHX3 | zinc finger homeobox 3 |
| 16726880 | 2.29 | 0.033662 | 0.345545 | NEAT1 | nuclear paraspeckle assembly transcript 1 (non-protein coding) |
| 17056861 | 2.28 | 0.047567 | 0.39354 | AMPH | amphiphysin; NULL |
| 16686839 | 2.28 | 0.021378 | 0.290256 | TRABD2B | TraB domain containing 2B |
| 16893143 | 2.28 | 0.013861 | 0.242279 | TWIST2 | twist basic helix-loop-helix transcription factor 2 |
| 16743091 | 2.27 | 0.006157 | 0.17483 | FZD4 | frizzled family receptor 4 |
| 16822014 | 2.26 | 0.000393 | 0.050838 | CPNE7 | copine VII |
| 16911493 | 2.26 | 0.042539 | 0.376378 | SPTLC3 | serine palmitoyltransferase, long chain base subunit 3; NULL |
| 17112975 | 2.25 | 0.000431 | 0.052725 | RAB40A | RAB40A, member RAS oncogene family |
| 16804333 | 2.23 | 0.014816 | 0.248659 | AKAP13 | A kinase (PRKA) anchor protein 13; NULL |
| 16800061 | 2.23 | 0.003614 | 0.140358 | CAPN3; OTTHUMG00000171261; RP11-164J13.1 | calpain 3, (p94); NULL |
| 17053892 | 2.22 | 0.024802 | 0.307202 | INSIG1 | insulin induced gene 1 |
| 16991180 | 2.22 | 0.001364 | 0.089852 | SMIM3 | small integral membrane protein 3 |
| 16794158 | 2.22 | 0.000127 | 0.033472 | ZFP36L1 | ZFP36 ring finger protein-like 1 |
| 16929562 | 2.21 | 0.001379 | 0.09005 | HMOX1 | heme oxygenase (decycling) 1 |
| 17048563 | 2.21 | 0.028684 | 0.325104 | PEG10 | paternally expressed 10 |
| 17004657 | 2.2 | 0.009769 | 0.210689 | BMP6 | bone morphogenetic protein 6 |
| 16889653 | 2.2 | 0.007979 | 0.194631 | FAM117B | family with sequence similarity 117, member B |
| 16902646 | 2.2 | 0.003936 | 0.144359 | HS6ST1 | heparan sulfate 6-O-sulfotransferase 1 |
| 16990862 | 2.19 | 0.026894 | 0.317935 | ABLIM3 | actin binding LIM protein family, member 3; NULL |
| 16998059 | 2.19 | 0.001988 | 0.107578 | ARRDC3 | arrestin domain containing 3; NULL |
| 16819653 | 2.19 | 0.000355 | 0.049716 | MMP15 | matrix metallopeptidase 15 (membrane-inserted) |
| 16665346 | 2.18 | 0.00148 | 0.092829 | NFIA | nuclear factor I/A; NULL |
| 17022996 | 2.18 | 0.00127 | 0.086384 | ROS1; GOPC | c-ros oncogene 1 , receptor tyrosine kinase; golgi-associated PDZ and coiled-coil motif containing |
| 16683377 | 2.17 | 0.030416 | 0.332568 | ID3 | inhibitor of DNA binding 3, dominant negative helix-loop-helix protein |
| 17012447 | 2.15 | 0.004031 | 0.145494 | LAMA2 | laminin, alpha 2 |
| 17086762 | 2.15 | 0.005499 | 0.166188 | LINC00475 | long intergenic non-protein coding RNA 475; NULL |
| 17092767 | 2.15 | 0.002449 | 0.119562 | MLLT3 | myeloid/lymphoid or mixed-lineage leukemia (trithorax homolog, Drosophila); translocated to, 3; NULL |
| 16892836 | 2.14 | 2.01E-07 | 0.003227 | COPS8 | COP9 signalosome subunit 8; NULL |
| 17020152 | 2.14 | 0.000625 | 0.061791 | ELOVL5 | ELOVL fatty acid elongase 5; NULL |
| 17078983 | 2.14 | 0.005234 | 0.162793 | TMEM64 | transmembrane protein 64 |
| 17066897 | 2.13 | 0.000654 | 0.06297 | SLC25A37; FP15737 | solute carrier family 25 (mitochondrial iron transporter), member 37; NULL |
| 17086621 | 2.12 | 0.002208 | 0.113035 | C9orf47; S1PR3 | chromosome 9 open reading frame 47; sphingosine-1-phosphate receptor 3 |
| 16951247 | 2.11 | 0.000044 | 0.021075 | ANKRD28 | ankyrin repeat domain 28; NULL |
| 16687618 | 2.11 | 0.000697 | 0.065101 | DHCR24 | 24-dehydrocholesterol reductase |
| 17011450 | 2.11 | 0.000295 | 0.045972 | FOXO3 | forkhead box O3 |
| 16779667 | 2.11 | 0.030936 | 0.334441 | PCDH9 | protocadherin 9 |
| 17092712 | 2.11 | 0.013474 | 0.239873 | PLIN2; LOC100509484 | perilipin 2; uncharacterized LOC100509484 |
| 16959007 | 2.11 | 0.009783 | 0.210796 | PLXND1 | plexin D1; NULL |
| 16702007 | 2.1 | 0.011966 | 0.229161 | AKR1C3 | aldo-keto reductase family 1, member C3; NULL |
| 16760668 | 2.1 | 0.000009 | 0.011546 | LPCAT3 | lysophosphatidylcholine acyltransferase 3; NULL |
| 16936397 | 2.09 | 0.002053 | 0.108192 | MAPK12 | mitogen-activated protein kinase 12 |
| 16953701 | 2.08 | 0.005267 | 0.162984 | IP6K2 | inositol hexakisphosphate kinase 2; NULL |
| 16817918 | 2.08 | 0.036941 | 0.35738 | MIR4518 | microRNA 4518 |
| 17025417 | 2.07 | 0.001278 | 0.086384 | AGPAT4 | 1-acylglycerol-3-phosphate O-acyltransferase 4; NULL |
| 16761858 | 2.07 | 0.000039 | 0.020908 | RERG | RAS-like, estrogen-regulated, growth inhibitor; NULL |
| 16891575 | 2.06 | 0.000619 | 0.06168 | ACSL3 | acyl-CoA synthetase long-chain family member 3; NULL |
| 16682120 | 2.06 | 0.029795 | 0.329945 | ARHGEF19 | Rho guanine nucleotide exchange factor (GEF) 19 |
| 16994002 | 2.06 | 0.000176 | 0.038908 | LPCAT1 | lysophosphatidylcholine acyltransferase 1; NULL |
| 17112996 | 2.06 | 0.000136 | 0.034782 | MORF4L2 | mortality factor 4 like 2; NULL |
| 16731461 | 2.06 | 0.000031 | 0.019478 | NNMT | nicotinamide N-methyltransferase |
| 16849992 | 2.06 | 0.001576 | 0.096075 | PCYT2 | phosphate cytidylyltransferase 2, ethanolamine; NULL |
| 16688269 | 2.06 | 0.029221 | 0.327223 | SLC35D1 | solute carrier family 35 (UDP-glucuronic acid/UDP-N-acetylgalactosamine dual transporter), member D1 |
| 16843049 | 2.06 | 0.000878 | 0.074499 | SSH2 | slingshot protein phosphatase 2 |
| 16698816 | 2.05 | 0.004207 | 0.148732 | PLXNA2 | plexin A2; NULL |
| 17108067 | 2.05 | 0.001162 | 0.084239 | SLC6A8 | solute carrier family 6 (neurotransmitter transporter, creatine), member 8; NULL |
| 16883690 | 2.04 | 0.008194 | 0.196563 | IL1RL1 | interleukin 1 receptor-like 1; NULL |
| 16859851 | 2.04 | 0.0061 | 0.174213 | KLHL26 | kelch-like family member 26; NULL |
| 16723680 | 2.04 | 0.000742 | 0.067453 | LDLRAD3 | low density lipoprotein receptor class A domain containing 3 |
| 17005420 | 2.04 | 0.009556 | 0.209589 | LRRC16A | leucine rich repeat containing 16A |
| 16779855 | 2.04 | 0.0291 | 0.326392 | MYCBP2 | MYC binding protein 2, E3 ubiquitin protein ligase |
| 16977396 | 2.04 | 0.001353 | 0.089244 | SCD5 | stearoyl-CoA desaturase 5 |
| 16897026 | 2.04 | 0.002929 | 0.127479 | ZFP36L2 | ZFP36 ring finger protein-like 2 |
| 17080516 | 2.03 | 0.01303 | 0.237042 | ENPP2 | ectonucleotide pyrophosphatase/phosphodiesterase 2 |
| 17005787 | 2.03 | 0.008841 | 0.202788 | HIST1H2AH; HIST1H2AG; HIST1H2AM; HIST1H2AL; HIST1H2AK; HIST1H2AI | histone cluster 1, H2ah; histone cluster 1, H2ag; histone cluster 1, H2am; histone cluster 1, H2al; histone cluster 1, H2ak; histone cluster 1, H2ai |
| 16819244 | 2.03 | 0.000109 | 0.031133 | MT1CP | metallothionein 1C, pseudogene; NULL |
| 16874945 | 2.02 | 0.013348 | 0.239296 | FPR1 | formyl peptide receptor 1 |
| 16922327 | 2.02 | 0.000065 | 0.024931 | ITSN1 | intersectin 1 (SH3 domain protein); NULL |
| 16884335 | 2.01 | 0.001878 | 0.105409 | BCL2L11 | BCL2-like 11 (apoptosis facilitator) |
| 16713019 | 2.01 | 0.031463 | 0.336558 | ZNF438 | zinc finger protein 438 |
| 16966712 | 2 | 0.000179 | 0.038997 | DANCR | differentiation antagonizing non-protein coding RNA; NULL |
| 16806564 | 2 | 0.005539 | 0.166842 | MTMR10 | myotubularin related protein 10; NULL |
|  |  |  |  |  |  |
|  |  |  |  |  |  |
| **Down-regulated genes on day 1** | | |  |  |  |
| Transcript Cluster ID | Fold Change (linear) (Induced vs. Control) | ANOVA p-value (Induced vs. Control) | FDR p-value (Induced vs. Control) | Gene Symbol | Description |
| 16972950 | -2 | 0.01508 | 0.250671 | ANKRD37 | ankyrin repeat domain 37; NULL |
| 16801035 | -2 | 0.001052 | 0.080753 | AP4E1 | adaptor-related protein complex 4, epsilon 1 subunit |
| 16997146 | -2 | 0.004247 | 0.149493 | FOXD1 | forkhead box D1 |
| 16853481 | -2 | 0.000457 | 0.052973 | METTL4 | methyltransferase like 4 |
| 16741334 | -2 | 0.008235 | 0.19671 | MRGPRF | MAS-related GPR, member F |
| 17008105 | -2 | 0.001173 | 0.084438 | TBC1D22B | TBC1 domain family, member 22B |
| 16986409 | -2.01 | 0.045786 | 0.387793 | F2R | coagulation factor II (thrombin) receptor |
| 17024980 | -2.01 | 0.012852 | 0.235944 | FBXO5 | F-box protein 5 |
| 16971966 | -2.01 | 0.013297 | 0.239033 | FNIP2 | folliculin interacting protein 2 |
| 16889563 | -2.01 | 0.002213 | 0.113089 | FZD7 | frizzled family receptor 7 |
| 16988423 | -2.01 | 0.004866 | 0.159311 | PRR16 | proline rich 16 |
| 16831442 | -2.01 | 0.045167 | 0.385797 | TRPV2 | transient receptor potential cation channel, subfamily V, member 2 |
| 16978568 | -2.02 | 0.018822 | 0.274157 | CENPE | centromere protein E, 312kDa |
| 16967614 | -2.02 | 0.005833 | 0.171222 | DCK; MOB1B | deoxycytidine kinase; MOB kinase activator 1B |
| 16682425 | -2.02 | 0.001516 | 0.094225 | RCC2 | regulator of chromosome condensation 2 |
| 17009193 | -2.02 | 0.037133 | 0.358224 | SLC29A1 | solute carrier family 29 (nucleoside transporters), member 1; NULL |
| 16893121 | -2.03 | 0.000172 | 0.038677 | ASB1 | ankyrin repeat and SOCS box containing 1; NULL |
| 16789723 | -2.03 | 0.004483 | 0.152896 | CRIP2 | cysteine-rich protein 2; NULL |
| 17063480 | -2.03 | 0.000131 | 0.034224 | PARP12 | poly (ADP-ribose) polymerase family, member 12; NULL |
| 16924878 | -2.03 | 0.039065 | 0.364687 | TIAM1 | T-cell lymphoma invasion and metastasis 1 |
| 17012281 | -2.03 | 0.01486 | 0.248817 | TPD52L1 | tumor protein D52-like 1; NULL |
| 16789743 | -2.04 | 0.002982 | 0.128943 | CRIP1; OTTHUMG00000029910; AL928654.7 | cysteine-rich protein 1 (intestinal); NULL |
| 16989897 | -2.04 | 0.000113 | 0.031805 | CXXC5 | CXXC finger protein 5; NULL |
| 16697599 | -2.04 | 0.002312 | 0.116273 | DENND1B | DENN/MADD domain containing 1B; NULL |
| 17015637 | -2.04 | 0.043528 | 0.380328 | ELOVL2 | ELOVL fatty acid elongase 2 |
| 17117567 | -2.04 | 0.013218 | 0.238207 | SCN8A | sodium channel, voltage gated, type VIII, alpha subunit |
| 16790614 | -2.05 | 0.000184 | 0.039103 | AJUBA | ajuba LIM protein; NULL |
| 17001299 | -2.05 | 0.007025 | 0.183653 | DPYSL3 | dihydropyrimidinase-like 3; NULL |
| 17042860 | -2.05 | 0.001015 | 0.079947 | GPER | G protein-coupled estrogen receptor 1 |
| 16799637 | -2.05 | 0.023423 | 0.302601 | RAD51 | RAD51 homolog (S. cerevisiae) |
| 16798938 | -2.05 | 0.001437 | 0.091297 | SCG5 | secretogranin V (7B2 protein); NULL |
| 17088148 | -2.05 | 0.000701 | 0.065241 | SNX30 | sorting nexin family member 30 |
| 16969911 | -2.06 | 0.000446 | 0.052973 | ANK2 | ankyrin 2, neuronal; NULL |
| 16703407 | -2.06 | 0.003002 | 0.129279 | APBB1IP | amyloid beta (A4) precursor protein-binding, family B, member 1 interacting protein |
| 16679627 | -2.06 | 0.001436 | 0.091297 | CNST | consortin, connexin sorting protein |
| 16944665 | -2.06 | 0.000653 | 0.06297 | DTX3L | deltex 3-like (Drosophila) |
| 16707221 | -2.06 | 0.001896 | 0.105847 | KIF20B | kinesin family member 20B; NULL |
| 17071086 | -2.06 | 0.010139 | 0.214087 | PLEKHF2 | pleckstrin homology domain containing, family F (with FYVE domain) member 2 |
| 16854904 | -2.06 | 0.000706 | 0.06563 | PSTPIP2 | proline-serine-threonine phosphatase interacting protein 2 |
| 16901068 | -2.07 | 0.00699 | 0.183601 | AFF3 | AF4/FMR2 family, member 3; NULL |
| 17078003 | -2.07 | 0.04513 | 0.385797 | LOC100505718; OTTHUMG00000164460; RP11-600K15.1 | uncharacterized LOC100505718; NULL |
| 16867511 | -2.07 | 0.000636 | 0.062021 | LONP1 | lon peptidase 1, mitochondrial; NULL |
| 16717986 | -2.07 | 0.000773 | 0.068866 | NT5C2 | 5'-nucleotidase, cytosolic II; NULL |
| 17010692 | -2.07 | 0.002421 | 0.119005 | RWDD2A | RWD domain containing 2A |
| 17049676 | -2.07 | 0.013727 | 0.241741 | SERPINE1 | serpin peptidase inhibitor, clade E (nexin, plasminogen activator inhibitor type 1), member 1 |
| 16847949 | -2.08 | 0.000408 | 0.051922 | CEP112 | centrosomal protein 112kDa; NULL |
| 16726945 | -2.08 | 0.000014 | 0.014085 | EHBP1L1 | EH domain binding protein 1-like 1 |
| 16707503 | -2.08 | 0.005101 | 0.161846 | EXOC6 | exocyst complex component 6 |
| 16735332 | -2.08 | 0.016605 | 0.260168 | NLRP10 | NLR family, pyrin domain containing 10 |
| 16951756 | -2.08 | 0.007755 | 0.191306 | SLC4A7 | solute carrier family 4, sodium bicarbonate cotransporter, member 7; NULL |
| 16829085 | -2.08 | 0.022896 | 0.299997 | SLC7A5 | solute carrier family 7 (amino acid transporter light chain, L system), member 5 |
| 16971806 | -2.09 | 0.004828 | 0.158932 | GLRB | glycine receptor, beta |
| 17094064 | -2.09 | 0.000115 | 0.032033 | SHB | Src homology 2 domain containing adaptor protein B |
| 16803710 | -2.1 | 0.001037 | 0.080351 | ARNT2 | aryl-hydrocarbon receptor nuclear translocator 2; NULL |
| 16765513 | -2.1 | 0.00762 | 0.189817 | CBX5 | chromobox homolog 5 |
| 16745997 | -2.1 | 0.001181 | 0.084615 | ETS1 | v-ets erythroblastosis virus E26 oncogene homolog 1 (avian) |
| 17080450 | -2.1 | 0.00052 | 0.056622 | EXT1 | exostosin glycosyltransferase 1 |
| 17019484 | -2.1 | 0.001093 | 0.081445 | GTPBP2 | GTP binding protein 2 |
| 16822831 | -2.1 | 0.005173 | 0.161846 | HAGH | hydroxyacylglutathione hydrolase; NULL |
| 16673154 | -2.1 | 0.001475 | 0.092628 | NUF2 | NUF2, NDC80 kinetochore complex component, homolog (S. cerevisiae); NULL |
| 16768149 | -2.1 | 0.003898 | 0.144359 | RASSF9 | Ras association (RalGDS/AF-6) domain family (N-terminal) member 9 |
| 16802653 | -2.1 | 0.023935 | 0.304163 | THSD4 | thrombospondin, type I, domain containing 4 |
| 16911783 | -2.11 | 0.00036 | 0.050151 | DTD1 | D-tyrosyl-tRNA deacylase 1 |
| 16835158 | -2.11 | 0.011708 | 0.226854 | ITGB3; OTTHUMG00000171957; RP11-290H9.2 | integrin, beta 3 (platelet glycoprotein IIIa, antigen CD61); NULL |
| 17069937 | -2.11 | 0.001134 | 0.082827 | LOC100132891; OTTHUMG00000164497; RP11-383H13.1 | uncharacterized LOC100132891; NULL |
| 16742454 | -2.11 | 0.016242 | 0.257666 | PAK1 | p21 protein (Cdc42/Rac)-activated kinase 1; NULL |
| 16987008 | -2.11 | 0.000168 | 0.038063 | RASA1 | RAS p21 protein activator (GTPase activating protein) 1 |
| 16887810 | -2.12 | 0.001429 | 0.091297 | ZAK; OTTHUMG00000132297; AC013461.1 | sterile alpha motif and leucine zipper containing kinase AZK; NULL |
| 16719417 | -2.13 | 0.01556 | 0.253592 | ADAM12 | ADAM metallopeptidase domain 12; NULL |
| 16884956 | -2.13 | 0.031012 | 0.334673 | STEAP3 | STEAP family member 3, metalloreductase; NULL |
| 16804631 | -2.13 | 0.019407 | 0.278384 | TICRR | TOPBP1-interacting checkpoint and replication regulator |
| 17009482 | -2.14 | 0.03486 | 0.350249 | CENPQ | centromere protein Q |
| 16796414 | -2.14 | 0.000109 | 0.031133 | SYNE3 | spectrin repeat containing, nuclear envelope family member 3 |
| 17060061 | -2.15 | 0.001808 | 0.104171 | ASNS | asparagine synthetase (glutamine-hydrolyzing); NULL |
| 17064105 | -2.15 | 0.046577 | 0.389702 | EZH2 | enhancer of zeste homolog 2 (Drosophila); NULL |
| 16707468 | -2.15 | 0.029007 | 0.326382 | KIF11 | kinesin family member 11 |
| 16802605 | -2.15 | 0.002021 | 0.108077 | LRRC49 | leucine rich repeat containing 49; NULL |
| 16779435 | -2.15 | 0.000002 | 0.00823 | THSD1 | thrombospondin, type I, domain containing 1 |
| 16999421 | -2.16 | 0.003915 | 0.144359 | MARCH3 | membrane-associated ring finger (C3HC4) 3, E3 ubiquitin protein ligase |
| 16716590 | -2.16 | 0.003986 | 0.144845 | MYOF | myoferlin; NULL |
| 16693449 | -2.16 | 0.006228 | 0.175273 | S100A4 | S100 calcium binding protein A4 |
| 16777777 | -2.16 | 0.004399 | 0.151055 | SLC7A1 | solute carrier family 7 (cationic amino acid transporter, y+ system), member 1 |
| 16860644 | -2.17 | 0.000066 | 0.024941 | CEBPG | CCAAT/enhancer binding protein (C/EBP), gamma |
| 16972912 | -2.17 | 0.000349 | 0.049068 | SLC25A4 | solute carrier family 25 (mitochondrial carrier; adenine nucleotide translocator), member 4 |
| 16840902 | -2.18 | 0.008567 | 0.200476 | AURKB | aurora kinase B; NULL |
| 16768675 | -2.18 | 0.000774 | 0.068866 | FGD6 | FYVE, RhoGEF and PH domain containing 6 |
| 17010760 | -2.18 | 0.036144 | 0.354479 | NT5E | 5'-nucleotidase, ecto (CD73); NULL |
| 16691883 | -2.19 | 0.004594 | 0.155086 | FAM72D | family with sequence similarity 72, member D |
| 16705247 | -2.19 | 0.006459 | 0.177612 | NRBF2 | nuclear receptor binding factor 2 |
| 16702547 | -2.19 | 0.000435 | 0.052725 | OPTN | optineurin; NULL |
| 16903537 | -2.2 | 0.007055 | 0.18421 | NMI | N-myc (and STAT) interactor |
| 16958124 | -2.2 | 0.000153 | 0.036354 | PARP9 | poly (ADP-ribose) polymerase family, member 9 |
| 16677556 | -2.2 | 0.001197 | 0.085059 | TGFB2 | transforming growth factor, beta 2 |
| 16663514 | -2.21 | 0.022076 | 0.295042 | CDC20 | cell division cycle 20 |
| 16873060 | -2.21 | 0.002488 | 0.120098 | PLAUR | plasminogen activator, urokinase receptor; NULL |
| 16808340 | -2.22 | 0.007315 | 0.185958 | CATSPER2P1 | cation channel, sperm associated 2 pseudogene 1; NULL |
| 16697674 | -2.22 | 0.006081 | 0.174056 | LINC00862 | long intergenic non-protein coding RNA 862 |
| 16902933 | -2.22 | 0.011478 | 0.225445 | LYPD1 | LY6/PLAUR domain containing 1 |
| 16888912 | -2.22 | 0.049067 | 0.398435 | MYO1B | myosin IB; NULL |
| 16836492 | -2.22 | 0.003863 | 0.144061 | PRR11 | proline rich 11 |
| 16990483 | -2.23 | 0.003646 | 0.140709 | ARHGAP26 | Rho GTPase activating protein 26; NULL |
| 16700400 | -2.23 | 0.007415 | 0.186928 | C1orf198 | chromosome 1 open reading frame 198 |
| 16694701 | -2.23 | 0.001369 | 0.089921 | CRABP2 | cellular retinoic acid binding protein 2 |
| 17117871 | -2.23 | 0.010911 | 0.221927 | HMGA1 | high mobility group AT-hook 1 |
| 17025191 | -2.24 | 0.016672 | 0.26025 | EZR | ezrin |
| 16962911 | -2.24 | 0.042297 | 0.375701 | LRRC15 | leucine rich repeat containing 15 |
| 16826212 | -2.25 | 0.006809 | 0.181633 | C16orf87 | chromosome 16 open reading frame 87 |
| 16877473 | -2.25 | 0.009205 | 0.206167 | GEN1 | GEN1 Holliday junction 5' flap endonuclease |
| 16686158 | -2.25 | 0.00015 | 0.036125 | HYI | hydroxypyruvate isomerase (putative) |
| 17078870 | -2.25 | 0.006817 | 0.181721 | MMP16 | matrix metallopeptidase 16 (membrane-inserted) |
| 17084669 | -2.25 | 0.000259 | 0.043462 | RUSC2 | RUN and SH3 domain containing 2 |
| 16800156 | -2.25 | 0.001508 | 0.093861 | TMEM62 | transmembrane protein 62; NULL |
| 16838359 | -2.26 | 0.007896 | 0.193487 | BIRC5 | baculoviral IAP repeat containing 5; NULL |
| 17087610 | -2.26 | 0.000666 | 0.063337 | MURC | muscle-related coiled-coil protein |
| 16706906 | -2.27 | 0.038027 | 0.361639 | ADIRF; AGAP11 | adipogenesis regulatory factor; ankyrin repeat and GTPase domain Arf GTPase activating protein 11 |
| 16965527 | -2.27 | 0.019958 | 0.282211 | PI4K2B; LOC285540 | phosphatidylinositol 4-kinase type 2 beta; uncharacterized LOC285540 |
| 16961475 | -2.28 | 0.004301 | 0.149976 | EIF5A2 | eukaryotic translation initiation factor 5A2 |
| 16784299 | -2.29 | 0.000047 | 0.021075 | CDKN3 | cyclin-dependent kinase inhibitor 3; NULL |
| 16666224 | -2.29 | 0.042244 | 0.37539 | LHX8 | LIM homeobox 8 |
| 17113079 | -2.29 | 0.00098 | 0.078231 | MORC4 | MORC family CW-type zinc finger 4; NULL |
| 16663621 | -2.29 | 0.020095 | 0.283217 | PTPRF | protein tyrosine phosphatase, receptor type, F; NULL |
| 16873296 | -2.3 | 0.016452 | 0.259242 | PPP1R13L | protein phosphatase 1, regulatory subunit 13 like; NULL |
| 16938875 | -2.3 | 0.017311 | 0.264207 | STAC | SH3 and cysteine rich domain |
| 16862439 | -2.31 | 0.006463 | 0.177612 | AXL | AXL receptor tyrosine kinase |
| 16962380 | -2.31 | 0.029926 | 0.330665 | ETV5 | ets variant 5; NULL |
| 16974626 | -2.31 | 0.003188 | 0.132952 | LDB2 | LIM domain binding 2; NULL |
| 16952912 | -2.31 | 0.000118 | 0.032567 | LRRC2 | leucine rich repeat containing 2 |
| 16958786 | -2.31 | 0.000139 | 0.034855 | MGLL | monoglyceride lipase |
| 17057946 | -2.31 | 0.004439 | 0.151828 | PSPH | phosphoserine phosphatase; NULL |
| 16956897 | -2.31 | 0.000852 | 0.073274 | SENP7 | SUMO1/sentrin specific peptidase 7; NULL |
| 16961003 | -2.32 | 0.000372 | 0.050469 | TRIM59; OTTHUMG00000162252; RP11-432B6.3 | tripartite motif containing 59; NULL |
| 16839220 | -2.33 | 0.0109 | 0.22183 | FAM101B | family with sequence similarity 101, member B |
| 16672390 | -2.33 | 0.000237 | 0.041932 | IFI16 | interferon, gamma-inducible protein 16; NULL |
| 17062985 | -2.33 | 0.018916 | 0.274352 | PODXL | podocalyxin-like; NULL |
| 16749459 | -2.33 | 0.000498 | 0.055704 | PPFIBP1 | PTPRF interacting protein, binding protein 1 (liprin beta 1); NULL |
| 16842673 | -2.33 | 0.001203 | 0.085083 | SPAG5; SGK494 | sperm associated antigen 5; uncharacterized serine/threonine-protein kinase SgK494 |
| 16683574 | -2.33 | 0.001578 | 0.096075 | STPG1 | sperm-tail PG-rich repeat containing 1 |
| 16696425 | -2.33 | 0.020192 | 0.283543 | TNFSF4 | tumor necrosis factor (ligand) superfamily, member 4 |
| 17045198 | -2.34 | 0.004943 | 0.160291 | ANLN | anillin, actin binding protein; NULL |
| 16889530 | -2.34 | 0.004426 | 0.151611 | CDK15 | cyclin-dependent kinase 15; NULL |
| 16818207 | -2.34 | 0.012595 | 0.233268 | FUS | fused in sarcoma; NULL |
| 16991859 | -2.34 | 0.032934 | 0.342647 | HMMR | hyaluronan-mediated motility receptor (RHAMM) |
| 16974968 | -2.34 | 0.001252 | 0.086384 | SEL1L3 | sel-1 suppressor of lin-12-like 3 (C. elegans); NULL |
| 16929615 | -2.35 | 0.002421 | 0.119005 | APOL6 | apolipoprotein L, 6 |
| 16979133 | -2.35 | 0.00006 | 0.024259 | CAMK2D | calcium/calmodulin-dependent protein kinase II delta; NULL |
| 16980470 | -2.35 | 0.000217 | 0.041323 | NR3C2 | nuclear receptor subfamily 3, group C, member 2; NULL |
| 16873562 | -2.35 | 0.001372 | 0.089921 | PTGIR | prostaglandin I2 (prostacyclin) receptor (IP) |
| 16798801 | -2.36 | 0.006747 | 0.181267 | ARHGAP11B | Rho GTPase activating protein 11B |
| 16985599 | -2.36 | 0.016869 | 0.261082 | CCNB1 | cyclin B1; NULL |
| 16672489 | -2.36 | 0.002828 | 0.126344 | LOC100505633 | uncharacterized LOC100505633 |
| 17070061 | -2.36 | 0.000095 | 0.028898 | LY96 | lymphocyte antigen 96 |
| 16774976 | -2.36 | 0.00839 | 0.198826 | WDFY2 | WD repeat and FYVE domain containing 2 |
| 17105401 | -2.37 | 0.022317 | 0.297023 | CENPI | centromere protein I; NULL |
| 17106438 | -2.37 | 0.005534 | 0.166802 | DOCK11 | dedicator of cytokinesis 11 |
| 17005589 | -2.37 | 0.045418 | 0.386109 | HIST1H2AE; HIST1H2AB | histone cluster 1, H2ae; histone cluster 1, H2ab |
| 16733553 | -2.37 | 0.00365 | 0.140709 | NTM; LOC100653217 | neurotrimin; NULL; neurotrimin-like |
| 16944695 | -2.37 | 0.003615 | 0.140358 | PARP14 | poly (ADP-ribose) polymerase family, member 14 |
| 16916901 | -2.37 | 0.006873 | 0.182149 | RASSF2 | Ras association (RalGDS/AF-6) domain family member 2 |
| 16982635 | -2.37 | 0.022662 | 0.298831 | TRIP13 | thyroid hormone receptor interactor 13 |
| 16799739 | -2.38 | 0.021127 | 0.288708 | CHAC1 | ChaC, cation transport regulator homolog 1 (E. coli) |
| 16797196 | -2.39 | 0.00235 | 0.117332 | AHNAK2 | AHNAK nucleoprotein 2 |
| 17067102 | -2.39 | 0.004247 | 0.149493 | CDCA2 | cell division cycle associated 2 |
| 16912379 | -2.39 | 0.007952 | 0.194294 | TPX2 | TPX2, microtubule-associated, homolog (Xenopus laevis) |
| 16804559 | -2.4 | 0.008789 | 0.202402 | FANCI | Fanconi anemia, complementation group I; NULL |
| 17012148 | -2.4 | 0.009646 | 0.210084 | GJA1 | gap junction protein, alpha 1, 43kDa |
| 16767794 | -2.4 | 0.003173 | 0.132786 | OSBPL8 | oxysterol binding protein-like 8; NULL |
| 16691090 | -2.4 | 0.013334 | 0.239232 | PTPN22 | protein tyrosine phosphatase, non-receptor type 22 (lymphoid); NULL |
| 17025152 | -2.4 | 0.017608 | 0.266358 | SERAC1 | serine active site containing 1 |
| 16820620 | -2.4 | 0.000062 | 0.024317 | WWP2 | WW domain containing E3 ubiquitin protein ligase 2; NULL |
| 16719515 | -2.41 | 0.00543 | 0.165564 | MKI67 | antigen identified by monoclonal antibody Ki-67 |
| 16769569 | -2.41 | 0.005122 | 0.161846 | NUAK1 | NUAK family, SNF1-like kinase, 1 |
| 16828810 | -2.41 | 0.00349 | 0.137508 | TLDC1; KIAA1609 | TBC/LysM-associated domain containing 1; NULL |
| 16701185 | -2.42 | 0.001221 | 0.085471 | CEP170; CEP170P1 | centrosomal protein 170kDa; centrosomal protein 170kDa pseudogene 1; NULL |
| 16834409 | -2.42 | 0.022937 | 0.300146 | CNTNAP1 | contactin associated protein 1 |
| 16694617 | -2.42 | 0.002486 | 0.120098 | IQGAP3 | IQ motif containing GTPase activating protein 3 |
| 16962921 | -2.43 | 0.000958 | 0.077324 | ATP13A3; LOC100507033; OTTHUMG00000156035; AC108676.1 | ATPase type 13A3; uncharacterized LOC100507033; NULL |
| 16773700 | -2.43 | 0.000216 | 0.041323 | B3GALTL | beta 1,3-galactosyltransferase-like |
| 16791898 | -2.43 | 0.000018 | 0.015346 | CFL2 | cofilin 2 (muscle) |
| 16761631 | -2.43 | 0.00042 | 0.052365 | DUSP16 | dual specificity phosphatase 16 |
| 16979845 | -2.43 | 0.042821 | 0.37753 | KRT18P54 | NULL; keratin 18 pseudogene 54 |
| 16809748 | -2.43 | 0.023778 | 0.303525 | MNS1 | meiosis-specific nuclear structural 1 |
| 16692667 | -2.43 | 0.001079 | 0.081031 | MTMR11 | myotubularin related protein 11; NULL |
| 17051286 | -2.44 | 0.029765 | 0.329854 | FLNC | filamin C, gamma |
| 16960844 | -2.44 | 0.004703 | 0.156411 | VEPH1 | ventricular zone expressed PH domain-containing 1; NULL |
| 16968213 | -2.45 | 0.018759 | 0.273727 | ANXA3 | annexin A3; NULL |
| 16706180 | -2.45 | 0.000794 | 0.069654 | PLAU | plasminogen activator, urokinase |
| 17024775 | -2.45 | 0.000467 | 0.053448 | SYNE1 | spectrin repeat containing, nuclear envelope 1; NULL |
| 17092512 | -2.46 | 0.001601 | 0.097142 | ZDHHC21 | zinc finger, DHHC-type containing 21 |
| 16819430 | -2.47 | 0.002731 | 0.124684 | CPNE2 | copine II; NULL |
| 17011593 | -2.47 | 0.000028 | 0.019043 | FIG4 | FIG4 homolog, SAC1 lipid phosphatase domain containing (S. cerevisiae) |
| 16711299 | -2.47 | 0.007061 | 0.184271 | LINC00702 | NULL; long intergenic non-protein coding RNA 702 |
| 16771680 | -2.48 | 0.000004 | 0.010118 | CLIP1 | CAP-GLY domain containing linker protein 1; NULL |
| 16661687 | -2.48 | 0.009709 | 0.210084 | EPB41 | erythrocyte membrane protein band 4.1 (elliptocytosis 1, RH-linked) |
| 17106357 | -2.48 | 0.000311 | 0.047335 | PLS3 | plastin 3; NULL |
| 16717359 | -2.49 | 0.000004 | 0.010118 | GOT1 | glutamic-oxaloacetic transaminase 1, soluble; NULL |
| 17007910 | -2.49 | 0.000646 | 0.062573 | MAPK13 | mitogen-activated protein kinase 13 |
| 16799793 | -2.49 | 0.021625 | 0.29212 | NUSAP1 | nucleolar and spindle associated protein 1; NULL |
| 16956149 | -2.5 | 0.000313 | 0.04746 | FOXP1 | forkhead box P1; NULL |
| 16929573 | -2.5 | 0.016455 | 0.259242 | MCM5 | minichromosome maintenance complex component 5; NULL |
| 16737344 | -2.5 | 0.006053 | 0.174006 | PAMR1 | peptidase domain containing associated with muscle regeneration 1 |
| 16773840 | -2.51 | 0.008133 | 0.196205 | BRCA2 | breast cancer 2, early onset |
| 17059756 | -2.51 | 0.001857 | 0.105127 | CDK6 | cyclin-dependent kinase 6 |
| 16968797 | -2.51 | 0.012052 | 0.229591 | HERC3 | HECT and RLD domain containing E3 ubiquitin protein ligase 3; NULL |
| 16690704 | -2.51 | 0.039977 | 0.368134 | SLC16A4 | solute carrier family 16, member 4 (monocarboxylic acid transporter 5); NULL |
| 16703563 | -2.52 | 0.00113 | 0.082729 | BAMBI | BMP and activin membrane-bound inhibitor homolog (Xenopus laevis) |
| 16677425 | -2.52 | 0.002197 | 0.112778 | CENPF | centromere protein F, 350/400kDa; NULL |
| 16995645 | -2.52 | 0.000015 | 0.014242 | DAB2 | Dab, mitogen-responsive phosphoprotein, homolog 2 (Drosophila); NULL |
| 17016499 | -2.52 | 0.031145 | 0.33564 | HIST1H1B | histone cluster 1, H1b |
| 16920730 | -2.53 | 0.023 | 0.300427 | APCDD1L | adenomatosis polyposis coli down-regulated 1-like |
| 16892075 | -2.53 | 0.002008 | 0.10805 | ARMC9 | armadillo repeat containing 9; NULL |
| 16899413 | -2.53 | 0.000397 | 0.051216 | EVA1A | eva-1 homolog A (C. elegans); NULL |
| 16764398 | -2.53 | 0.004084 | 0.14656 | FMNL3 | formin-like 3; NULL |
| 16687875 | -2.53 | 0.021941 | 0.294328 | JUN | jun proto-oncogene |
| 16922759 | -2.54 | 0.0107 | 0.220326 | KCNJ15 | potassium inwardly-rectifying channel, subfamily J, member 15; NULL |
| 17066018 | -2.54 | 0.000473 | 0.053638 | ZDHHC2 | zinc finger, DHHC-type containing 2 |
| 16929442 | -2.55 | 0.000062 | 0.024317 | TIMP3 | TIMP metallopeptidase inhibitor 3 |
| 16922501 | -2.56 | 0.000567 | 0.058703 | DOPEY2 | dopey family member 2 |
| 17056506 | -2.56 | 0.000016 | 0.014437 | RP9P | retinitis pigmentosa 9 pseudogene |
| 16789149 | -2.56 | 0.001387 | 0.090274 | TNFAIP2 | tumor necrosis factor, alpha-induced protein 2; NULL |
| 17061129 | -2.57 | 0.041795 | 0.373518 | RASA4; RASA4B; UPK3BL; OTTHUMG00000180800; RP11-514P8.6 | RAS p21 protein activator 4; RAS p21 protein activator 4B; NULL |
| 17074848 | -2.58 | 0.000031 | 0.019478 | DLC1 | deleted in liver cancer 1; NULL |
| 16957170 | -2.58 | 0.001955 | 0.107324 | KIAA1524 | KIAA1524 |
| 16981730 | -2.58 | 0.004123 | 0.147047 | VEGFC | vascular endothelial growth factor C |
| 16796694 | -2.58 | 0.000239 | 0.041932 | WARS | tryptophanyl-tRNA synthetase; NULL |
| 16826539 | -2.59 | 0.000002 | 0.008241 | AKTIP; OTTHUMG00000177947; RP11-44F14.11; OTTHUMG00000161912; CTD-2015B23.2 | AKT interacting protein; NULL |
| 16972616 | -2.59 | 0.00815 | 0.196205 | NEIL3 | nei endonuclease VIII-like 3 (E. coli) |
| 16835436 | -2.6 | 0.044959 | 0.385248 | LOC100506373 | uncharacterized LOC100506373 |
| 17010198 | -2.6 | 0.005871 | 0.171658 | RIMS1 | regulating synaptic membrane exocytosis 1; NULL |
| 16906571 | -2.6 | 0.001534 | 0.094693 | STAT4 | signal transducer and activator of transcription 4; NULL |
| 16673126 | -2.61 | 0.012477 | 0.232698 | RGS4 | regulator of G-protein signaling 4; NULL |
| 16751319 | -2.61 | 0.00139 | 0.090274 | SCN8A | sodium channel, voltage gated, type VIII, alpha subunit |
| 17010246 | -2.62 | 0.037357 | 0.359423 | KCNQ5 | potassium voltage-gated channel, KQT-like subfamily, member 5 |
| 16712576 | -2.62 | 0.035902 | 0.353859 | PRTFDC1 | phosphoribosyl transferase domain containing 1 |
| 17065938 | -2.62 | 0.005167 | 0.161846 | RNA5SP255 | RNA, 5S ribosomal pseudogene 255 |
| 16804062 | -2.62 | 0.006631 | 0.179459 | TM6SF1 | transmembrane 6 superfamily member 1 |
| 16715793 | -2.63 | 0.006771 | 0.181451 | KCNMA1 | potassium large conductance calcium-activated channel, subfamily M, alpha member 1; NULL |
| 16798919 | -2.64 | 0.007098 | 0.184529 | ARHGAP11A | Rho GTPase activating protein 11A |
| 16798951 | -2.64 | 0.000544 | 0.057743 | GREM1 | gremlin 1, DAN family BMP antagonist |
| 16979825 | -2.64 | 0.020007 | 0.282502 | PABPC4L | poly(A) binding protein, cytoplasmic 4-like |
| 16820508 | -2.64 | 0.001209 | 0.085244 | TANGO6; TMCO7 | transport and golgi organization 6 homolog (Drosophila); NULL |
| 16665447 | -2.65 | 0.004003 | 0.145167 | USP1 | ubiquitin specific peptidase 1 |
| 17062127 | -2.65 | 0.006144 | 0.174596 | WNT2 | wingless-type MMTV integration site family member 2 |
| 16886717 | -2.66 | 0.032146 | 0.338967 | GALNT5 | UDP-N-acetyl-alpha-D-galactosamine:polypeptide N-acetylgalactosaminyltransferase 5 (GalNAc-T5) |
| 16755498 | -2.66 | 0.001811 | 0.104171 | TMPO | thymopoietin; NULL |
| 16901957 | -2.67 | 0.007383 | 0.1867 | CKAP2L | cytoskeleton associated protein 2-like; NULL |
| 16874005 | -2.67 | 0.019766 | 0.280941 | DBP | D site of albumin promoter (albumin D-box) binding protein |
| 16692724 | -2.69 | 0.002986 | 0.128943 | ANP32E | acidic (leucine-rich) nuclear phosphoprotein 32 family, member E |
| 16971382 | -2.69 | 0.019606 | 0.279735 | DCLK2 | doublecortin-like kinase 2 |
| 16711909 | -2.69 | 0.035036 | 0.350696 | FRMD4A | FERM domain containing 4A; NULL |
| 16855600 | -2.7 | 0.011014 | 0.222053 | CCBE1 | collagen and calcium binding EGF domains 1 |
| 16707180 | -2.7 | 0.000948 | 0.077324 | IFIT2 | interferon-induced protein with tetratricopeptide repeats 2 |
| 16663958 | -2.7 | 0.00438 | 0.150966 | KIF2C | kinesin family member 2C; NULL |
| 16669087 | -2.7 | 0.001409 | 0.091097 | SLC22A15 | solute carrier family 22, member 15 |
| 16970080 | -2.71 | 0.006572 | 0.179354 | CEP170P1 | centrosomal protein 170kDa pseudogene 1 |
| 16981266 | -2.71 | 0.000233 | 0.041768 | DDX60L | DEAD (Asp-Glu-Ala-Asp) box polypeptide 60-like; NULL |
| 16942919 | -2.71 | 0.000858 | 0.073466 | HTR1F | 5-hydroxytryptamine (serotonin) receptor 1F, G protein-coupled |
| 17087588 | -2.71 | 0.000113 | 0.031805 | TMEFF1; MSANTD3-TMEFF1; MSANTD3 | transmembrane protein with EGF-like and two follistatin-like domains 1; MSANTD3-TMEFF1 readthrough; Myb/SANT-like DNA-binding domain containing 3 |
| 16784098 | -2.72 | 0.000959 | 0.077324 | FRMD6 | FERM domain containing 6; NULL |
| 16901974 | -2.72 | 0.007484 | 0.187646 | IL1A | interleukin 1, alpha |
| 16899357 | -2.72 | 0.002456 | 0.119562 | LOXL3 | lysyl oxidase-like 3; NULL |
| 16808304 | -2.72 | 0.003429 | 0.136449 | PPIP5K1 | diphosphoinositol pentakisphosphate kinase 1 |
| 16910609 | -2.72 | 0.000227 | 0.041323 | TRIB3 | tribbles homolog 3 (Drosophila) |
| 17013283 | -2.72 | 0.000054 | 0.022892 | UTRN | utrophin; NULL |
| 17063221 | -2.73 | 0.036377 | 0.355548 | FAM180A | family with sequence similarity 180, member A |
| 17084878 | -2.74 | 0.001169 | 0.084429 | CCIN | calicin |
| 16990553 | -2.74 | 0.000167 | 0.038063 | KCTD16 | potassium channel tetramerisation domain containing 16 |
| 16989636 | -2.74 | 0.003087 | 0.130574 | KIF20A | kinesin family member 20A; NULL |
| 16819082 | -2.74 | 0.010056 | 0.212797 | LPCAT2 | lysophosphatidylcholine acyltransferase 2; NULL |
| 16763600 | -2.75 | 0.005237 | 0.162793 | AMIGO2 | adhesion molecule with Ig-like domain 2 |
| 16688386 | -2.75 | 0.004814 | 0.158701 | DEPDC1 | DEP domain containing 1 |
| 17019698 | -2.75 | 0.010854 | 0.221699 | RCAN2 | regulator of calcineurin 2 |
| 16670894 | -2.75 | 0.000014 | 0.014085 | TUFT1 | tuftelin 1 |
| 17092870 | -2.76 | 0.001863 | 0.105149 | MIR31HG | MIR31 host gene (non-protein coding) |
| 16818842 | -2.77 | 0.000005 | 0.010118 | CYLD | cylindromatosis (turban tumor syndrome); NULL |
| 16838330 | -2.78 | 0.001828 | 0.104465 | SYNGR2 | synaptogyrin 2; NULL |
| 16858970 | -2.79 | 0.007237 | 0.185752 | CD97 | CD97 molecule |
| 16907303 | -2.8 | 0.000001 | 0.006578 | TMEM237 | transmembrane protein 237; NULL |
| 16694361 | -2.82 | 0.000081 | 0.027291 | ARHGEF2; OTTHUMG00000017458; RP11-336K24.4 | Rho/Rac guanine nucleotide exchange factor (GEF) 2; NULL |
| 16660785 | -2.82 | 0.000373 | 0.050469 | NIPAL3 | NIPA-like domain containing 3; NULL |
| 16980946 | -2.83 | 0.005008 | 0.16049 | PDGFC | platelet derived growth factor C; NULL |
| 16852179 | -2.83 | 0.031455 | 0.336558 | SLC14A1 | solute carrier family 14 (urea transporter), member 1 (Kidd blood group); NULL |
| 16682098 | -2.84 | 0.000105 | 0.030607 | EPHA2 | EPH receptor A2 |
| 17005858 | -2.84 | 0.019966 | 0.282211 | HIST1H2AI; HIST1H2AH; HIST1H2AG; HIST1H2AM; HIST1H2AL; HIST1H2AK; HIST1H3F | histone cluster 1, H2ai; histone cluster 1, H2ah; histone cluster 1, H2ag; histone cluster 1, H2am; histone cluster 1, H2al; histone cluster 1, H2ak; histone cluster 1, H3f |
| 16969686 | -2.85 | 0.000389 | 0.050838 | CCDC109B | coiled-coil domain containing 109B |
| 17084904 | -2.86 | 0.017184 | 0.263668 | MELK | maternal embryonic leucine zipper kinase; NULL |
| 16707551 | -2.87 | 0.009866 | 0.211466 | CEP55 | centrosomal protein 55kDa |
| 16801557 | -2.89 | 0.000625 | 0.061791 | CCNB2 | cyclin B2; NULL |
| 16674845 | -2.89 | 0.00782 | 0.192216 | LAMC2 | laminin, gamma 2 |
| 16850517 | -2.92 | 0.005267 | 0.162984 | NDC80 | NDC80 kinetochore complex component |
| 16942103 | -2.93 | 0.003751 | 0.142044 | FLNB | filamin B, beta; NULL |
| 16894283 | -2.93 | 0.001263 | 0.086384 | MBOAT2 | membrane bound O-acyltransferase domain containing 2; NULL |
| 16920548 | -2.94 | 0.004016 | 0.145273 | AURKA | aurora kinase A; NULL |
| 16815310 | -2.94 | 0.000915 | 0.076215 | TNFRSF12A | tumor necrosis factor receptor superfamily, member 12A |
| 16979339 | -2.96 | 0.000075 | 0.026566 | PDE5A | phosphodiesterase 5A, cGMP-specific; NULL |
| 16817017 | -2.96 | 0.008896 | 0.203052 | PLK1 | polo-like kinase 1; NULL |
| 16830202 | -2.96 | 0.000087 | 0.027826 | XAF1 | XIAP associated factor 1; NULL |
| 16775434 | -2.97 | 0.000124 | 0.032886 | LMO7; OTTHUMG00000172802; RP11-29G8.3 | LIM domain 7; NULL |
| 16902945 | -2.97 | 0.002952 | 0.128391 | NCKAP5 | NCK-associated protein 5 |
| 17055937 | -2.97 | 0.000081 | 0.027291 | OSBPL3 | oxysterol binding protein-like 3; NULL |
| 16818773 | -2.98 | 0.00045 | 0.052973 | ADCY7 | adenylate cyclase 7; NULL |
| 16844775 | -2.98 | 0.018945 | 0.274443 | KRT19 | keratin 19 |
| 16717272 | -2.98 | 0.001637 | 0.098071 | LOXL4 | lysyl oxidase-like 4 |
| 17010552 | -2.98 | 0.003684 | 0.141139 | TTK | TTK protein kinase; NULL |
| 16813342 | -2.99 | 0.008472 | 0.199993 | PRC1 | protein regulator of cytokinesis 1; NULL |
| 17012546 | -3 | 0.003283 | 0.133706 | TMEM200A | transmembrane protein 200A |
| 16793225 | -3.01 | 0.011023 | 0.222151 | DLGAP5 | discs, large (Drosophila) homolog-associated protein 5 |
| 16707196 | -3.01 | 0.000065 | 0.024931 | IFIT1 | interferon-induced protein with tetratricopeptide repeats 1 |
| 16723422 | -3.01 | 0.044726 | 0.384276 | KIAA1549L | KIAA1549-like |
| 16736891 | -3.01 | 0.001716 | 0.101278 | KIF18A | kinesin family member 18A |
| 16744205 | -3.02 | 0.00195 | 0.107283 | ARHGAP20 | Rho GTPase activating protein 20 |
| 16820849 | -3.05 | 0.038209 | 0.362658 | CALB2 | calbindin 2 |
| 16697544 | -3.06 | 0.000583 | 0.05966 | ASPM | asp (abnormal spindle) homolog, microcephaly associated (Drosophila) |
| 17067332 | -3.07 | 0.047308 | 0.392615 | ESCO2 | establishment of sister chromatid cohesion N-acetyltransferase 2; NULL |
| 17002667 | -3.07 | 0.015798 | 0.254835 | FAM196B | family with sequence similarity 196, member B |
| 16688799 | -3.09 | 0.002592 | 0.12268 | ELTD1 | EGF, latrophilin and seven transmembrane domain containing 1 |
| 16909081 | -3.1 | 0.030727 | 0.333602 | DOCK10 | dedicator of cytokinesis 10; NULL |
| 16909958 | -3.1 | 0.006173 | 0.174915 | PER2 | period circadian clock 2 |
| 16965377 | -3.1 | 0.001114 | 0.082539 | SLIT2 | slit homolog 2 (Drosophila) |
| 16996722 | -3.11 | 0.030406 | 0.332568 | CENPK | centromere protein K; NULL |
| 16901393 | -3.13 | 0.000023 | 0.01717 | FHL2 | four and a half LIM domains 2 |
| 16901624 | -3.14 | 0.000517 | 0.056622 | MALL | mal, T-cell differentiation protein-like |
| 17067696 | -3.15 | 0.000777 | 0.068866 | NRG1 | neuregulin 1 |
| 16707184 | -3.16 | 0.00044 | 0.052725 | IFIT3 | interferon-induced protein with tetratricopeptide repeats 3 |
| 16852858 | -3.16 | 0.042457 | 0.376243 | SERPINB7 | serpin peptidase inhibitor, clade B (ovalbumin), member 7 |
| 16777278 | -3.16 | 0.007816 | 0.192216 | SKA3 | spindle and kinetochore associated complex subunit 3 |
| 17019190 | -3.17 | 0.000098 | 0.029473 | C6orf132 | chromosome 6 open reading frame 132 |
| 16908171 | -3.17 | 0.000464 | 0.053413 | MARCH4 | membrane-associated ring finger (C3HC4) 4, E3 ubiquitin protein ligase |
| 17059776 | -3.19 | 0.000416 | 0.052263 | SAMD9L | sterile alpha motif domain containing 9-like |
| 16778274 | -3.21 | 0.015883 | 0.255658 | TRPC4 | transient receptor potential cation channel, subfamily C, member 4; NULL |
| 16962632 | -3.22 | 0.006109 | 0.174213 | LEPREL1 | leprecan-like 1; NULL |
| 16689354 | -3.23 | 0.000013 | 0.014085 | GBP2 | guanylate binding protein 2, interferon-inducible; NULL |
| 16901755 | -3.24 | 0.001892 | 0.105826 | BUB1 | BUB1 mitotic checkpoint serine/threonine kinase; NULL |
| 16705159 | -3.25 | 0.01113 | 0.222811 | CDK1 | cyclin-dependent kinase 1 |
| 16689312 | -3.3 | 0.011566 | 0.225687 | GBP3 | guanylate binding protein 3; NULL |
| 17005865 | -3.3 | 0.008698 | 0.201671 | HIST1H2BM | histone cluster 1, H2bm |
| 16979225 | -3.3 | 0.004018 | 0.145273 | PRSS12 | protease, serine, 12 (neurotrypsin, motopsin) |
| 16886491 | -3.3 | 0.012289 | 0.231145 | TNFAIP6 | tumor necrosis factor, alpha-induced protein 6 |
| 17096904 | -3.31 | 0.000183 | 0.039103 | CTNNAL1 | catenin (cadherin-associated protein), alpha-like 1 |
| 17016403 | -3.32 | 0.0149 | 0.248994 | HIST1H3G; HIST1H3F; HIST1H3B; HIST1H3H; HIST1H3J; HIST1H3I; HIST1H3E; HIST1H3C; HIST1H3D; HIST1H3A | histone cluster 1, H3g; histone cluster 1, H3f; histone cluster 1, H3b; histone cluster 1, H3h; histone cluster 1, H3j; histone cluster 1, H3i; histone cluster 1, H3e; histone cluster 1, H3c; histone cluster 1, H3d; histone cluster 1, H3a |
| 16911283 | -3.32 | 0.00037 | 0.050469 | PLCB1 | phospholipase C, beta 1 (phosphoinositide-specific) |
| 16808334 | -3.33 | 0.000374 | 0.050469 | PPIP5K1; OTTHUMG00000059903; AC011330.5 | diphosphoinositol pentakisphosphate kinase 1; NULL |
| 16788036 | -3.37 | 0.000315 | 0.047597 | BDKRB1 | bradykinin receptor B1 |
| 16965346 | -3.37 | 0.026092 | 0.314335 | NCAPG | non-SMC condensin I complex, subunit G; NULL |
| 17069886 | -3.37 | 0.023281 | 0.301965 | TRAM1 | translocation associated membrane protein 1 |
| 16722081 | -3.38 | 0.00013 | 0.03407 | MICAL2 | microtubule associated monooxygenase, calponin and LIM domain containing 2; NULL |
| 17016263 | -3.39 | 0.011471 | 0.225445 | CMAHP | cytidine monophospho-N-acetylneuraminic acid hydroxylase, pseudogene; NULL |
| 16988703 | -3.4 | 0.038014 | 0.361639 | LMNB1 | lamin B1 |
| 16847432 | -3.41 | 0.044649 | 0.384095 | BRIP1 | BRCA1 interacting protein C-terminal helicase 1 |
| 16742384 | -3.41 | 0.000177 | 0.038908 | LRRC32 | leucine rich repeat containing 32 |
| 16768923 | -3.42 | 0.026551 | 0.316398 | SLC9A7P1 | solute carrier family 9, subfamily A (NHE7, cation proton antiporter 7), member 7 pseudogene 1 |
| 17014114 | -3.42 | 0.000183 | 0.039103 | SYNJ2 | synaptojanin 2; NULL |
| 16802519 | -3.43 | 0.000794 | 0.069654 | KIF23 | kinesin family member 23 |
| 16826160 | -3.43 | 0.004143 | 0.147213 | SHCBP1 | SHC SH2-domain binding protein 1 |
| 17012342 | -3.44 | 0.000036 | 0.020495 | HINT3 | histidine triad nucleotide binding protein 3 |
| 16796412 | -3.46 | 0.000692 | 0.064754 | LINC00341 | long intergenic non-protein coding RNA 341 |
| 17045078 | -3.47 | 0.005061 | 0.161313 | BMPER | BMP binding endothelial regulator |
| 16841340 | -3.49 | 0.004388 | 0.150966 | MYH2 | myosin, heavy chain 2, skeletal muscle, adult |
| 16658536 | -3.49 | 0.002219 | 0.113232 | PER3 | period circadian clock 3 |
| 16697695 | -3.5 | 0.005702 | 0.169627 | KIF14 | kinesin family member 14 |
| 16672214 | -3.5 | 0.000247 | 0.042317 | PEAR1 | platelet endothelial aggregation receptor 1; NULL |
| 16974121 | -3.54 | 0.000193 | 0.039211 | AFAP1 | actin filament associated protein 1 |
| 17066224 | -3.54 | 0.000053 | 0.022867 | SH2D4A | SH2 domain containing 4A |
| 16938654 | -3.56 | 0.001119 | 0.08259 | KRT18P15 | NULL; keratin 18 pseudogene 15 |
| 16746290 | -3.58 | 0.00365 | 0.140709 | OPCML; LOC100653275 | opioid binding protein/cell adhesion molecule-like; NULL; uncharacterized LOC100653275 |
| 17075776 | -3.58 | 0.003247 | 0.133571 | PBK | PDZ binding kinase |
| 16854856 | -3.59 | 0.000046 | 0.021075 | EPG5 | ectopic P-granules autophagy protein 5 homolog (C. elegans) |
| 16736821 | -3.59 | 0.000537 | 0.057426 | LGR4 | leucine-rich repeat containing G protein-coupled receptor 4 |
| 16811638 | -3.59 | 0.000771 | 0.068866 | SEMA7A | semaphorin 7A, GPI membrane anchor (John Milton Hagen blood group) |
| 16987610 | -3.62 | 0.000098 | 0.029473 | RGMB | RGM domain family, member B; NULL |
| 16997383 | -3.63 | 0.043793 | 0.380948 | F2RL2 | coagulation factor II (thrombin) receptor-like 2 |
| 17056984 | -3.67 | 0.016672 | 0.26025 | INHBA | inhibin, beta A |
| 16800229 | -3.69 | 0.000617 | 0.061567 | MAP1A | microtubule-associated protein 1A |
| 16851383 | -3.7 | 0.012472 | 0.232698 | GATA6 | GATA binding protein 6 |
| 17050591 | -3.7 | 0.049272 | 0.398811 | MET | met proto-oncogene (hepatocyte growth factor receptor) |
| 17000650 | -3.7 | 0.000106 | 0.030881 | TMEM173 | transmembrane protein 173; NULL |
| 16783602 | -3.72 | 0.007128 | 0.184636 | SSTR1 | somatostatin receptor 1 |
| 16807763 | -3.76 | 0.001721 | 0.101452 | EHD4 | EH-domain containing 4 |
| 16818114 | -3.76 | 0.000166 | 0.038063 | HSD3B7 | hydroxy-delta-5-steroid dehydrogenase, 3 beta- and steroid delta-isomerase 7 |
| 16947551 | -3.79 | 0.004347 | 0.150481 | C3orf80 | chromosome 3 open reading frame 80 |
| 16920338 | -3.81 | 0.000093 | 0.028629 | ATP9A | ATPase, class II, type 9A |
| 17016363 | -3.82 | 0.02201 | 0.294548 | HIST1H3B; HIST1H3F; HIST1H3H; HIST1H3J; HIST1H3G; HIST1H3I; HIST1H3E; HIST1H3C; HIST1H3D; HIST1H3A | histone cluster 1, H3b; histone cluster 1, H3f; histone cluster 1, H3h; histone cluster 1, H3j; histone cluster 1, H3g; histone cluster 1, H3i; histone cluster 1, H3e; histone cluster 1, H3c; histone cluster 1, H3d; histone cluster 1, H3a |
| 16905528 | -3.85 | 0.001706 | 0.100796 | PDE11A | phosphodiesterase 11A; NULL |
| 16811249 | -3.89 | 0.000008 | 0.011546 | UACA | uveal autoantigen with coiled-coil domains and ankyrin repeats; NULL |
| 17078183 | -3.92 | 0.000437 | 0.052725 | MSC | musculin |
| 16852871 | -3.97 | 0.006325 | 0.176622 | SERPINB2; SERPINB10 | serpin peptidase inhibitor, clade B (ovalbumin), member 2; NULL; serpin peptidase inhibitor, clade B (ovalbumin), member 10 |
| 17083793 | -4.03 | 0.000168 | 0.038063 | ADAMTSL1 | ADAMTS-like 1; NULL |
| 16835672 | -4.1 | 0.018247 | 0.271424 | ITGA3 | integrin, alpha 3 (antigen CD49C, alpha 3 subunit of VLA-3 receptor); NULL |
| 16767851 | -4.12 | 0.00039 | 0.050838 | E2F7 | E2F transcription factor 7; NULL |
| 16833567 | -4.15 | 0.000099 | 0.029542 | DUSP14 | dual specificity phosphatase 14 |
| 16828886 | -4.15 | 0.021165 | 0.288914 | GINS2 | GINS complex subunit 2 (Psf2 homolog) |
| 16858137 | -4.2 | 0.002545 | 0.121542 | ICAM1 | intercellular adhesion molecule 1 |
| 16847841 | -4.23 | 0.000462 | 0.053322 | SMURF2 | SMAD specific E3 ubiquitin protein ligase 2; NULL |
| 16697196 | -4.24 | 0.000188 | 0.039211 | FAM129A | family with sequence similarity 129, member A |
| 17058968 | -4.31 | 0.019915 | 0.28208 | FGL2 | fibrinogen-like 2 |
| 16682077 | -4.44 | 0.010967 | 0.221978 | HSPB7 | heat shock 27kDa protein family, member 7 (cardiovascular) |
| 16679301 | -4.45 | 0.000054 | 0.022892 | FMN2 | formin 2; NULL |
| 16968331 | -4.46 | 0.001809 | 0.104171 | FGF5 | fibroblast growth factor 5 |
| 16967831 | -4.53 | 0.03975 | 0.367291 | EPGN | epithelial mitogen |
| 16951485 | -4.59 | 0.006871 | 0.182149 | SGOL1 | shugoshin-like 1 (S. pombe); NULL |
| 16716478 | -4.6 | 0.001819 | 0.104308 | ANKRD1 | ankyrin repeat domain 1 (cardiac muscle) |
| 16768738 | -4.69 | 0.007655 | 0.1903 | NTN4 | netrin 4 |
| 16755908 | -4.72 | 0.000466 | 0.053448 | DRAM1 | DNA-damage regulated autophagy modulator 1 |
| 16764724 | -4.73 | 0.00536 | 0.164354 | SMAGP | small cell adhesion glycoprotein |
| 16844558 | -4.74 | 0.018632 | 0.273002 | KRTAP1-5 | keratin associated protein 1-5 |
| 16859795 | -4.75 | 0.0028 | 0.126143 | GDF15 | growth differentiation factor 15 |
| 17059355 | -4.78 | 0.043316 | 0.379719 | SEMA3D | sema domain, immunoglobulin domain (Ig), short basic domain, secreted, (semaphorin) 3D |
| 17096471 | -4.83 | 0.000023 | 0.01717 | TBC1D2 | TBC1 domain family, member 2 |
| 16852312 | -4.89 | 0.001783 | 0.103744 | SKA1 | spindle and kinetochore associated complex subunit 1 |
| 16759218 | -4.92 | 0.00305 | 0.130327 | GPR133 | G protein-coupled receptor 133; NULL |
| 16775763 | -4.96 | 0.001691 | 0.10019 | MIR622 | microRNA 622 |
| 16970404 | -5.01 | 0.000336 | 0.048518 | FGF2 | fibroblast growth factor 2 (basic) |
| 16687123 | -5.06 | 0.00006 | 0.024259 | RAB3B | RAB3B, member RAS oncogene family |
| 16774427 | -5.2 | 0.000439 | 0.052725 | LACC1; CCDC122 | laccase (multicopper oxidoreductase) domain containing 1; coiled-coil domain containing 122 |
| 16784760 | -5.21 | 0.000068 | 0.025539 | DACT1 | dishevelled-binding antagonist of beta-catenin 1 |
| 17087413 | -5.24 | 0.005206 | 0.162365 | GALNT12 | UDP-N-acetyl-alpha-D-galactosamine:polypeptide N-acetylgalactosaminyltransferase 12 (GalNAc-T12) |
| 17110401 | -5.24 | 0.003136 | 0.131871 | SLC9A7 | solute carrier family 9, subfamily A (NHE7, cation proton antiporter 7), member 7 |
| 16984730 | -5.29 | 0.000846 | 0.072981 | FST | follistatin |
| 16675558 | -5.34 | 0.000151 | 0.036125 | NEK7 | NIMA-related kinase 7; NULL |
| 17017018 | -5.36 | 0.000014 | 0.014085 | IER3 | immediate early response 3; NULL |
| 17077826 | -5.37 | 0.005019 | 0.160736 | MYBL1; LOC645895 | v-myb myeloblastosis viral oncogene homolog (avian)-like 1; uncharacterized LOC645895 |
| 16914972 | -5.48 | 0.000028 | 0.019043 | DOK5 | docking protein 5 |
| 16738630 | -5.69 | 0.004656 | 0.155542 | LPXN | leupaxin |
| 16779546 | -5.79 | 0.000148 | 0.035982 | DIAPH3 | diaphanous homolog 3 (Drosophila) |
| 17050765 | -5.94 | 0.005904 | 0.171879 | KCND2 | potassium voltage-gated channel, Shal-related subfamily, member 2 |
| 16701037 | -6.01 | 0.000744 | 0.067469 | GREM2 | gremlin 2, DAN family BMP antagonist |
| 16749423 | -6.12 | 0.002989 | 0.128943 | ARNTL2 | aryl hydrocarbon receptor nuclear translocator-like 2 |
| 16767422 | -6.2 | 0.000175 | 0.038908 | PTPRB | protein tyrosine phosphatase, receptor type, B; NULL |
| 16743721 | -6.25 | 0.00034 | 0.048586 | MMP1 | matrix metallopeptidase 1 (interstitial collagenase) |
| 16855545 | -6.33 | 0.000224 | 0.041323 | ALPK2 | alpha-kinase 2 |
| 16859314 | -6.49 | 0.000046 | 0.021075 | KLF2 | Kruppel-like factor 2 (lung) |
| 16960355 | -6.61 | 0.019561 | 0.279336 | TM4SF1 | transmembrane 4 L six family member 1 |
| 17013851 | -6.85 | 1.47E-07 | 0.003227 | MYCT1 | myc target 1 |
| 17072135 | -7.04 | 0.000634 | 0.062021 | NOV | nephroblastoma overexpressed |
| 16950440 | -7.22 | 0.001265 | 0.086384 | OXTR | oxytocin receptor |
| 16803754 | -7.24 | 0.000406 | 0.051888 | KIAA1199 | KIAA1199; NULL |
| 17084130 | -7.37 | 1.36E-07 | 0.003227 | TEK | TEK tyrosine kinase, endothelial |
| 17056426 | -8 | 0.00061 | 0.061035 | PDE1C | phosphodiesterase 1C, calmodulin-dependent 70kDa |
| 16967631 | -8 | 0.000035 | 0.020495 | SLC4A4 | solute carrier family 4, sodium bicarbonate cotransporter, member 4 |
| 17115996 | -8.11 | 0.000239 | 0.041932 | KRT18P10 | NULL; keratin 18 pseudogene 10 |
| 16904667 | -8.28 | 0.003494 | 0.137508 | SCN9A | sodium channel, voltage-gated, type IX, alpha subunit; NULL |
| 16785127 | -8.29 | 0.000014 | 0.014085 | RHOJ | ras homolog family member J |
| 16691327 | -8.68 | 9.13E-07 | 0.006291 | NGF | nerve growth factor (beta polypeptide) |
| 17050797 | -8.9 | 0.000018 | 0.01575 | CPED1 | cadherin-like and PC-esterase domain containing 1 |
| 16996146 | -9 | 0.00608 | 0.174056 | ESM1 | endothelial cell-specific molecule 1 |
| 17005138 | -9.05 | 0.000023 | 0.01717 | CAP2 | CAP, adenylate cyclase-associated protein, 2 (yeast) |
| 16716371 | -11.61 | 0.000006 | 0.011074 | CH25H | cholesterol 25-hydroxylase |
| 16844585 | -13.43 | 0.004358 | 0.150581 | KRTAP2-3; KRTAP2-4 | keratin associated protein 2-3; keratin associated protein 2-4 |
| 17044177 | -13.75 | 0.000187 | 0.039211 | IL6 | interleukin 6 (interferon, beta 2); NULL |
| 16855510 | -14.17 | 0.000007 | 0.011546 | ATP8B1 | ATPase, aminophospholipid transporter, class I, type 8B, member 1 |
| 17080486 | -15.1 | 0.000004 | 0.010118 | TNFRSF11B | tumor necrosis factor receptor superfamily, member 11b |
| 16844663 | -15.65 | 0.000333 | 0.048472 | KRT34 | keratin 34 |
| 16697471 | -19.75 | 0.000009 | 0.011546 | B3GALT2 | UDP-Gal:betaGlcNAc beta 1,3-galactosyltransferase, polypeptide 2 |
