# Supplementary material for: Identification of transcription factors potentially involved in human adipogenesis in vitro
Source: Mol Genet Genomic Med. 2017 Mar 3;5(3):210–22. doi: 10.1002/mgg3.269 (PMC5441431; doi:10.1002/mgg3.269)
Supplement: Supplementary file 2 — Table S2. A complete list of differentially expressed genes on day 7. [file MGG3-5-210-s002.doc]

|  |  |  |  |  |  |
| --- | --- | --- | --- | --- | --- |
| **Up-regulated genes on day 7** | |  |  |  |  |
| Transcript Cluster ID | Fold Change (linear) (Induced vs. Control) | ANOVA p-value (Induced vs. Control) | FDR p-value (Induced vs. Control) | Gene Symbol | Description |
| 17078592 | 268.78 | 0.000004 | 0.011756 | FABP4 | fatty acid binding protein 4, adipocyte; NULL |
| 17059955 | 157.69 | 9.62E-07 | 0.005199 | PDK4 | pyruvate dehydrogenase kinase, isozyme 4 |
| 17021510 | 137.28 | 0.000001 | 0.006004 | CNR1 | cannabinoid receptor 1 (brain); NULL |
| 16949397 | 98.98 | 0.00007 | 0.030482 | ADIPOQ | adiponectin, C1Q and collagen domain containing |
| 16779958 | 95.1 | 5.62E-07 | 0.005199 | EDNRB | endothelin receptor type B |
| 16774303 | 57.28 | 0.000056 | 0.027975 | RGCC | regulator of cell cycle |
| 16676988 | 51.31 | 0.000017 | 0.020308 | HSD11B1 | hydroxysteroid (11-beta) dehydrogenase 1 |
| 16763182 | 40.54 | 3.32E-07 | 0.005199 | ABCD2 | ATP-binding cassette, sub-family D (ALD), member 2 |
| 17102829 | 38.48 | 0.000001 | 0.006004 | MAOA | monoamine oxidase A |
| 16767335 | 36.22 | 0.000018 | 0.020308 | CPM | carboxypeptidase M; NULL |
| 16813173 | 35.71 | 7.42E-07 | 0.005199 | PLIN1 | perilipin 1 |
| 16729472 | 32.01 | 0.004052 | 0.137903 | THRSP | thyroid hormone responsive |
| 16950609 | 29.67 | 9.00E-07 | 0.005199 | CIDEC | cell death-inducing DFFA-like effector c |
| 16938133 | 26.07 | 0.000259 | 0.05248 | GALNT15 | UDP-N-acetyl-alpha-D-galactosamine:polypeptide N-acetylgalactosaminyltransferase 15 |
| 16978236 | 25.73 | 0.013623 | 0.232397 | ADH1B | alcohol dehydrogenase 1B (class I), beta polypeptide |
| 17075589 | 22.46 | 0.000438 | 0.060845 | NEFL | neurofilament, light polypeptide |
| 16883690 | 21.83 | 0.000117 | 0.038889 | IL1RL1 | interleukin 1 receptor-like 1; NULL |
| 16761938 | 20.9 | 0.000003 | 0.009894 | LMO3 | LIM domain only 3 (rhombotin-like 2); NULL |
| 16834516 | 20.61 | 0.000143 | 0.040991 | AOC3 | amine oxidase, copper containing 3 |
| 16821541 | 20.32 | 0.000277 | 0.053107 | CRISPLD2 | cysteine-rich secretory protein LCCL domain containing 2 |
| 16675158 | 19.65 | 0.044114 | 0.365318 | PRG4 | proteoglycan 4; NULL |
| 16722299 | 19.13 | 0.003491 | 0.129909 | PDE3B | phosphodiesterase 3B, cGMP-inhibited; NULL |
| 17113346 | 18.9 | 0.002893 | 0.120934 | CHRDL1 | chordin-like 1; NULL |
| 16756649 | 18.37 | 0.00007 | 0.030482 | ACACB | acetyl-CoA carboxylase beta; NULL |
| 17066278 | 18.26 | 0.00169 | 0.095021 | LPL | lipoprotein lipase; NULL |
| 16977378 | 17.82 | 0.000029 | 0.021308 | TMEM150C | transmembrane protein 150C |
| 16769250 | 17.67 | 0.00518 | 0.155117 | IGF1 | insulin-like growth factor 1 (somatomedin C) |
| 16850958 | 17.56 | 0.000128 | 0.039172 | APCDD1 | adenomatosis polyposis coli down-regulated 1 |
| 16751190 | 17.01 | 0.000679 | 0.067528 | METTL7A | methyltransferase like 7A; NULL |
| 17061759 | 16.65 | 0.000293 | 0.054388 | NRCAM | neuronal cell adhesion molecule; NULL |
| 16819224 | 15.7 | 0.000133 | 0.039996 | MT1M | metallothionein 1M |
| 16819264 | 14.5 | 0.000059 | 0.02921 | MT1X | metallothionein 1X |
| 16667530 | 13.75 | 0.00066 | 0.066659 | PALMD | palmdelphin |
| 16963113 | 12.33 | 0.002751 | 0.118638 | APOD | apolipoprotein D; NULL |
| 17050154 | 12.13 | 0.001768 | 0.095788 | PRKAR2B | protein kinase, cAMP-dependent, regulatory, type II, beta |
| 16970435 | 11.4 | 0.026939 | 0.300549 | SPRY1 | sprouty homolog 1, antagonist of FGF signaling (Drosophila); NULL |
| 16751048 | 11.18 | 0.00073 | 0.068691 | GPD1 | glycerol-3-phosphate dehydrogenase 1 (soluble); NULL |
| 16711343 | 11.12 | 0.000012 | 0.018322 | AKR1C2; LOC101060798 | aldo-keto reductase family 1, member C2; aldo-keto reductase family 1 member C2-like; NULL |
| 16716659 | 10.05 | 0.000484 | 0.061367 | RBP4 | retinol binding protein 4, plasma |
| 16722278 | 9.96 | 0.004799 | 0.148851 | SPON1 | spondin 1, extracellular matrix protein |
| 17063005 | 9.42 | 0.000118 | 0.038905 | PLXNA4 | plexin A4 |
| 16841907 | 9.16 | 0.000448 | 0.060845 | RASD1 | RAS, dexamethasone-induced 1 |
| 16726065 | 8.74 | 0.018126 | 0.25932 | LGALS12 | lectin, galactoside-binding, soluble, 12 |
| 16919022 | 8.59 | 0.000184 | 0.045852 | SAMHD1 | SAM domain and HD domain 1 |
| 16991192 | 8.39 | 0.000198 | 0.046364 | GPX3 | glutathione peroxidase 3 (plasma); NULL |
| 16950825 | 8.06 | 0.000219 | 0.047999 | TIMP4 | TIMP metallopeptidase inhibitor 4 |
| 16790744 | 7.86 | 0.005583 | 0.160234 | SLC7A8 | solute carrier family 7 (amino acid transporter light chain, L system), member 8; NULL |
| 16771602 | 7.84 | 0.000199 | 0.046364 | HPD | 4-hydroxyphenylpyruvate dioxygenase |
| 16982047 | 7.67 | 0.000045 | 0.026559 | ACSL1 | acyl-CoA synthetase long-chain family member 1; NULL |
| 17001846 | 7.24 | 0.000121 | 0.038923 | CCDC69 | coiled-coil domain containing 69; NULL |
| 16704320 | 7.21 | 0.002485 | 0.112211 | RASSF4 | Ras association (RalGDS/AF-6) domain family member 4; NULL |
| 16851309 | 7.17 | 0.000061 | 0.029261 | GREB1L | growth regulation by estrogen in breast cancer-like; NULL |
| 16955822 | 7.02 | 0.001022 | 0.077385 | ADAMTS9 | ADAM metallopeptidase with thrombospondin type 1 motif, 9; NULL |
| 16695627 | 6.81 | 0.008271 | 0.188161 | ADAMTS4 | ADAM metallopeptidase with thrombospondin type 1 motif, 4 |
| 17018497 | 6.77 | 0.000014 | 0.020098 | FKBP5; LOC285847 | FK506 binding protein 5; uncharacterized LOC285847 |
| 16778392 | 6.67 | 0.000047 | 0.026571 | FOXO1 | forkhead box O1 |
| 16700888 | 6.62 | 0.000083 | 0.032987 | NID1 | nidogen 1 |
| 16671187 | 6.58 | 0.000975 | 0.076144 | NPR1 | natriuretic peptide receptor A/guanylate cyclase A (atrionatriuretic peptide receptor A) |
| 17016089 | 6.52 | 0.018404 | 0.260661 | PRL | prolactin |
| 16967875 | 6.5 | 0.009387 | 0.198588 | PARM1 | prostate androgen-regulated mucin-like protein 1 |
| 17080082 | 6.36 | 0.000573 | 0.064876 | ANGPT1 | angiopoietin 1 |
| 16846864 | 6.34 | 0.001907 | 0.099509 | MMD | monocyte to macrophage differentiation-associated |
| 17114288 | 6.33 | 0.000416 | 0.060761 | GPC3 | glypican 3 |
| 16785379 | 6.18 | 0.005199 | 0.155149 | HSPA2 | heat shock 70kDa protein 2 |
| 16696979 | 6.17 | 0.000039 | 0.026476 | GLUL | glutamate-ammonia ligase; NULL |
| 16998551 | 6.13 | 0.004376 | 0.143559 | SLCO4C1 | solute carrier organic anion transporter family, member 4C1 |
| 16981099 | 6.06 | 0.004522 | 0.146279 | NPY1R | neuropeptide Y receptor Y1; NULL |
| 16883715 | 6.05 | 0.000658 | 0.066659 | IL18R1 | interleukin 18 receptor 1 |
| 16836021 | 5.91 | 0.008893 | 0.194288 | ABCC3 | ATP-binding cassette, sub-family C (CFTR/MRP), member 3; NULL |
| 16709268 | 5.83 | 0.005602 | 0.160234 | ACSL5 | acyl-CoA synthetase long-chain family member 5; NULL |
| 16952782 | 5.76 | 0.00556 | 0.160234 | TMEM158 | transmembrane protein 158 (gene/pseudogene) |
| 17022623 | 5.75 | 0.000923 | 0.075552 | REV3L | REV3-like, polymerase (DNA directed), zeta, catalytic subunit; NULL |
| 16708192 | 5.63 | 0.001529 | 0.09101 | ABCC2 | ATP-binding cassette, sub-family C (CFTR/MRP), member 2 |
| 16761820 | 5.58 | 0.000659 | 0.066659 | MGP | matrix Gla protein |
| 16928204 | 5.56 | 0.000725 | 0.068691 | POM121L9P; LOC727983 | POM121 transmembrane nucleoporin-like 9, pseudogene; putative POM121-like protein 1-like |
| 16718414 | 5.48 | 0.007482 | 0.179891 | GPAM | glycerol-3-phosphate acyltransferase, mitochondrial; NULL |
| 16832429 | 5.47 | 0.000135 | 0.039996 | TMEM97 | transmembrane protein 97 |
| 17025294 | 5.46 | 0.000692 | 0.067528 | LOC100129518; SOD2 | uncharacterized LOC100129518; superoxide dismutase 2, mitochondrial |
| 16879094 | 5.46 | 0.025709 | 0.296049 | VIT | vitrin |
| 17048072 | 5.45 | 0.000074 | 0.030971 | STEAP1 | six transmembrane epithelial antigen of the prostate 1 |
| 16696177 | 5.34 | 0.001723 | 0.095243 | SLC19A2 | solute carrier family 19 (thiamine transporter), member 2 |
| 16958638 | 5.19 | 0.000184 | 0.045852 | KLF15 | Kruppel-like factor 15 |
| 16955197 | 5.17 | 0.003768 | 0.13411 | WNT5A | wingless-type MMTV integration site family, member 5A |
| 16696120 | 5.14 | 0.000814 | 0.07176 | DPT | dermatopontin |
| 16850107 | 5.07 | 0.000213 | 0.047666 | FASN | fatty acid synthase |
| 16731441 | 5.03 | 0.000007 | 0.015346 | ZBTB16 | zinc finger and BTB domain containing 16; NULL |
| 16706630 | 4.93 | 0.000204 | 0.046619 | FAM213A | family with sequence similarity 213, member A |
| 16676983 | 4.93 | 0.001446 | 0.090054 | G0S2 | G0/G1switch 2 |
| 16819233 | 4.93 | 0.006805 | 0.17365 | MT1A | metallothionein 1A |
| 16999041 | 4.85 | 0.006338 | 0.1674 | CDO1 | cysteine dioxygenase type 1 |
| 16702935 | 4.74 | 0.001345 | 0.08697 | CACNB2 | calcium channel, voltage-dependent, beta 2 subunit; NULL |
| 17047795 | 4.73 | 0.023166 | 0.285114 | CD36 | CD36 molecule (thrombospondin receptor); NULL |
| 16837348 | 4.73 | 0.00367 | 0.133414 | MAP2K6 | mitogen-activated protein kinase kinase 6 |
| 16985518 | 4.73 | 0.000534 | 0.063691 | PIK3R1 | phosphoinositide-3-kinase, regulatory subunit 1 (alpha); NULL |
| 16933140 | 4.7 | 0.004993 | 0.151839 | GGT5 | gamma-glutamyltransferase 5 |
| 16819252 | 4.7 | 0.007237 | 0.177002 | MT1F | metallothionein 1F; NULL |
| 16919962 | 4.66 | 0.000123 | 0.038976 | SULF2 | sulfatase 2; NULL |
| 16696811 | 4.64 | 0.003069 | 0.124042 | ANGPTL1 | angiopoietin-like 1 |
| 16826639 | 4.58 | 0.000271 | 0.052967 | CES1; LOC100653057 | carboxylesterase 1; liver carboxylesterase 1-like; NULL |
| 16938630 | 4.57 | 0.000006 | 0.01431 | GPD1L | glycerol-3-phosphate dehydrogenase 1-like; NULL |
| 16867326 | 4.56 | 0.001091 | 0.079724 | PLIN4 | perilipin 4 |
| 16733516 | 4.54 | 0.033717 | 0.330139 | ADAMTS15 | ADAM metallopeptidase with thrombospondin type 1 motif, 15 |
| 17095056 | 4.54 | 0.017324 | 0.254568 | PRUNE2 | prune homolog 2 (Drosophila); NULL |
| 16857886 | 4.53 | 0.012475 | 0.224742 | ANGPTL4 | angiopoietin-like 4; NULL |
| 17110071 | 4.51 | 0.002463 | 0.111864 | SRPX | sushi-repeat containing protein, X-linked |
| 16966733 | 4.44 | 0.003498 | 0.129909 | RASL11B | RAS-like, family 11, member B |
| 16912975 | 4.41 | 0.000048 | 0.026571 | ACSS2 | acyl-CoA synthetase short-chain family member 2; NULL |
| 17113147 | 4.38 | 0.00012 | 0.038923 | TSC22D3 | TSC22 domain family, member 3; NULL |
| 16840846 | 4.34 | 0.000458 | 0.060845 | PER1 | period circadian clock 1 |
| 16739733 | 4.33 | 0.049664 | 0.37801 | HRASLS5 | HRAS-like suppressor family, member 5 |
| 17076609 | 4.31 | 0.000736 | 0.068863 | SFRP1 | secreted frizzled-related protein 1 |
| 17102951 | 4.3 | 0.005682 | 0.1609 | CHST7 | carbohydrate (N-acetylglucosamine 6-O) sulfotransferase 7 |
| 16997393 | 4.27 | 0.001231 | 0.082663 | ZBED3 | zinc finger, BED-type containing 3 |
| 16769761 | 4.26 | 0.000136 | 0.040128 | TMEM119 | transmembrane protein 119 |
| 16909257 | 4.2 | 0.00045 | 0.060845 | SLC19A3 | solute carrier family 19, member 3; NULL |
| 16666485 | 4.19 | 0.000156 | 0.042948 | IFI44L | interferon-induced protein 44-like; NULL |
| 16695741 | 4.19 | 0.000363 | 0.059473 | OLFML2B | olfactomedin-like 2B |
| 16687618 | 4.12 | 0.000302 | 0.054748 | DHCR24 | 24-dehydrocholesterol reductase |
| 17014309 | 4.09 | 0.000056 | 0.027975 | ACAT2; LOC100129518; SOD2 | acetyl-CoA acetyltransferase 2; uncharacterized LOC100129518; superoxide dismutase 2, mitochondrial |
| 16748989 | 4.08 | 0.005139 | 0.154518 | PDE3A | phosphodiesterase 3A, cGMP-inhibited |
| 16834436 | 4.06 | 0.000537 | 0.063691 | RAMP2 | receptor (G protein-coupled) activity modifying protein 2 |
| 16906175 | 4.04 | 0.002319 | 0.109744 | FRZB | frizzled-related protein |
| 16889268 | 4.01 | 0.00098 | 0.076198 | AOX1 | aldehyde oxidase 1; NULL |
| 16896561 | 3.99 | 0.009258 | 0.197396 | CYP1B1 | cytochrome P450, family 1, subfamily B, polypeptide 1 |
| 16668564 | 3.97 | 0.037086 | 0.342685 | PROK1 | prokineticin 1 |
| 16871235 | 3.96 | 0.000417 | 0.060761 | CEBPA | CCAAT/enhancer binding protein (C/EBP), alpha |
| 16906346 | 3.96 | 0.004109 | 0.138785 | DIRC1 | disrupted in renal carcinoma 1 |
| 17095703 | 3.92 | 0.001151 | 0.080942 | NFIL3 | nuclear factor, interleukin 3 regulated |
| 16974830 | 3.89 | 0.002126 | 0.105805 | PPARGC1A | peroxisome proliferator-activated receptor gamma, coactivator 1 alpha; NULL |
| 17093227 | 3.87 | 0.006645 | 0.172073 | AQP7; LOC100509620 | aquaporin 7; aquaporin-7-like; NULL |
| 17061662 | 3.85 | 0.00007 | 0.030482 | LAMB1 | laminin, beta 1 |
| 17105332 | 3.85 | 0.015216 | 0.242084 | SRPX2 | sushi-repeat containing protein, X-linked 2 |
| 16876777 | 3.84 | 0.00053 | 0.063691 | RNF144A | ring finger protein 144A; NULL |
| 16906285 | 3.82 | 0.001484 | 0.09042 | CALCRL | calcitonin receptor-like; NULL |
| 16763577 | 3.8 | 0.001412 | 0.089143 | SLC38A4 | solute carrier family 38, member 4 |
| 16953597 | 3.77 | 0.000091 | 0.03502 | SLC26A6 | solute carrier family 26, member 6; NULL |
| 17092712 | 3.76 | 0.000396 | 0.060302 | PLIN2; LOC100509484 | perilipin 2; uncharacterized LOC100509484 |
| 17102129 | 3.76 | 0.000001 | 0.005199 | SAT1 | spermidine/spermine N1-acetyltransferase 1; NULL |
| 16872783 | 3.75 | 0.00803 | 0.185387 | LIPE | lipase, hormone-sensitive |
| 16773493 | 3.75 | 0.000883 | 0.074487 | RASL11A | RAS-like, family 11, member A |
| 16716795 | 3.73 | 0.000406 | 0.060588 | SORBS1; KIAA0894; RP11-476E15.3 | sorbin and SH3 domain containing 1; NULL |
| 16780917 | 3.68 | 0.000196 | 0.046364 | IRS2 | insulin receptor substrate 2 |
| 17012447 | 3.68 | 0.001209 | 0.08234 | LAMA2 | laminin, alpha 2 |
| 16701975 | 3.65 | 0.000455 | 0.060845 | AKR1C1 | aldo-keto reductase family 1, member C1; NULL |
| 16836311 | 3.62 | 0.00377 | 0.13411 | NOG | noggin |
| 16856299 | 3.6 | 0.005646 | 0.160345 | CFD | complement factor D (adipsin) |
| 16937741 | 3.6 | 0.001375 | 0.087738 | PPARG | peroxisome proliferator-activated receptor gamma; NULL |
| 16707107 | 3.56 | 0.018911 | 0.262723 | LIPM | lipase, family member M |
| 16819213 | 3.54 | 0.006823 | 0.17365 | MT1L; NUTF2 | metallothionein 1L (gene/pseudogene); nuclear transport factor 2 |
| 16970465 | 3.51 | 0.001145 | 0.080942 | FAT4 | FAT atypical cadherin 4 |
| 16779667 | 3.48 | 0.002456 | 0.111748 | PCDH9 | protocadherin 9 |
| 16947173 | 3.46 | 0.000196 | 0.046364 | MME | membrane metallo-endopeptidase; NULL |
| 16773453 | 3.43 | 0.000456 | 0.060845 | WASF3 | WAS protein family, member 3 |
| 16708249 | 3.41 | 0.000015 | 0.020098 | SCD | stearoyl-CoA desaturase (delta-9-desaturase) |
| 16666965 | 3.4 | 0.001247 | 0.083497 | LRRC8B | leucine rich repeat containing 8 family, member B |
| 16826738 | 3.4 | 0.002489 | 0.112272 | MT1G | metallothionein 1G |
| 16995890 | 3.37 | 0.000199 | 0.046364 | HMGCS1 | 3-hydroxy-3-methylglutaryl-CoA synthase 1 (soluble); NULL |
| 17087790 | 3.37 | 0.000464 | 0.060845 | SLC44A1 | solute carrier family 44, member 1 |
| 16689546 | 3.35 | 0.025747 | 0.296125 | TGFBR3 | transforming growth factor, beta receptor III; NULL |
| 16760668 | 3.33 | 0.000166 | 0.043542 | LPCAT3 | lysophosphatidylcholine acyltransferase 3; NULL |
| 17063461 | 3.32 | 0.00695 | 0.17461 | HIPK2 | homeodomain interacting protein kinase 2 |
| 16723546 | 3.31 | 0.003693 | 0.133733 | CAT | catalase |
| 17019805 | 3.31 | 0.025194 | 0.293689 | TNFRSF21 | tumor necrosis factor receptor superfamily, member 21 |
| 16936947 | 3.3 | 0.022573 | 0.281319 | ITPR1 | inositol 1,4,5-trisphosphate receptor, type 1; NULL |
| 16699877 | 3.3 | 0.000827 | 0.071982 | LBR | lamin B receptor; NULL |
| 16739132 | 3.28 | 0.000041 | 0.026559 | FADS1; MIR1908 | fatty acid desaturase 1; microRNA 1908; NULL |
| 16942270 | 3.28 | 0.000254 | 0.052003 | PTPRG | protein tyrosine phosphatase, receptor type, G; NULL |
| 16749782 | 3.25 | 0.001128 | 0.080595 | FGD4 | FYVE, RhoGEF and PH domain containing 4; NULL |
| 16911632 | 3.25 | 0.017743 | 0.25732 | PCSK2 | proprotein convertase subtilisin/kexin type 2 |
| 16883647 | 3.23 | 0.000667 | 0.066994 | IL1R1 | interleukin 1 receptor, type I; NULL |
| 17071298 | 3.23 | 0.048863 | 0.375829 | OSR2 | odd-skipped related 2 (Drosophila); NULL |
| 16713318 | 3.21 | 0.021197 | 0.274101 | NAMPT; NAMPTL | nicotinamide phosphoribosyltransferase; nicotinamide phosphoribosyltransferase-like |
| 16859840 | 3.2 | 0.006422 | 0.168319 | TMEM59L; SYNGR1 | transmembrane protein 59-like; synaptogyrin 1; NULL |
| 16999475 | 3.19 | 0.014914 | 0.240982 | FBN2 | fibrillin 2 |
| 17012859 | 3.19 | 0.019553 | 0.266451 | PDE7B | phosphodiesterase 7B |
| 16986249 | 3.18 | 0.000546 | 0.063762 | HMGCR | 3-hydroxy-3-methylglutaryl-CoA reductase; NULL |
| 16966127 | 3.13 | 0.011563 | 0.218079 | KLB | klotho beta |
| 16658864 | 3.13 | 0.000429 | 0.060845 | PGD | phosphogluconate dehydrogenase; NULL |
| 16975671 | 3.09 | 0.029771 | 0.314383 | CORIN | corin, serine peptidase; NULL |
| 16743091 | 3.09 | 0.00018 | 0.045341 | FZD4 | frizzled family receptor 4 |
| 16978995 | 3.07 | 0.00012 | 0.038923 | ELOVL6 | ELOVL fatty acid elongase 6; NULL |
| 16729290 | 3.07 | 0.007873 | 0.183792 | TSKU | tsukushi, small leucine rich proteoglycan |
| 16850069 | 3.05 | 0.000696 | 0.067528 | DCXR | dicarbonyl/L-xylulose reductase; NULL |
| 16819217 | 3.04 | 0.003443 | 0.129436 | MT1E | metallothionein 1E |
| 17023150 | 3.03 | 0.000933 | 0.075552 | MAN1A1 | mannosidase, alpha, class 1A, member 1 |
| 16967794 | 3.02 | 0.030779 | 0.318052 | CXCL1 | chemokine (C-X-C motif) ligand 1 (melanoma growth stimulating activity, alpha) |
| 16870443 | 3 | 0.006404 | 0.168128 | CRLF1 | cytokine receptor-like factor 1 |
| 17048083 | 2.99 | 0.000688 | 0.067528 | STEAP2 | STEAP family member 2, metalloreductase; NULL |
| 16678114 | 2.98 | 0.001147 | 0.080942 | EPHX1 | epoxide hydrolase 1, microsomal (xenobiotic) |
| 16762146 | 2.98 | 0.001789 | 0.096243 | KCNJ8 | potassium inwardly-rectifying channel, subfamily J, member 8; NULL |
| 17048102 | 2.97 | 0.0079 | 0.184026 | C7orf63 | chromosome 7 open reading frame 63; NULL |
| 17075789 | 2.97 | 0.015686 | 0.244761 | SCARA5 | scavenger receptor class A, member 5 (putative) |
| 16693082 | 2.97 | 0.003124 | 0.124678 | SELENBP1 | selenium binding protein 1; NULL |
| 16743432 | 2.97 | 0.037509 | 0.343443 | SESN3 | sestrin 3 |
| 16739435 | 2.95 | 0.005747 | 0.161511 | C11orf48 | chromosome 11 open reading frame 48 |
| 16998342 | 2.95 | 0.005171 | 0.154996 | PCSK1 | proprotein convertase subtilisin/kexin type 1 |
| 16966721 | 2.95 | 0.000106 | 0.038079 | SNORA26 | small nucleolar RNA, H/ACA box 26 |
| 16743111 | 2.94 | 0.002154 | 0.106469 | CTSC | cathepsin C; NULL |
| 17075973 | 2.94 | 0.014892 | 0.240982 | DUSP4 | dual specificity phosphatase 4 |
| 16668397 | 2.93 | 0.001863 | 0.097982 | GSTM5 | glutathione S-transferase mu 5; NULL |
| 16906733 | 2.93 | 0.001151 | 0.080942 | STK17B | serine/threonine kinase 17b |
| 16913537 | 2.92 | 0.000482 | 0.061367 | LBP | lipopolysaccharide binding protein |
| 16762759 | 2.91 | 0.037246 | 0.342912 | TMTC1 | transmembrane and tetratricopeptide repeat containing 1 |
| 16914062 | 2.91 | 0.000189 | 0.046364 | TTPAL | tocopherol (alpha) transfer protein-like |
| 16962584 | 2.9 | 0.000144 | 0.040991 | BCL6 | B-cell CLL/lymphoma 6; NULL |
| 17048563 | 2.87 | 0.004405 | 0.143761 | PEG10 | paternally expressed 10 |
| 16947148 | 2.86 | 0.000115 | 0.038672 | ARHGEF26 | Rho guanine nucleotide exchange factor (GEF) 26 |
| 16763138 | 2.86 | 0.019508 | 0.265988 | KIF21A | kinesin family member 21A |
| 16842103 | 2.86 | 0.006866 | 0.173713 | SHMT1 | serine hydroxymethyltransferase 1 (soluble); NULL |
| 16830883 | 2.85 | 0.003082 | 0.124129 | ALOX15B | arachidonate 15-lipoxygenase, type B |
| 16753670 | 2.83 | 0.000544 | 0.063691 | IRAK3 | interleukin-1 receptor-associated kinase 3 |
| 17024144 | 2.81 | 0.000044 | 0.026559 | IFNGR1 | interferon gamma receptor 1; NULL |
| 16868481 | 2.81 | 0.045105 | 0.367442 | OLFM2 | olfactomedin 2; NULL |
| 16730104 | 2.81 | 0.000006 | 0.013984 | SCARNA9; SCARNA9L | small Cajal body-specific RNA 9; small Cajal body-specific RNA 9-like |
| 16967863 | 2.8 | 0.007395 | 0.179276 | AREG; AREGB | amphiregulin; amphiregulin B |
| 16851565 | 2.79 | 0.002302 | 0.109466 | TTC39C | tetratricopeptide repeat domain 39C |
| 17096728 | 2.78 | 0.008657 | 0.191979 | ABCA1 | ATP-binding cassette, sub-family A (ABC1), member 1 |
| 16909165 | 2.78 | 0.000274 | 0.053107 | IRS1 | insulin receptor substrate 1 |
| 16766822 | 2.78 | 0.03057 | 0.31705 | LRIG3 | leucine-rich repeats and immunoglobulin-like domains 3 |
| 17113744 | 2.77 | 0.000109 | 0.038495 | CUL4B | cullin 4B |
| 16984304 | 2.75 | 0.007626 | 0.181343 | C7 | complement component 7; NULL |
| 17059828 | 2.75 | 0.017439 | 0.255088 | TFPI2 | tissue factor pathway inhibitor 2 |
| 16834091 | 2.74 | 0.000293 | 0.054388 | IGFBP4 | insulin-like growth factor binding protein 4 |
| 16991180 | 2.74 | 0.000945 | 0.075552 | SMIM3 | small integral membrane protein 3 |
| 16955800 | 2.73 | 0.033856 | 0.330645 | PRICKLE2 | prickle homolog 2 (Drosophila) |
| 16855973 | 2.69 | 0.001103 | 0.080018 | CYB5A | cytochrome b5 type A (microsomal) |
| 17088462 | 2.69 | 0.002478 | 0.112211 | PAPPA | pregnancy-associated plasma protein A, pappalysin 1 |
| 17109272 | 2.69 | 0.004402 | 0.143761 | PIR-FIGF; PIR; FIGF | PIR-FIGF readthrough; pirin (iron-binding nuclear protein); c-fos induced growth factor (vascular endothelial growth factor D); NULL |
| 17113774 | 2.68 | 0.000083 | 0.032987 | C1GALT1C1 | C1GALT1-specific chaperone 1 |
| 16716782 | 2.68 | 0.002378 | 0.110174 | PDLIM1 | PDZ and LIM domain 1 |
| 16763195 | 2.68 | 0.004103 | 0.138785 | SLC2A13 | solute carrier family 2 (facilitated glucose transporter), member 13 |
| 17093463 | 2.67 | 0.004766 | 0.148084 | CNTFR | ciliary neurotrophic factor receptor |
| 16800980 | 2.67 | 0.004389 | 0.1437 | GABPB1-AS1; GABPB1 | GABPB1 antisense RNA 1; GA binding protein transcription factor, beta subunit 1; NULL |
| 16722562 | 2.67 | 0.003857 | 0.135273 | SAA1 | serum amyloid A1 |
| 16855305 | 2.65 | 0.003966 | 0.136606 | MRO | maestro; NULL |
| 16675323 | 2.64 | 0.001044 | 0.078131 | RGS2 | regulator of G-protein signaling 2, 24kDa; NULL |
| 16690566 | 2.64 | 0.023115 | 0.284889 | SORT1 | sortilin 1; NULL |
| 16966712 | 2.63 | 0.000352 | 0.059225 | DANCR | differentiation antagonizing non-protein coding RNA; NULL |
| 17076063 | 2.63 | 0.00028 | 0.053107 | GSR | glutathione reductase |
| 16779855 | 2.63 | 0.000498 | 0.061679 | MYCBP2 | MYC binding protein 2, E3 ubiquitin protein ligase |
| 16688506 | 2.62 | 0.007435 | 0.179648 | NEGR1 | neuronal growth regulator 1; NULL |
| 16796375 | 2.61 | 0.005458 | 0.158976 | CLMN | calmin (calponin-like, transmembrane); NULL |
| 16879385 | 2.61 | 0.001492 | 0.09042 | PKDCC | protein kinase domain containing, cytoplasmic; NULL |
| 16977396 | 2.61 | 0.000571 | 0.064876 | SCD5 | stearoyl-CoA desaturase 5 |
| 17020152 | 2.6 | 0.000608 | 0.06538 | ELOVL5 | ELOVL fatty acid elongase 5; NULL |
| 17059628 | 2.57 | 0.000382 | 0.059593 | CYP51A1; LRRD1 | cytochrome P450, family 51, subfamily A, polypeptide 1; leucine-rich repeats and death domain containing 1 |
| 16908897 | 2.57 | 0.002289 | 0.109389 | EPHA4 | EPH receptor A4; NULL |
| 16708179 | 2.56 | 0.007302 | 0.1783 | CUTC | cutC copper transporter homolog (E. coli); NULL |
| 17053892 | 2.56 | 0.009103 | 0.196266 | INSIG1 | insulin induced gene 1 |
| 16888963 | 2.56 | 0.008302 | 0.188543 | NABP1 | nucleic acid binding protein 1; NULL |
| 17067011 | 2.56 | 0.008994 | 0.194939 | NEFM | neurofilament, medium polypeptide |
| 17002820 | 2.56 | 0.000409 | 0.060755 | SH3PXD2B | SH3 and PX domains 2B |
| 16773165 | 2.56 | 0.005832 | 0.161724 | TNFRSF19 | tumor necrosis factor receptor superfamily, member 19 |
| 17097211 | 2.55 | 0.002555 | 0.114211 | PTGR1 | prostaglandin reductase 1 |
| 16909303 | 2.53 | 0.008045 | 0.185442 | PID1 | phosphotyrosine interaction domain containing 1 |
| 16884187 | 2.53 | 0.004602 | 0.147124 | SH3RF3 | SH3 domain containing ring finger 3 |
| 16997399 | 2.53 | 0.025872 | 0.296629 | SNORA47 | small nucleolar RNA, H/ACA box 47 |
| 16729168 | 2.52 | 0.000026 | 0.020308 | DGAT2 | diacylglycerol O-acyltransferase 2 |
| 16843049 | 2.5 | 0.026071 | 0.296884 | SSH2 | slingshot protein phosphatase 2 |
| 16729812 | 2.49 | 0.001045 | 0.078131 | TMEM135 | transmembrane protein 135; NULL |
| 16829139 | 2.48 | 0.000045 | 0.026559 | MVD | mevalonate (diphospho) decarboxylase; NULL |
| 17023799 | 2.48 | 0.00043 | 0.060845 | SLC2A12 | solute carrier family 2 (facilitated glucose transporter), member 12 |
| 17106688 | 2.47 | 0.0457 | 0.368809 | GRIA3 | glutamate receptor, ionotropic, AMPA 3; NULL |
| 17097132 | 2.47 | 0.000806 | 0.07176 | LPAR1 | lysophosphatidic acid receptor 1 |
| 17102512 | 2.47 | 0.005621 | 0.160277 | PRRG1 | proline rich Gla (G-carboxyglutamic acid) 1; NULL |
| 16731084 | 2.45 | 0.009682 | 0.201348 | SIK2 | salt-inducible kinase 2 |
| 16758885 | 2.44 | 0.001279 | 0.084751 | AACS | acetoacetyl-CoA synthetase |
| 16725783 | 2.44 | 0.000471 | 0.061199 | BEST1 | bestrophin 1 |
| 16748788 | 2.44 | 0.000607 | 0.06538 | MGST1 | microsomal glutathione S-transferase 1; NULL |
| 16806564 | 2.44 | 0.000283 | 0.053571 | MTMR10 | myotubularin related protein 10; NULL |
| 16996234 | 2.43 | 0.000297 | 0.054711 | PPAP2A | phosphatidic acid phosphatase type 2A; NULL |
| 17012711 | 2.43 | 0.00374 | 0.133926 | SNORA33 | small nucleolar RNA, H/ACA box 33 |
| 17069550 | 2.42 | 0.004208 | 0.140425 | ADHFE1 | alcohol dehydrogenase, iron containing, 1; NULL |
| 16819152 | 2.42 | 0.004683 | 0.147727 | CES1; LOC100653057; LOC100653086; CES1P1 | carboxylesterase 1; liver carboxylesterase 1-like; uncharacterized LOC100653086; carboxylesterase 1 pseudogene 1; NULL |
| 17058524 | 2.42 | 0.007058 | 0.175983 | MLXIPL | MLX interacting protein-like; NULL |
| 16831306 | 2.41 | 0.005466 | 0.159078 | HS3ST3B1 | heparan sulfate (glucosamine) 3-O-sulfotransferase 3B1 |
| 16832568 | 2.41 | 0.021395 | 0.274342 | SNORD4B | small nucleolar RNA, C/D box 4B |
| 16765192 | 2.4 | 0.009124 | 0.196496 | CSAD | cysteine sulfinic acid decarboxylase; NULL |
| 16671264 | 2.4 | 0.006482 | 0.169062 | SLC27A3 | solute carrier family 27 (fatty acid transporter), member 3; NULL |
| 16851026 | 2.39 | 0.036296 | 0.339644 | IMPA2 | inositol(myo)-1(or 4)-monophosphatase 2 |
| 16849992 | 2.39 | 0.001698 | 0.095021 | PCYT2 | phosphate cytidylyltransferase 2, ethanolamine; NULL |
| 16761858 | 2.39 | 0.002132 | 0.105977 | RERG | RAS-like, estrogen-regulated, growth inhibitor; NULL |
| 16820820 | 2.39 | 0.011376 | 0.21659 | SNORD111 | small nucleolar RNA, C/D box 111 |
| 16931000 | 2.38 | 0.015074 | 0.241638 | PNPLA3 | patatin-like phospholipase domain containing 3 |
| 16688469 | 2.38 | 0.034954 | 0.334557 | PTGER3 | prostaglandin E receptor 3 (subtype EP3); NULL |
| 17014798 | 2.35 | 0.046034 | 0.369453 | SMOC2 | SPARC related modular calcium binding 2 |
| 16946707 | 2.34 | 0.013114 | 0.228726 | AGTR1 | angiotensin II receptor, type 1 |
| 16704055 | 2.34 | 0.000766 | 0.069812 | HSD17B7P2 | hydroxysteroid (17-beta) dehydrogenase 7 pseudogene 2 |
| 16688269 | 2.34 | 0.006561 | 0.170567 | SLC35D1 | solute carrier family 35 (UDP-glucuronic acid/UDP-N-acetylgalactosamine dual transporter), member D1 |
| 16863115 | 2.32 | 0.003313 | 0.127676 | APOE; HMGA1 | apolipoprotein E; high mobility group AT-hook 1 |
| 17005573 | 2.32 | 0.000027 | 0.020458 | HIST1H2BD | histone cluster 1, H2bd |
| 17049270 | 2.32 | 0.004652 | 0.147489 | PILRB | paired immunoglobin-like type 2 receptor beta; NULL |
| 16855358 | 2.31 | 0.000198 | 0.046364 | CCDC68 | coiled-coil domain containing 68 |
| 16891603 | 2.31 | 0.013509 | 0.231541 | KCNE4 | potassium voltage-gated channel, Isk-related family, member 4 |
| 16921724 | 2.31 | 0.000218 | 0.047999 | NCAM2 | neural cell adhesion molecule 2; NULL |
| 17087861 | 2.3 | 0.001559 | 0.091522 | TMEM38B | transmembrane protein 38B |
| 16968122 | 2.29 | 0.002987 | 0.122314 | FRAS1 | Fraser syndrome 1 |
| 16740914 | 2.29 | 0.015376 | 0.242423 | PC | pyruvate carboxylase; NULL |
| 16834508 | 2.28 | 0.017245 | 0.253778 | AOC2 | amine oxidase, copper containing 2 (retina-specific) |
| 16822035 | 2.28 | 0.025191 | 0.293689 | DPEP1 | dipeptidase 1 (renal); NULL |
| 16914478 | 2.28 | 0.005575 | 0.160234 | EYA2 | eyes absent homolog 2 (Drosophila) |
| 16856803 | 2.28 | 0.034861 | 0.334557 | GADD45B | growth arrest and DNA-damage-inducible, beta |
| 17021217 | 2.28 | 0.015873 | 0.245934 | ME1 | malic enzyme 1, NADP(+)-dependent, cytosolic |
| 16722720 | 2.28 | 0.002496 | 0.112272 | NAV2 | neuron navigator 2; NULL |
| 17012392 | 2.28 | 0.047232 | 0.371931 | RSPO3 | R-spondin 3 |
| 16798132 | 2.27 | 0.026943 | 0.300549 | SNORD116-1 | small nucleolar RNA, C/D box 116-1 |
| 16979502 | 2.27 | 0.002744 | 0.118638 | TMEM155 | transmembrane protein 155; NULL |
| 17058142 | 2.26 | 0.040665 | 0.35367 | ZNF117 | zinc finger protein 117 |
| 16847933 | 2.25 | 0.03497 | 0.334557 | AXIN2; OTTHUMG00000179524; CTD-2535L24.2 | axin 2; NULL |
| 16741501 | 2.25 | 0.000812 | 0.07176 | DHCR7 | 7-dehydrocholesterol reductase; NULL |
| 16768413 | 2.24 | 0.011761 | 0.219333 | DCN | decorin; NULL |
| 16819244 | 2.24 | 0.002004 | 0.102169 | MT1CP | metallothionein 1C, pseudogene; NULL |
| 16922143 | 2.22 | 0.000509 | 0.062025 | C21orf119 | chromosome 21 open reading frame 119 |
| 17103327 | 2.22 | 0.015866 | 0.245934 | EBP | emopamil binding protein (sterol isomerase); NULL |
| 16681390 | 2.22 | 0.00381 | 0.135114 | ENO1-IT1 | NULL; ENO1 intronic transcript 1 (non-protein coding) |
| 17072669 | 2.21 | 0.000061 | 0.029261 | MYC | v-myc myelocytomatosis viral oncogene homolog (avian) |
| 17097731 | 2.21 | 0.018252 | 0.260033 | PAPPA-AS1 | PAPPA antisense RNA 1 |
| 16996246 | 2.21 | 0.00383 | 0.13519 | RNF138P1 | ring finger protein 138, E3 ubiquitin protein ligase pseudogene 1 |
| 16681304 | 2.2 | 0.013853 | 0.234499 | ERRFI1 | ERBB receptor feedback inhibitor 1 |
| 16670141 | 2.2 | 0.001066 | 0.078821 | GPR89B; GPR89A; GPR89C; LOC101060636; LOC101060247 | G protein-coupled receptor 89B; G protein-coupled receptor 89A; G protein-coupled receptor 89C; Golgi pH regulator B-like; NULL |
| 16798067 | 2.2 | 0.004319 | 0.142471 | PAR5; SNORD108 | Prader-Willi/Angelman syndrome-5; small nucleolar RNA, C/D box 108 |
| 16880001 | 2.2 | 0.004498 | 0.14568 | STON1-GTF2A1L; STON1; GTF2A1L; FLJ46838 | STON1-GTF2A1L readthrough; stonin 1; general transcription factor IIA, 1-like; FLJ46838 protein; NULL |
| 17060049 | 2.19 | 0.000625 | 0.065902 | DLX5 | distal-less homeobox 5 |
| 16720353 | 2.19 | 0.004001 | 0.136959 | PNPLA2 | patatin-like phospholipase domain containing 2; NULL |
| 16762154 | 2.18 | 0.016522 | 0.249785 | ABCC9 | ATP-binding cassette, sub-family C (CFTR/MRP), member 9 |
| 17104313 | 2.18 | 0.015875 | 0.245934 | AR | androgen receptor |
| 16906419 | 2.18 | 0.049234 | 0.376999 | SLC40A1 | solute carrier family 40 (iron-regulated transporter), member 1; NULL |
| 17078983 | 2.18 | 0.000044 | 0.026559 | TMEM64 | transmembrane protein 64 |
| 17002278 | 2.17 | 0.008364 | 0.189212 | EBF1 | early B-cell factor 1; NULL |
| 17004167 | 2.17 | 0.007028 | 0.175524 | IRF4 | interferon regulatory factor 4 |
| 16962620 | 2.17 | 0.000951 | 0.075552 | LPP-AS2 | LPP antisense RNA 2 |
| 16801707 | 2.17 | 0.001069 | 0.078922 | TLN2 | talin 2; NULL |
| 16893143 | 2.17 | 0.033784 | 0.330213 | TWIST2 | twist basic helix-loop-helix transcription factor 2 |
| 16756627 | 2.17 | 0.003148 | 0.12474 | UNG | uracil-DNA glycosylase |
| 16820937 | 2.16 | 0.001858 | 0.097916 | HP | haptoglobin |
| 17003640 | 2.15 | 0.009282 | 0.19753 | ADAMTS2 | ADAM metallopeptidase with thrombospondin type 1 motif, 2 |
| 17012207 | 2.15 | 0.00566 | 0.160477 | SMPDL3A | sphingomyelin phosphodiesterase, acid-like 3A |
| 16892836 | 2.14 | 0.000004 | 0.011756 | COPS8 | COP9 signalosome subunit 8; NULL |
| 16669963 | 2.14 | 0.001984 | 0.102165 | GPR89C; GPR89B; GPR89A; LOC101060636; LOC101060247 | G protein-coupled receptor 89C; G protein-coupled receptor 89B; G protein-coupled receptor 89A; Golgi pH regulator B-like; NULL |
| 16711125 | 2.14 | 0.008281 | 0.18826 | IDI1 | isopentenyl-diphosphate delta isomerase 1 |
| 16687352 | 2.14 | 0.020242 | 0.26977 | LRP8 | low density lipoprotein receptor-related protein 8, apolipoprotein e receptor |
| 16919408 | 2.14 | 0.001066 | 0.078821 | OSER1 | oxidative stress responsive serine-rich 1 |
| 16798228 | 2.14 | 0.032186 | 0.324187 | SNORD116-26 | small nucleolar RNA, C/D box 116-26 |
| 16684192 | 2.14 | 0.024206 | 0.290032 | SNORD99 | small nucleolar RNA, C/D box 99 |
| 17055614 | 2.13 | 0.000816 | 0.07176 | TWIST1 | twist basic helix-loop-helix transcription factor 1 |
| 17114075 | 2.13 | 0.001107 | 0.080034 | ZNF280C | zinc finger protein 280C |
| 16844936 | 2.12 | 0.010018 | 0.204235 | ACLY | ATP citrate lyase; NULL |
| 16794966 | 2.12 | 0.002581 | 0.11471 | C14orf1 | chromosome 14 open reading frame 1 |
| 16973498 | 2.12 | 0.007449 | 0.179714 | SPON2; LOC100130872 | spondin 2, extracellular matrix protein; uncharacterized LOC100130872; NULL |
| 16999321 | 2.12 | 0.027585 | 0.303882 | ZNF608 | zinc finger protein 608 |
| 16979638 | 2.11 | 0.01685 | 0.25119 | ANKRD50 | ankyrin repeat domain 50 |
| 16723680 | 2.11 | 0.000344 | 0.058679 | LDLRAD3 | low density lipoprotein receptor class A domain containing 3 |
| 16966137 | 2.11 | 0.009806 | 0.202019 | LIAS | lipoic acid synthetase |
| 16753397 | 2.11 | 0.020858 | 0.271942 | MIRLET7I | microRNA let-7i |
| 16889411 | 2.1 | 0.001329 | 0.086385 | CFLAR | CASP8 and FADD-like apoptosis regulator; NULL |
| 16658644 | 2.1 | 0.01109 | 0.213485 | H6PD | hexose-6-phosphate dehydrogenase (glucose 1-dehydrogenase) |
| 16668997 | 2.1 | 0.001469 | 0.090386 | OLFML3 | olfactomedin-like 3 |
| 17005787 | 2.09 | 0.010763 | 0.210577 | HIST1H2AH; HIST1H2AG; HIST1H2AM; HIST1H2AL; HIST1H2AK; HIST1H2AI | histone cluster 1, H2ah; histone cluster 1, H2ag; histone cluster 1, H2am; histone cluster 1, H2al; histone cluster 1, H2ak; histone cluster 1, H2ai |
| 16698356 | 2.09 | 0.01958 | 0.266719 | PIK3C2B | phosphatidylinositol-4-phosphate 3-kinase, catalytic subunit type 2 beta; NULL |
| 16789953 | 2.09 | 0.006249 | 0.166231 | SNORD126 | small nucleolar RNA, C/D box 126 |
| 16943336 | 2.09 | 0.003305 | 0.127676 | TMEM45A | transmembrane protein 45A |
| 16870782 | 2.09 | 0.045564 | 0.368653 | ZNF737 | zinc finger protein 737 |
| 17061467 | 2.08 | 0.015133 | 0.241697 | PUS7 | pseudouridylate synthase 7 homolog (S. cerevisiae); NULL |
| 17020715 | 2.07 | 0.016634 | 0.250301 | LINC00472 | long intergenic non-protein coding RNA 472 |
| 17009289 | 2.07 | 0.002855 | 0.120934 | RUNX2 | runt-related transcription factor 2; NULL |
| 17004642 | 2.07 | 0.006633 | 0.171881 | SNRNP48 | small nuclear ribonucleoprotein 48kDa (U11/U12) |
| 16860168 | 2.06 | 0.0148 | 0.240944 | ZNF431 | zinc finger protein 431 |
| 16884967 | 2.05 | 0.000416 | 0.060761 | DBI | diazepam binding inhibitor (GABA receptor modulator, acyl-CoA binding protein); NULL |
| 17035366 | 2.05 | 0.029701 | 0.313977 | LOC100996357; HCG27 | uncharacterized LOC100996357; HLA complex group 27 (non-protein coding) |
| 16700806 | 2.05 | 0.005545 | 0.160234 | LYST | lysosomal trafficking regulator |
| 16966809 | 2.05 | 0.033908 | 0.330918 | PDGFRA; FIP1L1 | platelet-derived growth factor receptor, alpha polypeptide; FIP1 like 1 (S. cerevisiae); NULL |
| 16820398 | 2.05 | 0.045872 | 0.369131 | SLC7A6; SLC7A6OS | solute carrier family 7 (amino acid transporter light chain, y+L system), member 6; solute carrier family 7, member 6 opposite strand; NULL |
| 16738933 | 2.05 | 0.019409 | 0.265837 | VWCE | von Willebrand factor C and EGF domains; NULL |
| 16836292 | 2.04 | 0.009008 | 0.195097 | ANKFN1 | ankyrin-repeat and fibronectin type III domain containing 1 |
| 16851486 | 2.04 | 0.000018 | 0.020308 | LAMA3 | laminin, alpha 3 |
| 17112996 | 2.04 | 0.000125 | 0.038992 | MORF4L2 | mortality factor 4 like 2; NULL |
| 16956285 | 2.04 | 0.003749 | 0.133926 | PDZRN3 | PDZ domain containing ring finger 3; NULL |
| 16798244 | 2.04 | 0.000582 | 0.064876 | SNRPN; LOC100506948; SNORD116-28; SNORD115-26; SNORD115-13; SNORD115-7; SNORD107; SNHG14 | small nuclear ribonucleoprotein polypeptide N; uncharacterized LOC100506948; small nucleolar RNA, C/D box 116-28; small nucleolar RNA, C/D box 115-26; small nucleolar RNA, C/D box 115-13; small nucleolar RNA, C/D box 115-7; small nucleolar RNA, C/D box 107; NULL |
| 17005276 | 2.04 | 0.01231 | 0.223718 | SOX4 | SRY (sex determining region Y)-box 4 |
| 16768438 | 2.03 | 0.001292 | 0.085375 | BTG1; C12orf79; OTTHUMG00000170091; RP11-24B21.1 | B-cell translocation gene 1, anti-proliferative; chromosome 12 open reading frame 79; NULL |
| 17114701 | 2.03 | 0.008895 | 0.194288 | CDR1 | cerebellar degeneration-related protein 1, 34kDa |
| 16665109 | 2.03 | 0.001136 | 0.080942 | PCSK9 | proprotein convertase subtilisin/kexin type 9 |
| 16798218 | 2.03 | 0.008968 | 0.194868 | SNRPN; LOC100506948; SNORD116-28; SNORD115-26; SNORD115-13; SNORD115-7; SNORD107 | small nuclear ribonucleoprotein polypeptide N; uncharacterized LOC100506948; small nucleolar RNA, C/D box 116-28; small nucleolar RNA, C/D box 115-26; small nucleolar RNA, C/D box 115-13; small nucleolar RNA, C/D box 115-7; small nucleolar RNA, C/D box 107 |
| 16696387 | 2.02 | 0.001087 | 0.079667 | DNM3OS; MIR214; MIR199A2 | DNM3 opposite strand/antisense RNA; microRNA 214; microRNA 199a-2 |
| 16757160 | 2.01 | 0.016073 | 0.246931 | ALDH2 | aldehyde dehydrogenase 2 family (mitochondrial) |
| 16722217 | 2.01 | 0.01356 | 0.231979 | ARNTL | aryl hydrocarbon receptor nuclear translocator-like; NULL |
| 16731169 | 2.01 | 0.025214 | 0.29376 | DLAT | dihydrolipoamide S-acetyltransferase; NULL |
| 16922134 | 2.01 | 0.002245 | 0.108405 | MRAP | melanocortin 2 receptor accessory protein |
| 17077359 | 2.01 | 0.001929 | 0.100266 | PENK | proenkephalin; NULL |
| 16850759 | 2.01 | 0.001121 | 0.080476 | PTPRM | protein tyrosine phosphatase, receptor type, M |
| 16894491 | 2.01 | 0.000153 | 0.042605 | ROCK2 | Rho-associated, coiled-coil containing protein kinase 2; NULL |
| 16917963 | 2 | 0.02433 | 0.290272 | CST2 | cystatin SA |
| 16686557 | 2 | 0.006043 | 0.164182 | PIK3R3; OTTHUMG00000007603; RP11-322N21.2 | phosphoinositide-3-kinase, regulatory subunit 3 (gamma); NULL |
| 16856604 | 2 | 0.009437 | 0.198667 | REEP6 | receptor accessory protein 6 |
| 16924979 | 2 | 0.001833 | 0.097157 | URB1 | URB1 ribosome biogenesis 1 homolog (S. cerevisiae) |
|  |  |  |  |  |  |
|  |  |  |  |  |  |
| **Down-regulated genes on day 7** | |  |  |  |  |
| Transcript Cluster ID | Fold Change (linear) (Induced vs. Control) | ANOVA p-value (Induced vs. Control) | FDR p-value (Induced vs. Control) | Gene Symbol | Description |
| 16990483 | -2 | 0.019984 | 0.268383 | ARHGAP26 | Rho GTPase activating protein 26; NULL |
| 16830912 | -2 | 0.008529 | 0.190514 | MIR3676 | microRNA 3676 |
| 16970132 | -2 | 0.033273 | 0.328674 | MYOZ2 | myozenin 2 |
| 17109716 | -2 | 0.000312 | 0.055193 | RPS6KA3 | ribosomal protein S6 kinase, 90kDa, polypeptide 3 |
| 17056291 | -2 | 0.000939 | 0.075552 | SCRN1 | secernin 1; NULL |
| 16692724 | -2.01 | 0.000181 | 0.045343 | ANP32E | acidic (leucine-rich) nuclear phosphoprotein 32 family, member E |
| 16662047 | -2.01 | 0.001593 | 0.092318 | FAM167B | family with sequence similarity 167, member B |
| 17042860 | -2.01 | 0.023867 | 0.288492 | GPER | G protein-coupled estrogen receptor 1 |
| 16866815 | -2.01 | 0.009519 | 0.199507 | KLF16 | Kruppel-like factor 16 |
| 16809748 | -2.01 | 0.003195 | 0.12508 | MNS1 | meiosis-specific nuclear structural 1 |
| 16830202 | -2.01 | 0.012813 | 0.226943 | XAF1 | XIAP associated factor 1; NULL |
| 16825638 | -2.01 | 0.00023 | 0.049353 | YPEL3 | yippee-like 3 (Drosophila); NULL |
| 17000858 | -2.02 | 0.000313 | 0.055193 | DIAPH1 | diaphanous-related formin 1; NULL |
| 16809610 | -2.02 | 0.002386 | 0.110295 | DYX1C1-CCPG1; CCPG1; DYX1C1 | DYX1C1-CCPG1 readthrough (NMD candidate); cell cycle progression 1; dyslexia susceptibility 1 candidate 1; NULL |
| 16726945 | -2.02 | 0.014925 | 0.241004 | EHBP1L1 | EH domain binding protein 1-like 1 |
| 17015919 | -2.02 | 0.000654 | 0.066659 | KIF13A | kinesin family member 13A |
| 17075478 | -2.02 | 0.024977 | 0.293066 | LOXL2 | lysyl oxidase-like 2 |
| 16820388 | -2.02 | 0.004561 | 0.146846 | PLA2G15 | phospholipase A2, group XV |
| 17093661 | -2.03 | 0.002859 | 0.120934 | FAM214B | family with sequence similarity 214, member B |
| 16925341 | -2.03 | 0.029045 | 0.310953 | RCAN1 | regulator of calcineurin 1; NULL |
| 16951756 | -2.03 | 0.034837 | 0.334557 | SLC4A7 | solute carrier family 4, sodium bicarbonate cotransporter, member 7; NULL |
| 16692667 | -2.04 | 0.00044 | 0.060845 | MTMR11 | myotubularin related protein 11; NULL |
| 17061104 | -2.04 | 0.048767 | 0.375829 | RASA4B; RASA4 | RAS p21 protein activator 4B; RAS p21 protein activator 4; NULL |
| 16964050 | -2.04 | 0.000384 | 0.059593 | WHSC1 | Wolf-Hirschhorn syndrome candidate 1; NULL |
| 16806823 | -2.05 | 0.014446 | 0.238228 | AVEN | apoptosis, caspase activation inhibitor |
| 16659357 | -2.05 | 0.001375 | 0.087738 | C1orf158 | chromosome 1 open reading frame 158 |
| 17042895 | -2.05 | 0.045456 | 0.368235 | MAFK | v-maf musculoaponeurotic fibrosarcoma oncogene homolog K (avian); NULL |
| 16806870 | -2.05 | 0.000738 | 0.068863 | SLC12A6 | solute carrier family 12 (potassium/chloride transporters), member 6; NULL |
| 16964597 | -2.05 | 0.001781 | 0.096048 | STK32B | serine/threonine kinase 32B |
| 16677556 | -2.05 | 0.002212 | 0.107621 | TGFB2 | transforming growth factor, beta 2 |
| 17048879 | -2.06 | 0.019941 | 0.268244 | ARPC1B | actin related protein 2/3 complex, subunit 1B, 41kDa; NULL |
| 16723662 | -2.06 | 0.007184 | 0.176841 | FJX1 | four jointed box 1 (Drosophila) |
| 16888865 | -2.07 | 0.003818 | 0.13519 | GLS | glutaminase; NULL |
| 16999776 | -2.07 | 0.040689 | 0.35367 | IRF1 | interferon regulatory factor 1 |
| 16809457 | -2.07 | 0.000026 | 0.020308 | MYO5A | myosin VA (heavy chain 12, myoxin); NULL |
| 16742454 | -2.07 | 0.000187 | 0.046132 | PAK1 | p21 protein (Cdc42/Rac)-activated kinase 1; NULL |
| 16813342 | -2.07 | 0.039951 | 0.351395 | PRC1 | protein regulator of cytokinesis 1; NULL |
| 16761631 | -2.08 | 0.001914 | 0.099772 | DUSP16 | dual specificity phosphatase 16 |
| 17043882 | -2.08 | 0.016827 | 0.251174 | HDAC9 | histone deacetylase 9; NULL |
| 17106003 | -2.08 | 0.002057 | 0.10376 | KRT18P49 | NULL; keratin 18 pseudogene 49 |
| 16684800 | -2.08 | 0.01637 | 0.24895 | TMEM54 | transmembrane protein 54; NULL |
| 17011593 | -2.09 | 0.000603 | 0.06538 | FIG4 | FIG4 homolog, SAC1 lipid phosphatase domain containing (S. cerevisiae) |
| 16956149 | -2.09 | 0.001768 | 0.095788 | FOXP1 | forkhead box P1; NULL |
| 16839352 | -2.09 | 0.002884 | 0.120934 | MYO1C | myosin IC; NULL |
| 16842850 | -2.1 | 0.000485 | 0.061367 | DHRS13 | dehydrogenase/reductase (SDR family) member 13 |
| 16922501 | -2.1 | 0.003302 | 0.127676 | DOPEY2 | dopey family member 2 |
| 16769159 | -2.1 | 0.000399 | 0.060302 | GNPTAB | N-acetylglucosamine-1-phosphate transferase, alpha and beta subunits |
| 16662885 | -2.1 | 0.000463 | 0.060845 | KIAA0754 | KIAA0754 |
| 17065938 | -2.1 | 0.027455 | 0.303335 | RNA5SP255 | RNA, 5S ribosomal pseudogene 255 |
| 16670894 | -2.1 | 0.003418 | 0.129436 | TUFT1 | tuftelin 1 |
| 17059756 | -2.11 | 0.009622 | 0.200651 | CDK6 | cyclin-dependent kinase 6 |
| 16828471 | -2.11 | 0.034492 | 0.333487 | CHST6 | carbohydrate (N-acetylglucosamine 6-O) sulfotransferase 6 |
| 17067941 | -2.11 | 0.001026 | 0.077523 | GPR124 | G protein-coupled receptor 124 |
| 16817692 | -2.11 | 0.00976 | 0.201499 | MVP | major vault protein; NULL |
| 16969911 | -2.12 | 0.011404 | 0.216673 | ANK2 | ankyrin 2, neuronal; NULL |
| 16716371 | -2.12 | 0.037446 | 0.343258 | CH25H | cholesterol 25-hydroxylase |
| 16735895 | -2.12 | 0.003652 | 0.133118 | DKK3 | dickkopf WNT signaling pathway inhibitor 3; NULL |
| 16711909 | -2.12 | 0.011075 | 0.213485 | FRMD4A | FERM domain containing 4A; NULL |
| 16664005 | -2.12 | 0.001499 | 0.09042 | PLK3 | polo-like kinase 3 |
| 16674521 | -2.12 | 0.001555 | 0.091522 | QSOX1; FLJ23867 | quiescin Q6 sulfhydryl oxidase 1; uncharacterized protein FLJ23867 |
| 16879791 | -2.12 | 0.011376 | 0.21659 | SOCS5 | suppressor of cytokine signaling 5 |
| 17018720 | -2.12 | 0.029198 | 0.311477 | TMEM217 | transmembrane protein 217 |
| 16957396 | -2.13 | 0.004264 | 0.141803 | CCDC80 | coiled-coil domain containing 80 |
| 16870432 | -2.13 | 0.000419 | 0.060761 | FKBP8 | FK506 binding protein 8, 38kDa; NULL |
| 17013126 | -2.13 | 0.010105 | 0.204765 | GPR126 | G protein-coupled receptor 126; NULL |
| 16958403 | -2.13 | 0.000163 | 0.043351 | HEG1 | heart development protein with EGF-like domains 1 |
| 16788258 | -2.13 | 0.002759 | 0.118638 | HHIPL1 | HHIP-like 1 |
| 16802960 | -2.13 | 0.001632 | 0.093579 | ISLR | immunoglobulin superfamily containing leucine-rich repeat |
| 17016946 | -2.13 | 0.000697 | 0.067528 | PPP1R18; OTTHUMG00000148752; KIAA1949; OTTHUMG00000031537; OTTHUMG00000148941; OTTHUMG00000149220; OTTHUMG00000149456; OTTHUMG00000004836; OTTHUMG00000149990 | protein phosphatase 1, regulatory subunit 18; NULL |
| 16823692 | -2.14 | 0.003166 | 0.124894 | NAGPA | N-acetylglucosamine-1-phosphodiester alpha-N-acetylglucosaminidase; NULL |
| 16669087 | -2.14 | 0.006896 | 0.174024 | SLC22A15 | solute carrier family 22, member 15 |
| 17077222 | -2.14 | 0.000591 | 0.064964 | TCEA1; TCEA1P2 | transcription elongation factor A (SII), 1; NULL; transcription elongation factor A (SII), 1 pseudogene 2 |
| 16732755 | -2.15 | 0.000358 | 0.059397 | GRAMD1B | GRAM domain containing 1B; NULL |
| 16872551 | -2.15 | 0.007691 | 0.182065 | TGFB1 | transforming growth factor, beta 1 |
| 16912379 | -2.15 | 0.032755 | 0.326589 | TPX2 | TPX2, microtubule-associated, homolog (Xenopus laevis) |
| 16659054 | -2.16 | 0.001361 | 0.087531 | AGTRAP | angiotensin II receptor-associated protein; NULL |
| 16700074 | -2.16 | 0.000006 | 0.01431 | CDC42BPA | CDC42 binding protein kinase alpha (DMPK-like); NULL |
| 17015637 | -2.16 | 0.003107 | 0.124447 | ELOVL2 | ELOVL fatty acid elongase 2 |
| 16661141 | -2.16 | 0.000115 | 0.038672 | SH3BGRL3 | SH3 domain binding glutamic acid-rich protein like 3 |
| 16803743 | -2.17 | 0.01786 | 0.257832 | ABHD17C | abhydrolase domain containing 17C |
| 16970080 | -2.17 | 0.004347 | 0.142789 | CEP170P1 | centrosomal protein 170kDa pseudogene 1 |
| 16768675 | -2.17 | 0.029901 | 0.314977 | FGD6 | FYVE, RhoGEF and PH domain containing 6 |
| 16842834 | -2.17 | 0.003507 | 0.129909 | FLOT2 | flotillin 2 |
| 16958124 | -2.17 | 0.00302 | 0.123231 | PARP9 | poly (ADP-ribose) polymerase family, member 9 |
| 16733920 | -2.17 | 0.004914 | 0.150656 | RNH1 | ribonuclease/angiogenin inhibitor 1; NULL |
| 17024775 | -2.17 | 0.021216 | 0.274101 | SYNE1 | spectrin repeat containing, nuclear envelope 1; NULL |
| 17106357 | -2.18 | 0.000819 | 0.071782 | PLS3 | plastin 3; NULL |
| 16765254 | -2.18 | 0.005931 | 0.162689 | RARG | retinoic acid receptor, gamma; NULL |
| 17070013 | -2.18 | 0.04393 | 0.364742 | RDH10 | retinol dehydrogenase 10 (all-trans) |
| 17098698 | -2.19 | 0.002924 | 0.120934 | FAM102A | family with sequence similarity 102, member A; NULL |
| 17071625 | -2.19 | 0.003309 | 0.127676 | FZD6 | frizzled family receptor 6; NULL |
| 16807288 | -2.19 | 0.017125 | 0.253176 | GPR176 | G protein-coupled receptor 176 |
| 16966049 | -2.19 | 0.002327 | 0.109806 | KLHL5 | kelch-like family member 5 |
| 16669105 | -2.19 | 0.033756 | 0.330153 | MAB21L3 | mab-21-like 3 (C. elegans) |
| 16725664 | -2.19 | 0.035196 | 0.335645 | RPLP0P2 | ribosomal protein, large, P0 pseudogene 2 |
| 16839019 | -2.19 | 0.011776 | 0.219333 | SLC16A3 | solute carrier family 16, member 3 (monocarboxylic acid transporter 4); NULL |
| 16777777 | -2.19 | 0.008308 | 0.188543 | SLC7A1 | solute carrier family 7 (cationic amino acid transporter, y+ system), member 1 |
| 16859802 | -2.19 | 0.002213 | 0.107621 | SSBP4 | single stranded DNA binding protein 4; NULL |
| 16931709 | -2.2 | 0.000279 | 0.053107 | ADM2 | adrenomedullin 2 |
| 17108003 | -2.2 | 0.022042 | 0.278069 | BGN | biglycan; NULL |
| 16743978 | -2.2 | 0.002006 | 0.102169 | GUCY1A2 | guanylate cyclase 1, soluble, alpha 2 |
| 16738630 | -2.2 | 0.02374 | 0.287892 | LPXN | leupaxin |
| 16834931 | -2.21 | 0.001924 | 0.100186 | FMNL1 | formin-like 1 |
| 16931766 | -2.21 | 0.014257 | 0.237174 | KLHDC7B | kelch domain containing 7B |
| 16894283 | -2.21 | 0.005974 | 0.163231 | MBOAT2 | membrane bound O-acyltransferase domain containing 2; NULL |
| 16969558 | -2.21 | 0.005611 | 0.160234 | SGMS2 | sphingomyelin synthase 2 |
| 16854904 | -2.22 | 0.000496 | 0.061679 | PSTPIP2 | proline-serine-threonine phosphatase interacting protein 2 |
| 16705810 | -2.22 | 0.047418 | 0.372167 | UNC5B | unc-5 homolog B (C. elegans) |
| 16944344 | -2.23 | 0.023271 | 0.285275 | ARHGAP31 | Rho GTPase activating protein 31 |
| 16789743 | -2.23 | 0.001221 | 0.082663 | CRIP1; OTTHUMG00000029910; AL928654.7 | cysteine-rich protein 1 (intestinal); NULL |
| 16773759 | -2.23 | 0.027002 | 0.30095 | FRY | furry homolog (Drosophila) |
| 16677071 | -2.23 | 0.02302 | 0.284485 | SERTAD4 | SERTA domain containing 4 |
| 16880669 | -2.24 | 0.00181 | 0.096668 | LGALSL | lectin, galactoside-binding-like; NULL |
| 16808316 | -2.24 | 0.000199 | 0.046364 | PPIP5K1; OTTHUMG00000059903; AC011330.5 | diphosphoinositol pentakisphosphate kinase 1; NULL |
| 16794980 | -2.24 | 0.001497 | 0.09042 | TGFB3 | transforming growth factor, beta 3 |
| 17080468 | -2.25 | 0.003996 | 0.136959 | SAMD12 | sterile alpha motif domain containing 12 |
| 16751319 | -2.25 | 0.001097 | 0.079916 | SCN8A | sodium channel, voltage gated, type VIII, alpha subunit |
| 17050522 | -2.25 | 0.009368 | 0.198401 | TES | testis derived transcript (3 LIM domains); NULL |
| 17077502 | -2.25 | 0.026577 | 0.299322 | TOX | thymocyte selection-associated high mobility group box |
| 16854437 | -2.26 | 0.003947 | 0.136271 | CDH2 | cadherin 2, type 1, N-cadherin (neuronal) |
| 16782862 | -2.26 | 0.002245 | 0.108405 | NYNRIN | NYN domain and retroviral integrase containing |
| 16979339 | -2.26 | 0.008873 | 0.194288 | PDE5A | phosphodiesterase 5A, cGMP-specific; NULL |
| 16803710 | -2.28 | 0.022978 | 0.284357 | ARNT2 | aryl-hydrocarbon receptor nuclear translocator 2; NULL |
| 16938271 | -2.28 | 0.000065 | 0.029652 | KAT2B | K(lysine) acetyltransferase 2B; NULL |
| 16995645 | -2.29 | 0.00064 | 0.06627 | DAB2 | Dab, mitogen-responsive phosphoprotein, homolog 2 (Drosophila); NULL |
| 17063480 | -2.29 | 0.005024 | 0.152584 | PARP12 | poly (ADP-ribose) polymerase family, member 12; NULL |
| 17087308 | -2.29 | 0.000009 | 0.016227 | TDRD7 | tudor domain containing 7 |
| 16845336 | -2.29 | 0.00038 | 0.059593 | VAT1 | vesicle amine transport protein 1 homolog (T. californica) |
| 16863753 | -2.3 | 0.00316 | 0.124894 | EMP3 | epithelial membrane protein 3 |
| 16996146 | -2.3 | 0.001896 | 0.099075 | ESM1 | endothelial cell-specific molecule 1 |
| 16938378 | -2.3 | 0.000348 | 0.059114 | NR1D2 | nuclear receptor subfamily 1, group D, member 2 |
| 16893531 | -2.31 | 0.000575 | 0.064876 | BOK | BCL2-related ovarian killer |
| 16854856 | -2.31 | 0.00539 | 0.15763 | EPG5 | ectopic P-granules autophagy protein 5 homolog (C. elegans) |
| 16674845 | -2.31 | 0.007823 | 0.183239 | LAMC2 | laminin, gamma 2 |
| 17013728 | -2.32 | 0.0001 | 0.037536 | MTHFD1L; LOC100996643 | methylenetetrahydrofolate dehydrogenase (NADP+ dependent) 1-like; monofunctional C1-tetrahydrofolate synthase, mitochondrial-like; NULL |
| 16888912 | -2.32 | 0.000756 | 0.069472 | MYO1B | myosin IB; NULL |
| 16805474 | -2.33 | 0.000254 | 0.052003 | ARRDC4 | arrestin domain containing 4 |
| 16762337 | -2.33 | 0.002985 | 0.122314 | BCAT1 | branched chain amino-acid transaminase 1, cytosolic |
| 16743056 | -2.33 | 0.000278 | 0.053107 | ME3 | malic enzyme 3, NADP(+)-dependent, mitochondrial; NULL |
| 16852666 | -2.33 | 0.000754 | 0.069414 | SEC11C | SEC11 homolog C (S. cerevisiae) |
| 16667702 | -2.33 | 0.015416 | 0.242778 | VCAM1 | vascular cell adhesion molecule 1 |
| 17060061 | -2.34 | 0.000402 | 0.060338 | ASNS | asparagine synthetase (glutamine-hydrolyzing); NULL |
| 16901393 | -2.34 | 0.028238 | 0.308107 | FHL2 | four and a half LIM domains 2 |
| 16873562 | -2.34 | 1.58E-07 | 0.005199 | PTGIR | prostaglandin I2 (prostacyclin) receptor (IP) |
| 16867784 | -2.35 | 0.024982 | 0.293066 | C3 | complement component 3; NULL |
| 16879067 | -2.35 | 0.011281 | 0.215459 | CRIM1 | cysteine rich transmembrane BMP regulator 1 (chordin-like); NULL |
| 17080450 | -2.35 | 0.000024 | 0.020308 | EXT1 | exostosin glycosyltransferase 1 |
| 16740412 | -2.35 | 0.001177 | 0.081639 | LTBP3 | latent transforming growth factor beta binding protein 3; NULL |
| 17077826 | -2.35 | 0.000453 | 0.060845 | MYBL1; LOC645895 | v-myb myeloblastosis viral oncogene homolog (avian)-like 1; uncharacterized LOC645895 |
| 16661862 | -2.35 | 0.006691 | 0.17283 | TINAGL1 | tubulointerstitial nephritis antigen-like 1; NULL |
| 16980470 | -2.36 | 0.047947 | 0.373733 | NR3C2 | nuclear receptor subfamily 3, group C, member 2; NULL |
| 16965519 | -2.36 | 0.025369 | 0.294772 | SOD3 | superoxide dismutase 3, extracellular |
| 16833629 | -2.37 | 0.008045 | 0.185442 | ARHGAP23; ARHGAP23P1; OTTHUMG00000176364; RP11-812E19.6 | Rho GTPase activating protein 23; NULL; Rho GTPase activating protein 23 pseudogene 1 |
| 16691314 | -2.37 | 0.045177 | 0.367593 | TSPAN2 | tetraspanin 2 |
| 16771680 | -2.38 | 0.000033 | 0.023297 | CLIP1 | CAP-GLY domain containing linker protein 1; NULL |
| 16770915 | -2.38 | 0.01611 | 0.247172 | WSB2 | WD repeat and SOCS box containing 2; NULL |
| 16775434 | -2.39 | 0.002876 | 0.120934 | LMO7; OTTHUMG00000172802; RP11-29G8.3 | LIM domain 7; NULL |
| 17070061 | -2.39 | 0.010243 | 0.205694 | LY96 | lymphocyte antigen 96 |
| 16873060 | -2.39 | 0.009228 | 0.197361 | PLAUR | plasminogen activator, urokinase receptor; NULL |
| 16844585 | -2.4 | 0.010419 | 0.207567 | KRTAP2-3; KRTAP2-4 | keratin associated protein 2-3; keratin associated protein 2-4 |
| 16899357 | -2.4 | 0.030021 | 0.315313 | LOXL3 | lysyl oxidase-like 3; NULL |
| 17020464 | -2.4 | 0.002839 | 0.12053 | RAB23 | RAB23, member RAS oncogene family |
| 16962380 | -2.41 | 0.001191 | 0.082121 | ETV5 | ets variant 5; NULL |
| 16764398 | -2.41 | 0.016471 | 0.249561 | FMNL3 | formin-like 3; NULL |
| 16971966 | -2.41 | 0.046907 | 0.371143 | FNIP2 | folliculin interacting protein 2 |
| 16736821 | -2.41 | 0.017937 | 0.258075 | LGR4 | leucine-rich repeat containing G protein-coupled receptor 4 |
| 16918351 | -2.42 | 0.000044 | 0.026559 | COMMD7 | COMM domain containing 7 |
| 16684674 | -2.42 | 0.004885 | 0.15034 | MTMR9LP | myotubularin related protein 9-like, pseudogene; NULL |
| 16962661 | -2.43 | 0.020363 | 0.270363 | CLDN1 | claudin 1 |
| 16942103 | -2.44 | 0.003498 | 0.129909 | FLNB | filamin B, beta; NULL |
| 16660282 | -2.44 | 0.003707 | 0.133926 | PLA2G5 | phospholipase A2, group V; NULL |
| 16987610 | -2.44 | 0.001594 | 0.092318 | RGMB | RGM domain family, member B; NULL |
| 16679301 | -2.45 | 0.000897 | 0.075178 | FMN2 | formin 2; NULL |
| 16669389 | -2.45 | 0.008323 | 0.188626 | PHGDH | phosphoglycerate dehydrogenase |
| 17102041 | -2.45 | 0.000437 | 0.060845 | SMS | spermine synthase |
| 16823750 | -2.46 | 0.005193 | 0.155149 | CARHSP1 | calcium regulated heat stable protein 1, 24kDa; NULL |
| 16791898 | -2.47 | 0.00048 | 0.061367 | CFL2 | cofilin 2 (muscle) |
| 17114829 | -2.47 | 0.000576 | 0.064876 | IDS; OTTHUMG00000022618; AF011889.5 | iduronate 2-sulfatase; NULL |
| 16944695 | -2.47 | 0.000461 | 0.060845 | PARP14 | poly (ADP-ribose) polymerase family, member 14 |
| 17062985 | -2.47 | 0.018902 | 0.262723 | PODXL | podocalyxin-like; NULL |
| 16858991 | -2.48 | 0.00095 | 0.075552 | PKN1 | protein kinase N1 |
| 17008105 | -2.48 | 0.000192 | 0.046364 | TBC1D22B | TBC1 domain family, member 22B |
| 16747184 | -2.5 | 0.014519 | 0.238546 | CD9 | CD9 molecule |
| 16815498 | -2.5 | 0.004271 | 0.141949 | GLIS2 | GLIS family zinc finger 2 |
| 16818842 | -2.51 | 0.000497 | 0.061679 | CYLD | cylindromatosis (turban tumor syndrome); NULL |
| 16754373 | -2.51 | 0.006714 | 0.173139 | GLIPR1 | GLI pathogenesis-related 1 |
| 17010760 | -2.52 | 0.001279 | 0.084751 | NT5E | 5'-nucleotidase, ecto (CD73); NULL |
| 16779435 | -2.52 | 0.000105 | 0.038079 | THSD1 | thrombospondin, type I, domain containing 1 |
| 16901068 | -2.53 | 0.00151 | 0.09042 | AFF3 | AF4/FMR2 family, member 3; NULL |
| 16683574 | -2.53 | 0.000007 | 0.01431 | STPG1 | sperm-tail PG-rich repeat containing 1 |
| 16820620 | -2.53 | 0.000127 | 0.039172 | WWP2 | WW domain containing E3 ubiquitin protein ligase 2; NULL |
| 16798951 | -2.54 | 0.037608 | 0.343898 | GREM1 | gremlin 1, DAN family BMP antagonist |
| 16979845 | -2.54 | 0.002201 | 0.107621 | KRT18P54 | NULL; keratin 18 pseudogene 54 |
| 16790614 | -2.55 | 0.004814 | 0.148921 | AJUBA | ajuba LIM protein; NULL |
| 16840982 | -2.55 | 0.04533 | 0.368151 | MYH10 | myosin, heavy chain 10, non-muscle; NULL |
| 16756310 | -2.55 | 0.00001 | 0.017666 | TCP11L2 | t-complex 11, testis-specific-like 2 |
| 16702547 | -2.56 | 0.000135 | 0.039996 | OPTN | optineurin; NULL |
| 16906571 | -2.56 | 0.02591 | 0.296629 | STAT4 | signal transducer and activator of transcription 4; NULL |
| 17078870 | -2.58 | 0.001089 | 0.079667 | MMP16 | matrix metallopeptidase 16 (membrane-inserted) |
| 16797444 | -2.59 | 0.022302 | 0.279654 | IGHV1-3 | immunoglobulin heavy variable 1-3 |
| 16886757 | -2.59 | 0.000438 | 0.060845 | PKP4 | plakophilin 4; NULL |
| 16739192 | -2.59 | 0.008126 | 0.186334 | RAB3IL1 | RAB3A interacting protein (rabin3)-like 1; NULL |
| 16717224 | -2.6 | 0.00172 | 0.095243 | MORN4 | MORN repeat containing 4 |
| 16767851 | -2.61 | 0.003089 | 0.124234 | E2F7 | E2F transcription factor 7; NULL |
| 16804062 | -2.61 | 0.024517 | 0.29099 | TM6SF1 | transmembrane 6 superfamily member 1 |
| 16945768 | -2.62 | 0.001019 | 0.077385 | CCRL1 | chemokine (C-C motif) receptor-like 1 |
| 16884523 | -2.62 | 0.032021 | 0.323344 | SLC20A1 | solute carrier family 20 (phosphate transporter), member 1 |
| 16764724 | -2.63 | 0.001529 | 0.09101 | SMAGP | small cell adhesion glycoprotein |
| 16947551 | -2.64 | 0.013482 | 0.231541 | C3orf80 | chromosome 3 open reading frame 80 |
| 16858970 | -2.64 | 0.003627 | 0.132519 | CD97 | CD97 molecule |
| 16694701 | -2.66 | 0.002583 | 0.11471 | CRABP2 | cellular retinoic acid binding protein 2 |
| 16783602 | -2.66 | 0.002495 | 0.112272 | SSTR1 | somatostatin receptor 1 |
| 17100201 | -2.69 | 0.002413 | 0.110869 | CLIC3 | chloride intracellular channel 3 |
| 17055937 | -2.69 | 0.002079 | 0.104247 | OSBPL3 | oxysterol binding protein-like 3; NULL |
| 16855545 | -2.7 | 0.004127 | 0.139154 | ALPK2 | alpha-kinase 2 |
| 16816542 | -2.7 | 0.002167 | 0.106729 | IQCK | IQ motif containing K; NULL |
| 16864393 | -2.72 | 0.000249 | 0.05183 | ATF5; MIR4751 | activating transcription factor 5; microRNA 4751 |
| 16820508 | -2.72 | 0.001973 | 0.101968 | TANGO6; TMCO7 | transport and golgi organization 6 homolog (Drosophila); NULL |
| 16937024 | -2.74 | 0.01079 | 0.210764 | BHLHE40 | basic helix-loop-helix family, member e40 |
| 16789723 | -2.74 | 0.000673 | 0.067361 | CRIP2 | cysteine-rich protein 2; NULL |
| 17094064 | -2.74 | 0.004993 | 0.151839 | SHB | Src homology 2 domain containing adaptor protein B |
| 16696425 | -2.74 | 0.003239 | 0.12634 | TNFSF4 | tumor necrosis factor (ligand) superfamily, member 4 |
| 17051626 | -2.75 | 0.020763 | 0.271942 | MEST | mesoderm specific transcript; NULL |
| 16749459 | -2.75 | 0.00023 | 0.049353 | PPFIBP1 | PTPRF interacting protein, binding protein 1 (liprin beta 1); NULL |
| 16685889 | -2.75 | 0.005376 | 0.15763 | SCMH1 | sex comb on midleg homolog 1 (Drosophila); NULL |
| 16910609 | -2.75 | 0.003738 | 0.133926 | TRIB3 | tribbles homolog 3 (Drosophila) |
| 16960911 | -2.76 | 0.022352 | 0.279838 | LXN | latexin |
| 17066018 | -2.78 | 0.000022 | 0.020308 | ZDHHC2 | zinc finger, DHHC-type containing 2 |
| 16717235 | -2.79 | 0.025662 | 0.295968 | AVPI1 | arginine vasopressin-induced 1 |
| 16922759 | -2.79 | 0.035863 | 0.338395 | KCNJ15 | potassium inwardly-rectifying channel, subfamily J, member 15; NULL |
| 16808304 | -2.79 | 0.001721 | 0.095243 | PPIP5K1 | diphosphoinositol pentakisphosphate kinase 1 |
| 16928533 | -2.8 | 0.001724 | 0.095243 | ASPHD2 | aspartate beta-hydroxylase domain containing 2 |
| 16728261 | -2.8 | 4.85E-07 | 0.005199 | CCND1 | cyclin D1 |
| 16745961 | -2.83 | 0.000243 | 0.051077 | KIRREL3 | kin of IRRE like 3 (Drosophila); NULL |
| 16729789 | -2.84 | 0.000205 | 0.046619 | PRSS23 | protease, serine, 23; NULL |
| 16670479 | -2.85 | 0.012675 | 0.226303 | C1orf51 | chromosome 1 open reading frame 51; NULL |
| 16663514 | -2.86 | 0.030367 | 0.316025 | CDC20 | cell division cycle 20 |
| 16701037 | -2.86 | 0.041021 | 0.35491 | GREM2 | gremlin 2, DAN family BMP antagonist |
| 16909081 | -2.88 | 0.008216 | 0.187422 | DOCK10 | dedicator of cytokinesis 10; NULL |
| 16673126 | -2.89 | 0.022014 | 0.277986 | RGS4 | regulator of G-protein signaling 4; NULL |
| 16960844 | -2.89 | 0.003938 | 0.136143 | VEPH1 | ventricular zone expressed PH domain-containing 1; NULL |
| 16688799 | -2.9 | 0.004028 | 0.137404 | ELTD1 | EGF, latrophilin and seven transmembrane domain containing 1 |
| 16739479 | -2.91 | 0.032942 | 0.327401 | LRRN4CL | LRRN4 C-terminal like |
| 17019190 | -2.93 | 0.000084 | 0.033046 | C6orf132 | chromosome 6 open reading frame 132 |
| 16807763 | -2.93 | 0.000951 | 0.075552 | EHD4 | EH-domain containing 4 |
| 17014114 | -2.93 | 0.015225 | 0.242084 | SYNJ2 | synaptojanin 2; NULL |
| 16986138 | -2.95 | 0.016856 | 0.251206 | ARHGEF28 | Rho guanine nucleotide exchange factor (GEF) 28; NULL |
| 16700400 | -2.96 | 0.004108 | 0.138785 | C1orf198 | chromosome 1 open reading frame 198 |
| 17012342 | -2.96 | 0.000361 | 0.059473 | HINT3 | histidine triad nucleotide binding protein 3 |
| 16693474 | -2.96 | 0.000115 | 0.038672 | S100A16 | S100 calcium binding protein A16; NULL |
| 16660785 | -2.98 | 0.000198 | 0.046364 | NIPAL3 | NIPA-like domain containing 3; NULL |
| 17010198 | -2.98 | 0.031624 | 0.32175 | RIMS1 | regulating synaptic membrane exocytosis 1; NULL |
| 16892075 | -3 | 0.000372 | 0.059593 | ARMC9 | armadillo repeat containing 9; NULL |
| 16802519 | -3 | 0.000384 | 0.059593 | KIF23 | kinesin family member 23 |
| 17025844 | -3 | 0.01154 | 0.21793 | THBS2 | thrombospondin 2 |
| 17051553 | -3.02 | 0.006846 | 0.17365 | CPA4 | carboxypeptidase A4 |
| 16968331 | -3.02 | 0.003863 | 0.135297 | FGF5 | fibroblast growth factor 5 |
| 16889563 | -3.05 | 0.012699 | 0.226399 | FZD7 | frizzled family receptor 7 |
| 16796694 | -3.05 | 0.000017 | 0.020308 | WARS | tryptophanyl-tRNA synthetase; NULL |
| 16821377 | -3.07 | 0.00381 | 0.135114 | CDH13 | cadherin 13, H-cadherin (heart); NULL |
| 16800229 | -3.07 | 0.000861 | 0.073625 | MAP1A | microtubule-associated protein 1A |
| 16706180 | -3.07 | 0.001812 | 0.096681 | PLAU | plasminogen activator, urokinase |
| 16839220 | -3.08 | 0.002078 | 0.104247 | FAM101B | family with sequence similarity 101, member B |
| 16767794 | -3.09 | 0.000244 | 0.051077 | OSBPL8 | oxysterol binding protein-like 8; NULL |
| 16697544 | -3.1 | 0.015749 | 0.245328 | ASPM | asp (abnormal spindle) homolog, microcephaly associated (Drosophila) |
| 17096904 | -3.11 | 0.000307 | 0.055116 | CTNNAL1 | catenin (cadherin-associated protein), alpha-like 1 |
| 16942648 | -3.11 | 0.011404 | 0.216673 | GXYLT2 | glucoside xylosyltransferase 2 |
| 17051286 | -3.12 | 0.023277 | 0.285275 | FLNC | filamin C, gamma |
| 16672214 | -3.12 | 0.021296 | 0.274101 | PEAR1 | platelet endothelial aggregation receptor 1; NULL |
| 16974121 | -3.14 | 0.000017 | 0.020308 | AFAP1 | actin filament associated protein 1 |
| 16929442 | -3.14 | 0.006647 | 0.172073 | TIMP3 | TIMP metallopeptidase inhibitor 3 |
| 16969686 | -3.15 | 0.001412 | 0.089143 | CCDC109B | coiled-coil domain containing 109B |
| 16984689 | -3.15 | 0.00747 | 0.179891 | ITGA2 | integrin, alpha 2 (CD49B, alpha 2 subunit of VLA-2 receptor); NULL |
| 17059776 | -3.17 | 0.000277 | 0.053107 | SAMD9L | sterile alpha motif domain containing 9-like |
| 17013851 | -3.18 | 0.00349 | 0.129909 | MYCT1 | myc target 1 |
| 16920730 | -3.19 | 0.001415 | 0.089219 | APCDD1L | adenomatosis polyposis coli down-regulated 1-like |
| 16862439 | -3.19 | 0.000611 | 0.06538 | AXL | AXL receptor tyrosine kinase |
| 16682098 | -3.19 | 0.000101 | 0.037634 | EPHA2 | EPH receptor A2 |
| 16663621 | -3.21 | 0.027065 | 0.301438 | PTPRF | protein tyrosine phosphatase, receptor type, F; NULL |
| 16817017 | -3.22 | 0.021392 | 0.274342 | PLK1 | polo-like kinase 1; NULL |
| 16778241 | -3.23 | 0.014649 | 0.239567 | POSTN | periostin, osteoblast specific factor; NULL |
| 17050797 | -3.24 | 0.00043 | 0.060845 | CPED1 | cadherin-like and PC-esterase domain containing 1 |
| 16707551 | -3.25 | 0.036901 | 0.342295 | CEP55 | centrosomal protein 55kDa |
| 16938875 | -3.25 | 0.007127 | 0.17674 | STAC | SH3 and cysteine rich domain |
| 17004612 | -3.28 | 0.037858 | 0.344415 | DSP | desmoplakin |
| 16693976 | -3.29 | 0.000945 | 0.075552 | MUC1 | mucin 1, cell surface associated; NULL |
| 17007543 | -3.3 | 0.001564 | 0.091522 | ITPR3 | inositol 1,4,5-trisphosphate receptor, type 3 |
| 16899413 | -3.32 | 0.000495 | 0.061679 | EVA1A | eva-1 homolog A (C. elegans); NULL |
| 16851383 | -3.34 | 0.000205 | 0.046619 | GATA6 | GATA binding protein 6 |
| 16965252 | -3.35 | 0.001318 | 0.085936 | BST1 | bone marrow stromal cell antigen 1 |
| 17007910 | -3.35 | 0.001436 | 0.089708 | MAPK13 | mitogen-activated protein kinase 13 |
| 16962632 | -3.36 | 0.005524 | 0.160202 | LEPREL1 | leprecan-like 1; NULL |
| 16916901 | -3.36 | 0.021643 | 0.275712 | RASSF2 | Ras association (RalGDS/AF-6) domain family member 2 |
| 16749423 | -3.37 | 0.0013 | 0.085553 | ARNTL2 | aryl hydrocarbon receptor nuclear translocator-like 2 |
| 17056426 | -3.37 | 0.005823 | 0.161651 | PDE1C | phosphodiesterase 1C, calmodulin-dependent 70kDa |
| 17025191 | -3.38 | 0.005354 | 0.15745 | EZR | ezrin |
| 17083793 | -3.39 | 0.000338 | 0.058169 | ADAMTSL1 | ADAMTS-like 1; NULL |
| 17060167 | -3.39 | 0.001636 | 0.09361 | TMEM130 | transmembrane protein 130; NULL |
| 16903771 | -3.45 | 0.002804 | 0.11955 | CACNB4 | calcium channel, voltage-dependent, beta 4 subunit |
| 17045622 | -3.46 | 0.000593 | 0.064964 | AEBP1; MIR4649 | AE binding protein 1; microRNA 4649; NULL |
| 16802653 | -3.49 | 0.000021 | 0.020308 | THSD4 | thrombospondin, type I, domain containing 4 |
| 17076726 | -3.51 | 0.005726 | 0.161352 | PLAT | plasminogen activator, tissue; NULL |
| 16723422 | -3.52 | 0.000097 | 0.036378 | KIAA1549L | KIAA1549-like |
| 16989636 | -3.54 | 0.044933 | 0.367332 | KIF20A | kinesin family member 20A; NULL |
| 17025152 | -3.56 | 0.000508 | 0.062025 | SERAC1 | serine active site containing 1 |
| 16789149 | -3.58 | 0.002424 | 0.111104 | TNFAIP2 | tumor necrosis factor, alpha-induced protein 2; NULL |
| 16689354 | -3.59 | 0.006926 | 0.174325 | GBP2 | guanylate binding protein 2, interferon-inducible; NULL |
| 16991472 | -3.59 | 0.008322 | 0.188626 | SGCD | sarcoglycan, delta (35kDa dystrophin-associated glycoprotein) |
| 16670574 | -3.6 | 0.000601 | 0.06538 | ECM1 | extracellular matrix protein 1; NULL |
| 17067696 | -3.6 | 0.001877 | 0.098404 | NRG1 | neuregulin 1 |
| 16884956 | -3.62 | 0.012382 | 0.224141 | STEAP3 | STEAP family member 3, metalloreductase; NULL |
| 17089549 | -3.64 | 0.030078 | 0.315502 | DNM1 | dynamin 1 |
| 16808330 | -3.64 | 0.000086 | 0.033506 | PPIP5K1; OTTHUMG00000059903; AC011330.5 | diphosphoinositol pentakisphosphate kinase 1; NULL |
| 16844663 | -3.65 | 0.001199 | 0.082121 | KRT34 | keratin 34 |
| 16818773 | -3.66 | 0.000645 | 0.066494 | ADCY7 | adenylate cyclase 7; NULL |
| 16938654 | -3.66 | 0.000568 | 0.064876 | KRT18P15 | NULL; keratin 18 pseudogene 15 |
| 16836492 | -3.66 | 0.035223 | 0.335712 | PRR11 | proline rich 11 |
| 16839177 | -3.67 | 0.003482 | 0.129909 | METRNL | meteorin, glial cell differentiation regulator-like |
| 16834409 | -3.68 | 0.010675 | 0.210229 | CNTNAP1 | contactin associated protein 1 |
| 16902945 | -3.69 | 0.000108 | 0.038495 | NCKAP5 | NCK-associated protein 5 |
| 16691327 | -3.7 | 0.003338 | 0.128082 | NGF | nerve growth factor (beta polypeptide) |
| 16859314 | -3.72 | 0.000984 | 0.076225 | KLF2 | Kruppel-like factor 2 (lung) |
| 16833567 | -3.76 | 0.019257 | 0.264975 | DUSP14 | dual specificity phosphatase 14 |
| 16859795 | -3.76 | 0.001762 | 0.095788 | GDF15 | growth differentiation factor 15 |
| 17007738 | -3.76 | 0.031585 | 0.32175 | SCUBE3 | signal peptide, CUB domain, EGF-like 3 |
| 16768923 | -3.79 | 0.005476 | 0.159193 | SLC9A7P1 | solute carrier family 9, subfamily A (NHE7, cation proton antiporter 7), member 7 pseudogene 1 |
| 16984730 | -3.8 | 0.037261 | 0.342912 | FST | follistatin |
| 16859763 | -3.81 | 0.001191 | 0.082121 | IFI30; PIK3R2 | interferon, gamma-inducible protein 30; phosphoinositide-3-kinase, regulatory subunit 2 (beta) |
| 16971382 | -3.84 | 0.028777 | 0.310379 | DCLK2 | doublecortin-like kinase 2 |
| 16873296 | -3.86 | 0.013732 | 0.233192 | PPP1R13L | protein phosphatase 1, regulatory subunit 13 like; NULL |
| 16823666 | -3.87 | 0.049192 | 0.376932 | PPL | periplakin; NULL |
| 16680790 | -3.9 | 0.000656 | 0.066659 | MEGF6 | multiple EGF-like-domains 6; NULL |
| 16707196 | -3.92 | 0.001691 | 0.095021 | IFIT1 | interferon-induced protein with tetratricopeptide repeats 1 |
| 16847841 | -3.94 | 0.000008 | 0.015748 | SMURF2 | SMAD specific E3 ubiquitin protein ligase 2; NULL |
| 16907572 | -3.97 | 0.001775 | 0.095868 | GPR1 | G protein-coupled receptor 1; NULL |
| 16693996 | -4.02 | 0.000713 | 0.068672 | THBS3 | thrombospondin 3; NULL |
| 16722081 | -4.03 | 0.003254 | 0.126637 | MICAL2 | microtubule associated monooxygenase, calponin and LIM domain containing 2; NULL |
| 16970404 | -4.06 | 0.009713 | 0.201348 | FGF2 | fibroblast growth factor 2 (basic) |
| 17016263 | -4.12 | 0.000414 | 0.060761 | CMAHP | cytidine monophospho-N-acetylneuraminic acid hydroxylase, pseudogene; NULL |
| 16811085 | -4.12 | 0.007611 | 0.181174 | ITGA11 | integrin, alpha 11 |
| 16768738 | -4.12 | 0.000954 | 0.075559 | NTN4 | netrin 4 |
| 16658536 | -4.13 | 0.0015 | 0.09042 | PER3 | period circadian clock 3 |
| 16818114 | -4.14 | 0.000306 | 0.055103 | HSD3B7 | hydroxy-delta-5-steroid dehydrogenase, 3 beta- and steroid delta-isomerase 7 |
| 17050591 | -4.14 | 0.003482 | 0.129909 | MET | met proto-oncogene (hepatocyte growth factor receptor) |
| 16685201 | -4.15 | 0.001203 | 0.082203 | COL8A2 | collagen, type VIII, alpha 2 |
| 16794719 | -4.16 | 0.00185 | 0.097649 | LTBP2 | latent transforming growth factor beta binding protein 2 |
| 16682333 | -4.17 | 0.000834 | 0.072469 | MFAP2 | microfibrillar-associated protein 2; NULL |
| 17045198 | -4.18 | 0.021974 | 0.277699 | ANLN | anillin, actin binding protein; NULL |
| 17056506 | -4.19 | 0.000025 | 0.020308 | RP9P | retinitis pigmentosa 9 pseudogene |
| 17057478 | -4.25 | 0.002351 | 0.109857 | IGFBP3 | insulin-like growth factor binding protein 3; NULL |
| 16775763 | -4.34 | 0.001696 | 0.095021 | MIR622 | microRNA 622 |
| 17062127 | -4.35 | 0.007568 | 0.180686 | WNT2 | wingless-type MMTV integration site family member 2 |
| 17092870 | -4.37 | 0.002332 | 0.109806 | MIR31HG | MIR31 host gene (non-protein coding) |
| 16746290 | -4.37 | 0.008163 | 0.186578 | OPCML; LOC100653275 | opioid binding protein/cell adhesion molecule-like; NULL; uncharacterized LOC100653275 |
| 16797196 | -4.45 | 0.000001 | 0.005199 | AHNAK2 | AHNAK nucleoprotein 2 |
| 16779546 | -4.62 | 0.000163 | 0.043351 | DIAPH3 | diaphanous homolog 3 (Drosophila) |
| 16962022 | -4.63 | 0.004066 | 0.138108 | LAMP3 | lysosomal-associated membrane protein 3 |
| 16785127 | -4.65 | 0.000008 | 0.01571 | RHOJ | ras homolog family member J |
| 16811249 | -4.66 | 0.000056 | 0.027975 | UACA | uveal autoantigen with coiled-coil domains and ankyrin repeats; NULL |
| 17066224 | -4.67 | 0.003185 | 0.124989 | SH2D4A | SH2 domain containing 4A |
| 17096471 | -4.69 | 0.000073 | 0.030482 | TBC1D2 | TBC1 domain family, member 2 |
| 17000650 | -4.75 | 0.000032 | 0.023178 | TMEM173 | transmembrane protein 173; NULL |
| 16835672 | -4.78 | 0.007507 | 0.179891 | ITGA3 | integrin, alpha 3 (antigen CD49C, alpha 3 subunit of VLA-3 receptor); NULL |
| 16908171 | -4.8 | 0.001059 | 0.078718 | MARCH4 | membrane-associated ring finger (C3HC4) 4, E3 ubiquitin protein ligase |
| 16802903 | -4.82 | 0.000133 | 0.039996 | LOXL1 | lysyl oxidase-like 1 |
| 16841340 | -4.88 | 0.001315 | 0.085936 | MYH2 | myosin, heavy chain 2, skeletal muscle, adult |
| 16675558 | -4.88 | 0.00021 | 0.047629 | NEK7 | NIMA-related kinase 7; NULL |
| 16956792 | -4.89 | 0.000014 | 0.020098 | ABI3BP | ABI family, member 3 (NESH) binding protein |
| 16968213 | -4.93 | 0.003746 | 0.133926 | ANXA3 | annexin A3; NULL |
| 17083370 | -5.05 | 0.002267 | 0.108894 | PDCD1LG2 | programmed cell death 1 ligand 2 |
| 16774427 | -5.08 | 0.003555 | 0.13106 | LACC1; CCDC122 | laccase (multicopper oxidoreductase) domain containing 1; coiled-coil domain containing 122 |
| 16788036 | -5.09 | 0.000251 | 0.051853 | BDKRB1 | bradykinin receptor B1 |
| 16909021 | -5.17 | 0.002416 | 0.110869 | SERPINE2 | serpin peptidase inhibitor, clade E (nexin, plasminogen activator inhibitor type 1), member 2; NULL |
| 16742384 | -5.21 | 0.004629 | 0.147394 | LRRC32 | leucine rich repeat containing 32 |
| 16811638 | -5.21 | 0.004279 | 0.142019 | SEMA7A | semaphorin 7A, GPI membrane anchor (John Milton Hagen blood group) |
| 16920156 | -5.25 | 0.005925 | 0.162689 | PTGIS | prostaglandin I2 (prostacyclin) synthase |
| 17110401 | -5.27 | 0.000005 | 0.012891 | SLC9A7 | solute carrier family 9, subfamily A (NHE7, cation proton antiporter 7), member 7 |
| 16979225 | -5.38 | 0.000588 | 0.064964 | PRSS12 | protease, serine, 12 (neurotrypsin, motopsin) |
| 16979985 | -5.41 | 0.000162 | 0.043351 | MGARP; NDUFC1 | mitochondria-localized glutamic acid-rich protein; NADH dehydrogenase (ubiquinone) 1, subcomplex unknown, 1, 6kDa |
| 17005138 | -5.42 | 0.000213 | 0.047666 | CAP2 | CAP, adenylate cyclase-associated protein, 2 (yeast) |
| 16784760 | -5.43 | 0.036514 | 0.340794 | DACT1 | dishevelled-binding antagonist of beta-catenin 1 |
| 16874005 | -5.49 | 0.003178 | 0.124894 | DBP | D site of albumin promoter (albumin D-box) binding protein |
| 16997802 | -5.5 | 0.041054 | 0.354928 | HAPLN1 | hyaluronan and proteoglycan link protein 1; NULL |
| 17001299 | -5.53 | 0.000476 | 0.061367 | DPYSL3 | dihydropyrimidinase-like 3; NULL |
| 17063221 | -5.53 | 0.039346 | 0.349047 | FAM180A | family with sequence similarity 180, member A |
| 16950440 | -5.62 | 0.015553 | 0.243466 | OXTR | oxytocin receptor |
| 16687123 | -5.72 | 0.000971 | 0.076112 | RAB3B | RAB3B, member RAS oncogene family |
| 17017018 | -5.91 | 0.017512 | 0.255571 | IER3 | immediate early response 3; NULL |
| 16715793 | -5.96 | 0.000077 | 0.031569 | KCNMA1 | potassium large conductance calcium-activated channel, subfamily M, alpha member 1; NULL |
| 16697196 | -6.05 | 0.001057 | 0.078718 | FAM129A | family with sequence similarity 129, member A |
| 17006949 | -6.08 | 0.024935 | 0.292873 | CFB; OTTHUMG00000159600; XXbac-BPG116M5.17; C2 | complement factor B; NULL |
| 17007982 | -6.21 | 0.007382 | 0.179276 | PI16 | peptidase inhibitor 16 |
| 17049904 | -6.59 | 0.000336 | 0.058169 | LRRC17 | leucine rich repeat containing 17 |
| 16716478 | -6.64 | 0.000976 | 0.076144 | ANKRD1 | ankyrin repeat domain 1 (cardiac muscle) |
| 16848173 | -6.65 | 0.019313 | 0.265352 | ABCA9 | ATP-binding cassette, sub-family A (ABC1), member 9; NULL |
| 16824572 | -6.67 | 0.036743 | 0.341571 | GPRC5B | G protein-coupled receptor, family C, group 5, member B; NULL |
| 16914972 | -6.73 | 0.000014 | 0.020098 | DOK5 | docking protein 5 |
| 16852858 | -6.73 | 0.01177 | 0.219333 | SERPINB7 | serpin peptidase inhibitor, clade B (ovalbumin), member 7 |
| 16904324 | -6.74 | 0.000009 | 0.016227 | FAP | fibroblast activation protein, alpha; NULL |
| 16967631 | -6.79 | 0.001225 | 0.082663 | SLC4A4 | solute carrier family 4, sodium bicarbonate cotransporter, member 4 |
| 16767422 | -6.9 | 0.003002 | 0.122701 | PTPRB | protein tyrosine phosphatase, receptor type, B; NULL |
| 16717272 | -7.16 | 0.000574 | 0.064876 | LOXL4 | lysyl oxidase-like 4 |
| 16773131 | -7.3 | 0.047207 | 0.371926 | SGCG | sarcoglycan, gamma (35kDa dystrophin-associated glycoprotein) |
| 17014257 | -7.75 | 0.005171 | 0.154996 | FNDC1 | fibronectin type III domain containing 1 |
| 17017641 | -7.84 | 0.007438 | 0.179648 | TNXB; TNXA; LOC101060681 | tenascin XB; tenascin XA (pseudogene); tenascin-X-like; NULL |
| 17108816 | -7.96 | 0.000506 | 0.062025 | MXRA5 | matrix-remodelling associated 5 |
| 17087413 | -8.11 | 0.000056 | 0.027975 | GALNT12 | UDP-N-acetyl-alpha-D-galactosamine:polypeptide N-acetylgalactosaminyltransferase 12 (GalNAc-T12) |
| 17087430 | -8.27 | 0.003027 | 0.123389 | COL15A1 | collagen, type XV, alpha 1 |
| 16697471 | -8.3 | 0.000619 | 0.065415 | B3GALT2 | UDP-Gal:betaGlcNAc beta 1,3-galactosyltransferase, polypeptide 2 |
| 16974968 | -8.34 | 0.000493 | 0.061679 | SEL1L3 | sel-1 suppressor of lin-12-like 3 (C. elegans); NULL |
| 16755908 | -8.36 | 0.000276 | 0.053107 | DRAM1 | DNA-damage regulated autophagy modulator 1 |
| 17115996 | -8.37 | 0.000095 | 0.036044 | KRT18P10 | NULL; keratin 18 pseudogene 10 |
| 16855510 | -8.49 | 0.00016 | 0.043351 | ATP8B1 | ATPase, aminophospholipid transporter, class I, type 8B, member 1 |
| 16858137 | -8.56 | 0.001772 | 0.095821 | ICAM1 | intercellular adhesion molecule 1 |
| 17059355 | -9.55 | 0.048693 | 0.375621 | SEMA3D | sema domain, immunoglobulin domain (Ig), short basic domain, secreted, (semaphorin) 3D |
| 16737344 | -9.9 | 0.013863 | 0.234579 | PAMR1 | peptidase domain containing associated with muscle regeneration 1 |
| 17050765 | -10.79 | 0.00129 | 0.085317 | KCND2 | potassium voltage-gated channel, Shal-related subfamily, member 2 |
| 16759218 | -10.87 | 0.00023 | 0.049353 | GPR133 | G protein-coupled receptor 133; NULL |
| 17072162 | -10.99 | 0.011752 | 0.219333 | COL14A1 | collagen, type XIV, alpha 1; NULL |
| 16962911 | -12.16 | 0.002301 | 0.109466 | LRRC15 | leucine rich repeat containing 15 |
| 17072135 | -12.73 | 0.01297 | 0.227463 | NOV | nephroblastoma overexpressed |
| 17044177 | -12.77 | 0.037098 | 0.342685 | IL6 | interleukin 6 (interferon, beta 2); NULL |
| 16904278 | -13.14 | 0.000164 | 0.043351 | DPP4 | dipeptidyl-peptidase 4; NULL |
| 17084130 | -13.49 | 0.000384 | 0.059593 | TEK | TEK tyrosine kinase, endothelial |
| 16904667 | -15.8 | 0.004755 | 0.14805 | SCN9A | sodium channel, voltage-gated, type IX, alpha subunit; NULL |
| 17080486 | -25.38 | 0.000024 | 0.020308 | TNFRSF11B | tumor necrosis factor receptor superfamily, member 11b |
| 17095887 | -26.25 | 5.36E-07 | 0.005199 | ASPN | asporin |
| 16803754 | -100.71 | 0.000285 | 0.053726 | KIAA1199 | KIAA1199; NULL |
