# Supplementary material for: Identification of transcription factors potentially involved in human adipogenesis in vitro
Source: Mol Genet Genomic Med. 2017 Mar 3;5(3):210–22. doi: 10.1002/mgg3.269 (PMC5441431; doi:10.1002/mgg3.269)
Supplement: Supplementary file 3 — Table S3. A complete list of differentially expressed genes on day 14. [file MGG3-5-210-s003.doc]

|  |  |  |  |  |  |
| --- | --- | --- | --- | --- | --- |
| **Up-regulated genes on day 14** | | |  |  |  |
| Transcript Cluster ID | Fold Change (linear) (Induced vs. Control) | ANOVA p-value (Induced vs. Control) | FDR p-value (Induced vs. Control) | Gene Symbol | Description |
| 17078592 | 659.49 | 1.95E-07 | 0.000942 | FABP4 | fatty acid binding protein 4, adipocyte; NULL |
| 16949397 | 341.67 | 1.95E-07 | 0.000942 | ADIPOQ | adiponectin, C1Q and collagen domain containing |
| 17021510 | 195.39 | 1.16E-07 | 0.00091 | CNR1 | cannabinoid receptor 1 (brain); NULL |
| 16729472 | 192.13 | 0.000008 | 0.007669 | THRSP | thyroid hormone responsive |
| 17059955 | 149.37 | 6.85E-08 | 0.00091 | PDK4 | pyruvate dehydrogenase kinase, isozyme 4 |
| 16722299 | 78.36 | 0.000007 | 0.007536 | PDE3B | phosphodiesterase 3B, cGMP-inhibited; NULL |
| 16779958 | 76.34 | 0.000007 | 0.007334 | EDNRB | endothelin receptor type B |
| 16813173 | 69.31 | 0.000002 | 0.003633 | PLIN1 | perilipin 1 |
| 16950609 | 63.41 | 0.000001 | 0.0032 | CIDEC | cell death-inducing DFFA-like effector c |
| 17066278 | 60.21 | 0.000008 | 0.007669 | LPL | lipoprotein lipase; NULL |
| 16834516 | 57.15 | 0.000001 | 0.0032 | AOC3 | amine oxidase, copper containing 3 |
| 16763182 | 56.74 | 7.69E-09 | 0.000371 | ABCD2 | ATP-binding cassette, sub-family D (ALD), member 2 |
| 17102829 | 47.4 | 0.000001 | 0.0032 | MAOA | monoamine oxidase A |
| 16774303 | 45.9 | 0.000097 | 0.028085 | RGCC | regulator of cell cycle |
| 16756649 | 45.1 | 0.000025 | 0.014302 | ACACB | acetyl-CoA carboxylase beta; NULL |
| 16726065 | 41.57 | 0.000141 | 0.032437 | LGALS12 | lectin, galactoside-binding, soluble, 12 |
| 16716659 | 40.15 | 0.000004 | 0.006116 | RBP4 | retinol binding protein 4, plasma |
| 17050154 | 35.03 | 0.000037 | 0.017356 | PRKAR2B | protein kinase, cAMP-dependent, regulatory, type II, beta |
| 16821541 | 33.56 | 0.000015 | 0.011346 | CRISPLD2 | cysteine-rich secretory protein LCCL domain containing 2 |
| 16667530 | 30.83 | 0.000031 | 0.016183 | PALMD | palmdelphin |
| 16751048 | 29.1 | 0.000003 | 0.004912 | GPD1 | glycerol-3-phosphate dehydrogenase 1 (soluble); NULL |
| 16706630 | 27.43 | 0.000002 | 0.003712 | FAM213A | family with sequence similarity 213, member A |
| 16767335 | 26.6 | 0.001326 | 0.082528 | CPM | carboxypeptidase M; NULL |
| 17075589 | 26 | 0.000065 | 0.023565 | NEFL | neurofilament, light polypeptide |
| 16676988 | 25.73 | 0.000262 | 0.043363 | HSD11B1 | hydroxysteroid (11-beta) dehydrogenase 1 |
| 16850958 | 24.92 | 0.000012 | 0.009812 | APCDD1 | adenomatosis polyposis coli down-regulated 1 |
| 16967875 | 23.86 | 0.002123 | 0.10251 | PARM1 | prostate androgen-regulated mucin-like protein 1 |
| 16883690 | 23.26 | 0.000023 | 0.013682 | IL1RL1 | interleukin 1 receptor-like 1; NULL |
| 17061759 | 21.5 | 0.000134 | 0.032038 | NRCAM | neuronal cell adhesion molecule; NULL |
| 16761938 | 20.53 | 2.56E-07 | 0.001123 | LMO3 | LIM domain only 3 (rhombotin-like 2); NULL |
| 17113346 | 19.28 | 0.001594 | 0.089727 | CHRDL1 | chordin-like 1; NULL |
| 16867326 | 17.7 | 0.000001 | 0.0032 | PLIN4 | perilipin 4 |
| 16718414 | 17.15 | 0.000063 | 0.023348 | GPAM | glycerol-3-phosphate acyltransferase, mitochondrial; NULL |
| 16819264 | 16.69 | 8.91E-08 | 0.00091 | MT1X | metallothionein 1X |
| 16872783 | 16.02 | 0.000004 | 0.005188 | LIPE | lipase, hormone-sensitive |
| 16950825 | 14.04 | 0.000038 | 0.017393 | TIMP4 | TIMP metallopeptidase inhibitor 4 |
| 16780929 | 13.89 | 0.027706 | 0.298397 | COL4A1 | collagen, type IV, alpha 1 |
| 16998551 | 13.51 | 0.002682 | 0.112646 | SLCO4C1 | solute carrier organic anion transporter family, member 4C1 |
| 16769250 | 13.47 | 0.003331 | 0.120505 | IGF1 | insulin-like growth factor 1 (somatomedin C) |
| 16671187 | 12.93 | 0.000917 | 0.073359 | NPR1 | natriuretic peptide receptor A/guanylate cyclase A (atrionatriuretic peptide receptor A) |
| 16739733 | 12.75 | 0.00087 | 0.071393 | HRASLS5 | HRAS-like suppressor family, member 5 |
| 16909257 | 11.83 | 0.000005 | 0.006299 | SLC19A3 | solute carrier family 19, member 3; NULL |
| 17093227 | 11.16 | 0.000016 | 0.011364 | AQP7; LOC100509620 | aquaporin 7; aquaporin-7-like; NULL |
| 16841907 | 11.07 | 0.000042 | 0.018601 | RASD1 | RAS, dexamethasone-induced 1 |
| 16978236 | 11.04 | 0.00161 | 0.090369 | ADH1B | alcohol dehydrogenase 1B (class I), beta polypeptide |
| 16981099 | 10.46 | 0.006433 | 0.16372 | NPY1R | neuropeptide Y receptor Y1; NULL |
| 16982047 | 10.17 | 0.000019 | 0.012528 | ACSL1 | acyl-CoA synthetase long-chain family member 1; NULL |
| 16966127 | 10.06 | 0.000217 | 0.039858 | KLB | klotho beta |
| 16709268 | 8.77 | 0.002795 | 0.114291 | ACSL5 | acyl-CoA synthetase long-chain family member 5; NULL |
| 16915182 | 8.63 | 0.000198 | 0.038099 | PCK1 | phosphoenolpyruvate carboxykinase 1 (soluble); NULL |
| 16938133 | 8.25 | 0.006048 | 0.159828 | GALNT15 | UDP-N-acetyl-alpha-D-galactosamine:polypeptide N-acetylgalactosaminyltransferase 15 |
| 17063005 | 7.77 | 0.001075 | 0.076267 | PLXNA4 | plexin A4 |
| 16819224 | 7.74 | 0.000624 | 0.064195 | MT1M | metallothionein 1M |
| 16999041 | 7.66 | 0.000439 | 0.054061 | CDO1 | cysteine dioxygenase type 1 |
| 16829521 | 7.65 | 0.000011 | 0.009196 | TUSC5 | tumor suppressor candidate 5 |
| 16676983 | 7.64 | 0.00032 | 0.04744 | G0S2 | G0/G1switch 2 |
| 16955822 | 7.32 | 0.000059 | 0.022513 | ADAMTS9 | ADAM metallopeptidase with thrombospondin type 1 motif, 9; NULL |
| 16850107 | 7.23 | 0.000058 | 0.022513 | FASN | fatty acid synthase |
| 16826738 | 7.18 | 0.001064 | 0.075909 | MT1G | metallothionein 1G |
| 16761820 | 6.98 | 0.002193 | 0.104314 | MGP | matrix Gla protein |
| 16819252 | 6.94 | 0.005555 | 0.156381 | MT1F | metallothionein 1F; NULL |
| 16871235 | 6.88 | 0.000062 | 0.023091 | CEBPA | CCAAT/enhancer binding protein (C/EBP), alpha |
| 16743735 | 6.84 | 0.013072 | 0.219415 | MMP3 | matrix metallopeptidase 3 (stromelysin 1, progelatinase); NULL |
| 16700888 | 6.64 | 0.000332 | 0.048308 | NID1 | nidogen 1 |
| 16763577 | 6.54 | 0.001998 | 0.099341 | SLC38A4 | solute carrier family 38, member 4 |
| 16851309 | 6.49 | 0.000025 | 0.014302 | GREB1L | growth regulation by estrogen in breast cancer-like; NULL |
| 16834508 | 6.48 | 0.003238 | 0.1192 | AOC2 | amine oxidase, copper containing 2 (retina-specific) |
| 16846864 | 6.44 | 0.000786 | 0.068399 | MMD | monocyte to macrophage differentiation-associated |
| 16977378 | 6.41 | 0.000711 | 0.065717 | TMEM150C | transmembrane protein 150C |
| 17080648 | 6.39 | 0.026689 | 0.292854 | HAS2 | hyaluronan synthase 2 |
| 17114288 | 6.28 | 0.000127 | 0.031928 | GPC3 | glypican 3 |
| 16826639 | 6.21 | 0.001321 | 0.082528 | CES1; LOC100653057 | carboxylesterase 1; liver carboxylesterase 1-like; NULL |
| 16912975 | 6.2 | 0.000098 | 0.028085 | ACSS2 | acyl-CoA synthetase short-chain family member 2; NULL |
| 17075973 | 6.18 | 0.000006 | 0.007109 | DUSP4 | dual specificity phosphatase 4 |
| 16751190 | 6.14 | 0.003145 | 0.118753 | METTL7A | methyltransferase like 7A; NULL |
| 16958638 | 6.07 | 0.000096 | 0.028085 | KLF15 | Kruppel-like factor 15 |
| 16870453 | 5.98 | 0.020908 | 0.266392 | COMP | cartilage oligomeric matrix protein |
| 16765625 | 5.88 | 0.000333 | 0.048308 | PPP1R1A | protein phosphatase 1, regulatory (inhibitor) subunit 1A |
| 16720353 | 5.83 | 0.000208 | 0.039109 | PNPLA2 | patatin-like phospholipase domain containing 2; NULL |
| 16955197 | 5.8 | 0.001879 | 0.096922 | WNT5A | wingless-type MMTV integration site family, member 5A |
| 16868481 | 5.69 | 0.000777 | 0.067914 | OLFM2 | olfactomedin 2; NULL |
| 16796375 | 5.63 | 0.000343 | 0.04876 | CLMN | calmin (calponin-like, transmembrane); NULL |
| 16716795 | 5.62 | 0.000968 | 0.074483 | SORBS1; KIAA0894; RP11-476E15.3 | sorbin and SH3 domain containing 1; NULL |
| 17047795 | 5.6 | 0.012049 | 0.211809 | CD36 | CD36 molecule (thrombospondin receptor); NULL |
| 16689869 | 5.57 | 0.000697 | 0.065717 | F3 | coagulation factor III (thromboplastin, tissue factor) |
| 16776431 | 5.42 | 0.023864 | 0.281726 | COL4A2 | collagen, type IV, alpha 2; NULL |
| 16937741 | 5.34 | 0.000338 | 0.048453 | PPARG | peroxisome proliferator-activated receptor gamma; NULL |
| 16984304 | 5.3 | 0.002431 | 0.109467 | C7 | complement component 7; NULL |
| 16731441 | 5.29 | 0.000005 | 0.006286 | ZBTB16 | zinc finger and BTB domain containing 16; NULL |
| 16711343 | 5.23 | 0.006967 | 0.169028 | AKR1C2; LOC101060798 | aldo-keto reductase family 1, member C2; aldo-keto reductase family 1 member C2-like; NULL |
| 16919022 | 5.21 | 0.00157 | 0.089367 | SAMHD1 | SAM domain and HD domain 1 |
| 17095056 | 5.19 | 0.001991 | 0.099284 | PRUNE2 | prune homolog 2 (Drosophila); NULL |
| 16991208 | 5.03 | 0.000316 | 0.047138 | GPX3 | glutathione peroxidase 3 (plasma) |
| 17080082 | 5.01 | 0.001505 | 0.087335 | ANGPT1 | angiopoietin 1 |
| 16870443 | 5.01 | 0.001552 | 0.088776 | CRLF1 | cytokine receptor-like factor 1 |
| 16906285 | 4.95 | 0.001591 | 0.089691 | CALCRL | calcitonin receptor-like; NULL |
| 16778392 | 4.94 | 0.000003 | 0.004912 | FOXO1 | forkhead box O1 |
| 16933140 | 4.93 | 0.00682 | 0.167542 | GGT5 | gamma-glutamyltransferase 5 |
| 16696177 | 4.92 | 0.000112 | 0.029831 | SLC19A2 | solute carrier family 19 (thiamine transporter), member 2 |
| 16994597 | 4.84 | 0.013943 | 0.225941 | FAM134B | family with sequence similarity 134, member B |
| 16837348 | 4.84 | 0.012303 | 0.213731 | MAP2K6 | mitogen-activated protein kinase kinase 6 |
| 17077723 | 4.78 | 0.033907 | 0.321282 | CYP7B1 | cytochrome P450, family 7, subfamily B, polypeptide 1 |
| 16991192 | 4.77 | 0.005631 | 0.157222 | GPX3 | glutathione peroxidase 3 (plasma); NULL |
| 16771602 | 4.75 | 0.00037 | 0.049949 | HPD | 4-hydroxyphenylpyruvate dioxygenase |
| 16999475 | 4.72 | 0.002099 | 0.102409 | FBN2 | fibrillin 2 |
| 16745281 | 4.72 | 0.005494 | 0.155942 | MCAM | melanoma cell adhesion molecule; NULL |
| 17048072 | 4.71 | 0.001107 | 0.077239 | STEAP1 | six transmembrane epithelial antigen of the prostate 1 |
| 16733516 | 4.66 | 0.00685 | 0.167743 | ADAMTS15 | ADAM metallopeptidase with thrombospondin type 1 motif, 15 |
| 17085951 | 4.61 | 0.019743 | 0.261754 | RORB | RAR-related orphan receptor B |
| 16931000 | 4.6 | 0.002126 | 0.10251 | PNPLA3 | patatin-like phospholipase domain containing 3 |
| 17018497 | 4.59 | 6.17E-07 | 0.002126 | FKBP5; LOC285847 | FK506 binding protein 5; uncharacterized LOC285847 |
| 17014798 | 4.59 | 0.004317 | 0.137774 | SMOC2 | SPARC related modular calcium binding 2 |
| 16835816 | 4.58 | 0.000428 | 0.053582 | ACSF2 | acyl-CoA synthetase family member 2; NULL |
| 16819233 | 4.58 | 0.02409 | 0.282973 | MT1A | metallothionein 1A |
| 16696979 | 4.57 | 0.000655 | 0.064897 | GLUL | glutamate-ammonia ligase; NULL |
| 16695627 | 4.52 | 0.006566 | 0.164497 | ADAMTS4 | ADAM metallopeptidase with thrombospondin type 1 motif, 4 |
| 16696120 | 4.47 | 0.000794 | 0.068667 | DPT | dermatopontin |
| 16997393 | 4.45 | 0.00553 | 0.155987 | ZBED3 | zinc finger, BED-type containing 3 |
| 16696811 | 4.33 | 0.002185 | 0.104226 | ANGPTL1 | angiopoietin-like 1 |
| 17093463 | 4.33 | 0.000237 | 0.042023 | CNTFR | ciliary neurotrophic factor receptor |
| 16785379 | 4.32 | 0.008981 | 0.187293 | HSPA2 | heat shock 70kDa protein 2 |
| 16729168 | 4.31 | 0.000263 | 0.043363 | DGAT2 | diacylglycerol O-acyltransferase 2 |
| 17058524 | 4.3 | 0.002316 | 0.1072 | MLXIPL | MLX interacting protein-like; NULL |
| 16836021 | 4.29 | 0.012142 | 0.212476 | ABCC3 | ATP-binding cassette, sub-family C (CFTR/MRP), member 3; NULL |
| 17058142 | 4.25 | 0.011082 | 0.205076 | ZNF117 | zinc finger protein 117 |
| 17021217 | 4.24 | 0.001305 | 0.08213 | ME1 | malic enzyme 1, NADP(+)-dependent, cytosolic |
| 16704320 | 4.22 | 0.000832 | 0.069929 | RASSF4 | Ras association (RalGDS/AF-6) domain family member 4; NULL |
| 16975671 | 4.18 | 0.019929 | 0.262503 | CORIN | corin, serine peptidase; NULL |
| 17101231 | 4.17 | 0.001636 | 0.090781 | GYG2 | glycogenin 2 |
| 16883715 | 4.16 | 0.000584 | 0.062016 | IL18R1 | interleukin 18 receptor 1 |
| 16966733 | 4.12 | 0.003832 | 0.129334 | RASL11B | RAS-like, family 11, member B |
| 16896561 | 4.11 | 0.000119 | 0.031253 | CYP1B1 | cytochrome P450, family 1, subfamily B, polypeptide 1 |
| 16997399 | 4.11 | 0.000555 | 0.060129 | SNORA47 | small nucleolar RNA, H/ACA box 47 |
| 16856299 | 4.1 | 0.011827 | 0.210727 | CFD | complement factor D (adipsin) |
| 17012447 | 4.07 | 0.000199 | 0.038161 | LAMA2 | laminin, alpha 2 |
| 16779667 | 4.06 | 0.005478 | 0.155708 | PCDH9 | protocadherin 9 |
| 16722278 | 4.04 | 0.022691 | 0.27586 | SPON1 | spondin 1, extracellular matrix protein |
| 17113147 | 4 | 0.000032 | 0.016319 | TSC22D3 | TSC22 domain family, member 3; NULL |
| 16947148 | 3.96 | 0.000003 | 0.004912 | ARHGEF26 | Rho guanine nucleotide exchange factor (GEF) 26 |
| 16850069 | 3.95 | 0.000021 | 0.013319 | DCXR | dicarbonyl/L-xylulose reductase; NULL |
| 16912362 | 3.95 | 0.034681 | 0.324319 | ID1 | inhibitor of DNA binding 1, dominant negative helix-loop-helix protein |
| 16879385 | 3.95 | 0.000221 | 0.040124 | PKDCC | protein kinase domain containing, cytoplasmic; NULL |
| 16743091 | 3.93 | 0.000078 | 0.026305 | FZD4 | frizzled family receptor 4 |
| 16938630 | 3.91 | 1.19E-07 | 0.00091 | GPD1L | glycerol-3-phosphate dehydrogenase 1-like; NULL |
| 16731169 | 3.9 | 0.000521 | 0.05862 | DLAT | dihydrolipoamide S-acetyltransferase; NULL |
| 16842103 | 3.9 | 0.00216 | 0.103535 | SHMT1 | serine hydroxymethyltransferase 1 (soluble); NULL |
| 17063461 | 3.86 | 0.001931 | 0.098216 | HIPK2 | homeodomain interacting protein kinase 2 |
| 16840846 | 3.86 | 0.00024 | 0.042023 | PER1 | period circadian clock 1 |
| 16769761 | 3.83 | 0.000303 | 0.046378 | TMEM119 | transmembrane protein 119 |
| 17058152 | 3.82 | 0.031103 | 0.309513 | ERV3-1; ZNF117 | endogenous retrovirus group 3, member 1; zinc finger protein 117 |
| 16985518 | 3.81 | 0.000199 | 0.038161 | PIK3R1 | phosphoinositide-3-kinase, regulatory subunit 1 (alpha); NULL |
| 16729290 | 3.8 | 0.000047 | 0.019277 | TSKU | tsukushi, small leucine rich proteoglycan |
| 16725783 | 3.77 | 0.005119 | 0.150434 | BEST1 | bestrophin 1 |
| 16832429 | 3.73 | 0.004609 | 0.142821 | TMEM97 | transmembrane protein 97 |
| 16819152 | 3.7 | 0.005763 | 0.158401 | CES1; LOC100653057; LOC100653086; CES1P1 | carboxylesterase 1; liver carboxylesterase 1-like; uncharacterized LOC100653086; carboxylesterase 1 pseudogene 1; NULL |
| 16708249 | 3.7 | 0.000162 | 0.034726 | SCD | stearoyl-CoA desaturase (delta-9-desaturase) |
| 16889268 | 3.69 | 0.00202 | 0.099802 | AOX1 | aldehyde oxidase 1; NULL |
| 16666965 | 3.65 | 0.000612 | 0.063216 | LRRC8B | leucine rich repeat containing 8 family, member B |
| 16885135 | 3.62 | 0.005424 | 0.154884 | INHBB | inhibin, beta B |
| 16800242 | 3.6 | 0.007232 | 0.170944 | CKMT1B; CKMT1A | creatine kinase, mitochondrial 1B; creatine kinase, mitochondrial 1A; NULL |
| 16786255 | 3.58 | 0.001471 | 0.086859 | ACOT2 | acyl-CoA thioesterase 2 |
| 16701975 | 3.55 | 0.000116 | 0.030665 | AKR1C1 | aldo-keto reductase family 1, member C1; NULL |
| 16739435 | 3.54 | 0.003068 | 0.11741 | C11orf48 | chromosome 11 open reading frame 48 |
| 17051159 | 3.51 | 0.000962 | 0.074302 | HILPDA | hypoxia inducible lipid droplet-associated |
| 17048563 | 3.51 | 0.004203 | 0.135858 | PEG10 | paternally expressed 10 |
| 16729812 | 3.48 | 0.000994 | 0.074654 | TMEM135 | transmembrane protein 135; NULL |
| 16765192 | 3.47 | 0.001554 | 0.088776 | CSAD | cysteine sulfinic acid decarboxylase; NULL |
| 16995890 | 3.47 | 0.003649 | 0.126615 | HMGCS1 | 3-hydroxy-3-methylglutaryl-CoA synthase 1 (soluble); NULL |
| 16690566 | 3.45 | 0.007233 | 0.170944 | SORT1 | sortilin 1; NULL |
| 16760668 | 3.43 | 0.000558 | 0.060129 | LPCAT3 | lysophosphatidylcholine acyltransferase 3; NULL |
| 16899634 | 3.42 | 0.000154 | 0.03427 | RETSAT | retinol saturase (all-trans-retinol 13,14-reductase) |
| 16859840 | 3.4 | 0.00253 | 0.110802 | TMEM59L; SYNGR1 | transmembrane protein 59-like; synaptogyrin 1; NULL |
| 16678114 | 3.38 | 0.001486 | 0.086954 | EPHX1 | epoxide hydrolase 1, microsomal (xenobiotic) |
| 16857886 | 3.37 | 0.000105 | 0.02876 | ANGPTL4 | angiopoietin-like 4; NULL |
| 16834436 | 3.37 | 0.024955 | 0.286611 | RAMP2 | receptor (G protein-coupled) activity modifying protein 2 |
| 16806564 | 3.36 | 0.000481 | 0.055896 | MTMR10 | myotubularin related protein 10; NULL |
| 16909303 | 3.32 | 0.001057 | 0.075909 | PID1 | phosphotyrosine interaction domain containing 1 |
| 16687618 | 3.31 | 0.003554 | 0.12489 | DHCR24 | 24-dehydrocholesterol reductase |
| 16790744 | 3.31 | 0.042868 | 0.355902 | SLC7A8 | solute carrier family 7 (amino acid transporter light chain, L system), member 8; NULL |
| 16702175 | 3.29 | 0.004153 | 0.135309 | PFKFB3 | 6-phosphofructo-2-kinase/fructose-2,6-biphosphatase 3; NULL |
| 16713318 | 3.28 | 0.017134 | 0.246354 | NAMPT; NAMPTL | nicotinamide phosphoribosyltransferase; nicotinamide phosphoribosyltransferase-like |
| 16919962 | 3.26 | 0.001735 | 0.092634 | SULF2 | sulfatase 2; NULL |
| 16969591 | 3.25 | 0.002912 | 0.11624 | HADH | hydroxyacyl-CoA dehydrogenase; NULL |
| 16844936 | 3.23 | 0.000253 | 0.042689 | ACLY | ATP citrate lyase; NULL |
| 16765697 | 3.22 | 0.001486 | 0.086954 | ITGA7 | integrin, alpha 7; NULL |
| 16913537 | 3.21 | 0.000779 | 0.067914 | LBP | lipopolysaccharide binding protein |
| 17014309 | 3.2 | 0.002175 | 0.10383 | ACAT2; LOC100129518; SOD2 | acetyl-CoA acetyltransferase 2; uncharacterized LOC100129518; superoxide dismutase 2, mitochondrial |
| 16739752 | 3.2 | 0.007568 | 0.174846 | PLA2G16 | phospholipase A2, group XVI |
| 17016089 | 3.19 | 0.024496 | 0.28438 | PRL | prolactin |
| 16766946 | 3.17 | 0.031365 | 0.310727 | AVPR1A | arginine vasopressin receptor 1A |
| 16995771 | 3.17 | 0.002782 | 0.114291 | C6 | complement component 6; NULL |
| 16705961 | 3.17 | 0.006599 | 0.164645 | DDIT4 | DNA-damage-inducible transcript 4 |
| 16836311 | 3.16 | 0.002954 | 0.116744 | NOG | noggin |
| 17107907 | 3.16 | 0.000402 | 0.051776 | NSDHL | NAD(P) dependent steroid dehydrogenase-like; NULL |
| 17022623 | 3.16 | 0.002406 | 0.108966 | REV3L | REV3-like, polymerase (DNA directed), zeta, catalytic subunit; NULL |
| 17096728 | 3.15 | 0.000187 | 0.036889 | ABCA1 | ATP-binding cassette, sub-family A (ABC1), member 1 |
| 16716782 | 3.14 | 0.0108 | 0.202499 | PDLIM1 | PDZ and LIM domain 1 |
| 17047459 | 3.14 | 0.001232 | 0.07992 | SNORA14A | small nucleolar RNA, H/ACA box 14A |
| 16822035 | 3.11 | 0.000758 | 0.067703 | DPEP1 | dipeptidase 1 (renal); NULL |
| 17061662 | 3.08 | 0.000286 | 0.045051 | LAMB1 | laminin, beta 1 |
| 16903953 | 3.07 | 0.00825 | 0.182613 | ACVR1C | activin A receptor, type IC |
| 17001846 | 3.07 | 0.002402 | 0.108938 | CCDC69 | coiled-coil domain containing 69; NULL |
| 16820398 | 3.07 | 0.000775 | 0.067914 | SLC7A6; SLC7A6OS | solute carrier family 7 (amino acid transporter light chain, y+L system), member 6; solute carrier family 7, member 6 opposite strand; NULL |
| 17020152 | 3.03 | 0.000505 | 0.057561 | ELOVL5 | ELOVL fatty acid elongase 5; NULL |
| 16953597 | 3.03 | 0.000272 | 0.043888 | SLC26A6 | solute carrier family 26, member 6; NULL |
| 17092712 | 3.01 | 0.00237 | 0.108556 | PLIN2; LOC100509484 | perilipin 2; uncharacterized LOC100509484 |
| 16966721 | 3.01 | 0.00301 | 0.116744 | SNORA26 | small nucleolar RNA, H/ACA box 26 |
| 17003640 | 3 | 0.010545 | 0.200455 | ADAMTS2 | ADAM metallopeptidase with thrombospondin type 1 motif, 2 |
| 16773453 | 3 | 0.000183 | 0.036754 | WASF3 | WAS protein family, member 3 |
| 16855973 | 2.99 | 0.002997 | 0.116744 | CYB5A | cytochrome b5 type A (microsomal) |
| 17060049 | 2.99 | 0.000342 | 0.04876 | DLX5 | distal-less homeobox 5 |
| 16936761 | 2.98 | 0.044158 | 0.359151 | CHL1 | cell adhesion molecule with homology to L1CAM (close homolog of L1); NULL |
| 16978995 | 2.98 | 0.000106 | 0.028936 | ELOVL6 | ELOVL fatty acid elongase 6; NULL |
| 16711598 | 2.97 | 0.002349 | 0.108191 | ITIH5 | inter-alpha-trypsin inhibitor heavy chain family, member 5; NULL |
| 16658864 | 2.96 | 0.001402 | 0.084773 | PGD | phosphogluconate dehydrogenase; NULL |
| 16863115 | 2.95 | 0.005573 | 0.156381 | APOE; HMGA1 | apolipoprotein E; high mobility group AT-hook 1 |
| 17094459 | 2.95 | 0.006487 | 0.163944 | AQP7P1 | aquaporin 7 pseudogene 1 |
| 17014442 | 2.95 | 0.004358 | 0.138459 | SLC22A3 | solute carrier family 22 (extraneuronal monoamine transporter), member 3 |
| 16979917 | 2.95 | 0.017216 | 0.246731 | SLC7A11 | solute carrier family 7 (anionic amino acid transporter light chain, xc- system), member 11 |
| 16958573 | 2.94 | 0.010515 | 0.200035 | ALDH1L1 | aldehyde dehydrogenase 1 family, member L1; NULL |
| 16741969 | 2.94 | 0.000706 | 0.065717 | UCP2 | uncoupling protein 2 (mitochondrial, proton carrier); NULL |
| 16913441 | 2.93 | 0.027989 | 0.299608 | NNAT | neuronatin |
| 16724485 | 2.93 | 0.003019 | 0.116744 | NR1H3 | nuclear receptor subfamily 1, group H, member 3; NULL |
| 16723546 | 2.91 | 0.001096 | 0.076986 | CAT | catalase |
| 17087790 | 2.91 | 0.011429 | 0.207135 | SLC44A1 | solute carrier family 44, member 1 |
| 16911632 | 2.9 | 0.011141 | 0.205267 | PCSK2 | proprotein convertase subtilisin/kexin type 2 |
| 17102129 | 2.9 | 0.001246 | 0.07992 | SAT1 | spermidine/spermine N1-acetyltransferase 1; NULL |
| 17110071 | 2.89 | 0.048114 | 0.37131 | SRPX | sushi-repeat containing protein, X-linked |
| 16773493 | 2.88 | 0.007136 | 0.170634 | RASL11A | RAS-like, family 11, member A |
| 16758885 | 2.84 | 0.000026 | 0.014748 | AACS | acetoacetyl-CoA synthetase |
| 16757160 | 2.84 | 0.001856 | 0.096135 | ALDH2 | aldehyde dehydrogenase 2 family (mitochondrial) |
| 16947173 | 2.81 | 0.003274 | 0.119797 | MME | membrane metallo-endopeptidase; NULL |
| 17046595 | 2.79 | 0.000974 | 0.074598 | VKORC1L1 | vitamin K epoxide reductase complex, subunit 1-like 1 |
| 16819217 | 2.78 | 0.003888 | 0.130247 | MT1E | metallothionein 1E |
| 16698356 | 2.78 | 0.011553 | 0.208602 | PIK3C2B | phosphatidylinositol-4-phosphate 3-kinase, catalytic subunit type 2 beta; NULL |
| 16763138 | 2.77 | 0.00035 | 0.04876 | KIF21A | kinesin family member 21A |
| 16698573 | 2.77 | 0.028801 | 0.302289 | PM20D1 | peptidase M20 domain containing 1 |
| 16932204 | 2.77 | 0.000291 | 0.045501 | SLC25A1 | solute carrier family 25 (mitochondrial carrier; citrate transporter), member 1; NULL |
| 16801707 | 2.77 | 0.000322 | 0.047683 | TLN2 | talin 2; NULL |
| 16708192 | 2.76 | 0.006257 | 0.161974 | ABCC2 | ATP-binding cassette, sub-family C (CFTR/MRP), member 2 |
| 16780917 | 2.76 | 0.00018 | 0.036571 | IRS2 | insulin receptor substrate 2 |
| 16664569 | 2.75 | 0.022178 | 0.273126 | CDKN2C | cyclin-dependent kinase inhibitor 2C (p18, inhibits CDK4) |
| 16942270 | 2.74 | 0.000466 | 0.055627 | PTPRG | protein tyrosine phosphatase, receptor type, G; NULL |
| 16671264 | 2.74 | 0.008653 | 0.1853 | SLC27A3 | solute carrier family 27 (fatty acid transporter), member 3; NULL |
| 16974830 | 2.73 | 0.01457 | 0.231078 | PPARGC1A | peroxisome proliferator-activated receptor gamma, coactivator 1 alpha; NULL |
| 16986249 | 2.72 | 0.000794 | 0.068667 | HMGCR | 3-hydroxy-3-methylglutaryl-CoA reductase; NULL |
| 16739132 | 2.71 | 0.000132 | 0.031928 | FADS1; MIR1908 | fatty acid desaturase 1; microRNA 1908; NULL |
| 17095703 | 2.71 | 0.002906 | 0.11613 | NFIL3 | nuclear factor, interleukin 3 regulated |
| 17004167 | 2.7 | 0.029371 | 0.304091 | IRF4 | interferon regulatory factor 4 |
| 16928204 | 2.7 | 0.019778 | 0.261888 | POM121L9P; LOC727983 | POM121 transmembrane nucleoporin-like 9, pseudogene; putative POM121-like protein 1-like |
| 16884967 | 2.69 | 0.00104 | 0.075752 | DBI | diazepam binding inhibitor (GABA receptor modulator, acyl-CoA binding protein); NULL |
| 17005223 | 2.69 | 0.004042 | 0.133343 | ID4 | inhibitor of DNA binding 4, dominant negative helix-loop-helix protein |
| 16761858 | 2.69 | 0.028027 | 0.299608 | RERG | RAS-like, estrogen-regulated, growth inhibitor; NULL |
| 16829139 | 2.68 | 0.003205 | 0.119176 | MVD | mevalonate (diphospho) decarboxylase; NULL |
| 16830883 | 2.67 | 0.028743 | 0.302123 | ALOX15B | arachidonate 15-lipoxygenase, type B |
| 16906175 | 2.67 | 0.013103 | 0.219542 | FRZB | frizzled-related protein |
| 17070307 | 2.65 | 0.001134 | 0.078119 | FABP5; LOC101060453 | fatty acid binding protein 5 (psoriasis-associated); fatty acid-binding protein, epidermal-like |
| 16831306 | 2.64 | 0.006496 | 0.163944 | HS3ST3B1 | heparan sulfate (glucosamine) 3-O-sulfotransferase 3B1 |
| 16890207 | 2.64 | 0.031617 | 0.311642 | MAP2 | microtubule-associated protein 2; NULL |
| 16891603 | 2.63 | 0.004376 | 0.138634 | KCNE4 | potassium voltage-gated channel, Isk-related family, member 4 |
| 16695741 | 2.63 | 0.018712 | 0.255415 | OLFML2B | olfactomedin-like 2B |
| 16677698 | 2.62 | 0.000698 | 0.065717 | MARC1 | mitochondrial amidoxime reducing component 1; NULL |
| 16702935 | 2.61 | 0.003855 | 0.12981 | CACNB2 | calcium channel, voltage-dependent, beta 2 subunit; NULL |
| 16699877 | 2.6 | 0.001669 | 0.09098 | LBR | lamin B receptor; NULL |
| 16748788 | 2.6 | 0.000239 | 0.042023 | MGST1 | microsomal glutathione S-transferase 1; NULL |
| 16884187 | 2.6 | 0.003109 | 0.118153 | SH3RF3 | SH3 domain containing ring finger 3 |
| 16743111 | 2.59 | 0.007905 | 0.179186 | CTSC | cathepsin C; NULL |
| 16928428 | 2.58 | 0.042669 | 0.355518 | ADRBK2 | adrenergic, beta, receptor kinase 2 |
| 16681304 | 2.58 | 0.010185 | 0.197179 | ERRFI1 | ERBB receptor feedback inhibitor 1 |
| 16920150 | 2.57 | 0.005789 | 0.158435 | KCNB1 | potassium voltage-gated channel, Shab-related subfamily, member 1 |
| 16996234 | 2.56 | 0.002106 | 0.10245 | PPAP2A | phosphatidic acid phosphatase type 2A; NULL |
| 16708179 | 2.55 | 0.001691 | 0.091539 | CUTC | cutC copper transporter homolog (E. coli); NULL |
| 17106688 | 2.55 | 0.044923 | 0.362283 | GRIA3 | glutamate receptor, ionotropic, AMPA 3; NULL |
| 16688506 | 2.55 | 0.023524 | 0.27936 | NEGR1 | neuronal growth regulator 1; NULL |
| 17112996 | 2.54 | 0.001225 | 0.07992 | MORF4L2 | mortality factor 4 like 2; NULL |
| 16908897 | 2.53 | 0.006592 | 0.164645 | EPHA4 | EPH receptor A4; NULL |
| 16914478 | 2.53 | 0.001144 | 0.078198 | EYA2 | eyes absent homolog 2 (Drosophila) |
| 16881838 | 2.53 | 0.002681 | 0.112646 | HK2 | hexokinase 2 |
| 16731084 | 2.53 | 0.002513 | 0.110705 | SIK2 | salt-inducible kinase 2 |
| 16966712 | 2.51 | 0.000005 | 0.006299 | DANCR | differentiation antagonizing non-protein coding RNA; NULL |
| 16911517 | 2.51 | 0.028417 | 0.301025 | ISM1 | isthmin 1, angiogenesis inhibitor |
| 17103327 | 2.5 | 0.004869 | 0.14629 | EBP | emopamil binding protein (sterol isomerase); NULL |
| 16894491 | 2.49 | 0.001949 | 0.098552 | ROCK2 | Rho-associated, coiled-coil containing protein kinase 2; NULL |
| 17100087 | 2.47 | 0.003605 | 0.125919 | AGPAT2 | 1-acylglycerol-3-phosphate O-acyltransferase 2 |
| 16696614 | 2.47 | 0.022968 | 0.276214 | KIAA0040 | KIAA0040 |
| 16772144 | 2.47 | 0.001708 | 0.092112 | SCARB1 | scavenger receptor class B, member 1; NULL |
| 17099705 | 2.46 | 0.028516 | 0.301407 | MIR3689F | microRNA 3689f |
| 16819883 | 2.46 | 0.004875 | 0.146391 | PDP2 | pyruvate dehyrogenase phosphatase catalytic subunit 2; NULL |
| 16722562 | 2.46 | 0.000129 | 0.031928 | SAA1 | serum amyloid A1 |
| 17090296 | 2.45 | 0.00715 | 0.170634 | ASS1 | argininosuccinate synthase 1; NULL |
| 16852702 | 2.44 | 0.001092 | 0.076964 | CDH20 | cadherin 20, type 2 |
| 17067231 | 2.44 | 0.026289 | 0.291071 | PTK2B | protein tyrosine kinase 2 beta; NULL |
| 16851565 | 2.43 | 0.000737 | 0.067222 | TTC39C | tetratricopeptide repeat domain 39C |
| 17114394 | 2.41 | 0.002391 | 0.108938 | LINC00087 | long intergenic non-protein coding RNA 87 |
| 17111501 | 2.41 | 0.013153 | 0.219763 | PFKFB1 | 6-phosphofructo-2-kinase/fructose-2,6-biphosphatase 1 |
| 16949792 | 2.4 | 0.040947 | 0.349936 | FAM43A | family with sequence similarity 43, member A |
| 16749782 | 2.4 | 0.001844 | 0.095945 | FGD4 | FYVE, RhoGEF and PH domain containing 4; NULL |
| 17024144 | 2.4 | 0.000033 | 0.01636 | IFNGR1 | interferon gamma receptor 1; NULL |
| 16936947 | 2.4 | 0.015369 | 0.236948 | ITPR1 | inositol 1,4,5-trisphosphate receptor, type 1; NULL |
| 16762146 | 2.4 | 0.004813 | 0.145722 | KCNJ8 | potassium inwardly-rectifying channel, subfamily J, member 8; NULL |
| 16919418 | 2.38 | 0.000587 | 0.062016 | FITM2 | fat storage-inducing transmembrane protein 2 |
| 17076063 | 2.38 | 0.002576 | 0.110955 | GSR | glutathione reductase |
| 17002898 | 2.38 | 0.017149 | 0.246354 | STC2 | stanniocalcin 2 |
| 16922443 | 2.37 | 0.009923 | 0.195709 | CLIC6 | chloride intracellular channel 6 |
| 16748989 | 2.37 | 0.025504 | 0.288999 | PDE3A | phosphodiesterase 3A, cGMP-inhibited |
| 16906733 | 2.37 | 0.004281 | 0.137171 | STK17B | serine/threonine kinase 17b |
| 17068134 | 2.37 | 0.012098 | 0.212206 | TACC1 | transforming, acidic coiled-coil containing protein 1 |
| 16946707 | 2.36 | 0.027141 | 0.295172 | AGTR1 | angiotensin II receptor, type 1 |
| 17113744 | 2.36 | 0.000113 | 0.029831 | CUL4B | cullin 4B |
| 16986501 | 2.36 | 0.000237 | 0.042023 | PDE8B | phosphodiesterase 8B |
| 16959386 | 2.36 | 0.01401 | 0.226442 | SLCO2A1 | solute carrier organic anion transporter family, member 2A1; NULL |
| 17109042 | 2.35 | 0.034678 | 0.324319 | MID1 | midline 1 (Opitz/BBB syndrome) |
| 16678518 | 2.34 | 0.03914 | 0.343304 | RHOU; DUSP5P1 | ras homolog family member U; dual specificity phosphatase 5 pseudogene 1 |
| 17072669 | 2.33 | 0.000306 | 0.046413 | MYC | v-myc myelocytomatosis viral oncogene homolog (avian) |
| 16741501 | 2.32 | 0.001112 | 0.077373 | DHCR7 | 7-dehydrocholesterol reductase; NULL |
| 16717816 | 2.32 | 0.000705 | 0.065717 | KCNIP2 | Kv channel interacting protein 2; NULL |
| 16947287 | 2.31 | 0.000456 | 0.055203 | TIPARP | TCDD-inducible poly(ADP-ribose) polymerase; NULL |
| 16962519 | 2.29 | 0.002892 | 0.115964 | ADIPOQ-AS1 | ADIPOQ antisense RNA 1 |
| 17004859 | 2.29 | 0.019359 | 0.259404 | TMEM170B | transmembrane protein 170B |
| 16757391 | 2.28 | 0.007424 | 0.172913 | DTX1 | deltex homolog 1 (Drosophila) |
| 17005787 | 2.25 | 0.029395 | 0.304203 | HIST1H2AH; HIST1H2AG; HIST1H2AM; HIST1H2AL; HIST1H2AK; HIST1H2AI | histone cluster 1, H2ah; histone cluster 1, H2ag; histone cluster 1, H2am; histone cluster 1, H2al; histone cluster 1, H2ak; histone cluster 1, H2ai |
| 16763195 | 2.25 | 0.013698 | 0.223749 | SLC2A13 | solute carrier family 2 (facilitated glucose transporter), member 13 |
| 16888669 | 2.24 | 0.010394 | 0.198698 | MIR1245A; MIR1245B | microRNA 1245a; microRNA 1245b |
| 16922134 | 2.24 | 0.005478 | 0.155708 | MRAP | melanocortin 2 receptor accessory protein |
| 16895673 | 2.24 | 0.001211 | 0.079847 | SLC5A6 | solute carrier family 5 (sodium-dependent vitamin transporter), member 6; NULL |
| 17019805 | 2.24 | 0.025403 | 0.288865 | TNFRSF21 | tumor necrosis factor receptor superfamily, member 21 |
| 16870821 | 2.24 | 0.028243 | 0.300525 | ZNF100 | zinc finger protein 100 |
| 17106183 | 2.23 | 0.000328 | 0.048061 | TMEM164 | transmembrane protein 164 |
| 17078983 | 2.23 | 0.000035 | 0.016644 | TMEM64 | transmembrane protein 64 |
| 16834091 | 2.22 | 0.001652 | 0.09098 | IGFBP4 | insulin-like growth factor binding protein 4 |
| 16824352 | 2.22 | 0.014817 | 0.232841 | XYLT1 | xylosyltransferase I |
| 17047411 | 2.21 | 0.000253 | 0.042689 | POR | P450 (cytochrome) oxidoreductase; NULL |
| 16819244 | 2.2 | 0.013987 | 0.226207 | MT1CP | metallothionein 1C, pseudogene; NULL |
| 17102512 | 2.2 | 0.01532 | 0.236581 | PRRG1 | proline rich Gla (G-carboxyglutamic acid) 1; NULL |
| 16729298 | 2.19 | 0.00422 | 0.136091 | ACER3 | alkaline ceramidase 3; NULL |
| 16822014 | 2.19 | 0.00251 | 0.110705 | CPNE7 | copine VII |
| 16991180 | 2.19 | 0.031069 | 0.30948 | SMIM3 | small integral membrane protein 3 |
| 17002278 | 2.18 | 0.040919 | 0.349845 | EBF1 | early B-cell factor 1; NULL |
| 16879118 | 2.18 | 0.003503 | 0.124045 | GPATCH11 | G patch domain containing 11 |
| 16830302 | 2.17 | 0.000477 | 0.055896 | ACADVL | acyl-CoA dehydrogenase, very long chain; NULL |
| 16835972 | 2.17 | 0.001796 | 0.094369 | CACNA1G | calcium channel, voltage-dependent, T type, alpha 1G subunit; NULL |
| 17104363 | 2.17 | 0.016011 | 0.240527 | EFNB1 | ephrin-B1 |
| 16970465 | 2.17 | 0.005358 | 0.153746 | FAT4 | FAT atypical cadherin 4 |
| 16750190 | 2.17 | 0.013716 | 0.223749 | PDZRN4 | PDZ domain containing ring finger 4 |
| 16836292 | 2.16 | 0.018512 | 0.254733 | ANKFN1 | ankyrin-repeat and fibronectin type III domain containing 1 |
| 16958506 | 2.16 | 0.005189 | 0.151659 | OSBPL11 | oxysterol binding protein-like 11 |
| 17107230 | 2.15 | 0.000219 | 0.040061 | FHL1 | four and a half LIM domains 1 |
| 17053892 | 2.15 | 0.027157 | 0.295172 | INSIG1 | insulin induced gene 1 |
| 17022996 | 2.15 | 0.000378 | 0.050487 | ROS1; GOPC | c-ros oncogene 1 , receptor tyrosine kinase; golgi-associated PDZ and coiled-coil motif containing |
| 16706641 | 2.15 | 0.004844 | 0.146031 | TSPAN14 | tetraspanin 14 |
| 16877941 | 2.14 | 0.002641 | 0.111971 | KCNK3 | potassium channel, subfamily K, member 3 |
| 16987287 | 2.14 | 0.000407 | 0.052249 | NR2F1; NR2F2 | nuclear receptor subfamily 2, group F, member 1; nuclear receptor subfamily 2, group F, member 2 |
| 16908154 | 2.14 | 0.002552 | 0.110864 | PECR | peroxisomal trans-2-enoyl-CoA reductase; NULL |
| 16855184 | 2.13 | 0.001061 | 0.075909 | ACAA2 | acetyl-CoA acyltransferase 2 |
| 16984365 | 2.13 | 0.001592 | 0.089691 | GHR | growth hormone receptor; NULL |
| 17097211 | 2.13 | 0.001242 | 0.07992 | PTGR1 | prostaglandin reductase 1 |
| 16925983 | 2.12 | 0.001209 | 0.079847 | C2CD2 | C2 calcium-dependent domain containing 2; NULL |
| 17104313 | 2.11 | 0.00878 | 0.185567 | AR | androgen receptor |
| 17013002 | 2.11 | 0.000861 | 0.070943 | HEBP2 | heme binding protein 2 |
| 16851486 | 2.11 | 0.001082 | 0.076626 | LAMA3 | laminin, alpha 3 |
| 16856604 | 2.11 | 0.005941 | 0.1597 | REEP6 | receptor accessory protein 6 |
| 16715088 | 2.1 | 0.023873 | 0.281742 | AIFM2 | apoptosis-inducing factor, mitochondrion-associated, 2 |
| 17059628 | 2.1 | 0.0055 | 0.155942 | CYP51A1; LRRD1 | cytochrome P450, family 51, subfamily A, polypeptide 1; leucine-rich repeats and death domain containing 1 |
| 16818610 | 2.1 | 0.005758 | 0.158401 | GPT2 | glutamic pyruvate transaminase (alanine aminotransferase) 2 |
| 17105332 | 2.1 | 0.025454 | 0.288951 | SRPX2 | sushi-repeat containing protein, X-linked 2 |
| 16914062 | 2.1 | 0.001899 | 0.097205 | TTPAL | tocopherol (alpha) transfer protein-like |
| 16757990 | 2.09 | 0.004813 | 0.145722 | ACADS | acyl-CoA dehydrogenase, C-2 to C-3 short chain |
| 16839642 | 2.09 | 0.006648 | 0.165136 | CLUH; KIAA0664 | clustered mitochondria (cluA/CLU1) homolog; NULL |
| 16833533 | 2.09 | 0.005788 | 0.158435 | MIR2909 | microRNA 2909 |
| 16821404 | 2.09 | 0.000214 | 0.039716 | MLYCD | malonyl-CoA decarboxylase |
| 17088462 | 2.09 | 0.018207 | 0.252776 | PAPPA | pregnancy-associated plasma protein A, pappalysin 1 |
| 16849992 | 2.09 | 0.00665 | 0.165136 | PCYT2 | phosphate cytidylyltransferase 2, ethanolamine; NULL |
| 16744289 | 2.09 | 0.000994 | 0.074654 | PPP2R1B | protein phosphatase 2, regulatory subunit A, beta; NULL |
| 17069550 | 2.08 | 0.005992 | 0.159828 | ADHFE1 | alcohol dehydrogenase, iron containing, 1; NULL |
| 16987766 | 2.08 | 0.017905 | 0.251228 | C5orf30 | chromosome 5 open reading frame 30; NULL |
| 17114701 | 2.08 | 0.000557 | 0.060129 | CDR1 | cerebellar degeneration-related protein 1, 34kDa |
| 16861887 | 2.08 | 0.014402 | 0.229945 | ECH1 | enoyl CoA hydratase 1, peroxisomal |
| 17011217 | 2.08 | 0.008353 | 0.183766 | GRIK2 | glutamate receptor, ionotropic, kainate 2; NULL |
| 16999321 | 2.08 | 0.017857 | 0.250969 | ZNF608 | zinc finger protein 608 |
| 16908618 | 2.07 | 0.011938 | 0.211293 | ABCB6; ATG9A | ATP-binding cassette, sub-family B (MDR/TAP), member 6; autophagy related 9A; NULL |
| 16939815 | 2.07 | 0.003081 | 0.117507 | ABHD5 | abhydrolase domain containing 5; NULL |
| 16966137 | 2.07 | 0.003858 | 0.129823 | LIAS | lipoic acid synthetase |
| 16737843 | 2.07 | 0.000961 | 0.074302 | LRP4 | low density lipoprotein receptor-related protein 4; NULL |
| 16814498 | 2.07 | 0.002992 | 0.116744 | METRN | meteorin, glial cell differentiation regulator |
| 16850759 | 2.07 | 0.001754 | 0.09335 | PTPRM | protein tyrosine phosphatase, receptor type, M |
| 16687352 | 2.06 | 0.001263 | 0.080233 | LRP8 | low density lipoprotein receptor-related protein 8, apolipoprotein e receptor |
| 16843049 | 2.06 | 0.005972 | 0.159828 | SSH2 | slingshot protein phosphatase 2 |
| 17095150 | 2.06 | 0.007405 | 0.172906 | TLE1 | transducin-like enhancer of split 1 (E(sp1) homolog, Drosophila); NULL |
| 16843728 | 2.05 | 0.001245 | 0.07992 | ACACA | acetyl-CoA carboxylase alpha; NULL |
| 17113774 | 2.05 | 0.000308 | 0.046413 | C1GALT1C1 | C1GALT1-specific chaperone 1 |
| 16705934 | 2.05 | 0.013811 | 0.224358 | CHST3 | carbohydrate (chondroitin 6) sulfotransferase 3 |
| 16688269 | 2.05 | 0.006153 | 0.160918 | SLC35D1 | solute carrier family 35 (UDP-glucuronic acid/UDP-N-acetylgalactosamine dual transporter), member D1 |
| 16894139 | 2.04 | 0.034228 | 0.322269 | RNF144A-AS1 | RNF144A antisense RNA 1; NULL |
| 17109432 | 2.03 | 0.003403 | 0.121919 | RAI2 | retinoic acid induced 2 |
| 17118378 | 2.02 | 0.013152 | 0.219763 | FAM27E3; FAM27E2 | family with sequence similarity 27, member E3; family with sequence similarity 27, member E2 |
| 16753670 | 2.02 | 0.004275 | 0.137077 | IRAK3 | interleukin-1 receptor-associated kinase 3 |
| 16845249 | 2.02 | 0.000325 | 0.047767 | RAMP2-AS1 | RAMP2 antisense RNA 1 |
| 16852982 | 2.01 | 0.007435 | 0.172963 | DOK6 | docking protein 6 |
| 16743647 | 2 | 0.007949 | 0.179474 | MMP7 | matrix metallopeptidase 7 (matrilysin, uterine) |
|  |  |  |  |  |  |
| **Down-regulated genes on day 14** | | | |  |  |
| Transcript Cluster ID | Fold Change (linear) (Induced vs. Control) | ANOVA p-value (Induced vs. Control) | FDR p-value (Induced vs. Control) | Gene Symbol | Description |
| 16855600 | -2 | 0.039116 | 0.343232 | CCBE1 | collagen and calcium binding EGF domains 1 |
| 17062255 | -2 | 0.018946 | 0.256884 | FAM3C | family with sequence similarity 3, member C |
| 17024335 | -2 | 0.000681 | 0.065717 | HIVEP2 | human immunodeficiency virus type I enhancer binding protein 2 |
| 16996433 | -2 | 0.023863 | 0.281726 | PLK2 | polo-like kinase 2; NULL |
| 17083452 | -2 | 0.006173 | 0.16108 | UHRF2 | ubiquitin-like with PHD and ring finger domains 2, E3 ubiquitin protein ligase; NULL |
| 16882285 | -2 | 0.000512 | 0.058063 | VAMP5 | vesicle-associated membrane protein 5 |
| 16811500 | -2.01 | 0.00196 | 0.098607 | NPTN | neuroplastin; NULL |
| 16859788 | -2.01 | 0.005468 | 0.155708 | PGPEP1 | pyroglutamyl-peptidase I; NULL |
| 16960149 | -2.01 | 0.001714 | 0.092272 | PLSCR4 | phospholipid scramblase 4; NULL |
| 16774789 | -2.01 | 0.019587 | 0.260934 | SETDB2 | SET domain, bifurcated 2 |
| 17059872 | -2.01 | 0.000612 | 0.063216 | SGCE | sarcoglycan, epsilon |
| 17069816 | -2.01 | 0.041963 | 0.35361 | SULF1 | sulfatase 1; NULL |
| 16928533 | -2.02 | 0.01568 | 0.239 | ASPHD2 | aspartate beta-hydroxylase domain containing 2 |
| 17015862 | -2.02 | 0.015095 | 0.235212 | ATXN1 | ataxin 1; NULL |
| 16718047 | -2.02 | 0.030131 | 0.306824 | CALHM2 | calcium homeostasis modulator 2 |
| 16834056 | -2.02 | 0.049436 | 0.376815 | CDC6 | cell division cycle 6; NULL |
| 16978976 | -2.02 | 0.029747 | 0.305294 | CFI | complement factor I |
| 17083742 | -2.02 | 0.012482 | 0.214684 | CNTLN | centlein, centrosomal protein |
| 17093090 | -2.02 | 0.002207 | 0.10478 | DDX58 | DEAD (Asp-Glu-Ala-Asp) box polypeptide 58 |
| 16845537 | -2.02 | 0.00341 | 0.121919 | HDAC5 | histone deacetylase 5 |
| 16850609 | -2.02 | 0.00919 | 0.188672 | MYL12A | myosin, light chain 12A, regulatory, non-sarcomeric; NULL |
| 16718938 | -2.02 | 0.004178 | 0.13568 | RGS10 | regulator of G-protein signaling 10 |
| 16768923 | -2.02 | 0.02995 | 0.306071 | SLC9A7P1 | solute carrier family 9, subfamily A (NHE7, cation proton antiporter 7), member 7 pseudogene 1 |
| 16719217 | -2.03 | 0.045184 | 0.362873 | CHST15 | carbohydrate (N-acetylgalactosamine 4-sulfate 6-O) sulfotransferase 15 |
| 16775968 | -2.03 | 0.002223 | 0.105032 | FARP1; FARP1-IT1 | FERM, RhoGEF (ARHGEF) and pleckstrin domain protein 1 (chondrocyte-derived); FARP1 intronic transcript 1 (non-protein coding); NULL |
| 17060503 | -2.03 | 0.014843 | 0.23286 | GAL3ST4 | galactose-3-O-sulfotransferase 4 |
| 17058758 | -2.03 | 0.01097 | 0.204098 | GATSL2 | GATS protein-like 2 |
| 16788258 | -2.03 | 0.031768 | 0.312102 | HHIPL1 | HHIP-like 1 |
| 16742454 | -2.03 | 0.000399 | 0.051776 | PAK1 | p21 protein (Cdc42/Rac)-activated kinase 1; NULL |
| 16886757 | -2.03 | 0.006167 | 0.161034 | PKP4 | plakophilin 4; NULL |
| 16808334 | -2.03 | 0.001654 | 0.09098 | PPIP5K1; OTTHUMG00000059903; AC011330.5 | diphosphoinositol pentakisphosphate kinase 1; NULL |
| 16665796 | -2.03 | 0.027351 | 0.29648 | SGIP1 | SH3-domain GRB2-like (endophilin) interacting protein 1; NULL |
| 16839019 | -2.03 | 0.010189 | 0.197179 | SLC16A3 | solute carrier family 16, member 3 (monocarboxylic acid transporter 4); NULL |
| 16906571 | -2.03 | 0.026582 | 0.292119 | STAT4 | signal transducer and activator of transcription 4; NULL |
| 16961003 | -2.03 | 0.001052 | 0.075909 | TRIM59; OTTHUMG00000162252; RP11-432B6.3 | tripartite motif containing 59; NULL |
| 16922920 | -2.04 | 0.017988 | 0.252036 | BACE2 | beta-site APP-cleaving enzyme 2; NULL |
| 16812598 | -2.04 | 0.008471 | 0.184735 | CPEB1; OTTHUMG00000172875; RP11-152F13.10 | cytoplasmic polyadenylation element binding protein 1; NULL |
| 16740828 | -2.04 | 0.01769 | 0.249975 | CTSF | cathepsin F; NULL |
| 16787135 | -2.04 | 0.001574 | 0.089511 | FLRT2; LOC100506718 | fibronectin leucine rich transmembrane protein 2; uncharacterized LOC100506718 |
| 16784098 | -2.04 | 0.000637 | 0.064339 | FRMD6 | FERM domain containing 6; NULL |
| 16952841 | -2.04 | 0.008746 | 0.185507 | FYCO1 | FYVE and coiled-coil domain containing 1 |
| 16802605 | -2.04 | 0.004089 | 0.134268 | LRRC49 | leucine rich repeat containing 49; NULL |
| 16841340 | -2.04 | 0.016387 | 0.24205 | MYH2 | myosin, heavy chain 2, skeletal muscle, adult |
| 17109716 | -2.04 | 0.000062 | 0.023091 | RPS6KA3 | ribosomal protein S6 kinase, 90kDa, polypeptide 3 |
| 16859802 | -2.04 | 0.000535 | 0.059471 | SSBP4 | single stranded DNA binding protein 4; NULL |
| 16688961 | -2.04 | 0.000947 | 0.074302 | SSX2IP | synovial sarcoma, X breakpoint 2 interacting protein; NULL |
| 17096242 | -2.05 | 0.044955 | 0.36236 | AAED1 | AhpC/TSA antioxidant enzyme domain containing 1 |
| 16989897 | -2.05 | 0.000065 | 0.023565 | CXXC5 | CXXC finger protein 5; NULL |
| 16665558 | -2.05 | 0.047053 | 0.368613 | DLEU2L | deleted in lymphocytic leukemia 2-like |
| 17073259 | -2.05 | 0.020664 | 0.264967 | LY6E | lymphocyte antigen 6 complex, locus E; NULL |
| 17059771 | -2.05 | 0.000347 | 0.04876 | SAMD9 | sterile alpha motif domain containing 9 |
| 16779766 | -2.06 | 0.003578 | 0.125238 | KLF12 | Kruppel-like factor 12 |
| 16748196 | -2.06 | 0.037378 | 0.336477 | LOC374443; OTTHUMG00000168360; RP11-705C15.2 | C-type lectin domain family 2, member D pseudogene; NULL |
| 16833060 | -2.06 | 0.001148 | 0.078213 | LRRC37B | leucine rich repeat containing 37B; NULL |
| 16809596 | -2.06 | 0.002143 | 0.103024 | RAB27A | RAB27A, member RAS oncogene family |
| 16667702 | -2.06 | 0.043197 | 0.356533 | VCAM1 | vascular cell adhesion molecule 1 |
| 16966049 | -2.07 | 0.000805 | 0.069223 | KLHL5 | kelch-like family member 5 |
| 16882561 | -2.07 | 0.049697 | 0.377773 | LINC00152; LOC541471 | NULL; uncharacterized LOC541471; long intergenic non-protein coding RNA 152 |
| 17042895 | -2.07 | 0.001038 | 0.075752 | MAFK | v-maf musculoaponeurotic fibrosarcoma oncogene homolog K (avian); NULL |
| 16702547 | -2.07 | 0.000481 | 0.055896 | OPTN | optineurin; NULL |
| 16854744 | -2.07 | 0.003809 | 0.129169 | TPGS2 | tubulin polyglutamylase complex subunit 2; NULL |
| 16662636 | -2.08 | 0.011056 | 0.204917 | DNALI1 | dynein, axonemal, light intermediate chain 1 |
| 16808401 | -2.08 | 0.001198 | 0.079575 | FRMD5 | FERM domain containing 5; NULL |
| 16769419 | -2.08 | 0.004222 | 0.136091 | GLT8D2 | glycosyltransferase 8 domain containing 2 |
| 16836824 | -2.08 | 0.003628 | 0.126365 | MRC2 | mannose receptor, C type 2 |
| 16824132 | -2.08 | 0.002856 | 0.11555 | NTAN1 | N-terminal asparagine amidase; NULL |
| 16785938 | -2.08 | 0.002728 | 0.113587 | TTC9 | tetratricopeptide repeat domain 9 |
| 16762337 | -2.09 | 0.00061 | 0.063216 | BCAT1 | branched chain amino-acid transaminase 1, cytosolic |
| 16847949 | -2.09 | 0.000171 | 0.035784 | CEP112 | centrosomal protein 112kDa; NULL |
| 17098698 | -2.09 | 0.002234 | 0.105032 | FAM102A | family with sequence similarity 102, member A; NULL |
| 16864129 | -2.09 | 0.016987 | 0.246273 | FLT3LG; OTTHUMG00000183164; CTD-3148I10.9 | fms-related tyrosine kinase 3 ligand; NULL |
| 16807288 | -2.09 | 0.012168 | 0.212619 | GPR176 | G protein-coupled receptor 176 |
| 16816200 | -2.09 | 0.000057 | 0.022513 | NDE1 | nudE nuclear distribution E homolog 1 (A. nidulans); NULL |
| 16843404 | -2.09 | 0.0003 | 0.046287 | SLFN11 | schlafen family member 11; NULL |
| 16915245 | -2.1 | 0.033673 | 0.320424 | APCDD1L-AS1 | APCDD1L antisense RNA 1 (head to head) |
| 16996030 | -2.1 | 0.017697 | 0.249975 | EMB | embigin |
| 16761617 | -2.1 | 0.046655 | 0.367353 | MANSC1 | MANSC domain containing 1 |
| 16981345 | -2.1 | 0.006034 | 0.159828 | SH3RF1 | SH3 domain containing ring finger 1; NULL |
| 17079338 | -2.11 | 0.00223 | 0.105032 | C8orf37 | chromosome 8 open reading frame 37 |
| 16944665 | -2.11 | 0.041983 | 0.353719 | DTX3L | deltex 3-like (Drosophila) |
| 16897834 | -2.11 | 0.001447 | 0.08636 | EFEMP1 | EGF containing fibulin-like extracellular matrix protein 1; NULL |
| 17071625 | -2.11 | 0.003236 | 0.1192 | FZD6 | frizzled family receptor 6; NULL |
| 16880669 | -2.11 | 0.011525 | 0.208586 | LGALSL | lectin, galactoside-binding-like; NULL |
| 17052935 | -2.11 | 0.009919 | 0.195709 | OR2A20P; OR2A9P; OR2A7 | olfactory receptor, family 2, subfamily A, member 20 pseudogene; olfactory receptor, family 2, subfamily A, member 9 pseudogene; olfactory receptor, family 2, subfamily A, member 7 |
| 17087588 | -2.11 | 0.000133 | 0.031928 | TMEFF1; MSANTD3-TMEFF1; MSANTD3 | transmembrane protein with EGF-like and two follistatin-like domains 1; MSANTD3-TMEFF1 readthrough; Myb/SANT-like DNA-binding domain containing 3 |
| 16720416 | -2.11 | 0.001562 | 0.089048 | TSPAN4 | tetraspanin 4; NULL |
| 16954217 | -2.11 | 0.002493 | 0.110675 | UBA7; MIR5193 | ubiquitin-like modifier activating enzyme 7; microRNA 5193; NULL |
| 16701185 | -2.12 | 0.004675 | 0.143696 | CEP170; CEP170P1 | centrosomal protein 170kDa; centrosomal protein 170kDa pseudogene 1; NULL |
| 16771680 | -2.12 | 0.00081 | 0.069352 | CLIP1 | CAP-GLY domain containing linker protein 1; NULL |
| 16743056 | -2.12 | 0.000132 | 0.031928 | ME3 | malic enzyme 3, NADP(+)-dependent, mitochondrial; NULL |
| 17012342 | -2.13 | 0.009865 | 0.195382 | HINT3 | histidine triad nucleotide binding protein 3 |
| 16840318 | -2.13 | 0.011358 | 0.206945 | LOC728392; NLRP1 | uncharacterized LOC728392; NLR family, pyrin domain containing 1; NULL |
| 16753030 | -2.13 | 0.031288 | 0.31041 | PIP4K2C | phosphatidylinositol-5-phosphate 4-kinase, type II, gamma; NULL |
| 16683574 | -2.13 | 0.000737 | 0.067222 | STPG1 | sperm-tail PG-rich repeat containing 1 |
| 16670599 | -2.14 | 0.043288 | 0.356774 | ADAMTSL4 | ADAMTS-like 4 |
| 17043882 | -2.14 | 0.007867 | 0.178752 | HDAC9 | histone deacetylase 9; NULL |
| 16999421 | -2.14 | 0.000275 | 0.044067 | MARCH3 | membrane-associated ring finger (C3HC4) 3, E3 ubiquitin protein ligase |
| 16712482 | -2.14 | 0.027957 | 0.299608 | PIP4K2A | phosphatidylinositol-5-phosphate 4-kinase, type II, alpha; NULL |
| 16721593 | -2.14 | 0.000013 | 0.010488 | PPFIBP2 | PTPRF interacting protein, binding protein 2 (liprin beta 2); NULL |
| 16667498 | -2.14 | 0.000437 | 0.053853 | SNX7 | sorting nexin 7 |
| 16922011 | -2.15 | 0.00098 | 0.074654 | BACH1; GRIK1-AS2; BACH1-IT1 | BTB and CNC homology 1, basic leucine zipper transcription factor 1; GRIK1 antisense RNA 2; NULL |
| 16972167 | -2.15 | 0.006677 | 0.165554 | CPE | carboxypeptidase E |
| 16834931 | -2.15 | 0.001899 | 0.097205 | FMNL1 | formin-like 1 |
| 16857905 | -2.15 | 0.02612 | 0.29045 | MARCH2 | membrane-associated ring finger (C3HC4) 2, E3 ubiquitin protein ligase |
| 17017965 | -2.15 | 0.022788 | 0.27586 | PSMB8 | proteasome (prosome, macropain) subunit, beta type, 8; NULL |
| 17011593 | -2.16 | 0.000684 | 0.065717 | FIG4 | FIG4 homolog, SAC1 lipid phosphatase domain containing (S. cerevisiae) |
| 17104283 | -2.16 | 0.012791 | 0.216905 | HEPH | hephaestin; NULL |
| 16717224 | -2.16 | 0.003342 | 0.120806 | MORN4 | MORN repeat containing 4 |
| 16762661 | -2.16 | 0.004016 | 0.132648 | PTHLH | parathyroid hormone-like hormone |
| 17002612 | -2.16 | 0.00783 | 0.178212 | SLIT3 | slit homolog 3 (Drosophila) |
| 16872551 | -2.16 | 0.0068 | 0.167323 | TGFB1 | transforming growth factor, beta 1 |
| 16845336 | -2.16 | 0.004138 | 0.135017 | VAT1 | vesicle amine transport protein 1 homolog (T. californica) |
| 16969911 | -2.18 | 0.008424 | 0.184492 | ANK2 | ankyrin 2, neuronal; NULL |
| 16979163 | -2.18 | 0.008657 | 0.185313 | ARSJ | arylsulfatase family, member J |
| 16854904 | -2.18 | 0.002757 | 0.114052 | PSTPIP2 | proline-serine-threonine phosphatase interacting protein 2 |
| 17000858 | -2.19 | 0.000703 | 0.065717 | DIAPH1 | diaphanous-related formin 1; NULL |
| 16775434 | -2.19 | 0.011414 | 0.206945 | LMO7; OTTHUMG00000172802; RP11-29G8.3 | LIM domain 7; NULL |
| 16867414 | -2.19 | 0.00862 | 0.185002 | PLIN3 | perilipin 3 |
| 16674521 | -2.19 | 0.000369 | 0.049948 | QSOX1; FLJ23867 | quiescin Q6 sulfhydryl oxidase 1; uncharacterized protein FLJ23867 |
| 17067941 | -2.2 | 0.000368 | 0.049948 | GPR124 | G protein-coupled receptor 124 |
| 16826803 | -2.2 | 0.000129 | 0.031928 | KIFC3 | kinesin family member C3; NULL |
| 16684800 | -2.2 | 0.005617 | 0.157128 | TMEM54 | transmembrane protein 54; NULL |
| 16922501 | -2.21 | 0.00019 | 0.037155 | DOPEY2 | dopey family member 2 |
| 17010522 | -2.22 | 0.002883 | 0.11588 | IRAK1BP1 | interleukin-1 receptor-associated kinase 1 binding protein 1 |
| 17078870 | -2.22 | 0.00184 | 0.095833 | MMP16 | matrix metallopeptidase 16 (membrane-inserted) |
| 16863753 | -2.23 | 0.001935 | 0.09827 | EMP3 | epithelial membrane protein 3 |
| 16942103 | -2.23 | 0.029135 | 0.302823 | FLNB | filamin B, beta; NULL |
| 16839220 | -2.24 | 0.036864 | 0.333921 | FAM101B | family with sequence similarity 101, member B |
| 16971966 | -2.24 | 0.011107 | 0.205076 | FNIP2 | folliculin interacting protein 2 |
| 16958124 | -2.24 | 0.004233 | 0.136286 | PARP9 | poly (ADP-ribose) polymerase family, member 9 |
| 16664005 | -2.24 | 0.006753 | 0.166581 | PLK3 | polo-like kinase 3 |
| 16741864 | -2.25 | 0.003556 | 0.12489 | FCHSD2 | FCH and double SH3 domains 2 |
| 16674845 | -2.25 | 0.042375 | 0.354846 | LAMC2 | laminin, gamma 2 |
| 16842266 | -2.25 | 0.000645 | 0.064629 | MFAP4 | microfibrillar-associated protein 4 |
| 17079005 | -2.25 | 0.01684 | 0.245053 | TMEM55A | transmembrane protein 55A |
| 16755498 | -2.25 | 0.000295 | 0.04571 | TMPO | thymopoietin; NULL |
| 16721916 | -2.26 | 0.007139 | 0.170634 | AMPD3 | adenosine monophosphate deaminase 3; NULL |
| 16847565 | -2.26 | 0.034854 | 0.325038 | CYB561 | cytochrome b561; NULL |
| 17016946 | -2.26 | 0.001539 | 0.088448 | PPP1R18; OTTHUMG00000148752; KIAA1949; OTTHUMG00000031537; OTTHUMG00000148941; OTTHUMG00000149220; OTTHUMG00000149456; OTTHUMG00000004836; OTTHUMG00000149990 | protein phosphatase 1, regulatory subunit 18; NULL |
| 17075448 | -2.26 | 0.014277 | 0.229047 | TNFRSF10D | tumor necrosis factor receptor superfamily, member 10d, decoy with truncated death domain |
| 16757427 | -2.26 | 0.006316 | 0.162705 | TPCN1 | two pore segment channel 1; NULL |
| 16922495 | -2.27 | 0.021341 | 0.268719 | CBR3 | carbonyl reductase 3 |
| 17080450 | -2.27 | 0.000347 | 0.04876 | EXT1 | exostosin glycosyltransferase 1 |
| 17051286 | -2.27 | 0.036972 | 0.334339 | FLNC | filamin C, gamma |
| 17074342 | -2.27 | 0.007542 | 0.174537 | LINC00965 | long intergenic non-protein coding RNA 965 |
| 16873826 | -2.27 | 0.040005 | 0.346218 | PLA2G4C | phospholipase A2, group IVC (cytosolic, calcium-independent); NULL |
| 16765254 | -2.27 | 0.001725 | 0.092449 | RARG | retinoic acid receptor, gamma; NULL |
| 17025844 | -2.27 | 0.023354 | 0.278711 | THBS2 | thrombospondin 2 |
| 16987531 | -2.28 | 0.003151 | 0.118753 | ERAP2 | endoplasmic reticulum aminopeptidase 2; NULL |
| 16973944 | -2.28 | 0.004555 | 0.142377 | EVC2 | Ellis van Creveld syndrome 2; NULL |
| 16873296 | -2.28 | 0.046717 | 0.367353 | PPP1R13L | protein phosphatase 1, regulatory subunit 13 like; NULL |
| 16763295 | -2.28 | 0.01481 | 0.232799 | PRICKLE1 | prickle homolog 1 (Drosophila); NULL |
| 16820508 | -2.28 | 0.000675 | 0.065472 | TANGO6; TMCO7 | transport and golgi organization 6 homolog (Drosophila); NULL |
| 16956714 | -2.29 | 0.00737 | 0.172612 | DCBLD2 | discoidin, CUB and LCCL domain containing 2; NULL |
| 16809457 | -2.29 | 0.001063 | 0.075909 | MYO5A | myosin VA (heavy chain 12, myoxin); NULL |
| 17008105 | -2.29 | 0.00053 | 0.059298 | TBC1D22B | TBC1 domain family, member 22B |
| 16796694 | -2.29 | 0.012179 | 0.212657 | WARS | tryptophanyl-tRNA synthetase; NULL |
| 17015637 | -2.3 | 0.000174 | 0.036024 | ELOVL2 | ELOVL fatty acid elongase 2 |
| 16782687 | -2.3 | 0.007845 | 0.178462 | IRF9; OTTHUMG00000171951; RP11-468E2.4 | interferon regulatory factor 9; NULL |
| 16924878 | -2.3 | 0.002458 | 0.10984 | TIAM1 | T-cell lymphoma invasion and metastasis 1 |
| 16771067 | -2.31 | 0.042058 | 0.353898 | CIT; MIR1178 | citron (rho-interacting, serine/threonine kinase 21); microRNA 1178; NULL |
| 16712168 | -2.31 | 0.024324 | 0.283686 | CUBN | cubilin (intrinsic factor-cobalamin receptor) |
| 16815498 | -2.31 | 0.003925 | 0.130995 | GLIS2 | GLIS family zinc finger 2 |
| 16825638 | -2.31 | 0.001233 | 0.07992 | YPEL3 | yippee-like 3 (Drosophila); NULL |
| 17048879 | -2.32 | 0.012791 | 0.216905 | ARPC1B | actin related protein 2/3 complex, subunit 1B, 41kDa; NULL |
| 16726945 | -2.32 | 0.004759 | 0.144841 | EHBP1L1 | EH domain binding protein 1-like 1 |
| 16736821 | -2.32 | 0.03265 | 0.315421 | LGR4 | leucine-rich repeat containing G protein-coupled receptor 4 |
| 16969558 | -2.32 | 0.003243 | 0.1192 | SGMS2 | sphingomyelin synthase 2 |
| 16955324 | -2.33 | 0.011788 | 0.210727 | IL17RD | interleukin 17 receptor D; NULL |
| 17020464 | -2.33 | 0.000079 | 0.026324 | RAB23 | RAB23, member RAS oncogene family |
| 16965377 | -2.33 | 0.023673 | 0.280435 | SLIT2 | slit homolog 2 (Drosophila) |
| 16783602 | -2.33 | 0.004506 | 0.141353 | SSTR1 | somatostatin receptor 1 |
| 16986913 | -2.33 | 0.000588 | 0.062016 | VCAN | versican |
| 16858970 | -2.34 | 0.001251 | 0.079942 | CD97 | CD97 molecule |
| 17077502 | -2.34 | 0.021365 | 0.26883 | TOX | thymocyte selection-associated high mobility group box |
| 16848062 | -2.35 | 0.03147 | 0.311185 | C17orf58 | chromosome 17 open reading frame 58; NULL |
| 16673748 | -2.35 | 0.040405 | 0.347646 | FMO4 | flavin containing monooxygenase 4; NULL |
| 16968797 | -2.35 | 0.000464 | 0.055536 | HERC3 | HECT and RLD domain containing E3 ubiquitin protein ligase 3; NULL |
| 16745470 | -2.35 | 0.001597 | 0.089787 | MIR100HG | mir-100-let-7a-2 cluster host gene (non-protein coding) |
| 16747570 | -2.36 | 0.000334 | 0.048308 | ENO2 | enolase 2 (gamma, neuronal); NULL |
| 16682098 | -2.36 | 0.000185 | 0.036889 | EPHA2 | EPH receptor A2 |
| 17080468 | -2.36 | 0.000613 | 0.063216 | SAMD12 | sterile alpha motif domain containing 12 |
| 17094064 | -2.36 | 0.000262 | 0.043363 | SHB | Src homology 2 domain containing adaptor protein B |
| 16986138 | -2.37 | 0.046591 | 0.367179 | ARHGEF28 | Rho guanine nucleotide exchange factor (GEF) 28; NULL |
| 16700400 | -2.37 | 0.021109 | 0.266994 | C1orf198 | chromosome 1 open reading frame 198 |
| 16918351 | -2.37 | 0.001227 | 0.07992 | COMMD7 | COMM domain containing 7 |
| 16980470 | -2.37 | 0.000555 | 0.060129 | NR3C2 | nuclear receptor subfamily 3, group C, member 2; NULL |
| 16911835 | -2.37 | 0.013552 | 0.222525 | RIN2 | Ras and Rab interactor 2; NULL |
| 16884523 | -2.37 | 0.008396 | 0.184224 | SLC20A1 | solute carrier family 20 (phosphate transporter), member 1 |
| 16764724 | -2.37 | 0.030794 | 0.30856 | SMAGP | small cell adhesion glycoprotein |
| 16849379 | -2.38 | 0.036885 | 0.333921 | TK1 | thymidine kinase 1, soluble |
| 17004612 | -2.39 | 0.039771 | 0.3454 | DSP | desmoplakin |
| 16894283 | -2.39 | 0.00945 | 0.191824 | MBOAT2 | membrane bound O-acyltransferase domain containing 2; NULL |
| 17060167 | -2.39 | 0.039846 | 0.345734 | TMEM130 | transmembrane protein 130; NULL |
| 17106357 | -2.4 | 0.000338 | 0.048453 | PLS3 | plastin 3; NULL |
| 16899413 | -2.41 | 0.000689 | 0.065717 | EVA1A | eva-1 homolog A (C. elegans); NULL |
| 16958403 | -2.41 | 0.000413 | 0.052742 | HEG1 | heart development protein with EGF-like domains 1 |
| 16938378 | -2.41 | 0.000915 | 0.073272 | NR1D2 | nuclear receptor subfamily 1, group D, member 2 |
| 16854856 | -2.42 | 0.002867 | 0.115803 | EPG5 | ectopic P-granules autophagy protein 5 homolog (C. elegans) |
| 17088148 | -2.42 | 0.000457 | 0.055203 | SNX30 | sorting nexin family member 30 |
| 16732755 | -2.43 | 0.003213 | 0.1192 | GRAMD1B | GRAM domain containing 1B; NULL |
| 16817692 | -2.43 | 0.003257 | 0.119453 | MVP | major vault protein; NULL |
| 17063480 | -2.43 | 0.000955 | 0.074302 | PARP12 | poly (ADP-ribose) polymerase family, member 12; NULL |
| 17055354 | -2.44 | 0.008751 | 0.185507 | ETV1 | ets variant 1; NULL |
| 16741334 | -2.44 | 0.018695 | 0.255415 | MRGPRF | MAS-related GPR, member F |
| 16723614 | -2.45 | 0.00228 | 0.106246 | CD44 | CD44 molecule (Indian blood group); NULL |
| 16879791 | -2.45 | 0.00113 | 0.078119 | SOCS5 | suppressor of cytokine signaling 5 |
| 16851768 | -2.47 | 0.009025 | 0.187641 | DSG2 | desmoglein 2 |
| 16907488 | -2.47 | 0.010314 | 0.198175 | RAPH1 | Ras association (RalGDS/AF-6) and pleckstrin homology domains 1; NULL |
| 17025191 | -2.49 | 0.002831 | 0.115211 | EZR | ezrin |
| 17013851 | -2.5 | 0.001835 | 0.095784 | MYCT1 | myc target 1 |
| 16800229 | -2.51 | 0.000878 | 0.071509 | MAP1A | microtubule-associated protein 1A |
| 16960844 | -2.51 | 0.001973 | 0.098918 | VEPH1 | ventricular zone expressed PH domain-containing 1; NULL |
| 16802960 | -2.52 | 0.002044 | 0.100574 | ISLR | immunoglobulin superfamily containing leucine-rich repeat |
| 16844585 | -2.52 | 0.023382 | 0.278771 | KRTAP2-3; KRTAP2-4 | keratin associated protein 2-3; keratin associated protein 2-4 |
| 16823750 | -2.53 | 0.00642 | 0.163702 | CARHSP1 | calcium regulated heat stable protein 1, 24kDa; NULL |
| 16901393 | -2.53 | 0.000415 | 0.052787 | FHL2 | four and a half LIM domains 2 |
| 16907572 | -2.54 | 0.014094 | 0.227222 | GPR1 | G protein-coupled receptor 1; NULL |
| 16809748 | -2.54 | 0.00025 | 0.042689 | MNS1 | meiosis-specific nuclear structural 1 |
| 16820620 | -2.54 | 0.001672 | 0.09098 | WWP2 | WW domain containing E3 ubiquitin protein ligase 2; NULL |
| 16974121 | -2.55 | 0.000011 | 0.009196 | AFAP1 | actin filament associated protein 1 |
| 16724471 | -2.56 | 0.001984 | 0.099284 | DDB2 | damage-specific DNA binding protein 2, 48kDa |
| 16842834 | -2.56 | 0.001168 | 0.078347 | FLOT2 | flotillin 2 |
| 16689384 | -2.56 | 0.043132 | 0.356297 | GBP4 | guanylate binding protein 4 |
| 16968735 | -2.56 | 0.018103 | 0.252249 | HERC6 | HECT and RLD domain containing E3 ubiquitin protein ligase family member 6; NULL |
| 17007910 | -2.56 | 0.0039 | 0.130478 | MAPK13 | mitogen-activated protein kinase 13 |
| 16768738 | -2.56 | 0.002697 | 0.113112 | NTN4 | netrin 4 |
| 17019820 | -2.57 | 0.015795 | 0.239093 | PTCHD4 | patched domain containing 4 |
| 16705089 | -2.58 | 0.000825 | 0.069882 | BICC1 | bicaudal C homolog 1 (Drosophila) |
| 16879067 | -2.58 | 0.034002 | 0.321282 | CRIM1 | cysteine rich transmembrane BMP regulator 1 (chordin-like); NULL |
| 16714135 | -2.58 | 0.002987 | 0.116744 | ERCC6; ERCC6-PGBD3; PGBD3; OTTHUMG00000171334; RP11-123B3.6 | excision repair cross-complementing rodent repair deficiency, complementation group 6; ERCC6-PGBD3 readthrough; piggyBac transposable element derived 3; NULL |
| 16956149 | -2.58 | 0.000555 | 0.060129 | FOXP1 | forkhead box P1; NULL |
| 17087308 | -2.58 | 0.001302 | 0.082065 | TDRD7 | tudor domain containing 7 |
| 16788014 | -2.59 | 0.001938 | 0.09827 | C14orf132 | chromosome 14 open reading frame 132 |
| 16951696 | -2.59 | 0.012643 | 0.216055 | NEK10 | NIMA-related kinase 10; NULL |
| 16768149 | -2.59 | 0.001471 | 0.086859 | RASSF9 | Ras association (RalGDS/AF-6) domain family (N-terminal) member 9 |
| 16815310 | -2.59 | 0.002192 | 0.104314 | TNFRSF12A | tumor necrosis factor receptor superfamily, member 12A |
| 16735895 | -2.6 | 0.000501 | 0.057334 | DKK3 | dickkopf WNT signaling pathway inhibitor 3; NULL |
| 16938271 | -2.6 | 0.001771 | 0.093744 | KAT2B | K(lysine) acetyltransferase 2B; NULL |
| 16909958 | -2.6 | 0.000059 | 0.022513 | PER2 | period circadian clock 2 |
| 16894911 | -2.6 | 0.004689 | 0.144044 | SDC1 | syndecan 1 |
| 16756310 | -2.6 | 0.000229 | 0.041153 | TCP11L2 | t-complex 11, testis-specific-like 2 |
| 16803743 | -2.61 | 0.005855 | 0.158712 | ABHD17C | abhydrolase domain containing 17C |
| 16660785 | -2.61 | 0.00004 | 0.018213 | NIPAL3 | NIPA-like domain containing 3; NULL |
| 17019190 | -2.62 | 0.000007 | 0.007314 | C6orf132 | chromosome 6 open reading frame 132 |
| 16835672 | -2.62 | 0.028041 | 0.299608 | ITGA3 | integrin, alpha 3 (antigen CD49C, alpha 3 subunit of VLA-3 receptor); NULL |
| 17043529 | -2.63 | 0.001833 | 0.095765 | C1GALT1 | core 1 synthase, glycoprotein-N-acetylgalactosamine 3-beta-galactosyltransferase, 1; NULL |
| 16981266 | -2.63 | 0.000695 | 0.065717 | DDX60L | DEAD (Asp-Glu-Ala-Asp) box polypeptide 60-like; NULL |
| 16952912 | -2.63 | 0.005845 | 0.158656 | LRRC2 | leucine rich repeat containing 2 |
| 17024775 | -2.63 | 0.000124 | 0.031928 | SYNE1 | spectrin repeat containing, nuclear envelope 1; NULL |
| 16803710 | -2.64 | 0.000045 | 0.018827 | ARNT2 | aryl-hydrocarbon receptor nuclear translocator 2; NULL |
| 17092794 | -2.65 | 0.022436 | 0.274527 | PTPLAD2 | protein tyrosine phosphatase-like A domain containing 2 |
| 17019218 | -2.65 | 0.002585 | 0.110955 | TRERF1 | transcriptional regulating factor 1 |
| 16716507 | -2.66 | 0.039817 | 0.345732 | PPP1R3C | protein phosphatase 1, regulatory subunit 3C |
| 16677201 | -2.67 | 0.044014 | 0.358989 | DTL | denticleless E3 ubiquitin protein ligase homolog (Drosophila) |
| 16843167 | -2.67 | 0.04772 | 0.370656 | EVI2A | ecotropic viral integration site 2A |
| 16962632 | -2.68 | 0.002264 | 0.105781 | LEPREL1 | leprecan-like 1; NULL |
| 16844061 | -2.68 | 0.018152 | 0.252574 | PLXDC1 | plexin domain containing 1; NULL |
| 16948021 | -2.69 | 0.001467 | 0.086826 | ECT2 | epithelial cell transforming sequence 2 oncogene; NULL |
| 16768675 | -2.69 | 0.002155 | 0.103526 | FGD6 | FYVE, RhoGEF and PH domain containing 6 |
| 16785789 | -2.7 | 0.027648 | 0.29809 | GALNT16; GALNTL1 | UDP-N-acetyl-alpha-D-galactosamine:polypeptide N-acetylgalactosaminyltransferase 16; NULL |
| 16680790 | -2.7 | 0.011306 | 0.206945 | MEGF6 | multiple EGF-like-domains 6; NULL |
| 17066018 | -2.7 | 0.000444 | 0.05443 | ZDHHC2 | zinc finger, DHHC-type containing 2 |
| 16839294 | -2.71 | 0.012052 | 0.211809 | ABR | active BCR-related; NULL |
| 16888912 | -2.71 | 0.000003 | 0.004912 | MYO1B | myosin IB; NULL |
| 16693474 | -2.71 | 0.000939 | 0.074275 | S100A16 | S100 calcium binding protein A16; NULL |
| 16957396 | -2.72 | 0.003417 | 0.121919 | CCDC80 | coiled-coil domain containing 80 |
| 16833567 | -2.72 | 0.000048 | 0.019277 | DUSP14 | dual specificity phosphatase 14 |
| 16970404 | -2.72 | 0.006208 | 0.161217 | FGF2 | fibroblast growth factor 2 (basic) |
| 16769159 | -2.73 | 0.000265 | 0.043363 | GNPTAB | N-acetylglucosamine-1-phosphate transferase, alpha and beta subunits |
| 16873060 | -2.73 | 0.003085 | 0.117507 | PLAUR | plasminogen activator, urokinase receptor; NULL |
| 16836896 | -2.74 | 0.018671 | 0.255362 | ACE; OTTHUMG00000178900; CTD-2501B8.1; ACE3P | angiotensin I converting enzyme; NULL; angiotensin I converting enzyme (peptidyl-dipeptidase A) 3, pseudogene |
| 16818842 | -2.75 | 0.000046 | 0.019245 | CYLD | cylindromatosis (turban tumor syndrome); NULL |
| 16806870 | -2.76 | 0.000376 | 0.050487 | SLC12A6 | solute carrier family 12 (potassium/chloride transporters), member 6; NULL |
| 16683300 | -2.76 | 0.010108 | 0.197097 | TCEA3 | transcription elongation factor A (SII), 3 |
| 17050797 | -2.78 | 0.001667 | 0.09098 | CPED1 | cadherin-like and PC-esterase domain containing 1 |
| 16981219 | -2.78 | 0.006493 | 0.163944 | DDX60 | DEAD (Asp-Glu-Ala-Asp) box polypeptide 60 |
| 16767851 | -2.78 | 0.007892 | 0.179015 | E2F7 | E2F transcription factor 7; NULL |
| 16850517 | -2.79 | 0.031847 | 0.312229 | NDC80 | NDC80 kinetochore complex component |
| 16751319 | -2.79 | 0.000038 | 0.017393 | SCN8A | sodium channel, voltage gated, type VIII, alpha subunit |
| 16955291 | -2.8 | 0.020156 | 0.262731 | ARHGEF3 | Rho guanine nucleotide exchange factor (GEF) 3; NULL |
| 16970080 | -2.8 | 0.010537 | 0.200378 | CEP170P1 | centrosomal protein 170kDa pseudogene 1 |
| 16942648 | -2.8 | 0.006551 | 0.164497 | GXYLT2 | glucoside xylosyltransferase 2 |
| 16747184 | -2.81 | 0.00022 | 0.040103 | CD9 | CD9 molecule |
| 16786010 | -2.81 | 0.022745 | 0.27586 | SIPA1L1 | signal-induced proliferation-associated 1 like 1; NULL |
| 16951756 | -2.81 | 0.000897 | 0.072682 | SLC4A7 | solute carrier family 4, sodium bicarbonate cotransporter, member 7; NULL |
| 16830202 | -2.81 | 0.000246 | 0.042531 | XAF1 | XIAP associated factor 1; NULL |
| 17096213 | -2.82 | 0.005225 | 0.152166 | CDC14B | cell division cycle 14B; NULL |
| 16859795 | -2.83 | 0.003329 | 0.120505 | GDF15 | growth differentiation factor 15 |
| 16820104 | -2.85 | 0.046469 | 0.3666 | PLEKHG4 | pleckstrin homology domain containing, family G (with RhoGef domain) member 4; NULL |
| 16813342 | -2.85 | 0.041513 | 0.351534 | PRC1 | protein regulator of cytokinesis 1; NULL |
| 16977286 | -2.85 | 0.012893 | 0.218096 | PRKG2 | protein kinase, cGMP-dependent, type II; NULL |
| 16729789 | -2.85 | 0.002478 | 0.110337 | PRSS23 | protease, serine, 23; NULL |
| 16986409 | -2.86 | 0.029808 | 0.305434 | F2R | coagulation factor II (thrombin) receptor |
| 16851383 | -2.87 | 0.003887 | 0.130247 | GATA6 | GATA binding protein 6 |
| 17066224 | -2.87 | 0.000426 | 0.053538 | SH2D4A | SH2 domain containing 4A |
| 16670479 | -2.88 | 0.000674 | 0.065472 | C1orf51 | chromosome 1 open reading frame 51; NULL |
| 16823692 | -2.88 | 0.001775 | 0.093744 | NAGPA | N-acetylglucosamine-1-phosphodiester alpha-N-acetylglucosaminidase; NULL |
| 16984689 | -2.89 | 0.003725 | 0.128099 | ITGA2 | integrin, alpha 2 (CD49B, alpha 2 subunit of VLA-2 receptor); NULL |
| 17084904 | -2.9 | 0.01893 | 0.256875 | MELK | maternal embryonic leucine zipper kinase; NULL |
| 16677425 | -2.91 | 0.048911 | 0.375005 | CENPF | centromere protein F, 350/400kDa; NULL |
| 16685201 | -2.92 | 0.003483 | 0.123521 | COL8A2 | collagen, type VIII, alpha 2 |
| 16672390 | -2.92 | 0.010132 | 0.197179 | IFI16 | interferon, gamma-inducible protein 16; NULL |
| 16962022 | -2.92 | 0.005711 | 0.157882 | LAMP3 | lysosomal-associated membrane protein 3 |
| 16740412 | -2.94 | 0.001147 | 0.078213 | LTBP3 | latent transforming growth factor beta binding protein 3; NULL |
| 17042925 | -2.95 | 0.009091 | 0.18774 | ELFN1 | extracellular leucine-rich repeat and fibronectin type III domain containing 1 |
| 16904365 | -2.95 | 0.002104 | 0.10245 | IFIH1 | interferon induced with helicase C domain 1 |
| 16778241 | -2.97 | 0.008095 | 0.180579 | POSTN | periostin, osteoblast specific factor; NULL |
| 16679301 | -2.98 | 0.002296 | 0.106568 | FMN2 | formin 2; NULL |
| 16668002 | -3.01 | 0.02 | 0.262653 | FAM102B | family with sequence similarity 102, member B |
| 16972616 | -3.01 | 0.014431 | 0.230295 | NEIL3 | nei endonuclease VIII-like 3 (E. coli) |
| 17089003 | -3.01 | 0.039654 | 0.344944 | OLFML2A | olfactomedin-like 2A |
| 16788036 | -3.02 | 0.008924 | 0.186793 | BDKRB1 | bradykinin receptor B1 |
| 16719171 | -3.02 | 0.026102 | 0.290444 | CPXM2 | carboxypeptidase X (M14 family), member 2 |
| 17077826 | -3.02 | 0.005108 | 0.150221 | MYBL1; LOC645895 | v-myb myeloblastosis viral oncogene homolog (avian)-like 1; uncharacterized LOC645895 |
| 16764398 | -3.03 | 0.000953 | 0.074302 | FMNL3 | formin-like 3; NULL |
| 16738630 | -3.03 | 0.014439 | 0.230347 | LPXN | leupaxin |
| 16767794 | -3.03 | 0.000277 | 0.044266 | OSBPL8 | oxysterol binding protein-like 8; NULL |
| 16886491 | -3.05 | 0.030418 | 0.307432 | TNFAIP6 | tumor necrosis factor, alpha-induced protein 6 |
| 16979339 | -3.06 | 0.001203 | 0.079781 | PDE5A | phosphodiesterase 5A, cGMP-specific; NULL |
| 17056506 | -3.07 | 0.003561 | 0.12489 | RP9P | retinitis pigmentosa 9 pseudogene |
| 16692667 | -3.09 | 0.000844 | 0.070393 | MTMR11 | myotubularin related protein 11; NULL |
| 16968213 | -3.1 | 0.006665 | 0.165355 | ANXA3 | annexin A3; NULL |
| 17096904 | -3.11 | 0.001261 | 0.080211 | CTNNAL1 | catenin (cadherin-associated protein), alpha-like 1 |
| 16987610 | -3.13 | 0.002366 | 0.108484 | RGMB | RGM domain family, member B; NULL |
| 16805474 | -3.15 | 0.025134 | 0.287479 | ARRDC4 | arrestin domain containing 4 |
| 16807763 | -3.15 | 0.005306 | 0.153145 | EHD4 | EH-domain containing 4 |
| 16985229 | -3.15 | 0.009023 | 0.187641 | RGS7BP | regulator of G-protein signaling 7 binding protein |
| 16839177 | -3.16 | 0.004588 | 0.14274 | METRNL | meteorin, glial cell differentiation regulator-like |
| 16746290 | -3.17 | 0.02621 | 0.290906 | OPCML; LOC100653275 | opioid binding protein/cell adhesion molecule-like; NULL; uncharacterized LOC100653275 |
| 16934045 | -3.17 | 0.003935 | 0.131074 | PIK3IP1 | phosphoinositide-3-kinase interacting protein 1; NULL |
| 17016043 | -3.18 | 0.011904 | 0.210913 | MBOAT1 | membrane bound O-acyltransferase domain containing 1 |
| 16683445 | -3.2 | 0.006854 | 0.167743 | FUCA1 | fucosidase, alpha-L- 1, tissue |
| 16677451 | -3.2 | 0.008989 | 0.187344 | KCNK2 | potassium channel, subfamily K, member 2; NULL |
| 16684674 | -3.2 | 0.000741 | 0.067352 | MTMR9LP | myotubularin related protein 9-like, pseudogene; NULL |
| 17070061 | -3.21 | 0.013966 | 0.226019 | LY96 | lymphocyte antigen 96 |
| 16920730 | -3.22 | 0.000935 | 0.074275 | APCDD1L | adenomatosis polyposis coli down-regulated 1-like |
| 16775763 | -3.22 | 0.016493 | 0.24257 | MIR622 | microRNA 622 |
| 16739192 | -3.22 | 0.026431 | 0.291585 | RAB3IL1 | RAB3A interacting protein (rabin3)-like 1; NULL |
| 17059776 | -3.22 | 0.000168 | 0.035286 | SAMD9L | sterile alpha motif domain containing 9-like |
| 16909482 | -3.23 | 0.034312 | 0.322715 | HTR2B | 5-hydroxytryptamine (serotonin) receptor 2B, G protein-coupled |
| 16892075 | -3.24 | 0.002397 | 0.108938 | ARMC9 | armadillo repeat containing 9; NULL |
| 17115996 | -3.24 | 0.025368 | 0.288691 | KRT18P10 | NULL; keratin 18 pseudogene 10 |
| 16995645 | -3.25 | 0.000028 | 0.01535 | DAB2 | Dab, mitogen-responsive phosphoprotein, homolog 2 (Drosophila); NULL |
| 16962380 | -3.26 | 0.00049 | 0.056392 | ETV5 | ets variant 5; NULL |
| 16779435 | -3.27 | 0.000213 | 0.039639 | THSD1 | thrombospondin, type I, domain containing 1 |
| 16802519 | -3.28 | 0.002726 | 0.113587 | KIF23 | kinesin family member 23 |
| 16722081 | -3.29 | 0.000999 | 0.074723 | MICAL2 | microtubule associated monooxygenase, calponin and LIM domain containing 2; NULL |
| 16692834 | -3.3 | 0.049113 | 0.375894 | CTSS | cathepsin S |
| 16847432 | -3.33 | 0.043283 | 0.356774 | BRIP1 | BRCA1 interacting protein C-terminal helicase 1 |
| 17055937 | -3.34 | 0.000215 | 0.039716 | OSBPL3 | oxysterol binding protein-like 3; NULL |
| 16672214 | -3.37 | 0.007365 | 0.172592 | PEAR1 | platelet endothelial aggregation receptor 1; NULL |
| 17017018 | -3.38 | 0.003489 | 0.123634 | IER3 | immediate early response 3; NULL |
| 16902945 | -3.4 | 0.000098 | 0.028085 | NCKAP5 | NCK-associated protein 5 |
| 16929442 | -3.41 | 0.006704 | 0.165893 | TIMP3 | TIMP metallopeptidase inhibitor 3 |
| 16908171 | -3.42 | 0.007211 | 0.170944 | MARCH4 | membrane-associated ring finger (C3HC4) 4, E3 ubiquitin protein ligase |
| 16789149 | -3.44 | 0.00085 | 0.07076 | TNFAIP2 | tumor necrosis factor, alpha-induced protein 2; NULL |
| 17012546 | -3.46 | 0.024294 | 0.283686 | TMEM200A | transmembrane protein 200A |
| 16723662 | -3.48 | 0.004721 | 0.14447 | FJX1 | four jointed box 1 (Drosophila) |
| 16816542 | -3.48 | 0.006227 | 0.161455 | IQCK | IQ motif containing K; NULL |
| 16664828 | -3.48 | 0.015595 | 0.238374 | PODN | podocan; NULL |
| 16836492 | -3.48 | 0.039845 | 0.345734 | PRR11 | proline rich 11 |
| 16677071 | -3.48 | 0.010619 | 0.201064 | SERTAD4 | SERTA domain containing 4 |
| 17057478 | -3.51 | 0.000661 | 0.065086 | IGFBP3 | insulin-like growth factor binding protein 3; NULL |
| 16773759 | -3.55 | 0.011355 | 0.206945 | FRY | furry homolog (Drosophila) |
| 16658536 | -3.55 | 0.000003 | 0.004912 | PER3 | period circadian clock 3 |
| 16749459 | -3.55 | 0.000309 | 0.046466 | PPFIBP1 | PTPRF interacting protein, binding protein 1 (liprin beta 1); NULL |
| 16984730 | -3.57 | 0.012634 | 0.216053 | FST | follistatin |
| 16944695 | -3.6 | 0.00014 | 0.032437 | PARP14 | poly (ADP-ribose) polymerase family, member 14 |
| 16694701 | -3.61 | 0.000417 | 0.052787 | CRABP2 | cellular retinoic acid binding protein 2 |
| 16848123 | -3.62 | 0.001224 | 0.07992 | ABCA8 | ATP-binding cassette, sub-family A (ABC1), member 8; NULL |
| 17079210 | -3.64 | 0.015865 | 0.239696 | GEM | GTP binding protein overexpressed in skeletal muscle |
| 17114829 | -3.64 | 0.000025 | 0.014302 | IDS; OTTHUMG00000022618; AF011889.5 | iduronate 2-sulfatase; NULL |
| 16774427 | -3.64 | 0.006365 | 0.163225 | LACC1; CCDC122 | laccase (multicopper oxidoreductase) domain containing 1; coiled-coil domain containing 122 |
| 16754373 | -3.65 | 0.004454 | 0.14029 | GLIPR1 | GLI pathogenesis-related 1 |
| 16760048 | -3.67 | 0.012454 | 0.214446 | FOXM1 | forkhead box M1 |
| 16688799 | -3.68 | 0.008865 | 0.186045 | ELTD1 | EGF, latrophilin and seven transmembrane domain containing 1 |
| 16673126 | -3.68 | 0.004756 | 0.144841 | RGS4 | regulator of G-protein signaling 4; NULL |
| 16693996 | -3.68 | 0.001014 | 0.075104 | THBS3 | thrombospondin 3; NULL |
| 16960911 | -3.69 | 0.00326 | 0.119453 | LXN | latexin |
| 16862439 | -3.7 | 0.000023 | 0.013682 | AXL | AXL receptor tyrosine kinase |
| 16723422 | -3.71 | 0.000066 | 0.023625 | KIAA1549L | KIAA1549-like |
| 16852858 | -3.71 | 0.031277 | 0.310365 | SERPINB7 | serpin peptidase inhibitor, clade B (ovalbumin), member 7 |
| 16751655 | -3.75 | 0.00849 | 0.184735 | IGFBP6 | insulin-like growth factor binding protein 6 |
| 16952118 | -3.75 | 0.010616 | 0.201064 | TRANK1 | tetratricopeptide repeat and ankyrin repeat containing 1 |
| 16798919 | -3.76 | 0.011696 | 0.209669 | ARHGAP11A | Rho GTPase activating protein 11A |
| 16811085 | -3.76 | 0.018811 | 0.256075 | ITGA11 | integrin, alpha 11 |
| 16749423 | -3.77 | 0.004755 | 0.144841 | ARNTL2 | aryl hydrocarbon receptor nuclear translocator-like 2 |
| 16991472 | -3.77 | 0.005975 | 0.159828 | SGCD | sarcoglycan, delta (35kDa dystrophin-associated glycoprotein) |
| 16969686 | -3.8 | 0.000161 | 0.034724 | CCDC109B | coiled-coil domain containing 109B |
| 16865699 | -3.86 | 0.020493 | 0.264558 | SSC5D | scavenger receptor cysteine rich domain containing (5 domains) |
| 16965519 | -3.88 | 0.006763 | 0.166745 | SOD3 | superoxide dismutase 3, extracellular |
| 16782862 | -3.89 | 0.00064 | 0.064339 | NYNRIN | NYN domain and retroviral integrase containing |
| 16847841 | -3.96 | 0.000005 | 0.006355 | SMURF2 | SMAD specific E3 ubiquitin protein ligase 2; NULL |
| 16889563 | -3.98 | 0.001449 | 0.08636 | FZD7 | frizzled family receptor 7 |
| 16716371 | -3.99 | 0.025493 | 0.288999 | CH25H | cholesterol 25-hydroxylase |
| 16697196 | -4.03 | 0.007379 | 0.17266 | FAM129A | family with sequence similarity 129, member A |
| 16811638 | -4.03 | 0.000335 | 0.048308 | SEMA7A | semaphorin 7A, GPI membrane anchor (John Milton Hagen blood group) |
| 17056426 | -4.06 | 0.006734 | 0.166361 | PDE1C | phosphodiesterase 1C, calmodulin-dependent 70kDa |
| 16697544 | -4.07 | 0.016973 | 0.246273 | ASPM | asp (abnormal spindle) homolog, microcephaly associated (Drosophila) |
| 16784760 | -4.07 | 0.018437 | 0.254478 | DACT1 | dishevelled-binding antagonist of beta-catenin 1 |
| 16693976 | -4.07 | 0.002384 | 0.108938 | MUC1 | mucin 1, cell surface associated; NULL |
| 16903771 | -4.08 | 0.001231 | 0.07992 | CACNB4 | calcium channel, voltage-dependent, beta 4 subunit |
| 16717272 | -4.1 | 0.000177 | 0.036026 | LOXL4 | lysyl oxidase-like 4 |
| 16707180 | -4.11 | 0.017216 | 0.246731 | IFIT2 | interferon-induced protein with tetratricopeptide repeats 2 |
| 16821377 | -4.13 | 0.000778 | 0.067914 | CDH13 | cadherin 13, H-cadherin (heart); NULL |
| 17108003 | -4.16 | 0.007957 | 0.179483 | BGN | biglycan; NULL |
| 16844294 | -4.17 | 0.008732 | 0.185507 | NR1D1; THRA | nuclear receptor subfamily 1, group D, member 1; thyroid hormone receptor, alpha |
| 16696425 | -4.17 | 0.017094 | 0.246354 | TNFSF4 | tumor necrosis factor (ligand) superfamily, member 4 |
| 16859314 | -4.18 | 0.000663 | 0.065155 | KLF2 | Kruppel-like factor 2 (lung) |
| 16818773 | -4.23 | 0.000227 | 0.041041 | ADCY7 | adenylate cyclase 7; NULL |
| 16965252 | -4.23 | 0.000961 | 0.074302 | BST1 | bone marrow stromal cell antigen 1 |
| 16979985 | -4.23 | 0.000201 | 0.038238 | MGARP; NDUFC1 | mitochondria-localized glutamic acid-rich protein; NADH dehydrogenase (ubiquinone) 1, subcomplex unknown, 1, 6kDa |
| 16968331 | -4.25 | 0.001793 | 0.094347 | FGF5 | fibroblast growth factor 5 |
| 16818114 | -4.26 | 0.00433 | 0.138105 | HSD3B7 | hydroxy-delta-5-steroid dehydrogenase, 3 beta- and steroid delta-isomerase 7 |
| 17014114 | -4.31 | 0.000519 | 0.058563 | SYNJ2 | synaptojanin 2; NULL |
| 16675558 | -4.33 | 0.000638 | 0.064339 | NEK7 | NIMA-related kinase 7; NULL |
| 16901755 | -4.34 | 0.032004 | 0.312915 | BUB1 | BUB1 mitotic checkpoint serine/threonine kinase; NULL |
| 16909081 | -4.34 | 0.000717 | 0.066071 | DOCK10 | dedicator of cytokinesis 10; NULL |
| 16719515 | -4.38 | 0.022794 | 0.27586 | MKI67 | antigen identified by monoclonal antibody Ki-67 |
| 16802653 | -4.42 | 0.000129 | 0.031928 | THSD4 | thrombospondin, type I, domain containing 4 |
| 16859763 | -4.45 | 0.00003 | 0.016107 | IFI30; PIK3R2 | interferon, gamma-inducible protein 30; phosphoinositide-3-kinase, regulatory subunit 2 (beta) |
| 16794719 | -4.56 | 0.000432 | 0.053707 | LTBP2 | latent transforming growth factor beta binding protein 2 |
| 17000650 | -4.64 | 0.000006 | 0.006536 | TMEM173 | transmembrane protein 173; NULL |
| 16834409 | -4.67 | 0.000319 | 0.04744 | CNTNAP1 | contactin associated protein 1 |
| 17115782 | -4.73 | 0.030074 | 0.306613 | CLIC2 | chloride intracellular channel 2; NULL |
| 16690704 | -4.73 | 0.002221 | 0.105032 | SLC16A4 | solute carrier family 16, member 4 (monocarboxylic acid transporter 5); NULL |
| 16848097 | -4.78 | 0.013048 | 0.219401 | FAM20A | family with sequence similarity 20, member A; NULL |
| 17096471 | -4.78 | 0.000087 | 0.027195 | TBC1D2 | TBC1 domain family, member 2 |
| 16689354 | -4.83 | 0.00006 | 0.022697 | GBP2 | guanylate binding protein 2, interferon-inducible; NULL |
| 16922759 | -4.86 | 0.039343 | 0.344326 | KCNJ15 | potassium inwardly-rectifying channel, subfamily J, member 15; NULL |
| 16728261 | -4.97 | 0.001162 | 0.078347 | CCND1 | cyclin D1 |
| 16909021 | -4.97 | 0.002651 | 0.111971 | SERPINE2 | serpin peptidase inhibitor, clade E (nexin, plasminogen activator inhibitor type 1), member 2; NULL |
| 17083370 | -5.02 | 0.000608 | 0.063216 | PDCD1LG2 | programmed cell death 1 ligand 2 |
| 16835158 | -5.09 | 0.04881 | 0.374627 | ITGB3; OTTHUMG00000171957; RP11-290H9.2 | integrin, beta 3 (platelet glycoprotein IIIa, antigen CD61); NULL |
| 16920156 | -5.11 | 0.004736 | 0.144743 | PTGIS | prostaglandin I2 (prostacyclin) synthase |
| 16707196 | -5.13 | 0.00069 | 0.065717 | IFIT1 | interferon-induced protein with tetratricopeptide repeats 1 |
| 17016263 | -5.16 | 0.002599 | 0.111003 | CMAHP | cytidine monophospho-N-acetylneuraminic acid hydroxylase, pseudogene; NULL |
| 17089549 | -5.16 | 0.047796 | 0.3707 | DNM1 | dynamin 1 |
| 17049904 | -5.17 | 0.00038 | 0.050585 | LRRC17 | leucine rich repeat containing 17 |
| 16979225 | -5.28 | 0.00755 | 0.174632 | PRSS12 | protease, serine, 12 (neurotrypsin, motopsin) |
| 16811249 | -5.29 | 0.000034 | 0.016617 | UACA | uveal autoantigen with coiled-coil domains and ankyrin repeats; NULL |
| 16682333 | -5.31 | 0.000247 | 0.042531 | MFAP2 | microfibrillar-associated protein 2; NULL |
| 16967631 | -5.32 | 0.001855 | 0.096135 | SLC4A4 | solute carrier family 4, sodium bicarbonate cotransporter, member 4 |
| 17004903 | -5.42 | 0.011824 | 0.210727 | EDN1 | endothelin 1 |
| 16739479 | -5.43 | 0.036679 | 0.333185 | LRRN4CL | LRRN4 C-terminal like |
| 17007543 | -5.44 | 0.003363 | 0.121352 | ITPR3 | inositol 1,4,5-trisphosphate receptor, type 3 |
| 17033493 | -5.47 | 0.028381 | 0.300993 | C2; CFB | complement component 2; complement factor B; NULL |
| 17044177 | -5.54 | 0.002557 | 0.110864 | IL6 | interleukin 6 (interferon, beta 2); NULL |
| 16670574 | -5.55 | 0.000122 | 0.031794 | ECM1 | extracellular matrix protein 1; NULL |
| 16742384 | -5.59 | 0.022251 | 0.273537 | LRRC32 | leucine rich repeat containing 32 |
| 16797196 | -5.61 | 0.000362 | 0.049823 | AHNAK2 | AHNAK nucleoprotein 2 |
| 16779546 | -5.81 | 0.000244 | 0.042298 | DIAPH3 | diaphanous homolog 3 (Drosophila) |
| 17045622 | -5.83 | 0.000069 | 0.02411 | AEBP1; MIR4649 | AE binding protein 1; microRNA 4649; NULL |
| 16802903 | -5.9 | 0.000085 | 0.026948 | LOXL1 | lysyl oxidase-like 1 |
| 17040902 | -6.04 | 0.025532 | 0.288999 | C2 | complement component 2; NULL |
| 17083793 | -6.15 | 0.000065 | 0.023565 | ADAMTSL1 | ADAMTS-like 1; NULL |
| 17010760 | -6.42 | 0.000102 | 0.028712 | NT5E | 5'-nucleotidase, ecto (CD73); NULL |
| 16706180 | -6.43 | 0.003552 | 0.12489 | PLAU | plasminogen activator, urokinase |
| 17005138 | -6.64 | 0.002197 | 0.104373 | CAP2 | CAP, adenylate cyclase-associated protein, 2 (yeast) |
| 16785127 | -6.74 | 0.000005 | 0.006286 | RHOJ | ras homolog family member J |
| 16914972 | -6.75 | 0.000104 | 0.028751 | DOK5 | docking protein 5 |
| 16874005 | -6.82 | 1.32E-07 | 0.00091 | DBP | D site of albumin promoter (albumin D-box) binding protein |
| 16687123 | -6.86 | 0.00029 | 0.045501 | RAB3B | RAB3B, member RAS oncogene family |
| 17067314 | -6.93 | 0.049667 | 0.377773 | SCARA3 | scavenger receptor class A, member 3 |
| 16867784 | -6.94 | 0.000437 | 0.053853 | C3 | complement component 3; NULL |
| 17050591 | -6.98 | 0.010489 | 0.199819 | MET | met proto-oncogene (hepatocyte growth factor receptor) |
| 17062127 | -7.07 | 0.00164 | 0.090929 | WNT2 | wingless-type MMTV integration site family member 2 |
| 16715793 | -7.1 | 0.000025 | 0.014302 | KCNMA1 | potassium large conductance calcium-activated channel, subfamily M, alpha member 1; NULL |
| 17110401 | -7.12 | 0.000035 | 0.016644 | SLC9A7 | solute carrier family 9, subfamily A (NHE7, cation proton antiporter 7), member 7 |
| 16823666 | -7.13 | 0.047856 | 0.370986 | PPL | periplakin; NULL |
| 16974968 | -7.35 | 0.003659 | 0.126728 | SEL1L3 | sel-1 suppressor of lin-12-like 3 (C. elegans); NULL |
| 16904667 | -7.36 | 0.008387 | 0.184093 | SCN9A | sodium channel, voltage-gated, type IX, alpha subunit; NULL |
| 17063221 | -7.47 | 0.003847 | 0.129713 | FAM180A | family with sequence similarity 180, member A |
| 16697471 | -7.68 | 0.002878 | 0.11588 | B3GALT2 | UDP-Gal:betaGlcNAc beta 1,3-galactosyltransferase, polypeptide 2 |
| 17045198 | -7.81 | 0.018644 | 0.255362 | ANLN | anillin, actin binding protein; NULL |
| 16759218 | -7.97 | 0.000469 | 0.055836 | GPR133 | G protein-coupled receptor 133; NULL |
| 16755908 | -8.08 | 0.00064 | 0.064339 | DRAM1 | DNA-damage regulated autophagy modulator 1 |
| 16701037 | -8.33 | 0.022943 | 0.276214 | GREM2 | gremlin 2, DAN family BMP antagonist |
| 17007738 | -8.38 | 0.036096 | 0.330508 | SCUBE3 | signal peptide, CUB domain, EGF-like 3 |
| 17087430 | -8.51 | 0.000488 | 0.056386 | COL15A1 | collagen, type XV, alpha 1 |
| 17019698 | -8.77 | 0.038872 | 0.342339 | RCAN2 | regulator of calcineurin 2 |
| 16956792 | -8.78 | 0.000005 | 0.006299 | ABI3BP | ABI family, member 3 (NESH) binding protein |
| 17092870 | -8.8 | 0.00227 | 0.105953 | MIR31HG | MIR31 host gene (non-protein coding) |
| 17001299 | -9.01 | 0.000127 | 0.031928 | DPYSL3 | dihydropyrimidinase-like 3; NULL |
| 17017641 | -9.04 | 0.02493 | 0.286599 | TNXB; TNXA; LOC101060681 | tenascin XB; tenascin XA (pseudogene); tenascin-X-like; NULL |
| 16855510 | -9.13 | 0.000016 | 0.011572 | ATP8B1 | ATPase, aminophospholipid transporter, class I, type 8B, member 1 |
| 17076726 | -9.35 | 0.002973 | 0.116744 | PLAT | plasminogen activator, tissue; NULL |
| 17072135 | -9.55 | 0.009405 | 0.191464 | NOV | nephroblastoma overexpressed |
| 16853399 | -9.71 | 0.041328 | 0.350772 | COLEC12 | collectin sub-family member 12 |
| 16767422 | -9.84 | 0.000088 | 0.027195 | PTPRB | protein tyrosine phosphatase, receptor type, B; NULL |
| 16904324 | -10.04 | 0.000081 | 0.026433 | FAP | fibroblast activation protein, alpha; NULL |
| 17014257 | -10.23 | 0.007567 | 0.174846 | FNDC1 | fibronectin type III domain containing 1 |
| 16858137 | -10.32 | 0.000168 | 0.035286 | ICAM1 | intercellular adhesion molecule 1 |
| 17006949 | -10.51 | 0.012373 | 0.213961 | CFB; OTTHUMG00000159600; XXbac-BPG116M5.17; C2 | complement factor B; NULL |
| 17007982 | -12.32 | 0.00292 | 0.116487 | PI16 | peptidase inhibitor 16 |
| 16848173 | -12.35 | 0.016434 | 0.242368 | ABCA9 | ATP-binding cassette, sub-family A (ABC1), member 9; NULL |
| 17108816 | -12.37 | 0.002087 | 0.102189 | MXRA5 | matrix-remodelling associated 5 |
| 17084130 | -13.74 | 0.000011 | 0.009196 | TEK | TEK tyrosine kinase, endothelial |
| 16852179 | -15.26 | 0.011537 | 0.208586 | SLC14A1 | solute carrier family 14 (urea transporter), member 1 (Kidd blood group); NULL |
| 16962911 | -16.04 | 0.000103 | 0.028712 | LRRC15 | leucine rich repeat containing 15 |
| 17087413 | -17.39 | 0.000107 | 0.028986 | GALNT12 | UDP-N-acetyl-alpha-D-galactosamine:polypeptide N-acetylgalactosaminyltransferase 12 (GalNAc-T12) |
| 16904278 | -20.09 | 0.000019 | 0.012465 | DPP4 | dipeptidyl-peptidase 4; NULL |
| 17050765 | -20.57 | 0.00025 | 0.042689 | KCND2 | potassium voltage-gated channel, Shal-related subfamily, member 2 |
| 17080486 | -22.21 | 0.000139 | 0.032437 | TNFRSF11B | tumor necrosis factor receptor superfamily, member 11b |
| 16721585 | -23.78 | 0.013418 | 0.221455 | OLFML1 | olfactomedin-like 1 |
| 17095887 | -24.23 | 0.000002 | 0.003579 | ASPN | asporin |
| 16737344 | -28.13 | 0.00573 | 0.158095 | PAMR1 | peptidase domain containing associated with muscle regeneration 1 |
| 17072162 | -35.47 | 0.002142 | 0.103024 | COL14A1 | collagen, type XIV, alpha 1; NULL |
| 16803754 | -90.99 | 0.00001 | 0.00903 | KIAA1199 | KIAA1199; NULL |
