# Supplementary material for: Identification of transcription factors potentially involved in human adipogenesis in vitro
Source: Mol Genet Genomic Med. 2017 Mar 3;5(3):210–22. doi: 10.1002/mgg3.269 (PMC5441431; doi:10.1002/mgg3.269)
Supplement: Supplementary file 4 — Table S4. A complete list of differentially expressed genes on day 21. [file MGG3-5-210-s004.doc]

| **Up-regulated genes on day 21** | | |  |  |  |
| --- | --- | --- | --- | --- | --- |
|  |  |  |  |  |  |
| Transcript Cluster ID | Fold Change (linear) (Induced vs. Control) | ANOVA p-value (Induced vs. Control) | FDR p-value (Induced vs. Control) | Gene Symbol | Description |
| 17078592 | 560.05 | 5.11E-10 | 0.000025 | FABP4 | fatty acid binding protein 4, adipocyte; NULL |
| 16949397 | 313.28 | 3.46E-08 | 0.000288 | ADIPOQ | adiponectin, C1Q and collagen domain containing |
| 17021510 | 231.56 | 3.87E-08 | 0.000288 | CNR1 | cannabinoid receptor 1 (brain); NULL |
| 16729472 | 169.24 | 2.15E-08 | 0.000259 | THRSP | thyroid hormone responsive |
| 17059955 | 148.11 | 0.000002 | 0.004055 | PDK4 | pyruvate dehydrogenase kinase, isozyme 4 |
| 16722299 | 86.8 | 7.33E-08 | 0.000442 | PDE3B | phosphodiesterase 3B, cGMP-inhibited; NULL |
| 16834516 | 84.93 | 0.000008 | 0.012182 | AOC3 | amine oxidase, copper containing 3 |
| 16763182 | 80.62 | 1.02E-07 | 0.000494 | ABCD2 | ATP-binding cassette, sub-family D (ALD), member 2 |
| 16779958 | 63.34 | 0.000014 | 0.017462 | EDNRB | endothelin receptor type B |
| 17066278 | 62.3 | 1.07E-09 | 0.000026 | LPL | lipoprotein lipase; NULL |
| 16950609 | 61.49 | 2.20E-07 | 0.000886 | CIDEC | cell death-inducing DFFA-like effector c |
| 16813173 | 61.42 | 0.000005 | 0.009338 | PLIN1 | perilipin 1 |
| 16756649 | 53.13 | 0.000007 | 0.011783 | ACACB | acetyl-CoA carboxylase beta; NULL |
| 16716659 | 48.08 | 7.30E-07 | 0.002346 | RBP4 | retinol binding protein 4, plasma |
| 16774303 | 38.81 | 0.005186 | 0.206871 | RGCC | regulator of cell cycle |
| 16726065 | 38.05 | 6.98E-07 | 0.002346 | LGALS12 | lectin, galactoside-binding, soluble, 12 |
| 17102829 | 34.69 | 0.000016 | 0.017993 | MAOA | monoamine oxidase A |
| 16883690 | 32.31 | 0.00005 | 0.031485 | IL1RL1 | interleukin 1 receptor-like 1; NULL |
| 16767335 | 32.17 | 0.001519 | 0.131246 | CPM | carboxypeptidase M; NULL |
| 16706630 | 31.42 | 0.000003 | 0.005564 | FAM213A | family with sequence similarity 213, member A |
| 16751048 | 31.38 | 9.46E-09 | 0.000152 | GPD1 | glycerol-3-phosphate dehydrogenase 1 (soluble); NULL |
| 16821541 | 30.44 | 0.000514 | 0.088388 | CRISPLD2 | cysteine-rich secretory protein LCCL domain containing 2 |
| 17050154 | 29.95 | 0.000069 | 0.037179 | PRKAR2B | protein kinase, cAMP-dependent, regulatory, type II, beta |
| 17075589 | 29.94 | 0.000122 | 0.049363 | NEFL | neurofilament, light polypeptide |
| 16667530 | 28.82 | 0.000006 | 0.011783 | PALMD | palmdelphin |
| 16850958 | 26.68 | 0.00001 | 0.013154 | APCDD1 | adenomatosis polyposis coli down-regulated 1 |
| 17061759 | 26.63 | 0.000047 | 0.03027 | NRCAM | neuronal cell adhesion molecule; NULL |
| 16967875 | 26.44 | 0.000782 | 0.101677 | PARM1 | prostate androgen-regulated mucin-like protein 1 |
| 17113346 | 25.36 | 0.000405 | 0.08302 | CHRDL1 | chordin-like 1; NULL |
| 16676988 | 24.04 | 0.000517 | 0.088388 | HSD11B1 | hydroxysteroid (11-beta) dehydrogenase 1 |
| 16950825 | 23.51 | 0.00021 | 0.062198 | TIMP4 | TIMP metallopeptidase inhibitor 4 |
| 16718414 | 23.04 | 0.000088 | 0.043065 | GPAM | glycerol-3-phosphate acyltransferase, mitochondrial; NULL |
| 16872783 | 22.4 | 1.57E-07 | 0.00069 | LIPE | lipase, hormone-sensitive |
| 17093227 | 18.84 | 0.001046 | 0.115396 | AQP7; LOC100509620 | aquaporin 7; aquaporin-7-like; NULL |
| 16761938 | 18.77 | 0.000045 | 0.03027 | LMO3 | LIM domain only 3 (rhombotin-like 2); NULL |
| 17021489 | 18.36 | 0.000791 | 0.101829 | CNR1 | cannabinoid receptor 1 (brain); NULL |
| 16867326 | 17.58 | 0.000033 | 0.026305 | PLIN4 | perilipin 4 |
| 16819264 | 16.6 | 0.000003 | 0.00629 | MT1X | metallothionein 1X |
| 16978236 | 14.51 | 0.004007 | 0.186422 | ADH1B | alcohol dehydrogenase 1B (class I), beta polypeptide |
| 16909257 | 14.43 | 0.000073 | 0.037556 | SLC19A3 | solute carrier family 19, member 3; NULL |
| 16780929 | 13.96 | 0.019876 | 0.355808 | COL4A1 | collagen, type IV, alpha 1 |
| 16999041 | 12.36 | 0.000735 | 0.100117 | CDO1 | cysteine dioxygenase type 1 |
| 16982047 | 11.62 | 8.53E-07 | 0.002571 | ACSL1 | acyl-CoA synthetase long-chain family member 1; NULL |
| 16769250 | 11.46 | 0.014189 | 0.306444 | IGF1 | insulin-like growth factor 1 (somatomedin C) |
| 16743735 | 11.08 | 0.004963 | 0.201435 | MMP3 | matrix metallopeptidase 3 (stromelysin 1, progelatinase); NULL |
| 16819224 | 11.06 | 0.000712 | 0.099805 | MT1M | metallothionein 1M |
| 17070249 | 10.78 | 0.041485 | 0.463925 | STMN2 | stathmin-like 2 |
| 16829521 | 10.77 | 0.000226 | 0.063075 | TUSC5 | tumor suppressor candidate 5 |
| 16998551 | 10.61 | 0.00678 | 0.232581 | SLCO4C1 | solute carrier organic anion transporter family, member 4C1 |
| 16981099 | 10.26 | 0.001323 | 0.124819 | NPY1R | neuropeptide Y receptor Y1; NULL |
| 16709268 | 10.11 | 0.002951 | 0.171473 | ACSL5 | acyl-CoA synthetase long-chain family member 5; NULL |
| 16915182 | 9.91 | 0.000366 | 0.079233 | PCK1 | phosphoenolpyruvate carboxykinase 1 (soluble); NULL |
| 16751190 | 9.63 | 0.002351 | 0.154574 | METTL7A | methyltransferase like 7A; NULL |
| 16999475 | 9.34 | 0.000368 | 0.07933 | FBN2 | fibrillin 2 |
| 16871235 | 9.32 | 0.000054 | 0.03268 | CEBPA | CCAAT/enhancer binding protein (C/EBP), alpha |
| 16955197 | 8.9 | 0.002013 | 0.144922 | WNT5A | wingless-type MMTV integration site family, member 5A |
| 16739733 | 8.78 | 0.000068 | 0.037179 | HRASLS5 | HRAS-like suppressor family, member 5 |
| 16851309 | 8.54 | 0.000003 | 0.006766 | GREB1L | growth regulation by estrogen in breast cancer-like; NULL |
| 16763577 | 8.43 | 0.001063 | 0.115806 | SLC38A4 | solute carrier family 38, member 4 |
| 16966127 | 8.26 | 0.000002 | 0.00468 | KLB | klotho beta |
| 16850107 | 8.17 | 0.000125 | 0.049724 | FASN | fatty acid synthase |
| 16868481 | 8.13 | 0.000133 | 0.050589 | OLFM2 | olfactomedin 2; NULL |
| 17085951 | 7.88 | 0.001317 | 0.124819 | RORB | RAR-related orphan receptor B |
| 16671187 | 7.82 | 0.000623 | 0.094919 | NPR1 | natriuretic peptide receptor A/guanylate cyclase A (atrionatriuretic peptide receptor A) |
| 16977378 | 7.78 | 0.000228 | 0.063075 | TMEM150C | transmembrane protein 150C |
| 16846864 | 7.38 | 0.004039 | 0.186981 | MMD | monocyte to macrophage differentiation-associated |
| 16676983 | 7.33 | 0.00073 | 0.100117 | G0S2 | G0/G1switch 2 |
| 16937741 | 7.09 | 0.000137 | 0.050937 | PPARG | peroxisome proliferator-activated receptor gamma; NULL |
| 16720353 | 6.95 | 0.000095 | 0.045146 | PNPLA2 | patatin-like phospholipase domain containing 2; NULL |
| 16906346 | 6.87 | 0.004867 | 0.19962 | DIRC1 | disrupted in renal carcinoma 1 |
| 16834508 | 6.73 | 0.005766 | 0.218954 | AOC2 | amine oxidase, copper containing 2 (retina-specific) |
| 16796375 | 6.65 | 0.000401 | 0.08302 | CLMN | calmin (calponin-like, transmembrane); NULL |
| 16761820 | 6.62 | 0.001125 | 0.118715 | MGP | matrix Gla protein |
| 16958638 | 6.5 | 0.001593 | 0.133008 | KLF15 | Kruppel-like factor 15 |
| 16689869 | 6.48 | 0.008903 | 0.257533 | F3 | coagulation factor III (thromboplastin, tissue factor) |
| 16955822 | 6.45 | 0.001107 | 0.118176 | ADAMTS9 | ADAM metallopeptidase with thrombospondin type 1 motif, 9; NULL |
| 16711343 | 6.39 | 0.002185 | 0.150063 | AKR1C2; LOC101060798 | aldo-keto reductase family 1, member C2; aldo-keto reductase family 1 member C2-like; NULL |
| 17063005 | 6.22 | 0.000779 | 0.101677 | PLXNA4 | plexin A4 |
| 16947045 | 6.2 | 0.032245 | 0.426277 | AADAC | arylacetamide deacetylase |
| 16919022 | 6.17 | 0.002638 | 0.164945 | SAMHD1 | SAM domain and HD domain 1 |
| 16984304 | 6.15 | 0.000785 | 0.10172 | C7 | complement component 7; NULL |
| 16870453 | 6.15 | 0.016132 | 0.323208 | COMP | cartilage oligomeric matrix protein |
| 16991208 | 6.08 | 0.016464 | 0.326611 | GPX3 | glutathione peroxidase 3 (plasma) |
| 17101231 | 6.02 | 0.000041 | 0.029698 | GYG2 | glycogenin 2 |
| 16696979 | 5.93 | 0.000611 | 0.094788 | GLUL | glutamate-ammonia ligase; NULL |
| 16837348 | 5.93 | 0.003809 | 0.18515 | MAP2K6 | mitogen-activated protein kinase kinase 6 |
| 16841907 | 5.87 | 0.00849 | 0.252979 | RASD1 | RAS, dexamethasone-induced 1 |
| 16938133 | 5.83 | 0.016815 | 0.328877 | GALNT15 | UDP-N-acetyl-alpha-D-galactosamine:polypeptide N-acetylgalactosaminyltransferase 15 |
| 16947148 | 5.75 | 0.000026 | 0.022806 | ARHGEF26 | Rho guanine nucleotide exchange factor (GEF) 26 |
| 17080082 | 5.72 | 0.004827 | 0.198944 | ANGPT1 | angiopoietin 1 |
| 17075973 | 5.72 | 0.00021 | 0.062198 | DUSP4 | dual specificity phosphatase 4 |
| 16985518 | 5.58 | 0.000404 | 0.08302 | PIK3R1 | phosphoinositide-3-kinase, regulatory subunit 1 (alpha); NULL |
| 16708192 | 5.53 | 0.000327 | 0.077065 | ABCC2 | ATP-binding cassette, sub-family C (CFTR/MRP), member 2 |
| 16922443 | 5.52 | 0.006511 | 0.227552 | CLIC6 | chloride intracellular channel 6 |
| 16763032 | 5.51 | 0.011865 | 0.285615 | PKP2 | plakophilin 2; NULL |
| 16826738 | 5.5 | 0.00823 | 0.249932 | MT1G | metallothionein 1G |
| 16995771 | 5.49 | 0.001134 | 0.11889 | C6 | complement component 6; NULL |
| 16700888 | 5.48 | 0.000047 | 0.03027 | NID1 | nidogen 1 |
| 17077723 | 5.46 | 0.015995 | 0.321282 | CYP7B1 | cytochrome P450, family 7, subfamily B, polypeptide 1 |
| 16836021 | 5.25 | 0.008608 | 0.253915 | ABCC3 | ATP-binding cassette, sub-family C (CFTR/MRP), member 3; NULL |
| 16835816 | 5.17 | 0.000206 | 0.061716 | ACSF2 | acyl-CoA synthetase family member 2; NULL |
| 16819252 | 5.16 | 0.000275 | 0.06946 | MT1F | metallothionein 1F; NULL |
| 17093463 | 5.13 | 0.000282 | 0.070156 | CNTFR | ciliary neurotrophic factor receptor |
| 16997393 | 5.13 | 0.0085 | 0.252979 | ZBED3 | zinc finger, BED-type containing 3 |
| 17095056 | 5.07 | 0.00156 | 0.132829 | PRUNE2 | prune homolog 2 (Drosophila); NULL |
| 17003640 | 5.05 | 0.005186 | 0.206871 | ADAMTS2 | ADAM metallopeptidase with thrombospondin type 1 motif, 2 |
| 17012447 | 5.05 | 0.000153 | 0.053542 | LAMA2 | laminin, alpha 2 |
| 16991192 | 5.01 | 0.008937 | 0.257617 | GPX3 | glutathione peroxidase 3 (plasma); NULL |
| 16953597 | 4.96 | 0.000227 | 0.063075 | SLC26A6 | solute carrier family 26, member 6; NULL |
| 17058152 | 4.92 | 0.024496 | 0.388737 | ERV3-1; ZNF117 | endogenous retrovirus group 3, member 1; zinc finger protein 117 |
| 16778392 | 4.9 | 0.000007 | 0.011838 | FOXO1 | forkhead box O1 |
| 16776431 | 4.89 | 0.0271 | 0.40114 | COL4A2 | collagen, type IV, alpha 2; NULL |
| 17058524 | 4.81 | 0.001186 | 0.120248 | MLXIPL | MLX interacting protein-like; NULL |
| 16731441 | 4.77 | 0.000282 | 0.070156 | ZBTB16 | zinc finger and BTB domain containing 16; NULL |
| 17111501 | 4.76 | 0.00024 | 0.064653 | PFKFB1 | 6-phosphofructo-2-kinase/fructose-2,6-biphosphatase 1 |
| 16885135 | 4.73 | 0.005928 | 0.220911 | INHBB | inhibin, beta B |
| 16769761 | 4.71 | 0.000056 | 0.032692 | TMEM119 | transmembrane protein 119 |
| 16779667 | 4.69 | 0.005132 | 0.205718 | PCDH9 | protocadherin 9 |
| 16966733 | 4.69 | 0.000014 | 0.017462 | RASL11B | RAS-like, family 11, member B |
| 16912975 | 4.67 | 0.000152 | 0.053542 | ACSS2 | acyl-CoA synthetase short-chain family member 2; NULL |
| 16765625 | 4.66 | 0.000031 | 0.025551 | PPP1R1A | protein phosphatase 1, regulatory (inhibitor) subunit 1A |
| 17014798 | 4.66 | 0.002141 | 0.149115 | SMOC2 | SPARC related modular calcium binding 2 |
| 16711598 | 4.61 | 0.000495 | 0.088112 | ITIH5 | inter-alpha-trypsin inhibitor heavy chain family, member 5; NULL |
| 16743091 | 4.59 | 0.000462 | 0.086081 | FZD4 | frizzled family receptor 4 |
| 16888669 | 4.54 | 0.005971 | 0.221623 | MIR1245A; MIR1245B | microRNA 1245a; microRNA 1245b |
| 16765192 | 4.49 | 0.006204 | 0.224015 | CSAD | cysteine sulfinic acid decarboxylase; NULL |
| 17113147 | 4.49 | 0.000018 | 0.018287 | TSC22D3 | TSC22 domain family, member 3; NULL |
| 16883715 | 4.48 | 0.003187 | 0.176407 | IL18R1 | interleukin 18 receptor 1 |
| 16704320 | 4.42 | 0.003351 | 0.179209 | RASSF4 | Ras association (RalGDS/AF-6) domain family member 4; NULL |
| 16766946 | 4.4 | 0.003816 | 0.18515 | AVPR1A | arginine vasopressin receptor 1A |
| 16696177 | 4.4 | 0.040408 | 0.460364 | SLC19A2 | solute carrier family 19 (thiamine transporter), member 2 |
| 17114288 | 4.39 | 0.000418 | 0.084669 | GPC3 | glypican 3 |
| 16826639 | 4.37 | 0.001807 | 0.140432 | CES1; LOC100653057 | carboxylesterase 1; liver carboxylesterase 1-like; NULL |
| 17058142 | 4.36 | 0.012553 | 0.292542 | ZNF117 | zinc finger protein 117 |
| 16974830 | 4.35 | 0.009374 | 0.263225 | PPARGC1A | peroxisome proliferator-activated receptor gamma, coactivator 1 alpha; NULL |
| 16856299 | 4.34 | 0.00439 | 0.192247 | CFD | complement factor D (adipsin) |
| 16870443 | 4.29 | 0.002504 | 0.160999 | CRLF1 | cytokine receptor-like factor 1 |
| 16931000 | 4.23 | 0.000797 | 0.101829 | PNPLA3 | patatin-like phospholipase domain containing 3 |
| 16889268 | 4.21 | 0.000522 | 0.088587 | AOX1 | aldehyde oxidase 1; NULL |
| 16716795 | 4.18 | 0.006348 | 0.225536 | SORBS1; KIAA0894; RP11-476E15.3 | sorbin and SH3 domain containing 1; NULL |
| 16701975 | 4.14 | 0.003971 | 0.18621 | AKR1C1 | aldo-keto reductase family 1, member C1; NULL |
| 16842103 | 4.13 | 0.003238 | 0.177832 | SHMT1 | serine hydroxymethyltransferase 1 (soluble); NULL |
| 16820937 | 4.12 | 0.00593 | 0.220911 | HP | haptoglobin |
| 16928428 | 4.11 | 0.00267 | 0.165434 | ADRBK2 | adrenergic, beta, receptor kinase 2 |
| 16938630 | 4.11 | 0.000077 | 0.038847 | GPD1L | glycerol-3-phosphate dehydrogenase 1-like; NULL |
| 16834436 | 4.09 | 0.010445 | 0.273893 | RAMP2 | receptor (G protein-coupled) activity modifying protein 2 |
| 16785379 | 4.05 | 0.009102 | 0.259369 | HSPA2 | heat shock 70kDa protein 2 |
| 17048563 | 3.97 | 0.003069 | 0.17496 | PEG10 | paternally expressed 10 |
| 16739435 | 3.96 | 0.001564 | 0.132829 | C11orf48 | chromosome 11 open reading frame 48 |
| 16706906 | 3.95 | 0.009551 | 0.264424 | ADIRF; AGAP11 | adipogenesis regulatory factor; ankyrin repeat and GTPase domain Arf GTPase activating protein 11 |
| 16840846 | 3.94 | 0.010956 | 0.279103 | PER1 | period circadian clock 1 |
| 16668564 | 3.94 | 0.046257 | 0.474289 | PROK1 | prokineticin 1 |
| 16896561 | 3.87 | 0.001348 | 0.126499 | CYP1B1 | cytochrome P450, family 1, subfamily B, polypeptide 1 |
| 16879385 | 3.84 | 0.004348 | 0.192208 | PKDCC | protein kinase domain containing, cytoplasmic; NULL |
| 16819233 | 3.83 | 0.00854 | 0.253003 | MT1A | metallothionein 1A |
| 16890207 | 3.82 | 0.047691 | 0.478191 | MAP2 | microtubule-associated protein 2; NULL |
| 16936761 | 3.81 | 0.002945 | 0.171473 | CHL1 | cell adhesion molecule with homology to L1CAM (close homolog of L1); NULL |
| 16928204 | 3.81 | 0.000439 | 0.085609 | POM121L9P; LOC727983 | POM121 transmembrane nucleoporin-like 9, pseudogene; putative POM121-like protein 1-like |
| 16850069 | 3.78 | 0.000287 | 0.070736 | DCXR | dicarbonyl/L-xylulose reductase; NULL |
| 16696120 | 3.76 | 0.001437 | 0.129382 | DPT | dermatopontin |
| 17018497 | 3.76 | 0.000555 | 0.091352 | FKBP5; LOC285847 | FK506 binding protein 5; uncharacterized LOC285847 |
| 16666965 | 3.74 | 0.000447 | 0.085609 | LRRC8B | leucine rich repeat containing 8 family, member B |
| 16690566 | 3.74 | 0.011093 | 0.280404 | SORT1 | sortilin 1; NULL |
| 16969591 | 3.73 | 0.00002 | 0.019453 | HADH | hydroxyacyl-CoA dehydrogenase; NULL |
| 16760668 | 3.73 | 0.000682 | 0.098251 | LPCAT3 | lysophosphatidylcholine acyltransferase 3; NULL |
| 16975671 | 3.72 | 0.008227 | 0.249932 | CORIN | corin, serine peptidase; NULL |
| 16729812 | 3.72 | 0.00067 | 0.097254 | TMEM135 | transmembrane protein 135; NULL |
| 16681304 | 3.68 | 0.016151 | 0.323465 | ERRFI1 | ERBB receptor feedback inhibitor 1 |
| 16729290 | 3.68 | 0.001757 | 0.13938 | TSKU | tsukushi, small leucine rich proteoglycan |
| 16722278 | 3.67 | 0.022762 | 0.375805 | SPON1 | spondin 1, extracellular matrix protein |
| 16717816 | 3.66 | 0.000218 | 0.06303 | KCNIP2 | Kv channel interacting protein 2; NULL |
| 16978995 | 3.65 | 0.00299 | 0.172451 | ELOVL6 | ELOVL fatty acid elongase 6; NULL |
| 16733516 | 3.64 | 0.002891 | 0.169568 | ADAMTS15 | ADAM metallopeptidase with thrombospondin type 1 motif, 15 |
| 16933140 | 3.64 | 0.004594 | 0.195011 | GGT5 | gamma-glutamyltransferase 5 |
| 17021217 | 3.64 | 0.000841 | 0.103672 | ME1 | malic enzyme 1, NADP(+)-dependent, cytosolic |
| 16731169 | 3.63 | 0.000017 | 0.017993 | DLAT | dihydrolipoamide S-acetyltransferase; NULL |
| 16914478 | 3.62 | 0.000613 | 0.094788 | EYA2 | eyes absent homolog 2 (Drosophila) |
| 16757160 | 3.57 | 0.000434 | 0.085609 | ALDH2 | aldehyde dehydrogenase 2 family (mitochondrial) |
| 16836311 | 3.57 | 0.001241 | 0.122092 | NOG | noggin |
| 17071298 | 3.57 | 0.033288 | 0.430853 | OSR2 | odd-skipped related 2 (Drosophila); NULL |
| 17022623 | 3.56 | 0.002707 | 0.16557 | REV3L | REV3-like, polymerase (DNA directed), zeta, catalytic subunit; NULL |
| 16678114 | 3.55 | 0.000816 | 0.10258 | EPHX1 | epoxide hydrolase 1, microsomal (xenobiotic) |
| 16997399 | 3.55 | 0.003325 | 0.179209 | SNORA47 | small nucleolar RNA, H/ACA box 47 |
| 16749782 | 3.54 | 0.001362 | 0.127274 | FGD4 | FYVE, RhoGEF and PH domain containing 4; NULL |
| 17004167 | 3.54 | 0.000249 | 0.065627 | IRF4 | interferon regulatory factor 4 |
| 16696811 | 3.52 | 0.004538 | 0.194829 | ANGPTL1 | angiopoietin-like 1 |
| 17063461 | 3.5 | 0.003918 | 0.186035 | HIPK2 | homeodomain interacting protein kinase 2 |
| 17047795 | 3.49 | 0.037844 | 0.45273 | CD36 | CD36 molecule (thrombospondin receptor); NULL |
| 16771602 | 3.49 | 0.000026 | 0.022806 | HPD | 4-hydroxyphenylpyruvate dioxygenase |
| 16806564 | 3.47 | 0.000195 | 0.061132 | MTMR10 | myotubularin related protein 10; NULL |
| 16970435 | 3.47 | 0.037029 | 0.448458 | SPRY1 | sprouty homolog 1, antagonist of FGF signaling (Drosophila); NULL |
| 16984365 | 3.46 | 0.000591 | 0.093769 | GHR | growth hormone receptor; NULL |
| 16708249 | 3.46 | 0.002827 | 0.169272 | SCD | stearoyl-CoA desaturase (delta-9-desaturase) |
| 16979917 | 3.46 | 0.004328 | 0.191539 | SLC7A11 | solute carrier family 7 (anionic amino acid transporter light chain, xc- system), member 11 |
| 16906285 | 3.45 | 0.004267 | 0.190894 | CALCRL | calcitonin receptor-like; NULL |
| 16899634 | 3.44 | 0.000106 | 0.046189 | RETSAT | retinol saturase (all-trans-retinol 13,14-reductase) |
| 16741969 | 3.44 | 0.00004 | 0.029698 | UCP2 | uncoupling protein 2 (mitochondrial, proton carrier); NULL |
| 16723546 | 3.43 | 0.000351 | 0.078792 | CAT | catalase |
| 16737843 | 3.43 | 0.004438 | 0.193005 | LRP4 | low density lipoprotein receptor-related protein 4; NULL |
| 17061662 | 3.42 | 0.00147 | 0.130097 | LAMB1 | laminin, beta 1 |
| 16820398 | 3.41 | 0.000185 | 0.060278 | SLC7A6; SLC7A6OS | solute carrier family 7 (amino acid transporter light chain, y+L system), member 6; solute carrier family 7, member 6 opposite strand; NULL |
| 16851565 | 3.4 | 0.000445 | 0.085609 | TTC39C | tetratricopeptide repeat domain 39C |
| 16658864 | 3.39 | 0.001059 | 0.115806 | PGD | phosphogluconate dehydrogenase; NULL |
| 16761858 | 3.39 | 0.00669 | 0.230619 | RERG | RAS-like, estrogen-regulated, growth inhibitor; NULL |
| 16903953 | 3.38 | 0.00042 | 0.084669 | ACVR1C | activin A receptor, type IC |
| 17094459 | 3.35 | 0.022366 | 0.372845 | AQP7P1 | aquaporin 7 pseudogene 1 |
| 17104313 | 3.35 | 0.001829 | 0.141171 | AR | androgen receptor |
| 16964516 | 3.32 | 0.009144 | 0.259813 | ADRA2C | adrenoceptor alpha 2C |
| 16906175 | 3.31 | 0.001893 | 0.141171 | FRZB | frizzled-related protein |
| 16780917 | 3.29 | 0.000072 | 0.037556 | IRS2 | insulin receptor substrate 2 |
| 17087790 | 3.27 | 0.008415 | 0.252748 | SLC44A1 | solute carrier family 44, member 1 |
| 16739752 | 3.26 | 0.000092 | 0.044195 | PLA2G16 | phospholipase A2, group XVI |
| 16786255 | 3.25 | 0.003928 | 0.18621 | ACOT2 | acyl-CoA thioesterase 2 |
| 17096728 | 3.23 | 0.000048 | 0.030707 | ABCA1 | ATP-binding cassette, sub-family A (ABC1), member 1 |
| 16800242 | 3.23 | 0.000121 | 0.049363 | CKMT1B; CKMT1A | creatine kinase, mitochondrial 1B; creatine kinase, mitochondrial 1A; NULL |
| 16919418 | 3.23 | 0.000017 | 0.017993 | FITM2 | fat storage-inducing transmembrane protein 2 |
| 16803114 | 3.23 | 0.00447 | 0.193525 | SCAMP5 | secretory carrier membrane protein 5; NULL |
| 16994597 | 3.22 | 0.007164 | 0.23825 | FAM134B | family with sequence similarity 134, member B |
| 16986501 | 3.21 | 0.003157 | 0.175932 | PDE8B | phosphodiesterase 8B |
| 17002898 | 3.21 | 0.002279 | 0.153751 | STC2 | stanniocalcin 2 |
| 16880001 | 3.21 | 0.01026 | 0.272567 | STON1-GTF2A1L; STON1; GTF2A1L; FLJ46838 | STON1-GTF2A1L readthrough; stonin 1; general transcription factor IIA, 1-like; FLJ46838 protein; NULL |
| 16725783 | 3.19 | 0.000496 | 0.088112 | BEST1 | bestrophin 1 |
| 16758885 | 3.17 | 0.000194 | 0.061132 | AACS | acetoacetyl-CoA synthetase |
| 16790744 | 3.17 | 0.042484 | 0.466653 | SLC7A8 | solute carrier family 7 (amino acid transporter light chain, L system), member 8; NULL |
| 16745281 | 3.16 | 0.009108 | 0.259369 | MCAM | melanoma cell adhesion molecule; NULL |
| 17001846 | 3.15 | 0.002785 | 0.168525 | CCDC69 | coiled-coil domain containing 69; NULL |
| 16773453 | 3.15 | 0.000492 | 0.088112 | WASF3 | WAS protein family, member 3 |
| 16966712 | 3.14 | 0.000109 | 0.046783 | DANCR | differentiation antagonizing non-protein coding RNA; NULL |
| 16765697 | 3.12 | 0.001214 | 0.120488 | ITGA7 | integrin, alpha 7; NULL |
| 16855973 | 3.11 | 0.002037 | 0.145542 | CYB5A | cytochrome b5 type A (microsomal) |
| 16773493 | 3.11 | 0.003903 | 0.185897 | RASL11A | RAS-like, family 11, member A |
| 16914062 | 3.1 | 0.001107 | 0.118176 | TTPAL | tocopherol (alpha) transfer protein-like |
| 16859840 | 3.09 | 0.000952 | 0.109173 | TMEM59L; SYNGR1 | transmembrane protein 59-like; synaptogyrin 1; NULL |
| 16773165 | 3.08 | 0.027417 | 0.402202 | TNFRSF19 | tumor necrosis factor receptor superfamily, member 19 |
| 16913441 | 3.07 | 0.036944 | 0.44806 | NNAT | neuronatin |
| 16687618 | 3.05 | 0.006369 | 0.225536 | DHCR24 | 24-dehydrocholesterol reductase |
| 17016089 | 3.05 | 0.047795 | 0.478191 | PRL | prolactin |
| 17011217 | 3.04 | 0.000235 | 0.064143 | GRIK2 | glutamate receptor, ionotropic, kainate 2; NULL |
| 16831306 | 3.04 | 0.016693 | 0.328093 | HS3ST3B1 | heparan sulfate (glucosamine) 3-O-sulfotransferase 3B1 |
| 16832429 | 3.04 | 0.011263 | 0.281642 | TMEM97 | transmembrane protein 97 |
| 16819883 | 3.01 | 0.000567 | 0.091694 | PDP2 | pyruvate dehyrogenase phosphatase catalytic subunit 2; NULL |
| 16716782 | 3 | 0.027286 | 0.402164 | PDLIM1 | PDZ and LIM domain 1 |
| 16942270 | 3 | 0.000135 | 0.050589 | PTPRG | protein tyrosine phosphatase, receptor type, G; NULL |
| 16847933 | 2.99 | 0.033656 | 0.433372 | AXIN2; OTTHUMG00000179524; CTD-2535L24.2 | axin 2; NULL |
| 16729168 | 2.99 | 0.000058 | 0.032843 | DGAT2 | diacylglycerol O-acyltransferase 2 |
| 16822035 | 2.99 | 0.000263 | 0.067375 | DPEP1 | dipeptidase 1 (renal); NULL |
| 16843728 | 2.98 | 0.000723 | 0.100117 | ACACA | acetyl-CoA carboxylase alpha; NULL |
| 16705011 | 2.98 | 0.0128 | 0.295291 | DKK1 | dickkopf WNT signaling pathway inhibitor 1; NULL |
| 17092712 | 2.98 | 0.012115 | 0.288087 | PLIN2; LOC100509484 | perilipin 2; uncharacterized LOC100509484 |
| 17020152 | 2.97 | 0.000612 | 0.094788 | ELOVL5 | ELOVL fatty acid elongase 5; NULL |
| 16959386 | 2.97 | 0.000365 | 0.079233 | SLCO2A1 | solute carrier organic anion transporter family, member 2A1; NULL |
| 17046595 | 2.97 | 0.000636 | 0.094971 | VKORC1L1 | vitamin K epoxide reductase complex, subunit 1-like 1 |
| 16750190 | 2.96 | 0.005035 | 0.203718 | PDZRN4 | PDZ domain containing ring finger 4 |
| 16925983 | 2.95 | 0.001123 | 0.118715 | C2CD2 | C2 calcium-dependent domain containing 2; NULL |
| 16874156 | 2.94 | 0.00737 | 0.240825 | GYS1 | glycogen synthase 1 (muscle); NULL |
| 16852702 | 2.91 | 0.000002 | 0.003958 | CDH20 | cadherin 20, type 2 |
| 16743111 | 2.9 | 0.010141 | 0.270651 | CTSC | cathepsin C; NULL |
| 17069550 | 2.89 | 0.011267 | 0.281642 | ADHFE1 | alcohol dehydrogenase, iron containing, 1; NULL |
| 16854594 | 2.89 | 0.001913 | 0.141171 | GAREM | GRB2 associated, regulator of MAPK1 |
| 16731084 | 2.89 | 0.006233 | 0.224029 | SIK2 | salt-inducible kinase 2 |
| 16945870 | 2.87 | 0.020092 | 0.355977 | TF | transferrin; NULL |
| 17022996 | 2.86 | 0.000749 | 0.10036 | ROS1; GOPC | c-ros oncogene 1 , receptor tyrosine kinase; golgi-associated PDZ and coiled-coil motif containing |
| 16702935 | 2.85 | 0.004788 | 0.198128 | CACNB2 | calcium channel, voltage-dependent, beta 2 subunit; NULL |
| 16698356 | 2.85 | 0.003595 | 0.181198 | PIK3C2B | phosphatidylinositol-4-phosphate 3-kinase, catalytic subunit type 2 beta; NULL |
| 17090296 | 2.84 | 0.002155 | 0.149314 | ASS1 | argininosuccinate synthase 1; NULL |
| 17107907 | 2.84 | 0.003235 | 0.177832 | NSDHL | NAD(P) dependent steroid dehydrogenase-like; NULL |
| 17102512 | 2.84 | 0.003338 | 0.179209 | PRRG1 | proline rich Gla (G-carboxyglutamic acid) 1; NULL |
| 16932204 | 2.84 | 0.000777 | 0.101677 | SLC25A1 | solute carrier family 25 (mitochondrial carrier; citrate transporter), member 1; NULL |
| 16970465 | 2.83 | 0.00285 | 0.169272 | FAT4 | FAT atypical cadherin 4 |
| 16739132 | 2.8 | 0.000732 | 0.100117 | FADS1; MIR1908 | fatty acid desaturase 1; microRNA 1908; NULL |
| 16688506 | 2.79 | 0.007107 | 0.237198 | NEGR1 | neuronal growth regulator 1; NULL |
| 16968122 | 2.77 | 0.045011 | 0.471888 | FRAS1 | Fraser syndrome 1 |
| 16966137 | 2.77 | 0.001397 | 0.127922 | LIAS | lipoic acid synthetase |
| 16962620 | 2.76 | 0.038318 | 0.454701 | LPP-AS2 | LPP antisense RNA 2 |
| 16740914 | 2.76 | 0.000451 | 0.085609 | PC | pyruvate carboxylase; NULL |
| 17047459 | 2.7 | 0.018815 | 0.345355 | SNORA14A | small nucleolar RNA, H/ACA box 14A |
| 16919962 | 2.7 | 0.001516 | 0.131246 | SULF2 | sulfatase 2; NULL |
| 16879118 | 2.68 | 0.011119 | 0.280404 | GPATCH11 | G patch domain containing 11 |
| 16763138 | 2.67 | 0.041098 | 0.46341 | KIF21A | kinesin family member 21A |
| 16909303 | 2.67 | 0.001896 | 0.141171 | PID1 | phosphotyrosine interaction domain containing 1 |
| 17067231 | 2.67 | 0.000414 | 0.08416 | PTK2B | protein tyrosine kinase 2 beta; NULL |
| 16801707 | 2.66 | 0.000312 | 0.075348 | TLN2 | talin 2; NULL |
| 16830883 | 2.65 | 0.008304 | 0.251076 | ALOX15B | arachidonate 15-lipoxygenase, type B |
| 16855358 | 2.65 | 0.000811 | 0.10258 | CCDC68 | coiled-coil domain containing 68 |
| 17104363 | 2.65 | 0.03766 | 0.451865 | EFNB1 | ephrin-B1 |
| 16748788 | 2.64 | 0.000696 | 0.098968 | MGST1 | microsomal glutathione S-transferase 1; NULL |
| 16866849 | 2.64 | 0.025605 | 0.392387 | MKNK2 | MAP kinase interacting serine/threonine kinase 2; NULL |
| 16908154 | 2.63 | 0.000617 | 0.094793 | PECR | peroxisomal trans-2-enoyl-CoA reductase; NULL |
| 16766822 | 2.62 | 0.003417 | 0.179209 | LRIG3 | leucine-rich repeats and immunoglobulin-like domains 3 |
| 16844936 | 2.61 | 0.000505 | 0.088388 | ACLY | ATP citrate lyase; NULL |
| 16822014 | 2.61 | 0.001612 | 0.133596 | CPNE7 | copine VII |
| 16819229 | 2.59 | 0.004621 | 0.195997 | MT1JP | metallothionein 1J, pseudogene |
| 17019805 | 2.58 | 0.008907 | 0.257533 | TNFRSF21 | tumor necrosis factor receptor superfamily, member 21 |
| 16958573 | 2.57 | 0.000881 | 0.105116 | ALDH1L1 | aldehyde dehydrogenase 1 family, member L1; NULL |
| 16756202 | 2.57 | 0.021038 | 0.362745 | EID3 | EP300 interacting inhibitor of differentiation 3 |
| 16696614 | 2.57 | 0.014859 | 0.312334 | KIAA0040 | KIAA0040 |
| 16671264 | 2.57 | 0.000743 | 0.100169 | SLC27A3 | solute carrier family 27 (fatty acid transporter), member 3; NULL |
| 17106183 | 2.56 | 0.00011 | 0.046783 | TMEM164 | transmembrane protein 164 |
| 16996234 | 2.55 | 0.005545 | 0.214794 | PPAP2A | phosphatidic acid phosphatase type 2A; NULL |
| 17088760 | 2.55 | 0.026975 | 0.399898 | PTGS1 | prostaglandin-endoperoxide synthase 1 (prostaglandin G/H synthase and cyclooxygenase) |
| 16863115 | 2.54 | 0.00014 | 0.051374 | APOE; HMGA1 | apolipoprotein E; high mobility group AT-hook 1 |
| 16999321 | 2.54 | 0.028249 | 0.406889 | ZNF608 | zinc finger protein 608 |
| 16855184 | 2.53 | 0.006676 | 0.230363 | ACAA2 | acetyl-CoA acyltransferase 2 |
| 17068134 | 2.52 | 0.00113 | 0.118751 | TACC1 | transforming, acidic coiled-coil containing protein 1 |
| 16824352 | 2.52 | 0.002058 | 0.145974 | XYLT1 | xylosyltransferase I |
| 16986249 | 2.51 | 0.01085 | 0.278039 | HMGCR | 3-hydroxy-3-methylglutaryl-CoA reductase; NULL |
| 17100087 | 2.5 | 0.000912 | 0.107035 | AGPAT2 | 1-acylglycerol-3-phosphate O-acyltransferase 2 |
| 17005573 | 2.5 | 0.003217 | 0.177494 | HIST1H2BD | histone cluster 1, H2bd |
| 16851486 | 2.5 | 0.003324 | 0.179209 | LAMA3 | laminin, alpha 3 |
| 16843049 | 2.5 | 0.008438 | 0.252748 | SSH2 | slingshot protein phosphatase 2 |
| 16906733 | 2.5 | 0.001851 | 0.141171 | STK17B | serine/threonine kinase 17b |
| 16990862 | 2.49 | 0.01454 | 0.310395 | ABLIM3 | actin binding LIM protein family, member 3; NULL |
| 17014309 | 2.49 | 0.031311 | 0.42293 | ACAT2; LOC100129518; SOD2 | acetyl-CoA acetyltransferase 2; uncharacterized LOC100129518; superoxide dismutase 2, mitochondrial |
| 16946707 | 2.49 | 0.043579 | 0.46786 | AGTR1 | angiotensin II receptor, type 1 |
| 16894491 | 2.48 | 0.001307 | 0.124787 | ROCK2 | Rho-associated, coiled-coil containing protein kinase 2; NULL |
| 17005894 | 2.48 | 0.003353 | 0.179209 | ZSCAN12P1 | zinc finger and SCAN domain containing 12 pseudogene 1 |
| 16908618 | 2.47 | 0.000811 | 0.10258 | ABCB6; ATG9A | ATP-binding cassette, sub-family B (MDR/TAP), member 6; autophagy related 9A; NULL |
| 16836292 | 2.47 | 0.026812 | 0.398649 | ANKFN1 | ankyrin-repeat and fibronectin type III domain containing 1 |
| 16856604 | 2.47 | 0.003143 | 0.175763 | REEP6 | receptor accessory protein 6 |
| 17118378 | 2.46 | 0.014187 | 0.306444 | FAM27E3; FAM27E2 | family with sequence similarity 27, member E3; family with sequence similarity 27, member E2 |
| 17103327 | 2.44 | 0.030642 | 0.419353 | EBP | emopamil binding protein (sterol isomerase); NULL |
| 16908897 | 2.44 | 0.000845 | 0.103688 | EPHA4 | EPH receptor A4; NULL |
| 16706499 | 2.44 | 0.029989 | 0.415122 | PPIF | peptidylprolyl isomerase F |
| 16789953 | 2.43 | 0.007405 | 0.240864 | SNORD126 | small nucleolar RNA, C/D box 126 |
| 16708179 | 2.42 | 0.002198 | 0.150576 | CUTC | cutC copper transporter homolog (E. coli); NULL |
| 17076063 | 2.42 | 0.000914 | 0.107035 | GSR | glutathione reductase |
| 16829139 | 2.42 | 0.010368 | 0.273224 | MVD | mevalonate (diphospho) decarboxylase; NULL |
| 17114970 | 2.39 | 0.020884 | 0.361892 | GABRE; MIR452; MIR224 | gamma-aminobutyric acid (GABA) A receptor, epsilon; microRNA 452; microRNA 224; NULL |
| 16888963 | 2.39 | 0.022419 | 0.373106 | NABP1 | nucleic acid binding protein 1; NULL |
| 16813189 | 2.39 | 0.01075 | 0.277446 | PEX11A | peroxisomal biogenesis factor 11 alpha |
| 16748989 | 2.38 | 0.00113 | 0.118751 | PDE3A | phosphodiesterase 3A, cGMP-inhibited |
| 17060049 | 2.37 | 0.012529 | 0.292542 | DLX5 | distal-less homeobox 5 |
| 16895673 | 2.37 | 0.000379 | 0.080081 | SLC5A6 | solute carrier family 5 (sodium-dependent vitamin transporter), member 6; NULL |
| 17024144 | 2.35 | 0.000707 | 0.099508 | IFNGR1 | interferon gamma receptor 1; NULL |
| 16909165 | 2.35 | 0.007363 | 0.240825 | IRS1 | insulin receptor substrate 1 |
| 16699877 | 2.35 | 0.013005 | 0.297075 | LBR | lamin B receptor; NULL |
| 16987766 | 2.34 | 0.011386 | 0.282168 | C5orf30 | chromosome 5 open reading frame 30; NULL |
| 16909319 | 2.34 | 0.00198 | 0.144028 | DNER | delta/notch-like EGF repeat containing |
| 16753670 | 2.34 | 0.006948 | 0.234881 | IRAK3 | interleukin-1 receptor-associated kinase 3 |
| 16779855 | 2.34 | 0.00185 | 0.141171 | MYCBP2 | MYC binding protein 2, E3 ubiquitin protein ligase |
| 16845249 | 2.34 | 0.004389 | 0.192247 | RAMP2-AS1 | RAMP2 antisense RNA 1 |
| 16920150 | 2.32 | 0.000014 | 0.017462 | KCNB1 | potassium voltage-gated channel, Shab-related subfamily, member 1 |
| 16696504 | 2.31 | 0.020046 | 0.355977 | GAS5; SNORD77; SNORD76; SNORD44; SNORD47; SNORD80; SNORD79; SNORD81 | growth arrest-specific 5 (non-protein coding); small nucleolar RNA, C/D box 77; small nucleolar RNA, C/D box 76; small nucleolar RNA, C/D box 44; small nucleolar RNA, C/D box 47; small nucleolar RNA, C/D box 80; small nucleolar RNA, C/D box 79; small nucleolar RNA, C/D box 81; NULL |
| 17088124 | 2.31 | 0.01849 | 0.342566 | HSDL2 | hydroxysteroid dehydrogenase like 2 |
| 16922134 | 2.31 | 0.010189 | 0.271473 | MRAP | melanocortin 2 receptor accessory protein |
| 16819217 | 2.3 | 0.002517 | 0.160999 | MT1E | metallothionein 1E |
| 17102129 | 2.3 | 0.004792 | 0.198128 | SAT1 | spermidine/spermine N1-acetyltransferase 1; NULL |
| 17095150 | 2.3 | 0.004578 | 0.194961 | TLE1 | transducin-like enhancer of split 1 (E(sp1) homolog, Drosophila); NULL |
| 16831313 | 2.29 | 0.000306 | 0.074145 | MGC12916 | uncharacterized protein MGC12916 |
| 16722720 | 2.29 | 0.018667 | 0.344001 | NAV2 | neuron navigator 2; NULL |
| 16849992 | 2.29 | 0.011526 | 0.283905 | PCYT2 | phosphate cytidylyltransferase 2, ethanolamine; NULL |
| 16702431 | 2.28 | 0.038214 | 0.454212 | ECHDC3 | enoyl CoA hydratase domain containing 3 |
| 16936947 | 2.28 | 0.000652 | 0.095854 | ITPR1 | inositol 1,4,5-trisphosphate receptor, type 1; NULL |
| 17099705 | 2.28 | 0.000727 | 0.100117 | MIR3689F | microRNA 3689f |
| 17025440 | 2.28 | 0.01307 | 0.297618 | PARK2 | parkinson protein 2, E3 ubiquitin protein ligase (parkin) |
| 16939815 | 2.27 | 0.001401 | 0.127922 | ABHD5 | abhydrolase domain containing 5; NULL |
| 16690103 | 2.27 | 0.000628 | 0.094971 | DBT | dihydrolipoamide branched chain transacylase E2 |
| 16995890 | 2.27 | 0.044992 | 0.471888 | HMGCS1 | 3-hydroxy-3-methylglutaryl-CoA synthase 1 (soluble); NULL |
| 16704351 | 2.27 | 0.007198 | 0.23825 | ZNF22 | zinc finger protein 22 |
| 17109042 | 2.26 | 0.015165 | 0.315093 | MID1 | midline 1 (Opitz/BBB syndrome) |
| 16947173 | 2.26 | 0.015656 | 0.3183 | MME | membrane metallo-endopeptidase; NULL |
| 16913537 | 2.25 | 0.041753 | 0.464815 | LBP | lipopolysaccharide binding protein |
| 16723680 | 2.25 | 0.008475 | 0.252979 | LDLRAD3 | low density lipoprotein receptor class A domain containing 3 |
| 16682771 | 2.25 | 0.025461 | 0.39161 | PLA2G2A | phospholipase A2, group IIA (platelets, synovial fluid); NULL |
| 17109432 | 2.25 | 0.00181 | 0.140432 | RAI2 | retinoic acid induced 2 |
| 16884187 | 2.25 | 0.012672 | 0.294087 | SH3RF3 | SH3 domain containing ring finger 3 |
| 16949792 | 2.24 | 0.02193 | 0.369793 | FAM43A | family with sequence similarity 43, member A |
| 16693082 | 2.24 | 0.028374 | 0.406889 | SELENBP1 | selenium binding protein 1; NULL |
| 16834523 | 2.23 | 0.039287 | 0.456858 | AOC4 | amine oxidase, copper containing 3 (vascular adhesion protein 1) pseudogene |
| 16705620 | 2.22 | 0.019565 | 0.35258 | TSPAN15 | tetraspanin 15; NULL |
| 16664569 | 2.21 | 0.005533 | 0.214794 | CDKN2C | cyclin-dependent kinase inhibitor 2C (p18, inhibits CDK4) |
| 16949785 | 2.21 | 0.010351 | 0.273076 | TMEM44-AS1 | TMEM44 antisense RNA 1 |
| 16832147 | 2.2 | 0.005765 | 0.218954 | ALDH3A2 | aldehyde dehydrogenase 3 family, member A2; NULL |
| 17082548 | 2.2 | 0.000445 | 0.085609 | DGAT1 | diacylglycerol O-acyltransferase 1; NULL |
| 16665346 | 2.2 | 0.005697 | 0.217551 | NFIA | nuclear factor I/A; NULL |
| 16852308 | 2.2 | 0.004008 | 0.186422 | SCARNA17 | small Cajal body-specific RNA 17 |
| 16706641 | 2.2 | 0.044984 | 0.471888 | TSPAN14 | tetraspanin 14 |
| 16941389 | 2.19 | 0.000007 | 0.011838 | GLYCTK | glycerate kinase |
| 16877941 | 2.19 | 0.000744 | 0.100169 | KCNK3 | potassium channel, subfamily K, member 3 |
| 16937725 | 2.19 | 0.020749 | 0.361247 | SYN2 | synapsin II |
| 17075698 | 2.19 | 0.003418 | 0.179209 | TRIM35 | tripartite motif containing 35 |
| 16937440 | 2.18 | 0.000393 | 0.082856 | IL17RC | interleukin 17 receptor C; NULL |
| 16918496 | 2.17 | 0.00013 | 0.050589 | AHCY | adenosylhomocysteinase |
| 16834273 | 2.17 | 0.002048 | 0.145657 | STAT5A | signal transducer and activator of transcription 5A; NULL |
| 17002278 | 2.16 | 0.047841 | 0.478191 | EBF1 | early B-cell factor 1; NULL |
| 16850759 | 2.16 | 0.000332 | 0.077764 | PTPRM | protein tyrosine phosphatase, receptor type, M |
| 17061467 | 2.16 | 0.006929 | 0.234481 | PUS7 | pseudouridylate synthase 7 homolog (S. cerevisiae); NULL |
| 16959007 | 2.15 | 0.004859 | 0.19962 | PLXND1 | plexin D1; NULL |
| 16953753 | 2.14 | 0.007163 | 0.23825 | SLC25A20 | solute carrier family 25 (carnitine/acylcarnitine translocase), member 20; NULL |
| 16988376 | 2.13 | 0.00292 | 0.170714 | HSD17B4 | hydroxysteroid (17-beta) dehydrogenase 4; NULL |
| 16684192 | 2.13 | 0.007044 | 0.236235 | SNORD99 | small nucleolar RNA, C/D box 99 |
| 17106688 | 2.12 | 0.033425 | 0.432037 | GRIA3 | glutamate receptor, ionotropic, AMPA 3; NULL |
| 16836528 | 2.12 | 0.007654 | 0.243316 | YPEL2 | yippee-like 2 (Drosophila); NULL |
| 16677698 | 2.11 | 0.001737 | 0.138916 | MARC1 | mitochondrial amidoxime reducing component 1; NULL |
| 16934476 | 2.11 | 0.015555 | 0.317973 | APOL2 | apolipoprotein L, 2; NULL |
| 16696187 | 2.11 | 0.026191 | 0.395461 | F5 | coagulation factor V (proaccelerin, labile factor) |
| 16883647 | 2.11 | 0.041853 | 0.464943 | IL1R1 | interleukin 1 receptor, type I; NULL |
| 17067011 | 2.11 | 0.028266 | 0.406889 | NEFM | neurofilament, medium polypeptide |
| 16763467 | 2.11 | 0.009295 | 0.26254 | PLEKHA8P1 | pleckstrin homology domain containing, family A member 8 pseudogene 1 |
| 16830302 | 2.1 | 0.001258 | 0.122821 | ACADVL | acyl-CoA dehydrogenase, very long chain; NULL |
| 16918679 | 2.1 | 0.012555 | 0.292542 | EDEM2; MMP24-AS1; OTTHUMG00000176080; RP4-614O4.11 | ER degradation enhancer, mannosidase alpha-like 2; MMP24 antisense RNA 1; NULL |
| 16987395 | 2.1 | 0.002313 | 0.154574 | RHOBTB3 | Rho-related BTB domain containing 3; NULL |
| 16677683 | 2.09 | 0.036412 | 0.444938 | MARC2 | mitochondrial amidoxime reducing component 2 |
| 16821239 | 2.09 | 0.008238 | 0.249932 | CENPN | centromere protein N |
| 16800980 | 2.09 | 0.00947 | 0.263992 | GABPB1-AS1; GABPB1 | GABPB1 antisense RNA 1; GA binding protein transcription factor, beta subunit 1; NULL |
| 16834091 | 2.09 | 0.001905 | 0.141171 | IGFBP4 | insulin-like growth factor binding protein 4 |
| 16891564 | 2.09 | 0.001943 | 0.142484 | MOGAT1 | monoacylglycerol O-acyltransferase 1 |
| 17049270 | 2.09 | 0.029804 | 0.414882 | PILRB | paired immunoglobin-like type 2 receptor beta; NULL |
| 17103303 | 2.09 | 0.035325 | 0.440693 | PORCN | porcupine homolog (Drosophila); NULL |
| 16990678 | 2.09 | 0.019536 | 0.35258 | STK32A | serine/threonine kinase 32A; NULL |
| 16752103 | 2.08 | 0.003947 | 0.18621 | PDE1B | phosphodiesterase 1B, calmodulin-dependent; NULL |
| 17004859 | 2.08 | 0.021578 | 0.366961 | TMEM170B | transmembrane protein 170B |
| 16835972 | 2.07 | 0.004683 | 0.196792 | CACNA1G | calcium channel, voltage-dependent, T type, alpha 1G subunit; NULL |
| 16884967 | 2.07 | 0.007724 | 0.243316 | DBI | diazepam binding inhibitor (GABA receptor modulator, acyl-CoA binding protein); NULL |
| 16861887 | 2.07 | 0.049399 | 0.482545 | ECH1 | enoyl CoA hydratase 1, peroxisomal |
| 16795664 | 2.07 | 0.000459 | 0.086081 | FOXN3; OTTHUMG00000170977; RP11-33N16.3 | forkhead box N3; NULL |
| 16757324 | 2.07 | 0.04891 | 0.481176 | OAS1 | 2'-5'-oligoadenylate synthetase 1, 40/46kDa; NULL |
| 17078558 | 2.07 | 0.038341 | 0.454868 | PAG1 | phosphoprotein associated with glycosphingolipid microdomains 1; NULL |
| 16813206 | 2.06 | 0.025431 | 0.39161 | ANPEP | alanyl (membrane) aminopeptidase; NULL |
| 16868489 | 2.06 | 0.001447 | 0.1295 | COL5A3 | collagen, type V, alpha 3 |
| 16818610 | 2.06 | 0.001292 | 0.124113 | GPT2 | glutamic pyruvate transaminase (alanine aminotransferase) 2 |
| 16772144 | 2.06 | 0.004374 | 0.192247 | SCARB1 | scavenger receptor class B, member 1; NULL |
| 16729298 | 2.05 | 0.026817 | 0.398649 | ACER3 | alkaline ceramidase 3; NULL |
| 16766001 | 2.05 | 0.005687 | 0.217486 | CS | citrate synthase; NULL |
| 16762146 | 2.05 | 0.016553 | 0.327569 | KCNJ8 | potassium inwardly-rectifying channel, subfamily J, member 8; NULL |
| 16818520 | 2.05 | 0.021913 | 0.369635 | LINC00273 | long intergenic non-protein coding RNA 273 |
| 16673945 | 2.05 | 0.010538 | 0.275197 | PRDX6 | peroxiredoxin 6; NULL |
| 17097211 | 2.05 | 0.006624 | 0.229582 | PTGR1 | prostaglandin reductase 1 |
| 16870821 | 2.05 | 0.030427 | 0.417884 | ZNF100 | zinc finger protein 100 |
| 16795965 | 2.04 | 0.001243 | 0.122105 | FBLN5 | fibulin 5; NULL |
| 17020715 | 2.04 | 0.044293 | 0.469876 | LINC00472 | long intergenic non-protein coding RNA 472 |
| 16707030 | 2.04 | 0.003678 | 0.181711 | PTEN | phosphatase and tensin homolog |
| 16692624 | 2.03 | 0.033236 | 0.430409 | HIST2H2BC | histone cluster 2, H2bc (pseudogene) |
| 16894139 | 2.03 | 0.033109 | 0.429449 | RNF144A-AS1 | RNF144A antisense RNA 1; NULL |
| 16815955 | 2.03 | 0.005115 | 0.205584 | SHISA9 | shisa homolog 9 (Xenopus laevis); NULL |
| 16966721 | 2.03 | 0.002873 | 0.169559 | SNORA26 | small nucleolar RNA, H/ACA box 26 |
| 16814498 | 2.02 | 0.006835 | 0.232909 | METRN | meteorin, glial cell differentiation regulator |
| 16698816 | 2.02 | 0.007753 | 0.24342 | PLXNA2 | plexin A2; NULL |
| 16775898 | 2.02 | 0.000149 | 0.053542 | RAP2A | RAP2A, member of RAS oncogene family |
| 16849652 | 2.02 | 0.020332 | 0.358204 | TBC1D16 | TBC1 domain family, member 16 |
| 17055614 | 2.02 | 0.00161 | 0.133596 | TWIST1 | twist basic helix-loop-helix transcription factor 1 |
| 16887179 | 2.01 | 0.026471 | 0.397518 | CERS6; CERS6-AS1 | ceramide synthase 6; CERS6 antisense RNA 1 |
| 17109714 | 2.01 | 0.036511 | 0.445454 | SCARNA9L | small Cajal body-specific RNA 9-like |
| 16852982 | 2 | 0.002885 | 0.169559 | DOK6 | docking protein 6 |
| 16734339 | 2 | 0.026169 | 0.395412 | MIR4298 | microRNA 4298 |
| 17014442 | 2 | 0.011193 | 0.281236 | SLC22A3 | solute carrier family 22 (extraneuronal monoamine transporter), member 3 |
|  |  |  |  |  |  |
| **Down-regulated genes on day 21** | | | |  |  |
| Transcript Cluster ID | Fold Change (linear) (Induced vs. Control) | ANOVA p-value (Induced vs. Control) | FDR p-value (Induced vs. Control) | Gene Symbol | Description |
| 16715699 | -2 | 0.037126 | 0.448604 | C10orf55 | chromosome 10 open reading frame 55 |
| 16817692 | -2 | 0.021655 | 0.367726 | MVP | major vault protein; NULL |
| 16934045 | -2 | 0.025447 | 0.39161 | PIK3IP1 | phosphoinositide-3-kinase interacting protein 1; NULL |
| 17025191 | -2.01 | 0.007635 | 0.243316 | EZR | ezrin |
| 16683574 | -2.01 | 0.001199 | 0.120319 | STPG1 | sperm-tail PG-rich repeat containing 1 |
| 17118419 | -2.02 | 0.010942 | 0.278949 | CDC14B | cell division cycle 14B |
| 16978976 | -2.02 | 0.009328 | 0.262773 | CFI | complement factor I |
| 17017370 | -2.02 | 0.003249 | 0.17805 | DDAH2; CLIC1 | dimethylarginine dimethylaminohydrolase 2; chloride intracellular channel 1; NULL |
| 16972616 | -2.02 | 0.029105 | 0.411533 | NEIL3 | nei endonuclease VIII-like 3 (E. coli) |
| 16798938 | -2.02 | 0.010247 | 0.272418 | SCG5 | secretogranin V (7B2 protein); NULL |
| 17002612 | -2.02 | 0.003364 | 0.179209 | SLIT3 | slit homolog 3 (Drosophila) |
| 17050797 | -2.03 | 0.009555 | 0.264424 | CPED1 | cadherin-like and PC-esterase domain containing 1 |
| 16661141 | -2.03 | 0.000433 | 0.085609 | SH3BGRL3 | SH3 domain binding glutamic acid-rich protein like 3 |
| 17008105 | -2.03 | 0.005382 | 0.210321 | TBC1D22B | TBC1 domain family, member 22B |
| 16825638 | -2.03 | 0.001058 | 0.115806 | YPEL3 | yippee-like 3 (Drosophila); NULL |
| 17043882 | -2.04 | 0.000728 | 0.100117 | HDAC9 | histone deacetylase 9; NULL |
| 16748196 | -2.04 | 0.041403 | 0.463925 | LOC374443; OTTHUMG00000168360; RP11-705C15.2 | C-type lectin domain family 2, member D pseudogene; NULL |
| 16999421 | -2.05 | 0.021046 | 0.362745 | MARCH3 | membrane-associated ring finger (C3HC4) 3, E3 ubiquitin protein ligase |
| 16783764 | -2.05 | 0.004295 | 0.190894 | C14orf28 | chromosome 14 open reading frame 28 |
| 17013404 | -2.05 | 0.01284 | 0.295703 | RAB32 | RAB32, member RAS oncogene family |
| 17065938 | -2.05 | 0.008925 | 0.257617 | RNA5SP255 | RNA, 5S ribosomal pseudogene 255 |
| 17069816 | -2.05 | 0.032553 | 0.428033 | SULF1 | sulfatase 1; NULL |
| 17010625 | -2.05 | 0.022122 | 0.371464 | TPBG | trophoblast glycoprotein |
| 16830202 | -2.05 | 0.004671 | 0.196792 | XAF1 | XIAP associated factor 1; NULL |
| 16974121 | -2.06 | 0.000642 | 0.095249 | AFAP1 | actin filament associated protein 1 |
| 16981266 | -2.06 | 0.008193 | 0.249932 | DDX60L | DEAD (Asp-Glu-Ala-Asp) box polypeptide 60-like; NULL |
| 16966049 | -2.06 | 0.000052 | 0.031936 | KLHL5 | kelch-like family member 5 |
| 16733553 | -2.06 | 0.006292 | 0.224972 | NTM; LOC100653217 | neurotrimin; NULL; neurotrimin-like |
| 16961551 | -2.06 | 0.013434 | 0.300044 | PLD1 | phospholipase D1, phosphatidylcholine-specific; NULL |
| 17024775 | -2.06 | 0.011052 | 0.280082 | SYNE1 | spectrin repeat containing, nuclear envelope 1; NULL |
| 17052348 | -2.06 | 0.005233 | 0.20752 | TMEM178B | transmembrane protein 178B |
| 16701185 | -2.07 | 0.011228 | 0.281642 | CEP170; CEP170P1 | centrosomal protein 170kDa; centrosomal protein 170kDa pseudogene 1; NULL |
| 17089119 | -2.07 | 0.004868 | 0.19962 | PBX3 | pre-B-cell leukemia homeobox 3; NULL |
| 16968314 | -2.07 | 0.019572 | 0.35258 | PRDM8 | PR domain containing 8; NULL |
| 16911835 | -2.07 | 0.028286 | 0.406889 | RIN2 | Ras and Rab interactor 2; NULL |
| 16683300 | -2.07 | 0.035345 | 0.4408 | TCEA3 | transcription elongation factor A (SII), 3 |
| 16789723 | -2.08 | 0.00602 | 0.222127 | CRIP2 | cysteine-rich protein 2; NULL |
| 16944665 | -2.08 | 0.015686 | 0.31857 | DTX3L | deltex 3-like (Drosophila) |
| 16812738 | -2.08 | 0.000462 | 0.086081 | HDGFRP3; OTTHUMG00000172685; RP11-382A20.3 | hepatoma-derived growth factor, related protein 3; NULL |
| 16867414 | -2.08 | 0.044802 | 0.471504 | PLIN3 | perilipin 3 |
| 16960149 | -2.08 | 0.000494 | 0.088112 | PLSCR4 | phospholipid scramblase 4; NULL |
| 16670479 | -2.09 | 0.001727 | 0.138553 | C1orf51 | chromosome 1 open reading frame 51; NULL |
| 16863753 | -2.09 | 0.016238 | 0.324156 | EMP3 | epithelial membrane protein 3 |
| 16980470 | -2.09 | 0.004551 | 0.194829 | NR3C2 | nuclear receptor subfamily 3, group C, member 2; NULL |
| 16931766 | -2.1 | 0.021281 | 0.365228 | KLHDC7B | kelch domain containing 7B |
| 16918351 | -2.11 | 0.001146 | 0.119127 | COMMD7 | COMM domain containing 7 |
| 17098506 | -2.12 | 0.006438 | 0.226577 | FAM129B | family with sequence similarity 129, member B; NULL |
| 16729789 | -2.12 | 0.004813 | 0.198539 | PRSS23 | protease, serine, 23; NULL |
| 16897834 | -2.13 | 0.000287 | 0.070736 | EFEMP1 | EGF containing fibulin-like extracellular matrix protein 1; NULL |
| 16815498 | -2.13 | 0.006302 | 0.224972 | GLIS2 | GLIS family zinc finger 2 |
| 16853277 | -2.13 | 0.047321 | 0.477526 | NFATC1 | nuclear factor of activated T-cells, cytoplasmic, calcineurin-dependent 1; NULL |
| 16872705 | -2.13 | 0.002153 | 0.149314 | POU2F2 | POU class 2 homeobox 2; NULL |
| 17043529 | -2.14 | 0.002007 | 0.144922 | C1GALT1 | core 1 synthase, glycoprotein-N-acetylgalactosamine 3-beta-galactosyltransferase, 1; NULL |
| 16987531 | -2.14 | 0.029068 | 0.411473 | ERAP2 | endoplasmic reticulum aminopeptidase 2; NULL |
| 17019190 | -2.15 | 0.001651 | 0.13533 | C6orf132 | chromosome 6 open reading frame 132 |
| 16972167 | -2.15 | 0.013833 | 0.303779 | CPE | carboxypeptidase E |
| 16684674 | -2.15 | 0.005069 | 0.204615 | MTMR9LP | myotubularin related protein 9-like, pseudogene; NULL |
| 16778274 | -2.15 | 0.036353 | 0.444834 | TRPC4 | transient receptor potential cation channel, subfamily C, member 4; NULL |
| 16957396 | -2.16 | 0.008043 | 0.248472 | CCDC80 | coiled-coil domain containing 80 |
| 17067941 | -2.16 | 0.004227 | 0.190679 | GPR124 | G protein-coupled receptor 124 |
| 16725664 | -2.16 | 0.036762 | 0.447356 | RPLP0P2 | ribosomal protein, large, P0 pseudogene 2 |
| 17059872 | -2.16 | 0.000343 | 0.078792 | SGCE | sarcoglycan, epsilon |
| 16906571 | -2.16 | 0.029906 | 0.415086 | STAT4 | signal transducer and activator of transcription 4; NULL |
| 17048879 | -2.17 | 0.002755 | 0.166893 | ARPC1B | actin related protein 2/3 complex, subunit 1B, 41kDa; NULL |
| 16742202 | -2.17 | 0.011764 | 0.285531 | ARRB1 | arrestin, beta 1; NULL |
| 16773840 | -2.17 | 0.01882 | 0.345355 | BRCA2 | breast cancer 2, early onset |
| 16812598 | -2.17 | 0.007728 | 0.243316 | CPEB1; OTTHUMG00000172875; RP11-152F13.10 | cytoplasmic polyadenylation element binding protein 1; NULL |
| 16817254 | -2.17 | 0.040547 | 0.460977 | IL4R | interleukin 4 receptor; NULL |
| 16951696 | -2.17 | 0.000764 | 0.101165 | NEK10 | NIMA-related kinase 10; NULL |
| 16898362 | -2.17 | 0.038997 | 0.456501 | SERTAD2 | SERTA domain containing 2 |
| 16683445 | -2.18 | 0.003361 | 0.179209 | FUCA1 | fucosidase, alpha-L- 1, tissue |
| 16907572 | -2.18 | 0.031767 | 0.423671 | GPR1 | G protein-coupled receptor 1; NULL |
| 17043982 | -2.18 | 0.043753 | 0.468379 | ITGB8 | integrin, beta 8 |
| 17073259 | -2.18 | 0.002175 | 0.150063 | LY6E | lymphocyte antigen 6 complex, locus E; NULL |
| 16775763 | -2.18 | 0.025262 | 0.391222 | MIR622 | microRNA 622 |
| 17106357 | -2.18 | 0.000242 | 0.064748 | PLS3 | plastin 3; NULL |
| 16676526 | -2.19 | 0.002379 | 0.156069 | FAM72A; LOC101060656; FAM72C | family with sequence similarity 72, member A; protein FAM72D-like; family with sequence similarity 72, member C; NULL |
| 16784098 | -2.19 | 0.0286 | 0.407469 | FRMD6 | FERM domain containing 6; NULL |
| 16968797 | -2.19 | 0.01485 | 0.312334 | HERC3 | HECT and RLD domain containing E3 ubiquitin protein ligase 3; NULL |
| 16664005 | -2.19 | 0.016491 | 0.326883 | PLK3 | polo-like kinase 3 |
| 16922495 | -2.2 | 0.029412 | 0.411783 | CBR3 | carbonyl reductase 3 |
| 16925602 | -2.2 | 0.021491 | 0.366452 | ERG | v-ets erythroblastosis virus E26 oncogene homolog (avian); NULL |
| 16996146 | -2.21 | 0.038878 | 0.456047 | ESM1 | endothelial cell-specific molecule 1 |
| 16958124 | -2.21 | 0.004152 | 0.189638 | PARP9 | poly (ADP-ribose) polymerase family, member 9 |
| 16726081 | -2.21 | 0.038166 | 0.454198 | RARRES3 | retinoic acid receptor responder (tazarotene induced) 3 |
| 17042925 | -2.22 | 0.027874 | 0.404406 | ELFN1 | extracellular leucine-rich repeat and fibronectin type III domain containing 1 |
| 16714135 | -2.22 | 0.030298 | 0.417701 | ERCC6; ERCC6-PGBD3; PGBD3; OTTHUMG00000171334; RP11-123B3.6 | excision repair cross-complementing rodent repair deficiency, complementation group 6; ERCC6-PGBD3 readthrough; piggyBac transposable element derived 3; NULL |
| 16802960 | -2.22 | 0.010206 | 0.271775 | ISLR | immunoglobulin superfamily containing leucine-rich repeat |
| 16722412 | -2.22 | 0.001617 | 0.133779 | NUCB2 | nucleobindin 2; NULL |
| 16875518 | -2.23 | 0.001645 | 0.13533 | CDC42EP5 | CDC42 effector protein (Rho GTPase binding) 5; NULL |
| 16995645 | -2.23 | 0.000298 | 0.073067 | DAB2 | Dab, mitogen-responsive phosphoprotein, homolog 2 (Drosophila); NULL |
| 16761631 | -2.23 | 0.01014 | 0.270651 | DUSP16 | dual specificity phosphatase 16 |
| 16833567 | -2.24 | 0.020073 | 0.355977 | DUSP14 | dual specificity phosphatase 14 |
| 16851383 | -2.24 | 0.020365 | 0.358204 | GATA6 | GATA binding protein 6 |
| 16955291 | -2.25 | 0.046042 | 0.473737 | ARHGEF3 | Rho guanine nucleotide exchange factor (GEF) 3; NULL |
| 16989897 | -2.25 | 0.006781 | 0.232581 | CXXC5 | CXXC finger protein 5; NULL |
| 16800229 | -2.25 | 0.000996 | 0.112259 | MAP1A | microtubule-associated protein 1A |
| 17019218 | -2.26 | 0.004845 | 0.199452 | TRERF1 | transcriptional regulating factor 1 |
| 16755498 | -2.27 | 0.000023 | 0.021303 | TMPO | thymopoietin; NULL |
| 17066018 | -2.27 | 0.000361 | 0.078792 | ZDHHC2 | zinc finger, DHHC-type containing 2 |
| 16901393 | -2.28 | 0.003075 | 0.175065 | FHL2 | four and a half LIM domains 2 |
| 16859795 | -2.28 | 0.008112 | 0.249435 | GDF15 | growth differentiation factor 15 |
| 16824132 | -2.28 | 0.005843 | 0.2203 | NTAN1 | N-terminal asparagine amidase; NULL |
| 17087308 | -2.28 | 0.003484 | 0.179613 | TDRD7 | tudor domain containing 7 |
| 17012546 | -2.28 | 0.030518 | 0.418715 | TMEM200A | transmembrane protein 200A |
| 16727570 | -2.29 | 0.002235 | 0.151815 | C11orf80 | chromosome 11 open reading frame 80; NULL |
| 16707180 | -2.29 | 0.0477 | 0.478191 | IFIT2 | interferon-induced protein with tetratricopeptide repeats 2 |
| 16665796 | -2.29 | 0.038794 | 0.45601 | SGIP1 | SH3-domain GRB2-like (endophilin) interacting protein 1; NULL |
| 17060167 | -2.29 | 0.036415 | 0.444938 | TMEM130 | transmembrane protein 130; NULL |
| 16724471 | -2.3 | 0.000754 | 0.10075 | DDB2 | damage-specific DNA binding protein 2, 48kDa |
| 16834931 | -2.3 | 0.001041 | 0.115116 | FMNL1 | formin-like 1 |
| 17010692 | -2.3 | 0.001586 | 0.133008 | RWDD2A | RWD domain containing 2A |
| 16960844 | -2.3 | 0.001281 | 0.124036 | VEPH1 | ventricular zone expressed PH domain-containing 1; NULL |
| 16705089 | -2.31 | 0.005389 | 0.210427 | BICC1 | bicaudal C homolog 1 (Drosophila) |
| 16902945 | -2.31 | 0.000275 | 0.06946 | NCKAP5 | NCK-associated protein 5 |
| 16787814 | -2.32 | 0.0489 | 0.481174 | IFI27 | interferon, alpha-inducible protein 27; NULL |
| 16948021 | -2.33 | 0.044397 | 0.470454 | ECT2 | epithelial cell transforming sequence 2 oncogene; NULL |
| 16745470 | -2.33 | 0.000174 | 0.057812 | MIR100HG | mir-100-let-7a-2 cluster host gene (non-protein coding) |
| 16991472 | -2.33 | 0.012862 | 0.295939 | SGCD | sarcoglycan, delta (35kDa dystrophin-associated glycoprotein) |
| 16833060 | -2.34 | 0.002342 | 0.154574 | LRRC37B | leucine rich repeat containing 37B; NULL |
| 16751319 | -2.34 | 0.00213 | 0.148687 | SCN8A | sodium channel, voltage gated, type VIII, alpha subunit |
| 16786010 | -2.34 | 0.039538 | 0.457048 | SIPA1L1 | signal-induced proliferation-associated 1 like 1; NULL |
| 16858970 | -2.35 | 0.020412 | 0.358715 | CD97 | CD97 molecule |
| 17000858 | -2.35 | 0.009757 | 0.266441 | DIAPH1 | diaphanous-related formin 1; NULL |
| 16684800 | -2.35 | 0.000168 | 0.056148 | TMEM54 | transmembrane protein 54; NULL |
| 16818842 | -2.36 | 0.006219 | 0.224015 | CYLD | cylindromatosis (turban tumor syndrome); NULL |
| 17015637 | -2.36 | 0.004159 | 0.18974 | ELOVL2 | ELOVL fatty acid elongase 2 |
| 16962022 | -2.36 | 0.018238 | 0.340359 | LAMP3 | lysosomal-associated membrane protein 3 |
| 16981219 | -2.37 | 0.009771 | 0.266441 | DDX60 | DEAD (Asp-Glu-Ala-Asp) box polypeptide 60 |
| 16732755 | -2.37 | 0.013887 | 0.304269 | GRAMD1B | GRAM domain containing 1B; NULL |
| 16809748 | -2.37 | 0.000263 | 0.067375 | MNS1 | meiosis-specific nuclear structural 1 |
| 16960922 | -2.37 | 0.005302 | 0.208916 | RARRES1 | retinoic acid receptor responder (tazarotene induced) 1 |
| 16924878 | -2.37 | 0.001824 | 0.141171 | TIAM1 | T-cell lymphoma invasion and metastasis 1 |
| 16986913 | -2.37 | 0.004753 | 0.197469 | VCAN | versican |
| 16836896 | -2.38 | 0.023233 | 0.379855 | ACE; OTTHUMG00000178900; CTD-2501B8.1; ACE3P | angiotensin I converting enzyme; NULL; angiotensin I converting enzyme (peptidyl-dipeptidase A) 3, pseudogene |
| 16718047 | -2.38 | 0.017815 | 0.337044 | CALHM2 | calcium homeostasis modulator 2 |
| 16767851 | -2.38 | 0.01572 | 0.31871 | E2F7 | E2F transcription factor 7; NULL |
| 16808401 | -2.38 | 0.000371 | 0.079448 | FRMD5 | FERM domain containing 5; NULL |
| 16740828 | -2.4 | 0.001283 | 0.124036 | CTSF | cathepsin F; NULL |
| 17070061 | -2.4 | 0.006372 | 0.225536 | LY96 | lymphocyte antigen 96 |
| 16970080 | -2.41 | 0.007132 | 0.237862 | CEP170P1 | centrosomal protein 170kDa pseudogene 1 |
| 16692667 | -2.41 | 0.003644 | 0.181367 | MTMR11 | myotubularin related protein 11; NULL |
| 17059776 | -2.41 | 0.000193 | 0.061132 | SAMD9L | sterile alpha motif domain containing 9-like |
| 17094064 | -2.41 | 0.006325 | 0.225271 | SHB | Src homology 2 domain containing adaptor protein B |
| 16962380 | -2.42 | 0.027874 | 0.404406 | ETV5 | ets variant 5; NULL |
| 16798951 | -2.42 | 0.025001 | 0.390712 | GREM1 | gremlin 1, DAN family BMP antagonist |
| 16693474 | -2.42 | 0.010495 | 0.27453 | S100A16 | S100 calcium binding protein A16; NULL |
| 16762661 | -2.43 | 0.037352 | 0.449858 | PTHLH | parathyroid hormone-like hormone |
| 16816542 | -2.44 | 0.024445 | 0.388309 | IQCK | IQ motif containing K; NULL |
| 17078870 | -2.44 | 0.006374 | 0.225536 | MMP16 | matrix metallopeptidase 16 (membrane-inserted) |
| 16664828 | -2.44 | 0.029131 | 0.411533 | PODN | podocan; NULL |
| 16670894 | -2.44 | 0.018711 | 0.344288 | TUFT1 | tuftelin 1 |
| 16958403 | -2.46 | 0.003183 | 0.176407 | HEG1 | heart development protein with EGF-like domains 1 |
| 16768738 | -2.46 | 0.0017 | 0.136624 | NTN4 | netrin 4 |
| 16668002 | -2.47 | 0.022918 | 0.376698 | FAM102B | family with sequence similarity 102, member B |
| 16852025 | -2.47 | 0.027555 | 0.402932 | FHOD3 | formin homology 2 domain containing 3 |
| 16847949 | -2.48 | 0.000632 | 0.094971 | CEP112 | centrosomal protein 112kDa; NULL |
| 16672390 | -2.48 | 0.018088 | 0.338799 | IFI16 | interferon, gamma-inducible protein 16; NULL |
| 16680790 | -2.48 | 0.005194 | 0.206885 | MEGF6 | multiple EGF-like-domains 6; NULL |
| 16888912 | -2.48 | 0.000215 | 0.06303 | MYO1B | myosin IB; NULL |
| 17056426 | -2.48 | 0.013614 | 0.30214 | PDE1C | phosphodiesterase 1C, calmodulin-dependent 70kDa |
| 16820508 | -2.48 | 0.000991 | 0.112139 | TANGO6; TMCO7 | transport and golgi organization 6 homolog (Drosophila); NULL |
| 16692724 | -2.5 | 0.000036 | 0.027635 | ANP32E | acidic (leucine-rich) nuclear phosphoprotein 32 family, member E |
| 16944695 | -2.5 | 0.00316 | 0.175932 | PARP14 | poly (ADP-ribose) polymerase family, member 14 |
| 16721593 | -2.5 | 0.00035 | 0.078792 | PPFIBP2 | PTPRF interacting protein, binding protein 2 (liprin beta 2); NULL |
| 16956714 | -2.51 | 0.032558 | 0.428033 | DCBLD2 | discoidin, CUB and LCCL domain containing 2; NULL |
| 16805345 | -2.52 | 0.039924 | 0.458537 | MCTP2 | multiple C2 domains, transmembrane 2 |
| 16854904 | -2.53 | 0.018246 | 0.340359 | PSTPIP2 | proline-serine-threonine phosphatase interacting protein 2 |
| 17019820 | -2.53 | 0.030047 | 0.415609 | PTCHD4 | patched domain containing 4 |
| 16689384 | -2.54 | 0.008488 | 0.252979 | GBP4 | guanylate binding protein 4 |
| 16705507 | -2.54 | 0.037487 | 0.450383 | SRGN | serglycin |
| 16768149 | -2.55 | 0.002743 | 0.166375 | RASSF9 | Ras association (RalGDS/AF-6) domain family (N-terminal) member 9 |
| 16739192 | -2.56 | 0.019977 | 0.355977 | RAB3IL1 | RAB3A interacting protein (rabin3)-like 1; NULL |
| 16677451 | -2.58 | 0.007552 | 0.243168 | KCNK2 | potassium channel, subfamily K, member 2; NULL |
| 16928533 | -2.59 | 0.000133 | 0.050589 | ASPHD2 | aspartate beta-hydroxylase domain containing 2 |
| 16705159 | -2.59 | 0.039366 | 0.456858 | CDK1 | cyclin-dependent kinase 1 |
| 16987610 | -2.61 | 0.021875 | 0.369489 | RGMB | RGM domain family, member B; NULL |
| 16803710 | -2.63 | 0.006307 | 0.224972 | ARNT2 | aryl-hydrocarbon receptor nuclear translocator 2; NULL |
| 16847432 | -2.63 | 0.031157 | 0.422303 | BRIP1 | BRCA1 interacting protein C-terminal helicase 1 |
| 16904365 | -2.63 | 0.006497 | 0.227552 | IFIH1 | interferon induced with helicase C domain 1 |
| 16763295 | -2.63 | 0.000378 | 0.080081 | PRICKLE1 | prickle homolog 1 (Drosophila); NULL |
| 16823692 | -2.64 | 0.000326 | 0.077065 | NAGPA | N-acetylglucosamine-1-phosphodiester alpha-N-acetylglucosaminidase; NULL |
| 16809596 | -2.64 | 0.013142 | 0.297962 | RAB27A | RAB27A, member RAS oncogene family |
| 16782862 | -2.65 | 0.001235 | 0.121781 | NYNRIN | NYN domain and retroviral integrase containing |
| 16697196 | -2.67 | 0.018937 | 0.346595 | FAM129A | family with sequence similarity 129, member A |
| 16798919 | -2.68 | 0.013992 | 0.305362 | ARHGAP11A | Rho GTPase activating protein 11A |
| 16823750 | -2.68 | 0.002654 | 0.165138 | CARHSP1 | calcium regulated heat stable protein 1, 24kDa; NULL |
| 16979339 | -2.68 | 0.025743 | 0.39292 | PDE5A | phosphodiesterase 5A, cGMP-specific; NULL |
| 16984730 | -2.69 | 0.00725 | 0.239417 | FST | follistatin |
| 16968735 | -2.69 | 0.030607 | 0.419336 | HERC6 | HECT and RLD domain containing E3 ubiquitin protein ligase family member 6; NULL |
| 16945768 | -2.71 | 0.00016 | 0.05539 | CCRL1 | chemokine (C-C motif) receptor-like 1 |
| 17062985 | -2.71 | 0.000766 | 0.101165 | PODXL | podocalyxin-like; NULL |
| 16778241 | -2.71 | 0.017661 | 0.336241 | POSTN | periostin, osteoblast specific factor; NULL |
| 16970404 | -2.76 | 0.006297 | 0.224972 | FGF2 | fibroblast growth factor 2 (basic) |
| 16723614 | -2.78 | 0.048814 | 0.480936 | CD44 | CD44 molecule (Indian blood group); NULL |
| 16682098 | -2.78 | 0.000438 | 0.085609 | EPHA2 | EPH receptor A2 |
| 16768675 | -2.78 | 0.009283 | 0.26253 | FGD6 | FYVE, RhoGEF and PH domain containing 6 |
| 16774427 | -2.78 | 0.001191 | 0.120248 | LACC1; CCDC122 | laccase (multicopper oxidoreductase) domain containing 1; coiled-coil domain containing 122 |
| 16884523 | -2.78 | 0.018321 | 0.340989 | SLC20A1 | solute carrier family 20 (phosphate transporter), member 1 |
| 16938271 | -2.8 | 0.001392 | 0.127922 | KAT2B | K(lysine) acetyltransferase 2B; NULL |
| 16862439 | -2.81 | 0.000456 | 0.085888 | AXL | AXL receptor tyrosine kinase |
| 16844061 | -2.81 | 0.039503 | 0.456858 | PLXDC1 | plexin domain containing 1; NULL |
| 16707196 | -2.83 | 0.002394 | 0.156672 | IFIT1 | interferon-induced protein with tetratricopeptide repeats 1 |
| 16740412 | -2.84 | 0.003856 | 0.185576 | LTBP3 | latent transforming growth factor beta binding protein 3; NULL |
| 16899413 | -2.85 | 0.00056 | 0.091471 | EVA1A | eva-1 homolog A (C. elegans); NULL |
| 16859314 | -2.86 | 0.028412 | 0.406949 | KLF2 | Kruppel-like factor 2 (lung) |
| 16892075 | -2.87 | 0.000505 | 0.088388 | ARMC9 | armadillo repeat containing 9; NULL |
| 16660282 | -2.89 | 0.021404 | 0.366407 | PLA2G5 | phospholipase A2, group V; NULL |
| 16789149 | -2.89 | 0.006955 | 0.234881 | TNFAIP2 | tumor necrosis factor, alpha-induced protein 2; NULL |
| 16813342 | -2.9 | 0.037134 | 0.448604 | PRC1 | protein regulator of cytokinesis 1; NULL |
| 16803743 | -2.91 | 0.00198 | 0.144028 | ABHD17C | abhydrolase domain containing 17C |
| 16942648 | -2.91 | 0.00762 | 0.243316 | GXYLT2 | glucoside xylosyltransferase 2 |
| 16971966 | -2.92 | 0.015089 | 0.314737 | FNIP2 | folliculin interacting protein 2 |
| 17114829 | -2.92 | 0.000251 | 0.065754 | IDS; OTTHUMG00000022618; AF011889.5 | iduronate 2-sulfatase; NULL |
| 17007910 | -2.94 | 0.002106 | 0.14784 | MAPK13 | mitogen-activated protein kinase 13 |
| 16689354 | -2.99 | 0.000853 | 0.103688 | GBP2 | guanylate binding protein 2, interferon-inducible; NULL |
| 16779435 | -2.99 | 0.005227 | 0.207454 | THSD1 | thrombospondin, type I, domain containing 1 |
| 16834409 | -3.02 | 0.017883 | 0.337774 | CNTNAP1 | contactin associated protein 1 |
| 16754373 | -3.03 | 0.002263 | 0.153076 | GLIPR1 | GLI pathogenesis-related 1 |
| 16672214 | -3.03 | 0.014659 | 0.31049 | PEAR1 | platelet endothelial aggregation receptor 1; NULL |
| 17096904 | -3.04 | 0.002125 | 0.148509 | CTNNAL1 | catenin (cadherin-associated protein), alpha-like 1 |
| 16839177 | -3.04 | 0.027614 | 0.403335 | METRNL | meteorin, glial cell differentiation regulator-like |
| 16811638 | -3.04 | 0.00579 | 0.219245 | SEMA7A | semaphorin 7A, GPI membrane anchor (John Milton Hagen blood group) |
| 17055937 | -3.05 | 0.000304 | 0.073966 | OSBPL3 | oxysterol binding protein-like 3; NULL |
| 16694701 | -3.06 | 0.006172 | 0.223696 | CRABP2 | cellular retinoic acid binding protein 2 |
| 17087588 | -3.06 | 0.001204 | 0.120387 | TMEFF1; MSANTD3-TMEFF1; MSANTD3 | transmembrane protein with EGF-like and two follistatin-like domains 1; MSANTD3-TMEFF1 readthrough; Myb/SANT-like DNA-binding domain containing 3 |
| 16956149 | -3.07 | 0.000921 | 0.107035 | FOXP1 | forkhead box P1; NULL |
| 16818773 | -3.1 | 0.001012 | 0.113201 | ADCY7 | adenylate cyclase 7; NULL |
| 16688799 | -3.1 | 0.008376 | 0.252306 | ELTD1 | EGF, latrophilin and seven transmembrane domain containing 1 |
| 16847841 | -3.11 | 0.000043 | 0.029872 | SMURF2 | SMAD specific E3 ubiquitin protein ligase 2; NULL |
| 16723662 | -3.14 | 0.02847 | 0.406949 | FJX1 | four jointed box 1 (Drosophila) |
| 16679301 | -3.15 | 0.002672 | 0.165434 | FMN2 | formin 2; NULL |
| 16968213 | -3.16 | 0.005266 | 0.208174 | ANXA3 | annexin A3; NULL |
| 16821377 | -3.16 | 0.000228 | 0.063075 | CDH13 | cadherin 13, H-cadherin (heart); NULL |
| 16658536 | -3.16 | 0.000041 | 0.029698 | PER3 | period circadian clock 3 |
| 17066224 | -3.16 | 0.000654 | 0.095888 | SH2D4A | SH2 domain containing 4A |
| 16865699 | -3.18 | 0.036232 | 0.444723 | SSC5D | scavenger receptor cysteine rich domain containing (5 domains) |
| 16903771 | -3.19 | 0.000725 | 0.100117 | CACNB4 | calcium channel, voltage-dependent, beta 4 subunit |
| 16767794 | -3.19 | 0.000044 | 0.03027 | OSBPL8 | oxysterol binding protein-like 8; NULL |
| 16889563 | -3.2 | 0.003627 | 0.181198 | FZD7 | frizzled family receptor 7 |
| 16915245 | -3.23 | 0.016686 | 0.328093 | APCDD1L-AS1 | APCDD1L antisense RNA 1 (head to head) |
| 16977286 | -3.23 | 0.001119 | 0.118715 | PRKG2 | protein kinase, cGMP-dependent, type II; NULL |
| 17057478 | -3.24 | 0.008009 | 0.247761 | IGFBP3 | insulin-like growth factor binding protein 3; NULL |
| 16909958 | -3.28 | 0.021966 | 0.369819 | PER2 | period circadian clock 2 |
| 16985229 | -3.28 | 0.014825 | 0.312198 | RGS7BP | regulator of G-protein signaling 7 binding protein |
| 16747184 | -3.29 | 0.000101 | 0.046169 | CD9 | CD9 molecule |
| 16807763 | -3.32 | 0.014296 | 0.307438 | EHD4 | EH-domain containing 4 |
| 16693976 | -3.32 | 0.000401 | 0.08302 | MUC1 | mucin 1, cell surface associated; NULL |
| 16929442 | -3.34 | 0.00283 | 0.169272 | TIMP3 | TIMP metallopeptidase inhibitor 3 |
| 16815310 | -3.35 | 0.049999 | 0.484527 | TNFRSF12A | tumor necrosis factor receptor superfamily, member 12A |
| 16702524 | -3.37 | 0.047486 | 0.47789 | CAMK1D | calcium/calmodulin-dependent protein kinase ID |
| 16764724 | -3.37 | 0.009798 | 0.266441 | SMAGP | small cell adhesion glycoprotein |
| 16722081 | -3.39 | 0.003094 | 0.175763 | MICAL2 | microtubule associated monooxygenase, calponin and LIM domain containing 2; NULL |
| 16769159 | -3.4 | 0.0012 | 0.120319 | GNPTAB | N-acetylglucosamine-1-phosphate transferase, alpha and beta subunits |
| 16719171 | -3.44 | 0.01345 | 0.300044 | CPXM2 | carboxypeptidase X (M14 family), member 2 |
| 16773759 | -3.48 | 0.01577 | 0.318745 | FRY | furry homolog (Drosophila) |
| 17077826 | -3.48 | 0.005887 | 0.220782 | MYBL1; LOC645895 | v-myb myeloblastosis viral oncogene homolog (avian)-like 1; uncharacterized LOC645895 |
| 16751655 | -3.49 | 0.006334 | 0.225277 | IGFBP6 | insulin-like growth factor binding protein 6 |
| 16842266 | -3.53 | 0.000584 | 0.093321 | MFAP4 | microfibrillar-associated protein 4 |
| 16802653 | -3.53 | 0.002045 | 0.145657 | THSD4 | thrombospondin, type I, domain containing 4 |
| 16723422 | -3.55 | 0.001054 | 0.115806 | KIAA1549L | KIAA1549-like |
| 16797196 | -3.56 | 0.004503 | 0.194361 | AHNAK2 | AHNAK nucleoprotein 2 |
| 16764398 | -3.57 | 0.000933 | 0.107873 | FMNL3 | formin-like 3; NULL |
| 17056506 | -3.57 | 0.000103 | 0.046189 | RP9P | retinitis pigmentosa 9 pseudogene |
| 16979225 | -3.58 | 0.015839 | 0.319478 | PRSS12 | protease, serine, 12 (neurotrypsin, motopsin) |
| 16785789 | -3.59 | 0.006843 | 0.232909 | GALNT16; GALNTL1 | UDP-N-acetyl-alpha-D-galactosamine:polypeptide N-acetylgalactosaminyltransferase 16; NULL |
| 16675558 | -3.63 | 0.002009 | 0.144922 | NEK7 | NIMA-related kinase 7; NULL |
| 16802519 | -3.64 | 0.011827 | 0.285615 | KIF23 | kinesin family member 23 |
| 16843167 | -3.65 | 0.018133 | 0.339333 | EVI2A | ecotropic viral integration site 2A |
| 16788036 | -3.66 | 0.025584 | 0.392261 | BDKRB1 | bradykinin receptor B1 |
| 16749459 | -3.67 | 0.005582 | 0.215713 | PPFIBP1 | PTPRF interacting protein, binding protein 1 (liprin beta 1); NULL |
| 16707551 | -3.68 | 0.04457 | 0.471203 | CEP55 | centrosomal protein 55kDa |
| 16811085 | -3.68 | 0.014554 | 0.31048 | ITGA11 | integrin, alpha 11 |
| 17005138 | -3.7 | 0.007568 | 0.243168 | CAP2 | CAP, adenylate cyclase-associated protein, 2 (yeast) |
| 17004903 | -3.73 | 0.00777 | 0.243657 | EDN1 | endothelin 1 |
| 16818114 | -3.73 | 0.010311 | 0.273076 | HSD3B7 | hydroxy-delta-5-steroid dehydrogenase, 3 beta- and steroid delta-isomerase 7 |
| 16749423 | -3.77 | 0.01071 | 0.277259 | ARNTL2 | aryl hydrocarbon receptor nuclear translocator-like 2 |
| 16848123 | -3.8 | 0.002875 | 0.169559 | ABCA8 | ATP-binding cassette, sub-family A (ABC1), member 8; NULL |
| 16979985 | -3.83 | 0.003341 | 0.179209 | MGARP; NDUFC1 | mitochondria-localized glutamic acid-rich protein; NADH dehydrogenase (ubiquinone) 1, subcomplex unknown, 1, 6kDa |
| 16697544 | -3.85 | 0.03212 | 0.425454 | ASPM | asp (abnormal spindle) homolog, microcephaly associated (Drosophila) |
| 16851768 | -3.91 | 0.012713 | 0.294468 | DSG2 | desmoglein 2 |
| 16742384 | -3.93 | 0.009869 | 0.267072 | LRRC32 | leucine rich repeat containing 32 |
| 17000650 | -3.94 | 0.000026 | 0.022806 | TMEM173 | transmembrane protein 173; NULL |
| 16728261 | -3.95 | 0.031202 | 0.422689 | CCND1 | cyclin D1 |
| 16909482 | -3.96 | 0.017437 | 0.334203 | HTR2B | 5-hydroxytryptamine (serotonin) receptor 2B, G protein-coupled |
| 16965252 | -3.97 | 0.000543 | 0.090315 | BST1 | bone marrow stromal cell antigen 1 |
| 17007543 | -4.05 | 0.003978 | 0.18621 | ITPR3 | inositol 1,4,5-trisphosphate receptor, type 3 |
| 16717272 | -4.11 | 0.00019 | 0.061125 | LOXL4 | lysyl oxidase-like 4 |
| 16693996 | -4.12 | 0.002066 | 0.146122 | THBS3 | thrombospondin 3; NULL |
| 16685201 | -4.14 | 0.007593 | 0.243168 | COL8A2 | collagen, type VIII, alpha 2 |
| 16682333 | -4.16 | 0.000342 | 0.078792 | MFAP2 | microfibrillar-associated protein 2; NULL |
| 16784760 | -4.2 | 0.006294 | 0.224972 | DACT1 | dishevelled-binding antagonist of beta-catenin 1 |
| 17056984 | -4.21 | 0.040509 | 0.460879 | INHBA | inhibin, beta A |
| 16931237 | -4.22 | 0.038868 | 0.456047 | FBLN1 | fibulin 1; NULL |
| 16984689 | -4.27 | 0.027864 | 0.404406 | ITGA2 | integrin, alpha 2 (CD49B, alpha 2 subunit of VLA-2 receptor); NULL |
| 16859763 | -4.37 | 0.001177 | 0.120248 | IFI30; PIK3R2 | interferon, gamma-inducible protein 30; phosphoinositide-3-kinase, regulatory subunit 2 (beta) |
| 16914972 | -4.43 | 0.001808 | 0.140432 | DOK5 | docking protein 5 |
| 16713530 | -4.48 | 0.019479 | 0.352353 | CXCL12 | chemokine (C-X-C motif) ligand 12 |
| 17092870 | -4.58 | 0.001834 | 0.141171 | MIR31HG | MIR31 host gene (non-protein coding) |
| 16811249 | -4.63 | 0.000823 | 0.10258 | UACA | uveal autoantigen with coiled-coil domains and ankyrin repeats; NULL |
| 17063221 | -4.65 | 0.016897 | 0.330049 | FAM180A | family with sequence similarity 180, member A |
| 17016263 | -4.69 | 0.002502 | 0.160999 | CMAHP | cytidine monophospho-N-acetylneuraminic acid hydroxylase, pseudogene; NULL |
| 16802903 | -4.72 | 0.000396 | 0.08302 | LOXL1 | lysyl oxidase-like 1 |
| 16884956 | -4.73 | 0.032799 | 0.428765 | STEAP3 | STEAP family member 3, metalloreductase; NULL |
| 16909021 | -4.74 | 0.003813 | 0.18515 | SERPINE2 | serpin peptidase inhibitor, clade E (nexin, plasminogen activator inhibitor type 1), member 2; NULL |
| 16920156 | -4.75 | 0.001403 | 0.127922 | PTGIS | prostaglandin I2 (prostacyclin) synthase |
| 17096471 | -4.78 | 0.000471 | 0.086427 | TBC1D2 | TBC1 domain family, member 2 |
| 16968331 | -4.85 | 0.017029 | 0.331138 | FGF5 | fibroblast growth factor 5 |
| 16794719 | -4.93 | 0.002688 | 0.165536 | LTBP2 | latent transforming growth factor beta binding protein 2 |
| 16965519 | -4.94 | 0.006854 | 0.23309 | SOD3 | superoxide dismutase 3, extracellular |
| 17050591 | -4.96 | 0.015863 | 0.319524 | MET | met proto-oncogene (hepatocyte growth factor receptor) |
| 16967631 | -4.96 | 0.002348 | 0.154574 | SLC4A4 | solute carrier family 4, sodium bicarbonate cotransporter, member 4 |
| 16873060 | -4.98 | 0.044673 | 0.471203 | PLAUR | plasminogen activator, urokinase receptor; NULL |
| 16670574 | -5.04 | 0.000065 | 0.03616 | ECM1 | extracellular matrix protein 1; NULL |
| 16848097 | -5.07 | 0.005139 | 0.205718 | FAM20A | family with sequence similarity 20, member A; NULL |
| 16969686 | -5.08 | 0.001252 | 0.122742 | CCDC109B | coiled-coil domain containing 109B |
| 16909081 | -5.13 | 0.002951 | 0.171473 | DOCK10 | dedicator of cytokinesis 10; NULL |
| 16715793 | -5.15 | 0.000581 | 0.093071 | KCNMA1 | potassium large conductance calcium-activated channel, subfamily M, alpha member 1; NULL |
| 17083793 | -5.19 | 0.0009 | 0.10616 | ADAMTSL1 | ADAMTS-like 1; NULL |
| 17083370 | -5.25 | 0.000559 | 0.091471 | PDCD1LG2 | programmed cell death 1 ligand 2 |
| 16960911 | -5.31 | 0.003442 | 0.179209 | LXN | latexin |
| 17108003 | -5.33 | 0.006146 | 0.223546 | BGN | biglycan; NULL |
| 16874005 | -5.33 | 0.000059 | 0.032843 | DBP | D site of albumin promoter (albumin D-box) binding protein |
| 17049904 | -5.33 | 0.000059 | 0.032843 | LRRC17 | leucine rich repeat containing 17 |
| 16785127 | -5.36 | 0.00001 | 0.013154 | RHOJ | ras homolog family member J |
| 16779546 | -5.41 | 0.003418 | 0.179209 | DIAPH3 | diaphanous homolog 3 (Drosophila) |
| 17045622 | -5.72 | 0.000449 | 0.085609 | AEBP1; MIR4649 | AE binding protein 1; microRNA 4649; NULL |
| 16904667 | -5.89 | 0.00877 | 0.255244 | SCN9A | sodium channel, voltage-gated, type IX, alpha subunit; NULL |
| 16956792 | -6.1 | 0.000084 | 0.041712 | ABI3BP | ABI family, member 3 (NESH) binding protein |
| 16687123 | -6.1 | 0.001635 | 0.135038 | RAB3B | RAB3B, member RAS oncogene family |
| 16677071 | -6.16 | 0.014681 | 0.31049 | SERTAD4 | SERTA domain containing 4 |
| 17014114 | -6.31 | 0.003457 | 0.179209 | SYNJ2 | synaptojanin 2; NULL |
| 16755908 | -6.54 | 0.000217 | 0.06303 | DRAM1 | DNA-damage regulated autophagy modulator 1 |
| 16701037 | -6.67 | 0.02996 | 0.415122 | GREM2 | gremlin 2, DAN family BMP antagonist |
| 17040902 | -7.07 | 0.015868 | 0.319524 | C2 | complement component 2; NULL |
| 16767422 | -7.41 | 0.00036 | 0.078792 | PTPRB | protein tyrosine phosphatase, receptor type, B; NULL |
| 17033493 | -7.55 | 0.021396 | 0.366407 | C2; CFB | complement component 2; complement factor B; NULL |
| 17110401 | -7.67 | 0.001879 | 0.141171 | SLC9A7 | solute carrier family 9, subfamily A (NHE7, cation proton antiporter 7), member 7 |
| 17072135 | -7.68 | 0.028351 | 0.406889 | NOV | nephroblastoma overexpressed |
| 16697471 | -7.74 | 0.004574 | 0.194961 | B3GALT2 | UDP-Gal:betaGlcNAc beta 1,3-galactosyltransferase, polypeptide 2 |
| 17076726 | -7.79 | 0.009823 | 0.266441 | PLAT | plasminogen activator, tissue; NULL |
| 16855510 | -7.81 | 0.000015 | 0.01759 | ATP8B1 | ATPase, aminophospholipid transporter, class I, type 8B, member 1 |
| 17010760 | -8.05 | 0.000469 | 0.086427 | NT5E | 5'-nucleotidase, ecto (CD73); NULL |
| 17017641 | -8.13 | 0.029382 | 0.411783 | TNXB; TNXA; LOC101060681 | tenascin XB; tenascin XA (pseudogene); tenascin-X-like; NULL |
| 16974968 | -8.31 | 0.001601 | 0.133109 | SEL1L3 | sel-1 suppressor of lin-12-like 3 (C. elegans); NULL |
| 16706180 | -8.34 | 0.01165 | 0.284784 | PLAU | plasminogen activator, urokinase |
| 17001299 | -8.41 | 0.000117 | 0.048828 | DPYSL3 | dihydropyrimidinase-like 3; NULL |
| 16690704 | -8.51 | 0.000833 | 0.103258 | SLC16A4 | solute carrier family 16, member 4 (monocarboxylic acid transporter 5); NULL |
| 17062127 | -8.76 | 0.00078 | 0.101677 | WNT2 | wingless-type MMTV integration site family member 2 |
| 16904324 | -8.83 | 0.001194 | 0.120248 | FAP | fibroblast activation protein, alpha; NULL |
| 17006949 | -8.99 | 0.008206 | 0.249932 | CFB; OTTHUMG00000159600; XXbac-BPG116M5.17; C2 | complement factor B; NULL |
| 16759218 | -9.01 | 0.000165 | 0.055995 | GPR133 | G protein-coupled receptor 133; NULL |
| 17087430 | -9.18 | 0.000437 | 0.085609 | COL15A1 | collagen, type XV, alpha 1 |
| 16858137 | -9.82 | 0.001421 | 0.129085 | ICAM1 | intercellular adhesion molecule 1 |
| 16867784 | -10.07 | 0.000553 | 0.091277 | C3 | complement component 3; NULL |
| 17044177 | -10.36 | 0.001917 | 0.141171 | IL6 | interleukin 6 (interferon, beta 2); NULL |
| 16852179 | -11.09 | 0.021098 | 0.363117 | SLC14A1 | solute carrier family 14 (urea transporter), member 1 (Kidd blood group); NULL |
| 16853399 | -11.59 | 0.044691 | 0.471203 | COLEC12 | collectin sub-family member 12 |
| 16914084 | -11.84 | 0.009109 | 0.259369 | WISP2 | WNT1 inducible signaling pathway protein 2; NULL |
| 16743721 | -12.21 | 0.032872 | 0.428765 | MMP1 | matrix metallopeptidase 1 (interstitial collagenase) |
| 17108816 | -12.48 | 0.002933 | 0.17125 | MXRA5 | matrix-remodelling associated 5 |
| 16848173 | -12.54 | 0.020169 | 0.356679 | ABCA9 | ATP-binding cassette, sub-family A (ABC1), member 9; NULL |
| 17087413 | -12.9 | 0.000608 | 0.094788 | GALNT12 | UDP-N-acetyl-alpha-D-galactosamine:polypeptide N-acetylgalactosaminyltransferase 12 (GalNAc-T12) |
| 16904278 | -13.97 | 0.000099 | 0.045954 | DPP4 | dipeptidyl-peptidase 4; NULL |
| 17095887 | -14.59 | 0.000045 | 0.03027 | ASPN | asporin |
| 17084130 | -15.36 | 2.43E-07 | 0.000902 | TEK | TEK tyrosine kinase, endothelial |
| 17007982 | -17.05 | 0.005923 | 0.220911 | PI16 | peptidase inhibitor 16 |
| 17050765 | -17.86 | 0.000512 | 0.088388 | KCND2 | potassium voltage-gated channel, Shal-related subfamily, member 2 |
| 16721585 | -17.86 | 0.018481 | 0.342566 | OLFML1 | olfactomedin-like 1 |
| 17014257 | -19.62 | 0.001771 | 0.139581 | FNDC1 | fibronectin type III domain containing 1 |
| 17080486 | -23.95 | 0.000094 | 0.045026 | TNFRSF11B | tumor necrosis factor receptor superfamily, member 11b |
| 16737344 | -25.77 | 0.009377 | 0.263225 | PAMR1 | peptidase domain containing associated with muscle regeneration 1 |
| 16962911 | -25.95 | 0.000528 | 0.089052 | LRRC15 | leucine rich repeat containing 15 |
| 17072162 | -27.82 | 0.001806 | 0.140432 | COL14A1 | collagen, type XIV, alpha 1; NULL |
| 16803754 | -101.87 | 0.000009 | 0.012515 | KIAA1199 | KIAA1199; NULL |
